# Chiral phosphoric acid-catalyzed enantioselective construction of structurally diverse benzothiazolopyrimidines†
†Electronic supplementary information (ESI) available. CCDC 1815484 and 1851627. For ESI and crystallographic data in CIF or other electronic format see DOI: 10.1039/c8sc05581e


**DOI:** 10.1039/c8sc05581e

**Published:** 2019-02-07

**Authors:** Lucie Jarrige, Danijel Glavač, Guillaume Levitre, Pascal Retailleau, Guillaume Bernadat, Luc Neuville, Géraldine Masson

**Affiliations:** a Institut de Chimie des Substances Naturelles , CNRS UPR 2301 , Université Paris-Sud , Université Paris-Saclay , 1, Avenue de la Terrasse, 91198 Gif-sur-Yvette Cedex , France . Email: geraldine.masson@cnrs.fr; b Ruđer Bošković Institute , Division of Organic Chemistry and Biochemistry , Bijenička cesta 54 , 10000 Zagreb , Croatia; c Laboratoire Chimie Thérapeutique , Faculté de Pharmacie Biocis 8076 , LabEx LERMIT , 5, rue J. B. Clément, 92296 Châtenay Malabry , France

## Abstract

A highly efficient catalytic enantioselective [4+2] cycloaddition was developed between 2-benzothiazolimines and enecarbamates. A wide range of benzothiazolopyrimidines bearing three contiguous stereogenic centers was obtained in high to excellent yields and with excellent diastereo- and enantioselectivities (d.r. > 98 : 2 and up to >99% ee). Furthermore, this chiral phosphoric acid-catalyzed strategy was scalable and enabled access to a new class of optically pure Lewis base isothiourea derivatives.

## Abstract 2

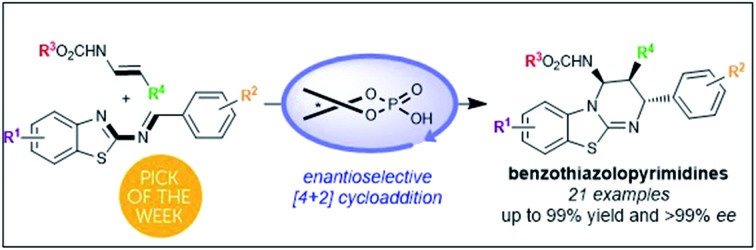
Chiral phosphoric acid catalyzed the formal [4+2]-cycloaddition of 2-benzothiazolimines with enecarbamates to provide benzothiazolopyrimidines with up to 99% yield and >99% ee.

## Introduction

**Scheme 1:**
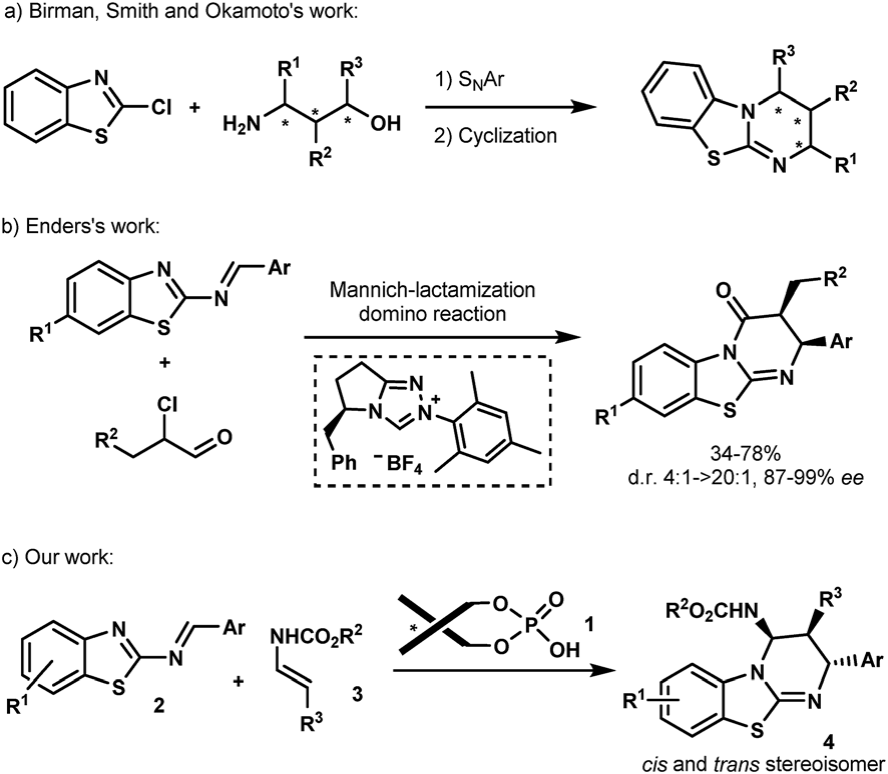
Previous studies and our strategy.

Isothioureas constitute an important class of organocatalysts due to their highly nucleophilic nature.^1^ Recent efforts have been directed to the development of chiral derivatives capable of promoting enantioselective transformations, which are difficult to control using traditional nucleophilic catalysts. Among the several promising examples, benzothiazolopyrimidine, better known as homobenzotetramisole (HBTM), has assumed a leading position as a chiral catalyst. Chiral HBTMs have been successfully used in a number of asymmetric transformations1*a* including the kinetic resolution of secondary alcohols and carboxylic acids,^2^ desymmetrization,^3^ acyl transfer,^4^ annulation processes,^5^ and lactonisation,^6^ among others.^7^ Nevertheless, despite the interest in optically active HBTMs,1*a* there have been only limited reports on their enantioselective synthesis.^8^ In 2008, Birman *et al.* disclosed the first asymmetric synthesis of HBTMs following a seven-step sequence involving a key S_N_Ar reaction between 2-chlorobenzo[*d*]thiazole and amino-alcohols prepared from the chiral pool of amino acids (Scheme 1a).2*f* Chiral amino-alcohols, produced by catalytic enantioselective procedures, were later engaged in similar routes as reported by the same group as well as by Smith *et al.* and Okamoto *et al.* (Scheme 1a).4a,b,^9^ More recently, Enders *et al.* established an efficient asymmetric synthesis of precursors of HBTMs by the *N*-heterocyclic carbene-catalyzed Mannich/lactamization domino reaction of 2-benzothiazolimines and α-chloroaldehydes (Scheme 1b).^10^ Despite these examples, no catalytic asymmetric approach has been reported so far to directly construct these heterocycles. An attractive direct access relying on the aza-Diels–Alder approach has been reported by Mellor *et al.* for the synthesis of homobenzotetramisole in an achiral stoichiometric context.^9^,^11^ The requirement of a full equivalent of the acid to perform the reaction is self-evident due to the presence of the basic isothiourea that might capture the acid during the formation of HBTMs.2f,^12^ Turning this process into a catalytic enantioselective approach would be highly appealing, not only to access new potent chiral Lewis bases but also for the generation of novel biologically active molecules.^13^

In the past few years, chiral phosphoric acids have been widely recognized as powerful catalysts for the synthesis of various heterocycles containing nitrogen atoms.^14^ In this context, we have recently documented phosphoric acid-catalyzed enantioselective aza-Diels–Alder reactions^14^,^15^ of various azadienes with enecarbamates as dienophiles.^16^ Based on these studies, we envisioned that this Brønsted acid **1** may be capable of promoting the [4+2] cycloaddition between 2-benzothiazolimines **2** and enecarbamates **3** (Scheme 1c) allowing the development of an enantioselective synthesis of benzothiazolopyrimidines **4**.

Because of the formation of the basic isothiourea during the process, catalyst poisoning might lead to difficulties in developing a catalytic version. Nevertheless, we reasoned that tuning the catalyst or using additives to induce a hydrogen shuttle could help in overcoming the problem.^17^

## Results and discussion

With this proposal in mind, we examined the aza-Diels–Alder reaction of 2-benzothiazolimine^18^**2a** with (*E*)-*N*-benzyl propenylcarbamate (**3a**) in the presence of chiral phosphoric acids **1**. Gratifyingly, in dichloromethane at room temperature, the reaction promoted by **1a** afforded benzothiazolopyrimidine **4a** as a single diastereomer, with an enantiomeric excess of 95% albeit with a low yield (Table 1, entry 1). Obviously, while yields remained low, we were delighted to observe that no full catalyst inhibition was occurring. Encouraged by this result, we screened various reaction conditions to search for the optimal reaction conditions. Performing the reaction at 50 °C in DCE slightly improved the kinetics (16 h instead of 24 h) even if overall yields were not superior and significant erosion of enantioselectivity was observed.^19^ Then, a series of phosphoric acids were screened in DCE at 50 °C (entries 2–4), and we found that the catalyst structure had a marked impact on the reaction efficiency. For instance, the use of chiral phosphoric acid **1c** having two bulky 3,3′-(2,4,6-triisopropylphenyl) groups improved the yield, presumably by facilitating the catalyst turnover. A further survey of reaction conditions revealed that the optimal conditions consisted in performing the reaction with 3 equivalents of **3a** in the presence of 10 mol% of **1c**, delivering **4a** in 90% yield with almost perfect diastereo- and enantioselectivity (entry 5).

**Table 1:** Optimization of the enantioselective [4+2] cycloaddition^*a*^

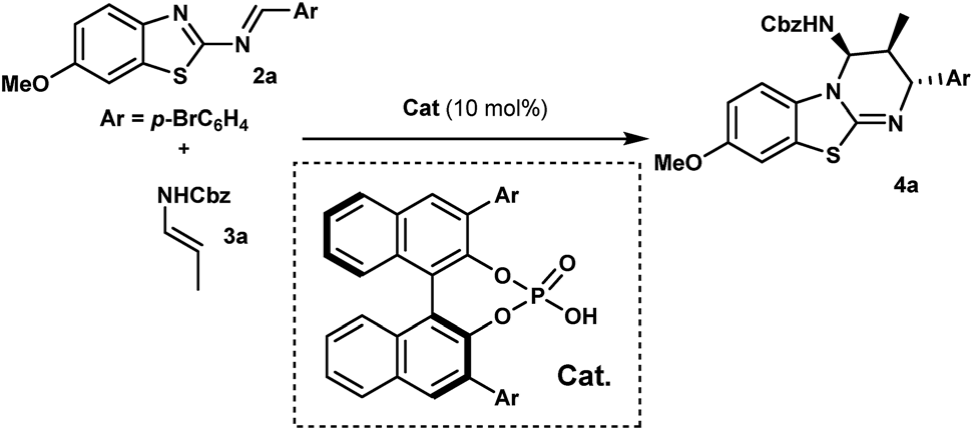					
Entry	Ar	*T* (°C)	Solvent	Yield^*b*^ (%)	ee^*c*^ (%)
1^*d*^	Ph (**1a**)	RT	DCM	40	95
2	**1a**	50	DCE	41	80
2	9-Phenantryl (**1b**)	50	DCE	29	85
4	2,4,6-TRIP (**1c**)	50	DCE	62	98
5^*e*^	**1c**	50	DCE	90 (68)^*f*^	99

^*a*^Reaction conditions: **2a** (0.10 mmol), **3a** (0.20 mmol) and **1** (0.01 mmol) in 1.0 mL of solvent for 16 h.

^*b*^Yields refer to the chromatographically pure diastereomer **4a** determined to be higher than 98 : 2 by ^1^H NMR.

^*c*^ee values were determined by HPLC with a chiral stationary phase.

^*d*^For 24 h.

^*e*^With 3 equiv. of **3a**.

^*f*^With 5 mol% of **1c**.

**Scheme 2:**
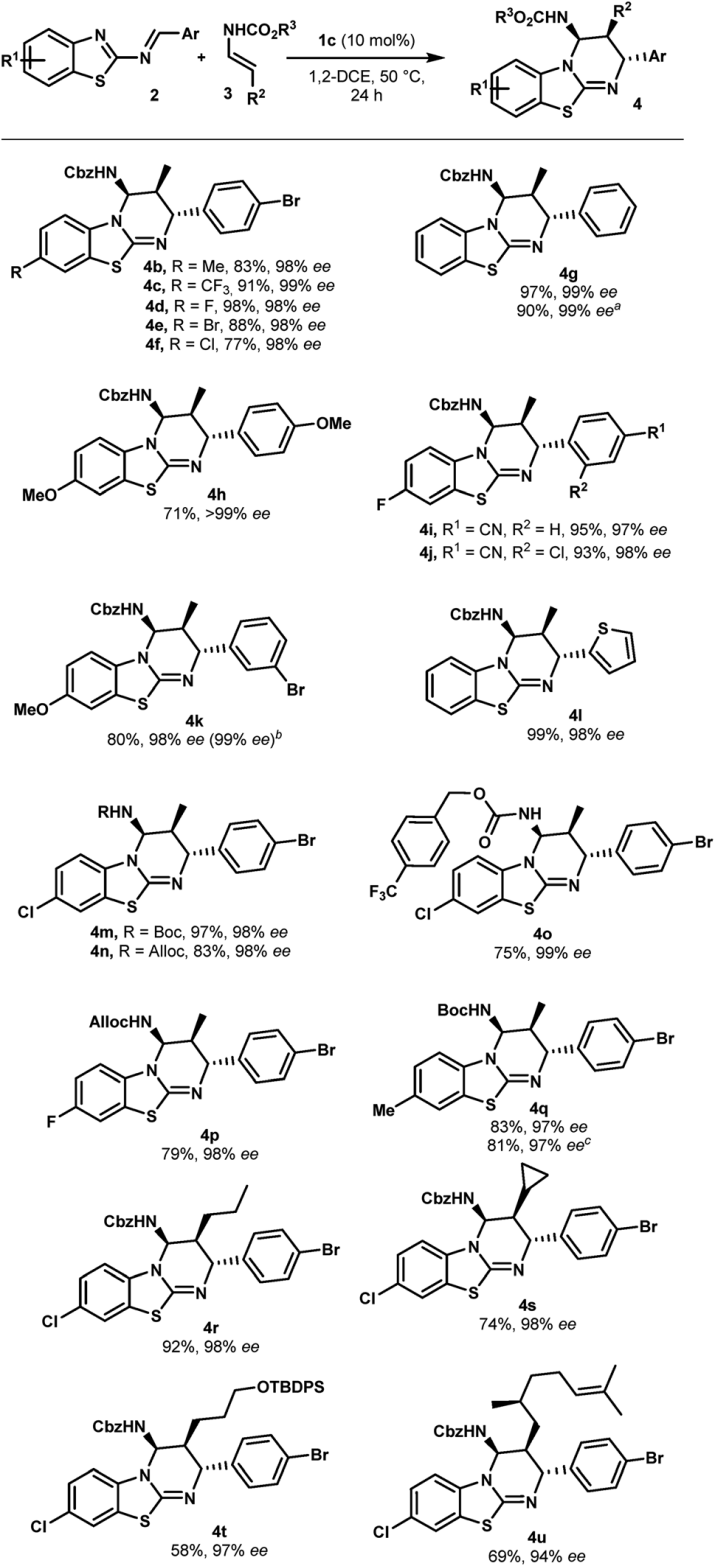
Scope of the enantioselective synthesis of benzothiazolopyrimidines **4**. ^a^ General reaction conditions: **2** (0.1 mmol), **3** (0.3 mmol), **1c** (10 mol%), 1,2-DCE (1 mL, 0.1 M), 50 °C, and 24 h. Yields refer to chromatographically purified compounds. The d.r. values were determined by ^1^H NMR analysis to be higher than 98 : 2 in all cases. The ee values were determined by HPLC on a chiral stationary phase: see the ESI† for details. ^a^ From 1.0 mmol of **2g**. ^b^ After recrystallization. ^c^ From 0.3 mmol of **2b**. ^d^ With 5 mol% of **1c**.

With optimized reaction conditions in hand, the scope of this chiral phosphoric acid-catalyzed enantioselective aza-Diels-Alder reaction was investigated and the results are summarized in Scheme 2. First, 2-benzothiazolimines bearing various substituents on the aromatic rings were evaluated. Pleasingly, the reaction was insensitive to the electronic nature of the substituents on the benzothiazole rings. For instance, the benzothiazole bearing either electron-donating or electron-withdrawing groups, such as methyl, halogen and trifluoromethyl groups, provided the corresponding benzothiazolopyrimidine **4b–f** in high yields and excellent enantioselectivities (up to 99% ee). An unsubstituted benzothiazole ring proceeded uneventfully to give cycloadduct **4g** with almost perfect enantioselectivity (up to 99% ee). It is worth noting that the reactions performed on a 1 mmol scale displayed the same results as the ones on a 0.10 mmol scale, further indicating the practicality of this protocol. On the other hand, the electronic properties of aromatic rings that came from aldehydes had no significant impact on the enantiomeric excess, though the reaction with the electron-rich imine gave a slightly lower yield (**4h***vs.***4a**). When the reaction was conducted with imine, which had bromine at the *meta*-position, the desired cycloadduct **4k** was obtained in 80% yield and 98% ee. Notably, **4k** can be enriched to >99% ee by single recrystallization, which is very interesting in view of their use as organocatalysts. Moreover, a relative and absolute configuration was unambiguously determined by single crystal X-ray analysis (see the ESI†). Finally, the reaction with imine **2l** derived from 2-thiophenecarboxaldehyde proceeded smoothly to give the cycloadduct **4l** in almost quantitative yields with 98% ee. This novel catalytic enantioselective cycloaddition was also applied to representative enecarbamates (Scheme 2). Notably, a variety of protecting groups bonded to the nitrogen atom of **3** (including Boc, Alloc and 4-(trifluoromethyl)benzylcarbamate) were well tolerated and the corresponding benzothiazolopyrimidines (**4m–q**) were, in all cases, isolated in excellent yields and excellent stereoselectivities. It is noteworthy that the catalyst loading was successfully reduced to 5.0 mol% (**4q**, entry 10). A comparable enantioselectivity (97%) was still achieved with a slightly lower chemical yield (70%).

In addition, β-substituted enecarbamates bearing different alkyl groups reacted efficiently to deliver expected trisubstituted-cycloadducts **4r–u** in good to excellent yields with high selectivities (94 to 98% ee). The reaction with **3c** or **3d** containing a cyclopropyl or silyl ether group formed products **4s** or **4t** with a slight decrease in the yield without affecting asymmetric induction.

**Fig. 1:**
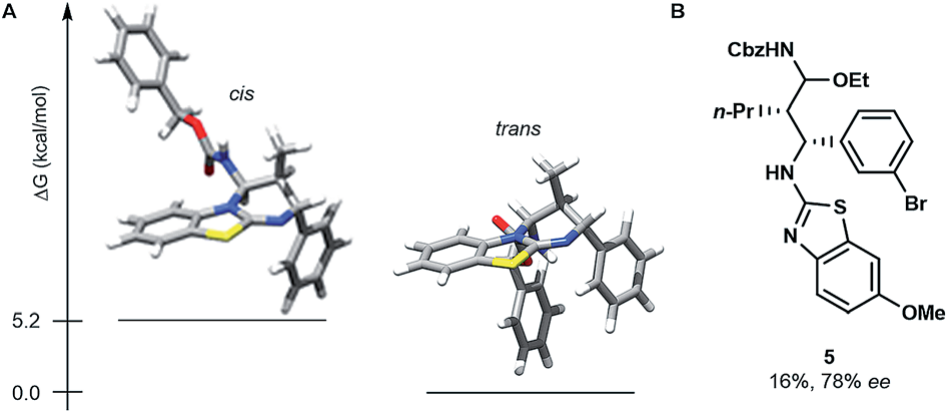
(A) Most stable *cis* and *trans* conformers of **4g** found at the B3LYP/6-31G(d) level. (B) Structure of intermediate **5** obtained in the presence of 3.0 equiv. of ethanol on the way to **4k**.

The stereoselective issue of the reaction ((*E*)-enecarbamates to the *cis* product) raises questions about the mechanism. In order to get insight, we computationally evaluated the free energy of both diastereoisomers of the product **4g** (Fig. 1A). The *trans* form was found favoured by more than 5.2 kcal mol^–1^ over its *cis* sibling (see the ESI†). This result together with the fact that the *cis* isomer is actually observed suggests that the cycloaddition occurs under kinetic control. A stepwise process was established by performing a control experiment in the presence of 3 equivalents of ethanol, which evidenced the formation of an *N*-Cbz-iminium intermediate (**5**, Fig. 1B).

**Scheme 3:**
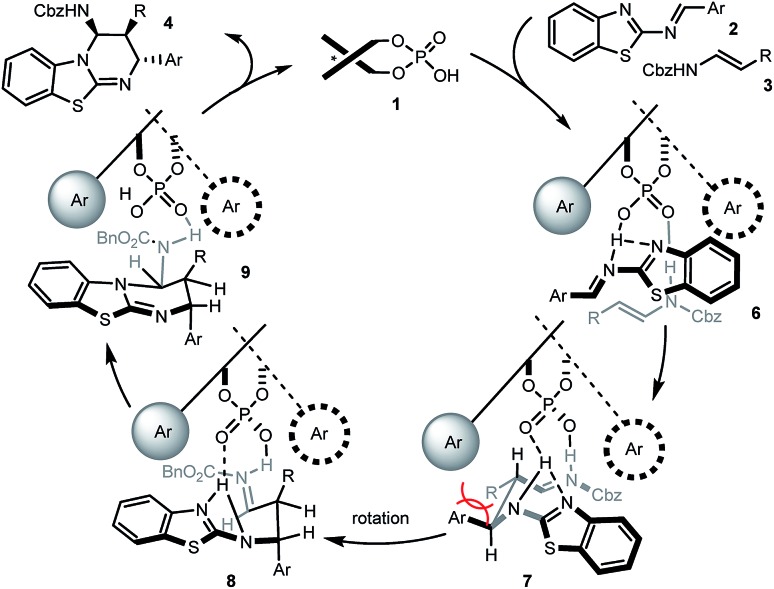
Plausible mechanism and transition states.

Based on this, as well as our previous work and other published reports,^14^,^16^,^20^ a possible catalytic mechanism was proposed in Scheme 3. We hypothesized that **1** would form a hydrogen network with the nitrogen Lewis basic sites of 2-benzothiazolimine **2** and the acidic site of enecarbamate **3**, leading to complex **6**. Mediated by the phosphoric acid double activation, subsequent bond formation would deliver complex **7**. The aromatic and R residue would be positioned in a *trans* situation as found in the product, but the pseudo equatorial arrangement would create substantial strain. Maintained in the chiral cavity by the double hydrogen anchors, rotation of the backbone would furnish a sterically less-congested conformer **8**. Finally the intramolecular nucleophilic addition of benzothiazole to iminium will give the observed *cis*–*trans* substituted benzothiazolopyrimidine **4**.^21^ Heating, rapid formation of hydrogen-bonded complex **6** or use of excess **3** may all facilitate the decomplexation of **9** and ensure the catalyst turnover.

**Scheme 4:**
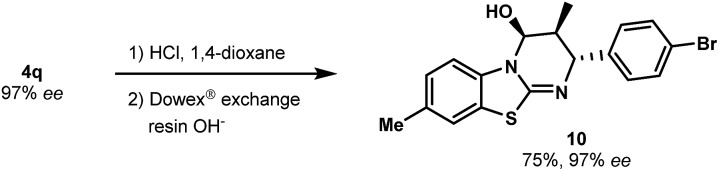
Derivatization of the benzothiazolopyrimidine product **4q**.

As a further illustration of the utility of this novel catalytic cycloaddition, we developed conditions to convert the carbamate group into other functional groups. The N-Boc group of **4q** was removed by mild acidic hydrolysis to produce free alcohol **10** (a key intermediate to install additional molecular diversity) with no erosion of enantiopurity (Scheme 4). The absolute configuration was confirmed without ambiguity by X-ray diffraction analysis (see the ESI†).

## Conclusions

We have developed an efficient enantioselective [4+2] cycloaddition organocatalyzed by chiral phosphoric-acid for the synthesis of benzothiazolopyrimidines bearing three contiguous stereogenic centers, which were unreported before. Involving 2-benzothioazolimines and enecarbamates as substrates, this scalable strategy furnished a wide range of compounds with very high yields and enantioselectivity in a diastereospecific manner. The obtained cycloadducts were successfully converted into corresponding free alcohol heterocycles without any loss of enantioselectivity. Further investigations are in progress concerning the catalytic activity of these new compounds as chiral nucleophilic organocatalysts.

## Conflicts of interest

There are no conflicts to declare.

## Supplementary Material

Supplementary information

Crystal structure data
