# Supplementary material for: Chiral phosphoric acid-catalyzed enantioselective construction of structurally diverse benzothiazolopyrimidines
Source: Chem Sci. 2019 Feb 7;10(13):3765–9. doi: 10.1039/c8sc05581e (PMC6461124; doi:10.1039/c8sc05581e)
Supplement: Supplementary file 1 [file SC-010-C8SC05581E-s001.pdf]

Electronic Supplementary Material (ESI) for Chemical Science.
This journal is © The Royal Society of Chemistry 2019

Supporting Information

Chiral Phosphoric Acid-Catalyzed Enantioselective Construction of Structurally Diverse Benzothiazolopyrimidines

*Lucie Jarrige, Danijel Glavač, Guillaume Levitre, Pascal Retailleau, Guillaume Bernadat, Luc Neuville and Géraldine Masson**

Table of Contents

I. General Notes S1

II. General Procedures S2

III. Catalyst and Reaction Condition Optimization S6

IV. Synthesis and Characterization of 2-benzothiazolimines **2** S7

V. Synthesis and Characterization of cycloadducts **4** S27

VI. Post-transformation : Synthesis of compound **5** and **10** S103

VII. Computational models of *cis* and *trans* diastereoisomers of cycloadduct **4g** S109

VIII. X-Ray data of compounds **4k** and **10** S113

I. General Notes

Unless otherwise stated, all reagents were obtained from commercial suppliers and used without further purification.
Analytical thin layer chromatography (TLC) plates were purchased from Merck KGaA (silica gel 60 F_{254}). Visualization was accomplished by irradiation with a UV light at 254 nm. Flash column chromatography was carried out using kieselgel 35-70 µm particle sized silica gel (200-400 mesh). Column chromatography was performed on Merck silica gel (60, particle size 0.040-0.063 mm).
Proton (^{1}H) and carbon (^{13}C) NMR spectra were recorded on Bruker spectrometers : Avance 300 MHz (QNP - ^{13}C, ^{31}P, ^{19}F - probe or Dual ^{13}C probe) and Avance 500 MHz (BB0 - ATM probe or BBI - ATM probe). Proton chemical shifts (δ) are reported in parts per million (ppm) relative to tetramethylsilane (TMS) with the solvent resonance employed as the internal standard ($CDCl_3$ δ 7.26 ppm; $(CD_3)_2CO$ δ 2.05 ppm; CD_3CN δ 1.94 ppm). Data are reported as follows: chemical shift, multiplicity (s = singlet, d = doublet, t = triplet, q = quartet, p = pentet, s = sextet, h = heptet, m = multiplet, br = broad), coupling constants (*J*) and integration. Coupling constants (*J*) are reported in Hertz (Hz). Carbon chemical shifts are reported in ppm from tetramethylsilane (TMS) with the solvent resonance as the internal standard ($CDCl_3$ δ 77.16 ppm; $(CD_3)_2CO$ δ 29.84 and 206.26 ppm; CD_3CN δ 1.32 and 118.26 ppm). The multiplicity of carbons was given using 2D spectra (HSQC and HMBC). Some quaternary carbons were determined using HMBC couplings.
UPLC-MS analyses were run using a Acquity Waters UPLC equipped with a Waters LCT Premier XE (ESI ionization) and a Waters Acquity PDA detector, using a column BEH C18 1.7 µm, 2.1 mm × 50 mm. Gradients were run using water and acetonitrile (1:1) with 0.1% of acetic acid. Temperature: 40 °C. UV detection from 210 to 410 nm. ESI+ detection in the 80−1500 *m/z* range.
Infrared spectra were recorded on Perkin Elmer Spectrum 100 FT-IR spectrometer and absorption frequencies were reported in reciprocal centimeters (cm^{-1}).
Melting points, measured in capillary tubes on a Büchi B-540 apparatus, are uncorrected.
Optical rotations were performed on a Jasco P-1010 polarimeter at 589 nm and 22 °C using a 700 µL cell with a path length of 1 dm.
Chiral HPLC analysis was performed on Hitachi LaChrom-Elite apparatus equipped with diode array UV detector (UV detection monitored at 254 nm). Enantiomeric ratios were determined by HPLC analysis employing a chiral stationary phase column specified in the individual experiment, by comparing the samples with the appropriate racemic mixtures.
Catalyst **1c** was prepared according to literature procedures.[1]
Enecarbamates **3** were synthesized according to literature procedures and data were already described.[2]

[1] Klussmann, M.; Ratjen, L.; Hoffmann, S.; Wakchaure, V.; Goddard, R.; List, B. *Synlett* **2010**, 2189.
[2] a) Carboni, A.; Dagousset, G.; Magnier, E.; Masson, G. *Org. Lett.* **2014**, *16*, 1240. b) Halli, J.; Kramer, P.; Bechthold, M.; Manolikakes, G. *Adv. Synth. Catal.* **2015**, *357*, 3321.

II. General Procedures

General procedure A: *Synthesis of enecarbamates 3 from acyl chlorides*

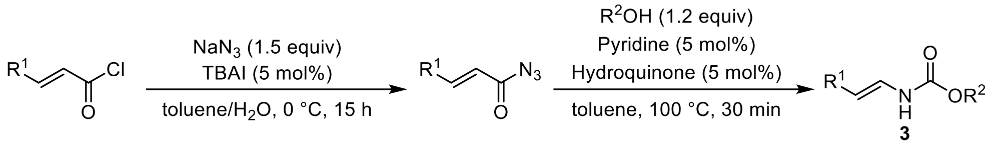

A solution of the corresponding acyl chloride (1.0 equiv) in toluene (4 M) was added dropwise to a solution of NaN_3 (1.5 equiv) and tetrabutylammonium iodide (TBAI, 0.05 equiv) in H_2O (3 M) at 0 °C. After stirring the reaction for 15 h at 0 °C, the organic layer was separated and washed with 10% aqueous solution of Na_2CO_3 and water. The organic solution was dried over $MgSO_4$ before use in the following step. Then, the toluene solution of acyl azide was added dropwise to a stirred mixture of hydroquinone (0.05 equiv), pyridine (0.05 equiv), and the corresponding alcohol (1.2 equiv) at 95 °C. The mixture was then stirred at 100 °C until the gas evolution stops. The resulting solution was evaporated under reduced pressure and the crude product was purified by flash chromatography on silica gel employing mixtures of heptane and ethyl acetate as eluents to afford the desired product **3**.

General procedure B: *Synthesis of enecarbamates 3 from carboxylic acids*

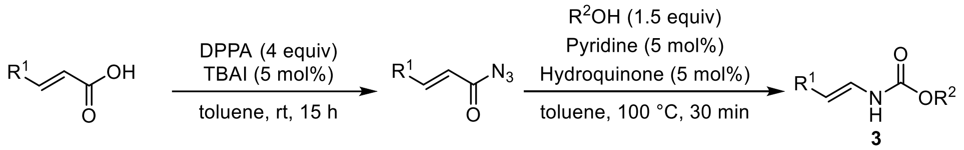

To a solution of the corresponding carboxylic acid (1 equiv) in toluene (0.15 M), was added Et_3N (4.0 equiv) and diphenylphosphoryl azide (DPPA, 4.0 equiv). The mixture was stirred at room temperature overnight. Then the reaction was diluted in CH_2Cl_2 and washed with brine. The organic layer was dried over $MgSO_4$ and concentrated *in vacuo*. The crude acyl azide was purified by column chromatography on silica gel (Heptane/EtOAc). Then, the solution of the acyl azide in toluene (1 M) was added dropwise to a stirred mixture of hydroquinone (0.05 equiv), pyridine (0.05 equiv), and corresponding alcohol (1.2 equiv) at 95 °C. The resulting solution was evaporated under reduced pressure and the crude product was purified by flash chromatography on silica gel employing mixtures of heptane and ethyl acetate as eluents to afford the desired product **3**.

General procedure C: *Synthesis of 2-benzothiazolimines 2*

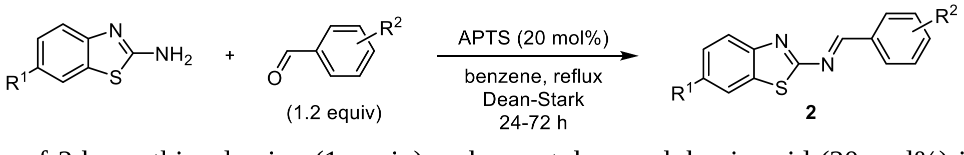

To solution of 2-benzothiazolamine (1 equiv) and *para*-toluenesulphonic acid (20 mol%) in benzene (0.1 M), was added the appropriate arylaldehyde (1.2 equiv). The mixture was stirred under reflux for a minimum of 24 h while water was removed in a Dean–Stark trap. After completion (monitored by TLC), the solvent was removed under reduced pressure and the crude product was directly purified by flash chromatography on silica gel employing mixtures of heptane and ethyl acetate as eluents to afford the desired product **2**. Imines **2a**, **2g**, **2h** and **2l** were already described in the literature.[3]

[3] a) Vicini, P.; Geronikaki, A.; Incerti, M.; Busonera, B.; Poni, G.; Cabras, C. A.; La Colla, P. *Bioorg. Med. Chem.* **2003**, *11*, 4785. b) Thakkar, S. S.; Thakor, P.; Ray, A.; Doshi, H.; Thakkar, V. R. *Bioorg. Med. Chem.* **2017**, *25*, 5396. c) Gan, C.; Zhou, L.; Zhao, Z.; Wang, H. *Med. Chem. Res.* **2013**, *22*, 4069.

General procedure D: *Enantioselective synthesis of homobenzotetramizoles **4***

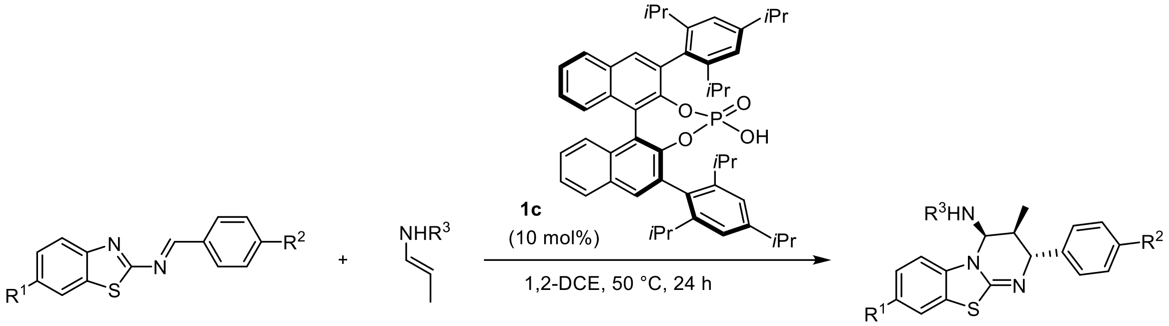

2 **3** (3 equiv) **4**

To a solution of imine **2** (1 equiv) and catalyst **1c** (10 mol%) in dry 1.2-DCE (0.1 M), was added the enecarbamate **3** (3 equiv) in one portion. The reaction mixture was stirred for 24 h at 50 °C. After completion (monitored by TLC), the solvent was removed under reduced pressure and the crude product was directly purified by flash chromatography on silica gel employing mixtures of heptane and ethyl acetate as eluents to afford the desired product **4**.

General procedure E: *Racemic synthesis of homobenzotetramizoles **4***

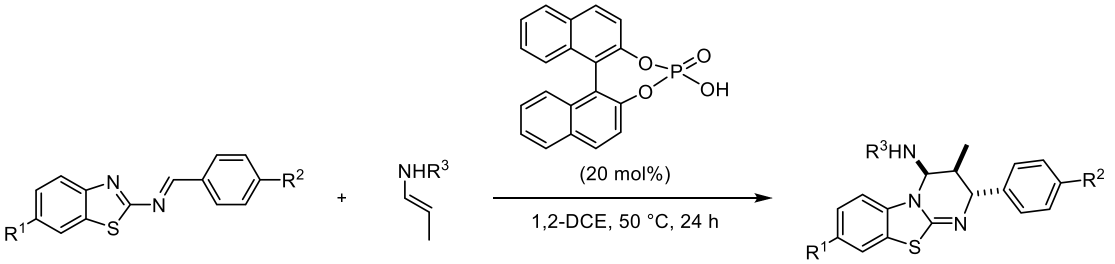

2 **3** (3 equiv) **4**

To a solution of imine **2** (1 equiv) and racemic phosphoric acid catalyst (20 mol%) in dry 1.2-DCE (0.1 M), was added the enecarbamate **3** (3 equiv) in one portion. The reaction mixture was stirred for 24 h at 50 °C. After completion (monitored by TLC), the solvent was removed under reduced pressure and the crude product was directly purified by flash chromatography on silica gel employing mixtures of heptane and ethyl acetate as eluents to afford the racemic compound **4**.

General procedure F: *Enantioselective synthesis of homobenzotetramizoles **4***

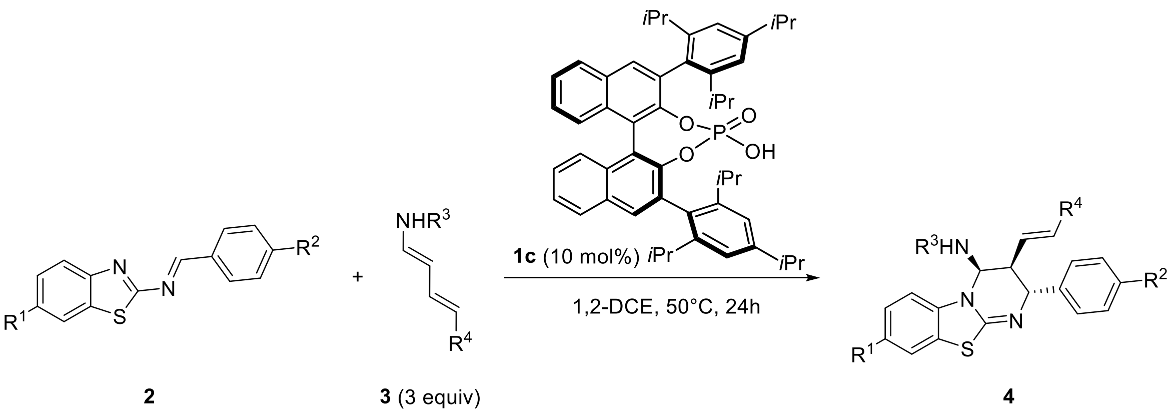

2 **3** (3 equiv) **4**

To a solution of imine **2** (1 equiv) and catalyst **1c** (10 mol%) in dry 1.2-DCE (0.1 M), was added the enecarbamate **3** (3 equiv) in one portion. The reaction mixture was stirred for 24 h at 50 °C. After completion (monitored by TLC), the solvent was removed under reduced pressure and the crude product

was directly purified by flash chromatography on silica gel employing mixtures of heptane and ethyl acetate as eluents to afford the desired product **4**.

General procedure G: *Racemic synthesis of homobenzotetramizoles **4***

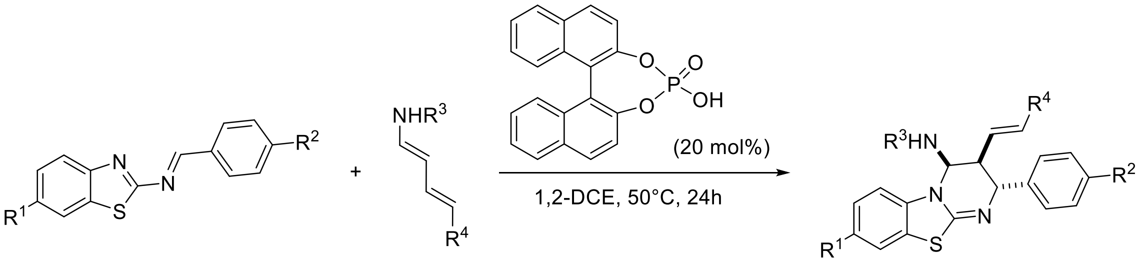

2 **3** (3 equiv) **4**

To a solution of imine **2** (1 equiv) and racemic phosphoric acid catalyst (20 mol%) in dry 1.2-DCE (0.1 M), was added the enecarbamate **3** (3 equiv) in one portion. The reaction mixture was stirred for 24 h at 50 °C. After completion (monitored by TLC), the solvent was removed under reduced pressure and the crude product was directly purified by flash chromatography on silica gel employing mixtures of heptane and ethyl acetate as eluents to afford the racemic compound **4**.

General procedure H: *Enantioselective synthesis of homobenzotetramizoles **4w** and **5***

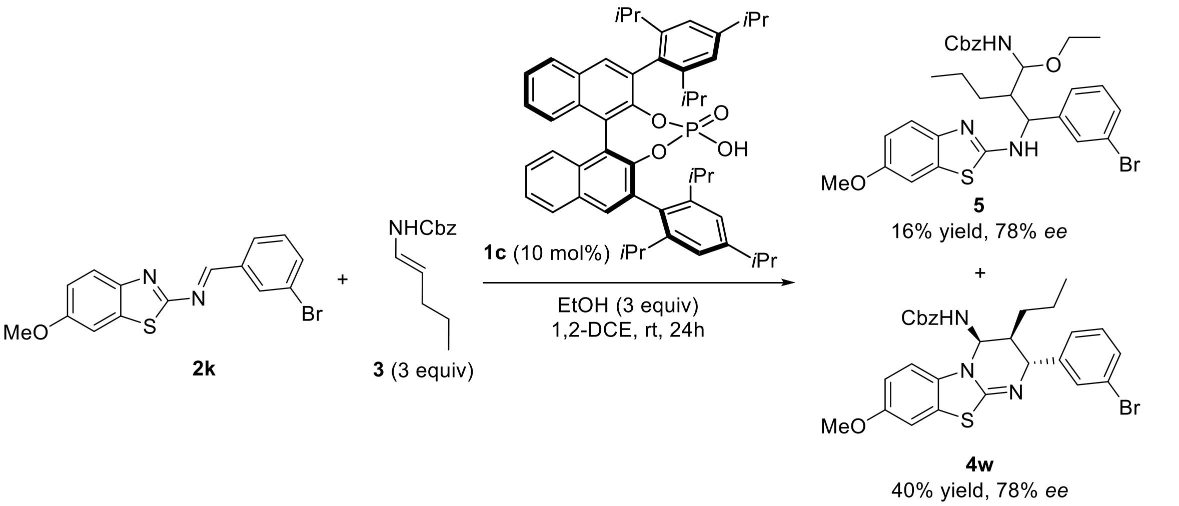

To a solution of imine **2k** (1 equiv) and catalyst **1c** (10 mol%) in dry 1.2-DCE (0.1 M), was added the enecarbamate **3e** (3 equiv) in one portion. The reaction mixture was stirred for 24 h at 50 °C. After completion (monitored by TLC), the solvent was removed under reduced pressure and the crude product was directly purified by flash chromatography on silica gel employing mixtures of heptane and ethyl acetate as eluents to afford the desired products **4w** and **5**.

General procedure I: *Racemic synthesis of homobenzotetramizoles* ***4w*** *and* ***5***

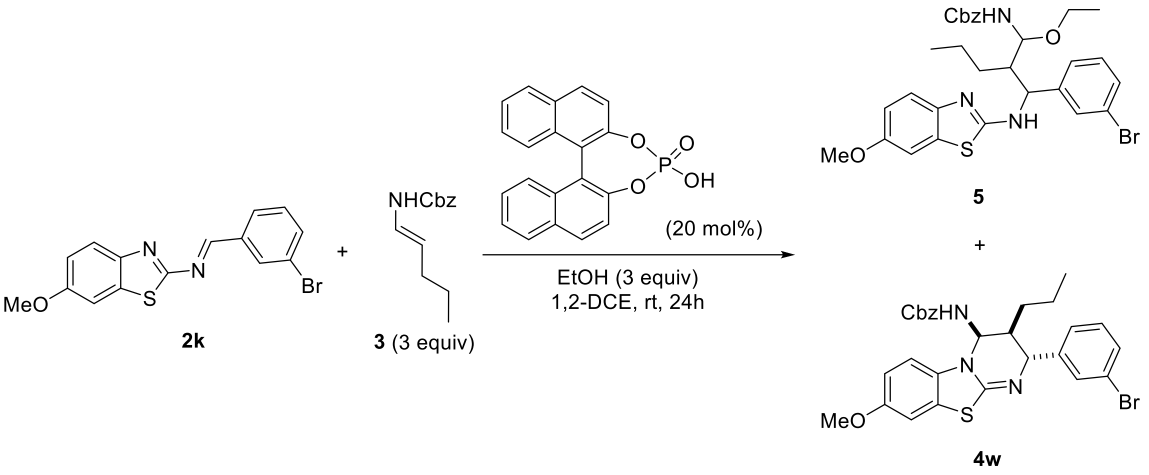

To a solution of imine **2k** (1 equiv) and racemic phosphoric acid catalyst (20 mol%) in dry 1.2-DCE (0.1 M), was added the enecarbamate **3e** (3 equiv) in one portion. The reaction mixture was stirred for 24 h at 50 °C. After completion (monitored by TLC), the solvent was removed under reduced pressure and the crude product was directly purified by flash chromatography on silica gel employing mixtures of heptane and ethyl acetate as eluents to afford the racemic compounds **4w** and **5**.

III. Catalyst and Reaction Condition Optimization

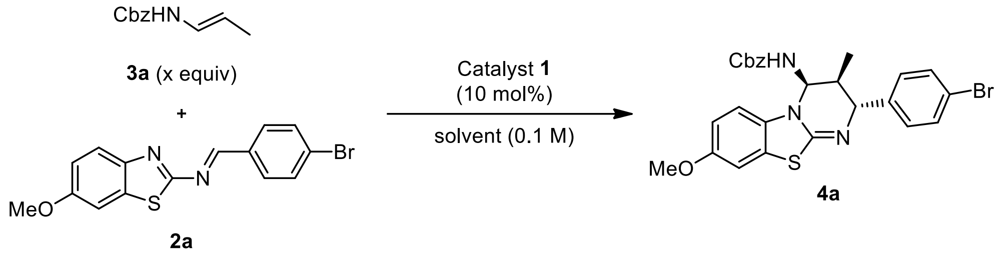

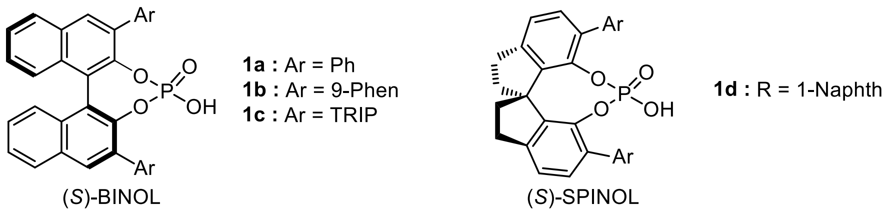

Entry	Catalyst **1**	*x*	Solvent	*T* [°C]	*t* [h]	Yield [%][b]	*ee* [%][c]
1	**1a**	1	CH_2Cl_2	RT	24	40	95
2	**1a**	2	CH_2Cl_2	0 °C then RT	72	35	90
3	**1a**	2	CH_2Cl_2	10	48	33	88
4	**1a**	2	toluene	RT	24	24	80
5	**1a**	2	1,2-DCE	50	24	41	80
6	**1b**	2	1,2-DCE	50	24	29	85
7	**1c**	2	1,2-DCE	50	24	62	98
8	**1c**	3	1,2-DCE	50	24	90	99
9	**1d**	3	1,2-DCE	50	24	55	98

Table S1. Catalyst and Reaction Condition Optimization. [a] Reaction conditions: **2a** (0.1 mmol), **3a** (*x* x0.1 mmol), **1** (10 mol%), solvent (1 mL, 0.1 M). [b] Yields refer to chromatographically pure diastereoisomer **4a** determined to be higher than 98:2 by ^{1}H NMR. [c] *ee* values were determined by HPLC with a chiral stationary phase. TRIP = 2,4,6-triisopropylphenyl.

IV. Synthesis and Characterization of 2-benzothiazolimines 2

(*E*)-1-(4-bromophenyl)-*N*-(6-methylbenzo[d]thiazol-2-yl)methanimine

2b

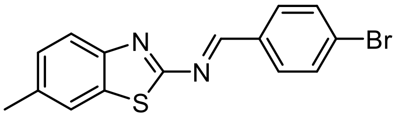

Compound **2b** was prepared according to the general procedure **C** from 4-bromobenzaldehyde (0.50 g, 2.70 mmol) and 2-amino-6-methylbenzothiazole (0.54 g, 3.26 mmol) as starting materials. Purification on a column of silica gel with a gradient of ethyl acetate in heptane (7:3) as eluent gave the desired product.

Chemical formula	$C_{15}H_{11}BrN_2S$
M (g.mol^{-1})	331.23
Yield	0.76 g, 85%
Aspect	Yellow solid
MP	196-199 °C
Rf	0.5 (Hept/AcOEt : 9/1)
^{1}H NMR (300 MHz, CDCl$_3$)	**δ :** 9.00 (s, 1H), 7.88 (d, *J* = 8.6 Hz, 2H), 7.85 (d, *J* = 8.3 Hz, 1H), 7.65 (d, *J* = 8.7 Hz, 2H), 7.63 (d, *J* = 1.3 Hz, 1H), 7.29 (dd, *J* = 8.4, 1.3 Hz, 1H), 2.49 (s, 3H)
^{13}C NMR (75 MHz, CDCl$_3$)	**δ :** 170.5 (C), 164.3 (CH), 149.8 (C), 135.6 (C), 135.0 (C), 133.8 (C), 132.5 (2 CH), 131.4 (2 CH), 128.3 (CH), 128.2 (C), 122.9 (CH), 121.6 (CH), 21.8 (CH_3)
IR (Neat, cm^{-1})	3095, 3052, 2912, 2859, 2729, 1900, 1761, 1681, 1619, 1601, 1585, 1560, 1498, 1481, 1435, 1401, 1367, 1311, 1299, 1285, 1248, 1225, 1212, 1164, 1147, 1100, 1065, 1057, 1004
HRMS (ESI+, m/z)	$[M+H]^+$ calcd. for $C_{15}H_{12}BrN_2S$ 330.9905, found 330.9915

(*E*)-1-(4-bromophenyl)-*N*-(6-methylbenzo[d]thiazol-2-yl)methanimine

2b

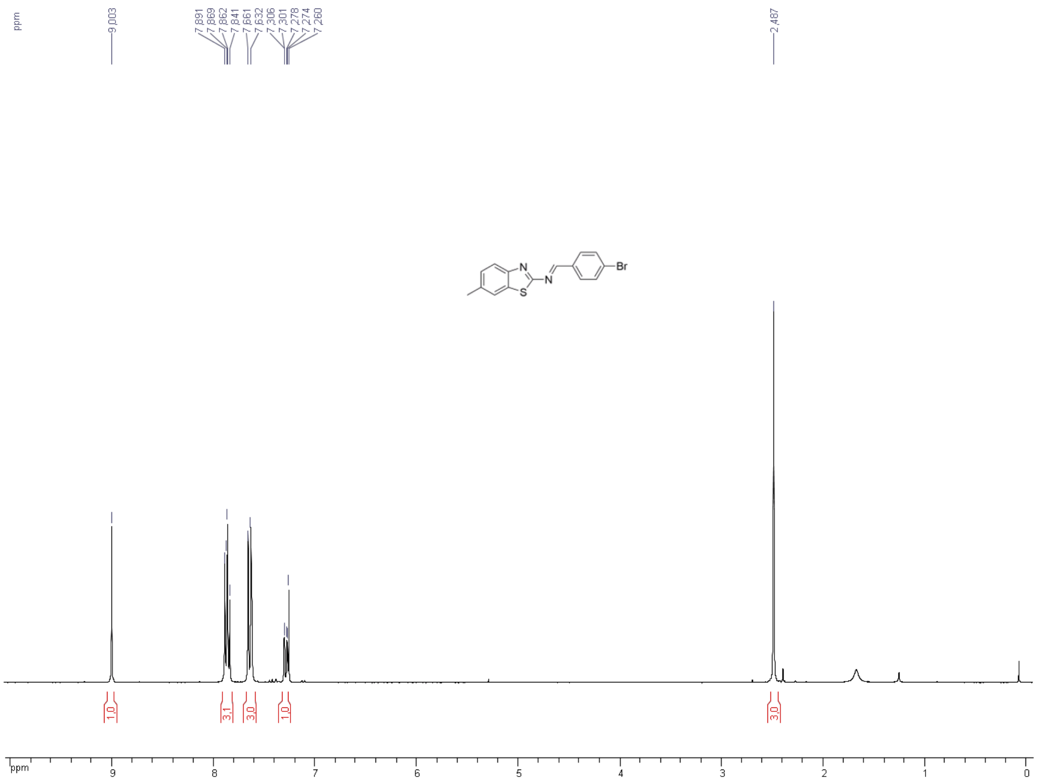

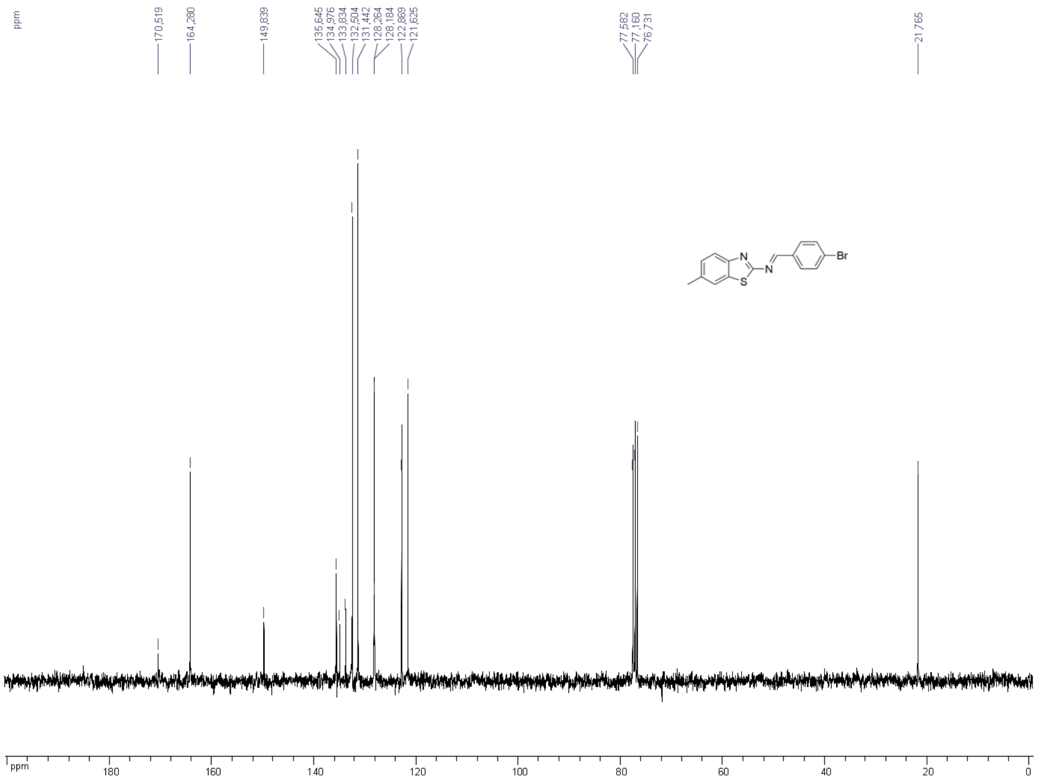

(*E*)-1-(4-bromophenyl)-*N*-(6-(trifluoromethyl)benzo[*d*]thiazol-2-yl)methanimine

2c

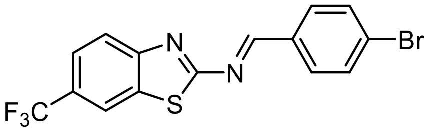

Compound **2c** was prepared according to the general procedure **C** from 4-bromobenzaldehyde (0.50 g, 2.70 mmol) and 2-amino-6-(trifluoromethyl)benzothiazole (0.71 g, 3.24 mmol) as starting materials. Purification on a column of silica gel with a gradient of ethyl acetate in heptane (from 99:1 to 9:1) as eluent gave the desired product.

Chemical formula	$C_{15}H_8BrF_3N_2S$
M (g.mol^{-1})	385.20
Yield	0.41 g, 39%
Aspect	Yellow solid
Rf	0.6 (Hept/AcOEt : 9/1)
^{1}H NMR (300 MHz, $CDCl_3$)	**δ :** 9.08 (s, 1H), 8.14 (s, 1H), 8.05 (d, *J* = 8.8 Hz, 1H), 7.90 (d, *J* = 7.7 Hz, 2H), 7.72 (dd, *J* = 8.7, 1.7 Hz, 1H), 7.68 (d, *J* = 8.5 Hz, 2H)
^{13}C NMR (75 MHz, $CDCl_3$)	**δ :** 174.2 (C), 165.9 (CH), 153.9 (C), 135.1 (C), 133.5 (C), 132.7 (2 CH), 131.8 (2 CH), 129.0 (C), 127.2 (d, *J* = 34.0 Hz, C), 124.1 (q, *J* = 269.6 Hz, CF_3), 123.7 (q, *J* = 3.2 Hz, CH), 123.5 (CH), 119.5 (q, *J* = 4.2 Hz, CH)
^{19}F NMR (282 MHz, $CDCl_3$)	**δ :** -61.48
IR (Neat, cm^{-1})	1596, 1585, 1558, 1500, 1485, 1459, 1449, 1411, 1404, 1366, 1322, 1298, 1279, 1249, 1230, 1179, 1152, 1136, 1097, 1077, 1067, 1051, 1007
HRMS (ESI+, m/z)	$[M+H]^+$ calcd. for $C_{15}H_9BrF_3N_2S$ 384.9622, found 384.9627

(*E*)-1-(4-bromophenyl)-*N*-(6-(trifluoromethyl)benzo[*d*]thiazol-2-yl)methanimine 2c

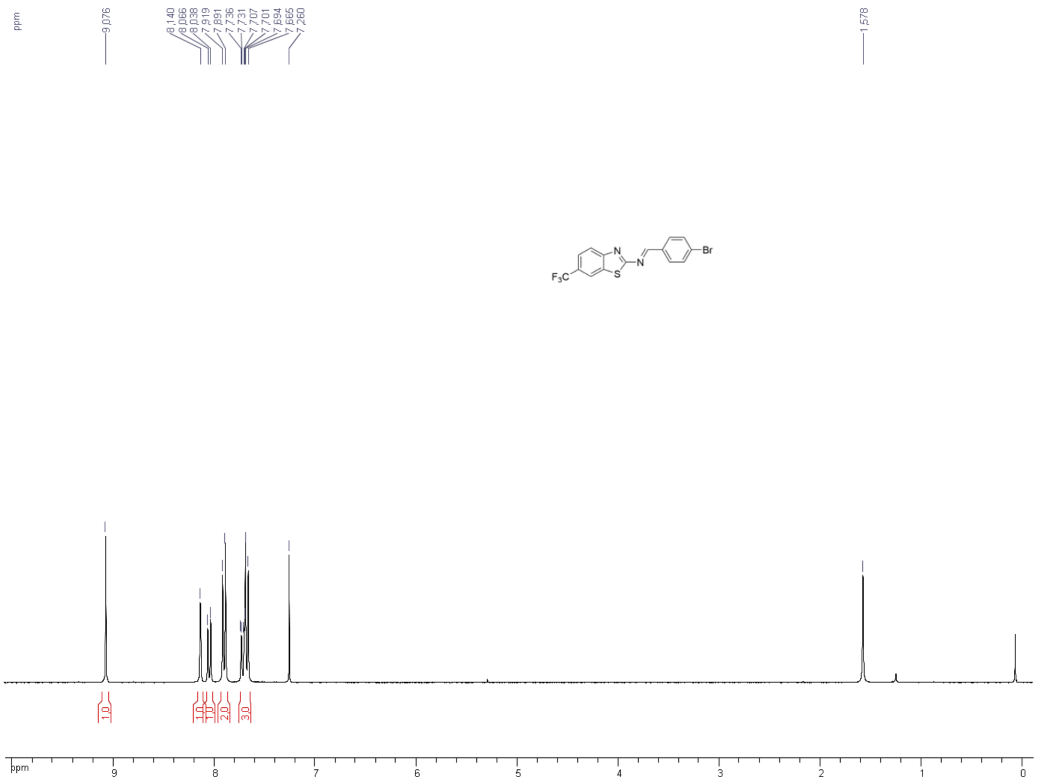

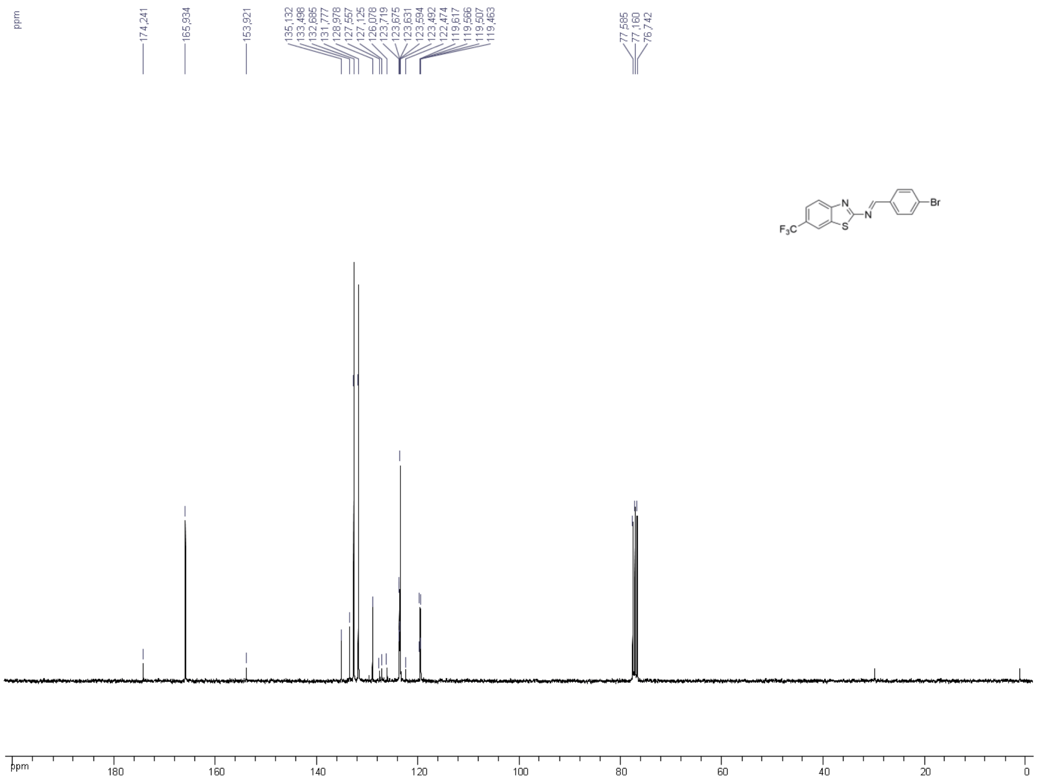

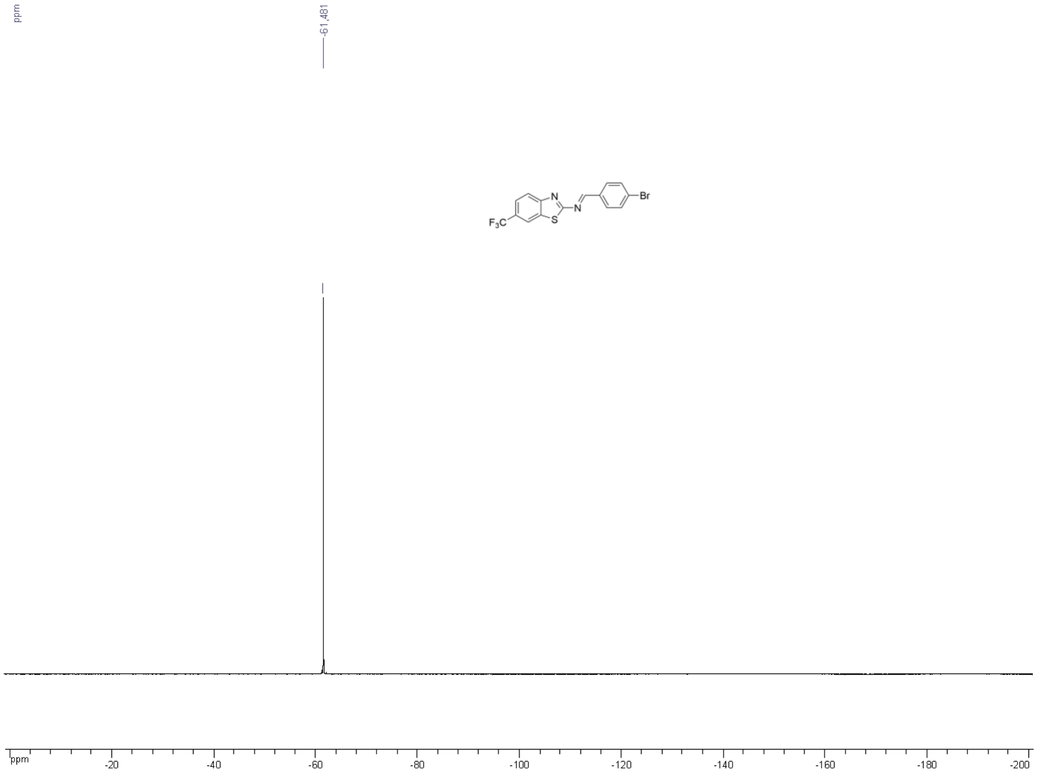
ppm
-61.481
F3C
N
S
N
Br
ppm
-20
-40
-60
-80
-100
-120
-140
-160
-180
-200

(*E*)-1-(4-bromophenyl)-*N*-(6-fluorobenzo[*d*]thiazol-2-yl)methanimine

2d

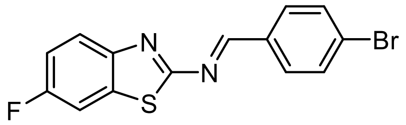

Compound **2d** was prepared according to the general procedure **C** from 4-bromobenzaldehyde (0.66 g, 3.57 mmol) and 2-amino-6-fluorobenzothiazole (0.50 g, 2.97 mmol) as starting materials. Purification on a column of silica gel with a gradient of ethyl acetate in heptane (from 95:5 to 1:1) as eluent gave the desired product.

Chemical formula	$C_{14}H_8BrFN_2S$
M (g.mol^{-1})	335.19
Yield	0.78 g, 78%
Aspect	Yellow solid
MP	196-200 °C
Rf	0.6 (Hept/AcOEt : 9/1)
^{1}H NMR (300 MHz, CDCl$_3$)	**δ :** 9.01 (s, 1H), 7.92 (dd, *J* = 8.8, 4.8 Hz, 1H), 7.89 (d, *J* = 8.1 Hz, 2H), 7.66 (d, *J* = 8.3 Hz, 2H), 7.53 (dd, *J* = 8.2, 2.6 Hz, 1H), 7.22 (dd, *J* = 8.9, 2.4 Hz, 1H)
^{13}C NMR (75 MHz, CDCl$_3$)	**δ :** 171.1 (C), 164.8 (CH), 160.5 (d, *J* = 246.9 Hz, CF), 148.4 (C), 133.7 (C), 132.6 (2 CH), 131.6 (2 CH), 131.1 (C), 128.5 (C), 124.3 (d, *J* = 10.4 Hz, CH), 115.4 (d, *J* = 24.7 Hz, CH), 108.2 (d, *J* = 24.7 Hz, CH)
^{19}F NMR (282 MHz, CDCl$_3$)	**δ :** -115.13
IR (Neat, cm^{-1})	3066, 2924, 2863, 1879, 1635, 1613, 1585, 1560, 1513, 1496, 1481, 1451, 1414, 1400, 1361, 1347, 1318, 1302, 1285, 1271, 1250, 1200, 1173, 1163, 1147, 1112, 1101, 1065, 1048, 1006
HRMS (ESI+, m/z)	$[M+H]^+$ calcd. for $C_{14}H_9BrFN_2S$ 334.9654, found 334.9659

(*E*)-1-(4-bromophenyl)-*N*-(6-fluorobenzo[*d*]thiazol-2-yl)methanimine 2d

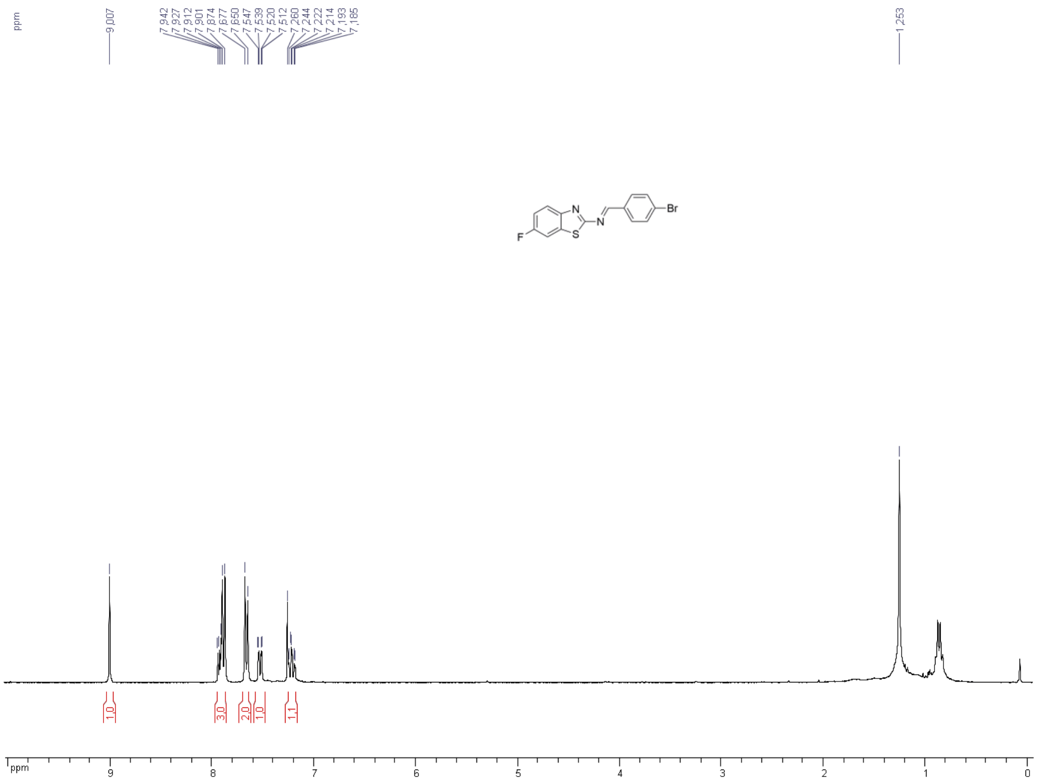

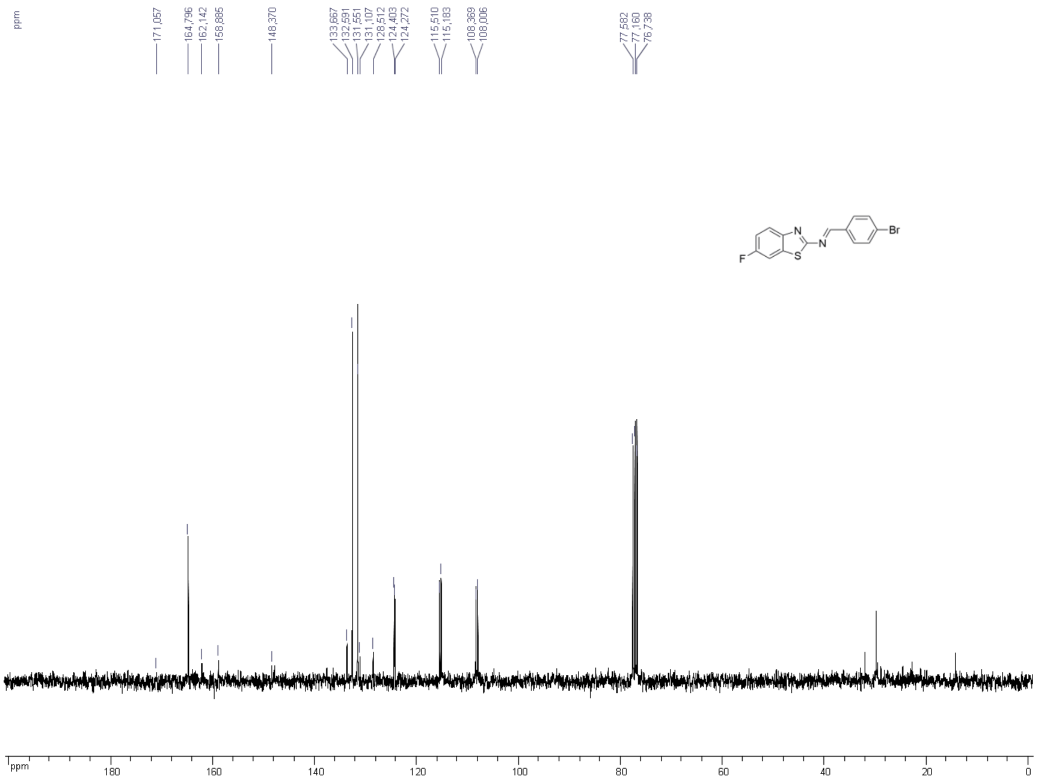

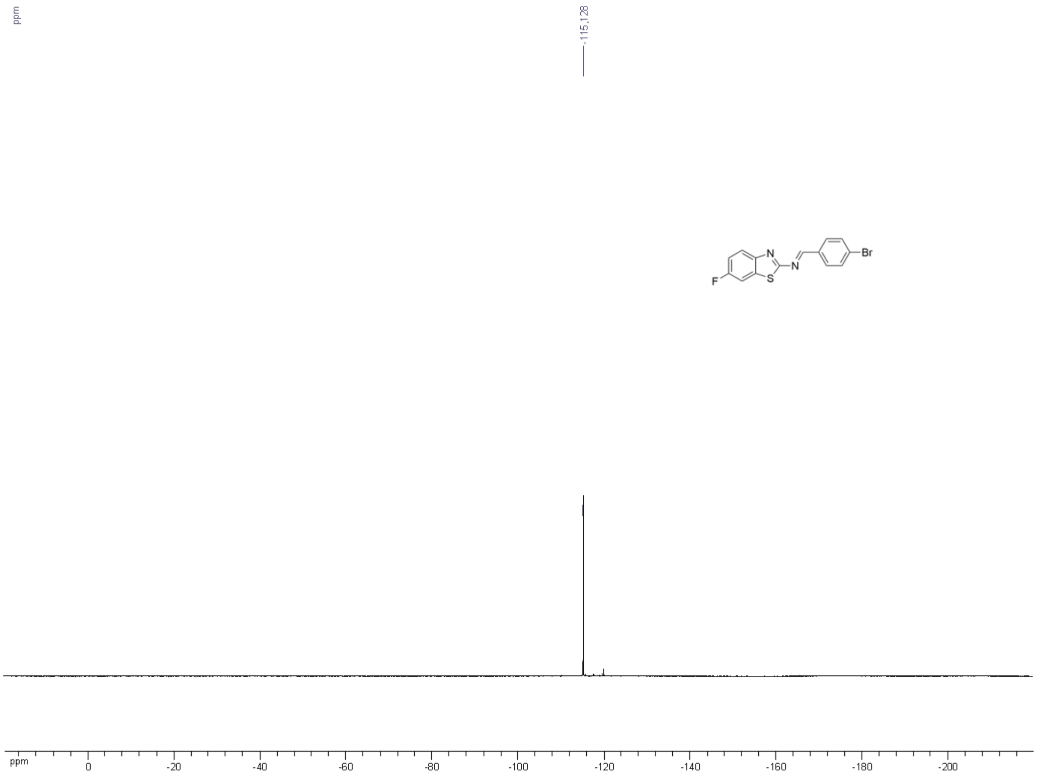
ppm
-115.128
F
N
S
N
Br
ppm
0
-20
-40
-60
-80
-100
-120
-140
-160
-180
-200

(*E*)-*N*-(6-bromobenzo[*d*]thiazol-2-yl)-1-(4-bromophenyl)methanimine

2e

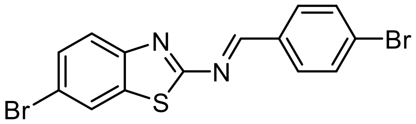

Compound **2e** was prepared according to the general procedure **C** from 4-bromobenzaldehyde (0.50 g, 2.70 mmol) and 2-amino-6-bromobenzothiazole (0.74 g, 3.24 mmol) as starting materials. Purification on a column of silica gel with a gradient of ethyl acetate in heptane (from 99:1 to 9:1) as eluent gave the desired product.

Chemical formula	$C_{14}H_8Br_2N_2S$
M (g.mol^{-1})	396.10
Yield	0.65 g, 61%
Aspect	Yellow solid
Rf	0.6 (Hept/AcOEt : 9/1)
^{1}H NMR (300 MHz, $CDCl_3$)	**δ :** 9.03 (s, 1H), 7.97 (d, *J* = 1.9 Hz, 1H), 7.88 (d, *J* = 8.7 Hz, 2H), 7.82 (d, *J* = 8.4 Hz, 1H), 7.66 (d, *J* = 8.4 Hz, 2H), 7.58 (dd, *J* = 8.4, 2.0 Hz, 1H)
^{13}C NMR (75 MHz, $CDCl_3$)	**δ :** 171.9 (C), 165.2 (CH), 150.7 (C), 136.5 (C), 133.6 (C), 132.6 (2 CH), 131.6 (2 CH), 130.2 (CH), 128.7 (C), 124.4 (2 CH), 118.8 (C)
IR (Neat, cm^{-1})	3079, 2978, 2867, 1618, 1600, 1579, 1560, 1542, 1494, 1480, 1466, 1426, 1393, 1363, 1301, 1287, 1276, 1250, 1226, 1149, 1102, 1081, 1066, 1047, 1004
HRMS (ESI+, m/z)	$[M+H]^+$ calcd. for $C_{14}H_9Br_2N_2S$ 394.8853, found 394.8869

(*E*)-*N*-(6-bromobenzo[*d*]thiazol-2-yl)-1-(4-bromophenyl)methanimine

2e

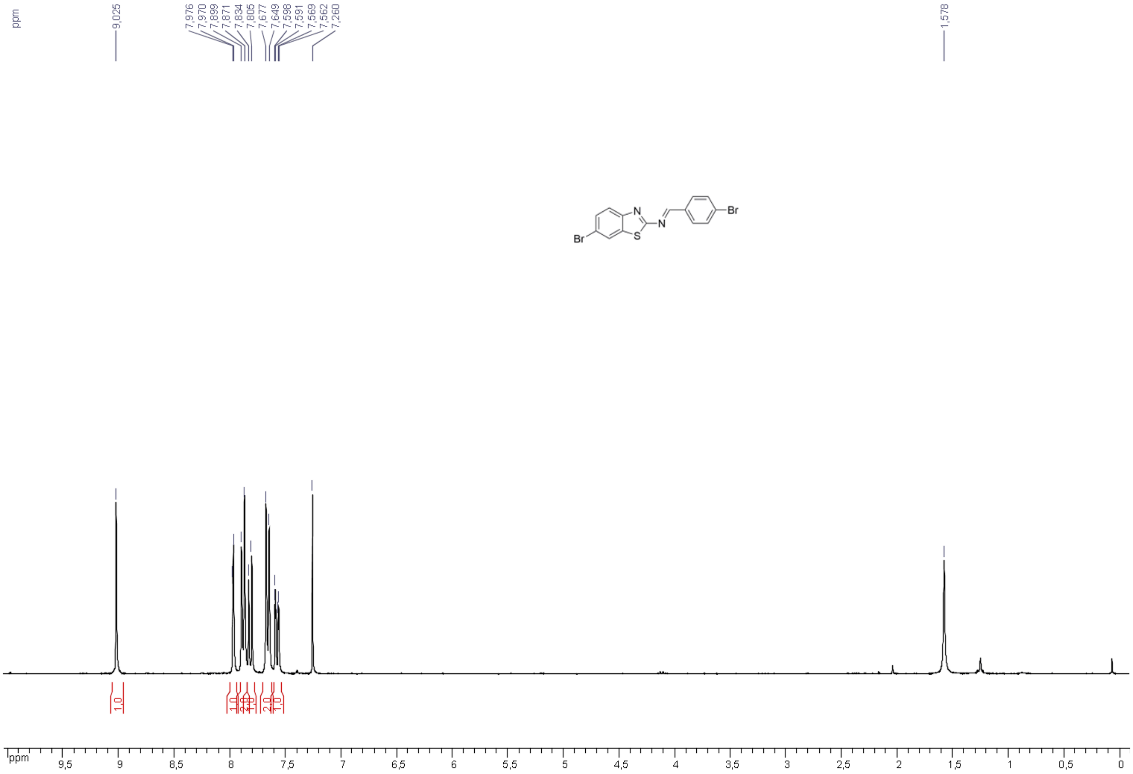

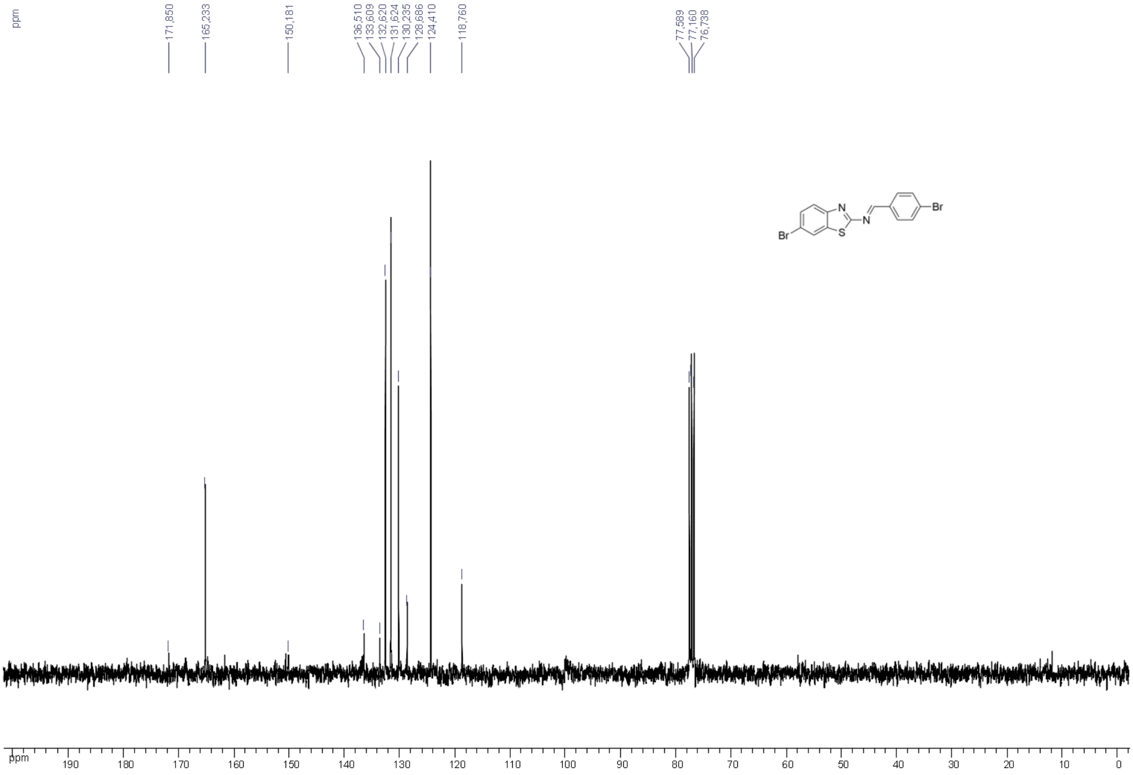

(E)-1-(4-bromophenyl)-N-(6-chlorobenzo[d]thiazol-2-yl)methanimine

2f

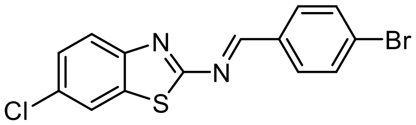

Compound **2f** was prepared according to the general procedure **C** from 4-bromobenzaldehyde (0.50 g, 2.70 mmol) and 2-amino-6-chlorobenzothiazole (0.60 g, 3.24 mmol) as starting materials. Purification on a column of silica gel with a gradient of ethyl acetate in heptane (from 99:1 to 75: 25) as eluent gave the desired product.

Chemical formula	$C_{14}H_8BrClN_2S$
M (g.mol^{-1})	351.65
Yield	0.93 g, 98%
Aspect	Yellow solid
Rf	0.5 (Hept/AcOEt : 9/1)
^{1}H NMR (300 MHz, CDCl$_3$)	**δ :** 9.02 (s, 1H), 7.89 (d, *J* = 8.3 Hz, 2H), 7.88 (d, *J* = 8.9 Hz, 1H), 7.82 (d, *J* = 2.1 Hz, 1H), 7.67 (d, *J* = 8.3 Hz, 2H), 7.44 (dd, *J* = 8.7, 2.0 Hz, 1H)
^{13}C NMR (75 MHz, CDCl$_3$)	**δ :** 171.8 (C), 165.2 (CH), 150.4 (C), 136.1 (C), 133.6 (C), 132.6 (2 CH), 131.6 (2 CH), 131.1 (C), 128.7 (C), 127.5 (CH), 124.1 (CH), 121.5 (CH)
IR (Neat, cm^{-1})	3079, 2922, 2863, 1723, 1614, 1600, 1584, 1560, 1549, 1496, 1485, 1438, 1429, 1398, 1366, 1305, 1273, 1283, 1228, 1153, 1102, 1065, 1049, 1007
HRMS (ESI+, m/z)	$[M+H]^+$ calcd. for $C_{14}H_9BrClN_2S$ 350.9361, found 350.9358

(*E*)-1-(4-bromophenyl)-*N*-(6-chlorobenzo[*d*]thiazol-2-yl)methanimine 2f

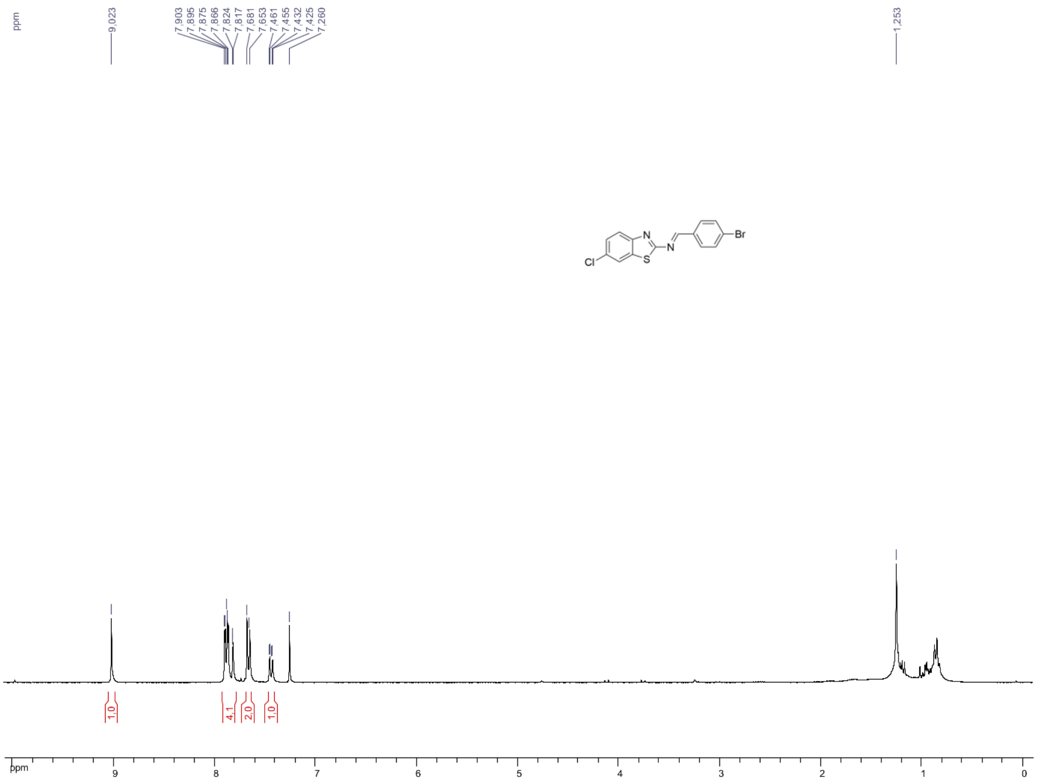

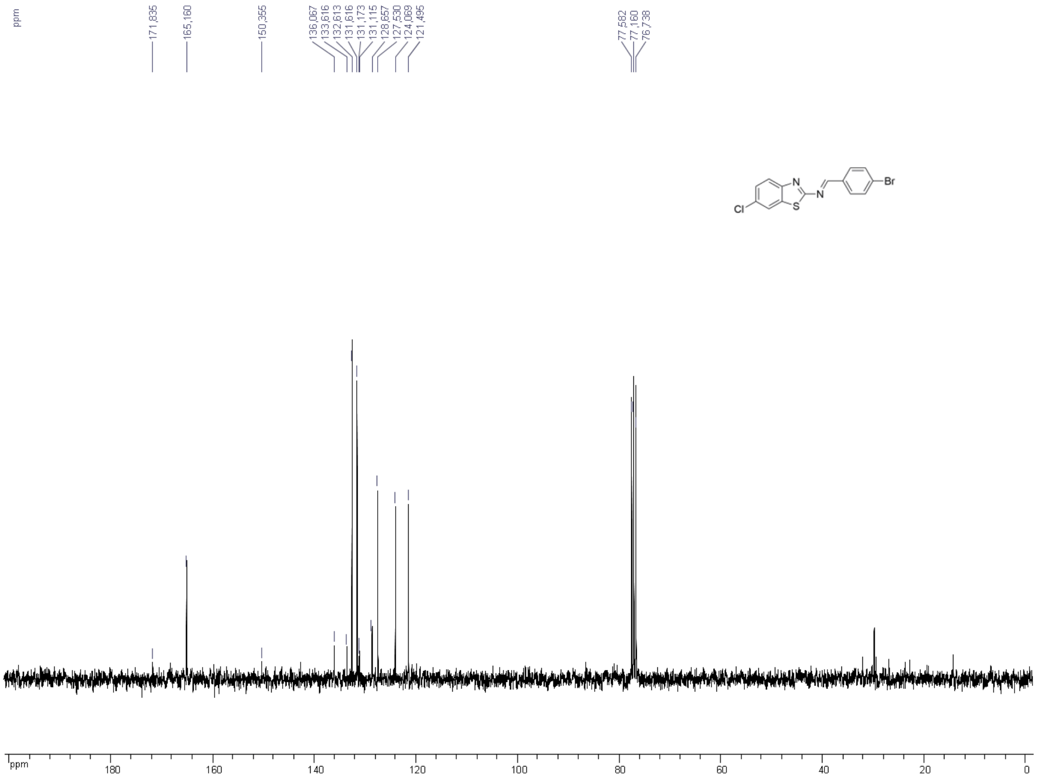

(*E*)-4-(((6-fluorobenzo[*d*]thiazol-2-yl)imino)methyl)benzonitrile

2i

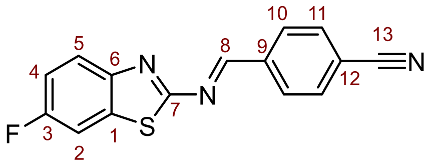

Compound **2i** was prepared according to the general procedure **C** from 4-cyanobenzaldehyde (0.50 g, 3.82 mmol) and 2-amino-6-fluorobenzothiazole (0.77 g, 4.58 mmol) as starting materials and purified by precipitation.

Chemical formula	$C_{15}H_8FN_3S$
M (g.mol^{-1})	281.31
Yield	0.16 g, 15%
Aspect	Yellow solid
Rf	0.2 (Hept/AcOEt : 9/1)
^{1}H NMR (300 MHz, CDCl$_3$)	**δ :** 9.13 (s, 1H), 8.14 (d, *J* = 8.4 Hz, 2H), 7.96 (dd, *J* = 8.9, 4.6 Hz, 1H), 7.82 (d, *J* = 8.3 Hz, 2H), 7.56 (dd, *J* = 8.0, 2.6 Hz, 1H), 7.26 (td, *J* = 8.9, 2.6 Hz, 1H)
^{13}C NMR (75 MHz, CDCl$_3$)	**δ :** 170.4 (C), 163.6 (CH), 160.7 (d, *J* = 246.1 Hz, CF), 148.3 (C), 138.4 (C), 136.2 (C), 132.9 (2 CH), 130.4 (2 CH), 124.7 (d, *J* = 10.0 Hz, CH), 118.2 (C), 116.3 (C), 115.7 (d, *J* = 24.5 Hz, CH), 108.3 (d, *J* = 27.0 Hz, CH)
^{19}F NMR (282 MHz, CDCl$_3$)	**δ :** -114.41
IR (Neat, cm^{-1})	3083, 3051, 2862, 2230, 1897, 1615, 1603, 1558, 1509, 1486, 1452, 1411, 1357, 1317, 1289, 1255, 1227, 1199, 1151, 1119, 1108, 1046, 1013
HRMS (ESI+, m/z)	$[M+H]^+$ calcd. for $C_{15}H_9FN_3S$ 282.0501, found 282.0511

(*E*)-4-(((6-fluorobenzo[*d*]thiazol-2-yl)imino)methyl)benzonitrile

2i

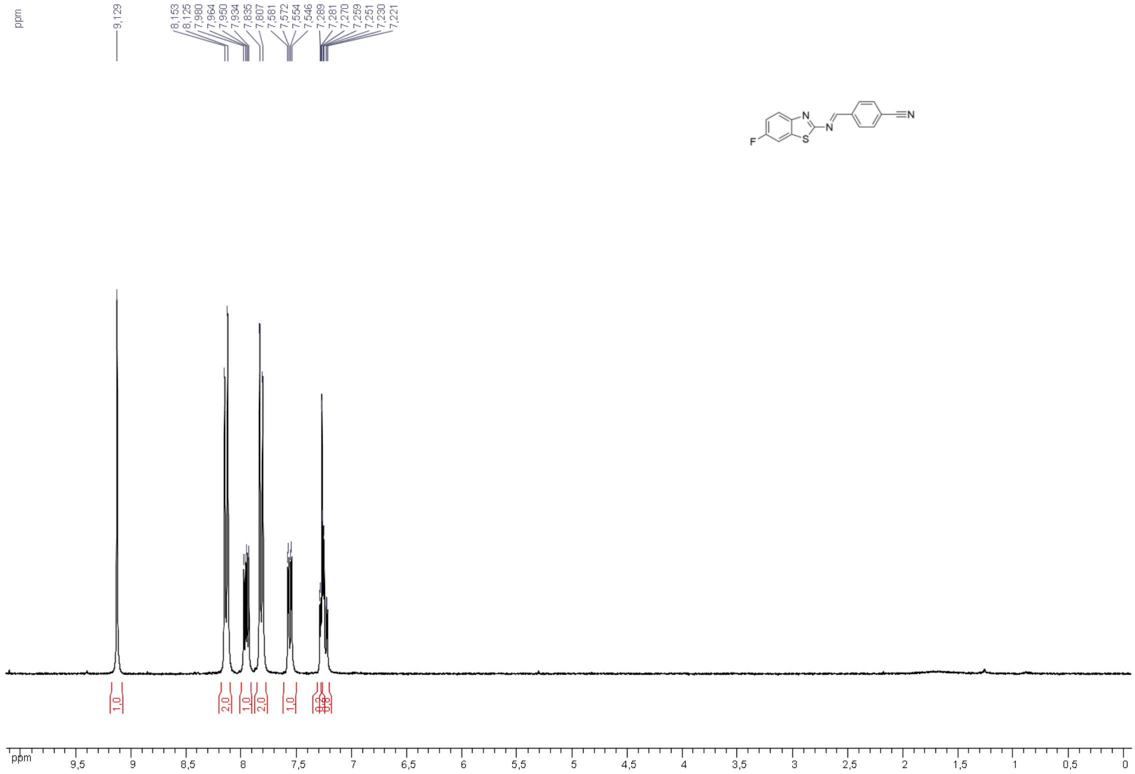

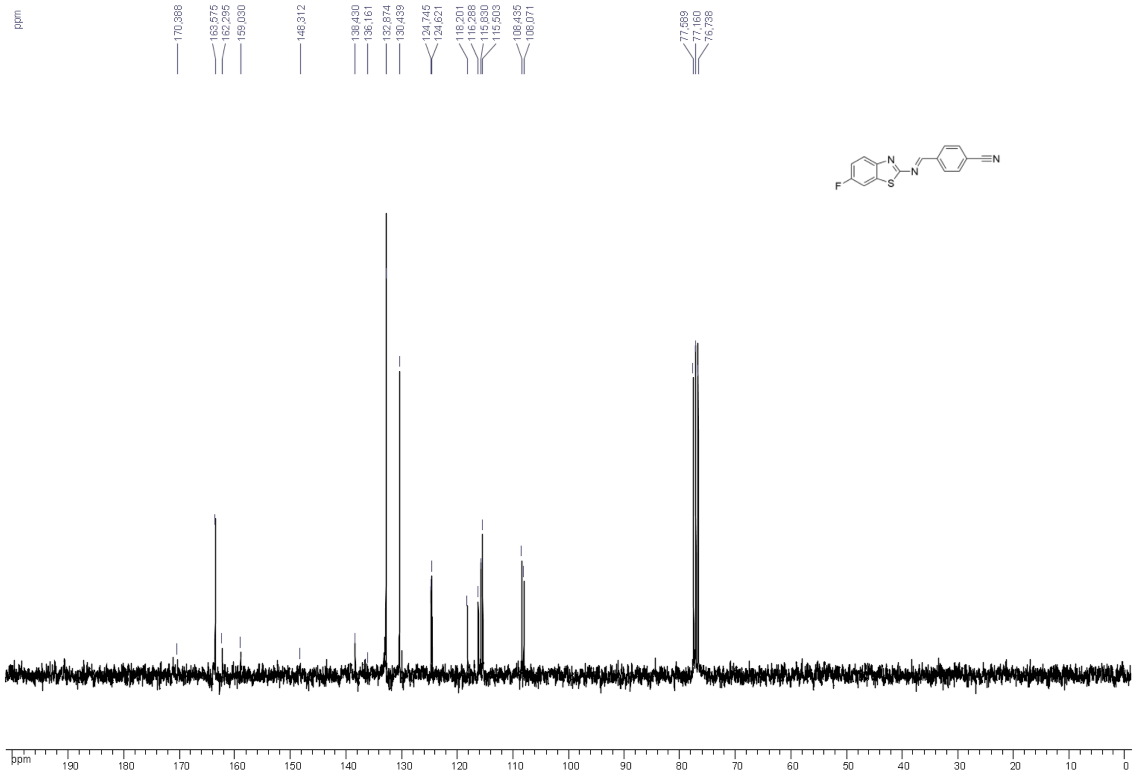

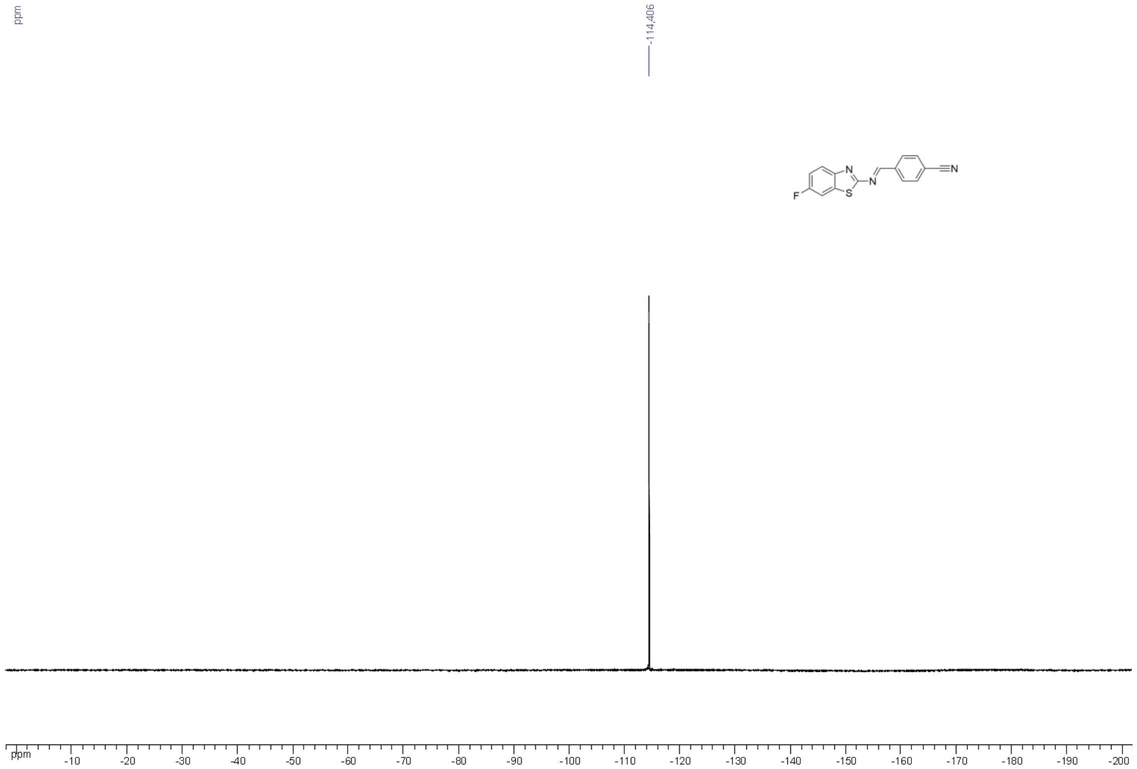

ppm
-114.406
F
N
S
N
N
ppm
-10
-20
-30
-40
-50
-60
-70
-80
-90
-100
-110
-120
-130
-140
-150
-160
-170
-180
-190
-200

(*E*)-1-(4-bromo-2-chlorophenyl)-*N*-(6-fluorobenzo[*d*]thiazol-2-yl)methanimine

2j

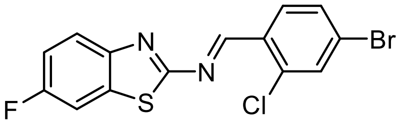

Compound **2j** was prepared according to the general procedure **C** from 4-bromo-2-chlorobenzaldehyde (0.37 g, 1.70 mmol) and 2-amino-6-fluorobenzothiazole (0.35 g, 2.08 mmol) as starting materials and purified by precipitation.

Chemical formula	$C_{14}H_7BrClFN_2S$
M (g.mol^{-1})	369.64
Yield	0.12 g, 19%
Aspect	Yellow solid
Rf	0.7 (Hept/AcOEt : 9/1)
^{1}H NMR (300 MHz, CDCl$_3$)	**δ :** 9.42 (s, 1H), 8.24 (d, *J* = 8.5 Hz, 1H), 7.95 (dd, *J* = 8.9, 4.9 Hz, 1H), 7.67 (d, *J* = 1.8 Hz, 1H), 7.55 (ddd, *J* = 8.4, 1.8, 0.8 Hz, 1H), 7.54 (dd, *J* = 8.1, 2.7 Hz, 1H), 7.23 (td, *J* = 8.7, 3.0 Hz, 1H)
^{13}C NMR (75 MHz, CDCl$_3$)	**δ :** 170.8 (C), 161.7 (CH), 160.6 (d, *J* = 250.8 Hz, CF), 148.4 (C), 138.3 (C), 136.0 (C), 133.1 (CH), 131.0 (CH and C), 130.5 (CH), 128.3 (C), 124.6 (d, *J* = 9.2 Hz, CH), 115.5 (d, *J* = 24.4 Hz, CH), 108.2 (d, *J* = 27.3 Hz, CH)
^{19}F NMR (282 MHz, CDCl$_3$)	**δ :** -114.72
IR (Neat, cm^{-1})	3092, 2993, 1944, 1913, 1733, 1698, 1612, 1588, 1572, 1543, 1481, 1463, 1442, 1412, 1376, 1362, 1320, 1308, 1283, 1263, 1248, 1198, 1170, 1158, 1126, 1077, 1051, 1044
HRMS (ESI+, m/z)	$[M+H]^+$ calcd. for $C_{14}H_8BrClFN_2S$ 368.9264, found 368.9273

(*E*)-1-(4-bromo-2-chlorophenyl)-*N*-(6-fluorobenzo[*d*]thiazol-2-yl)methanimine 2j

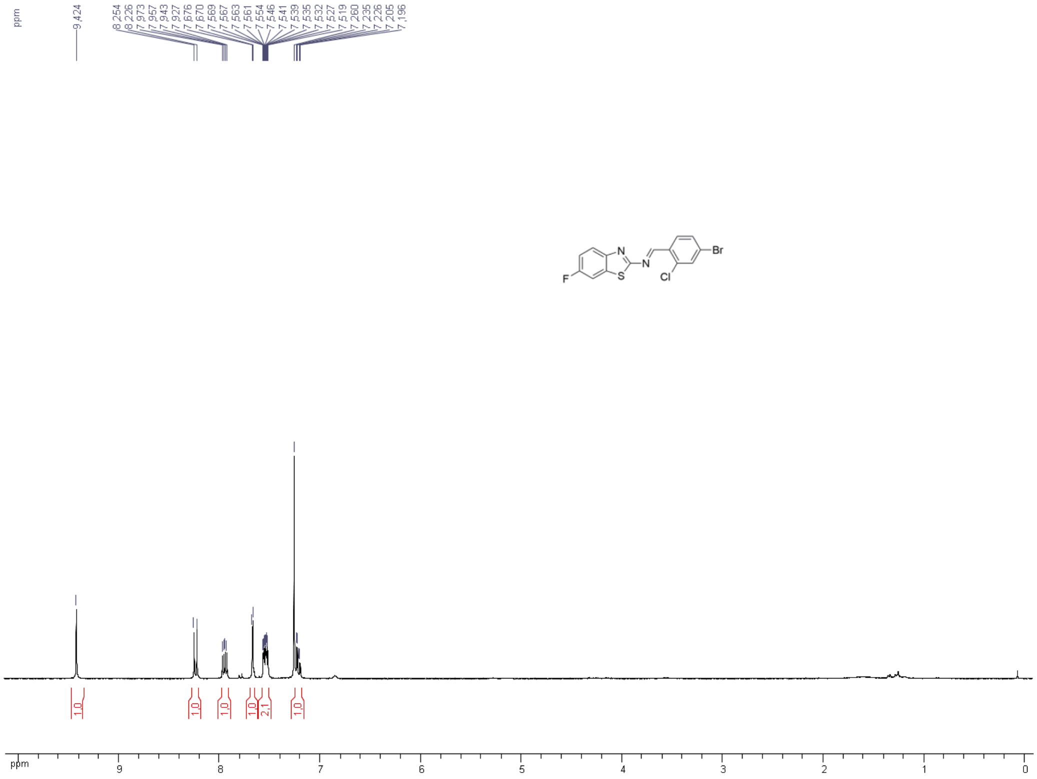

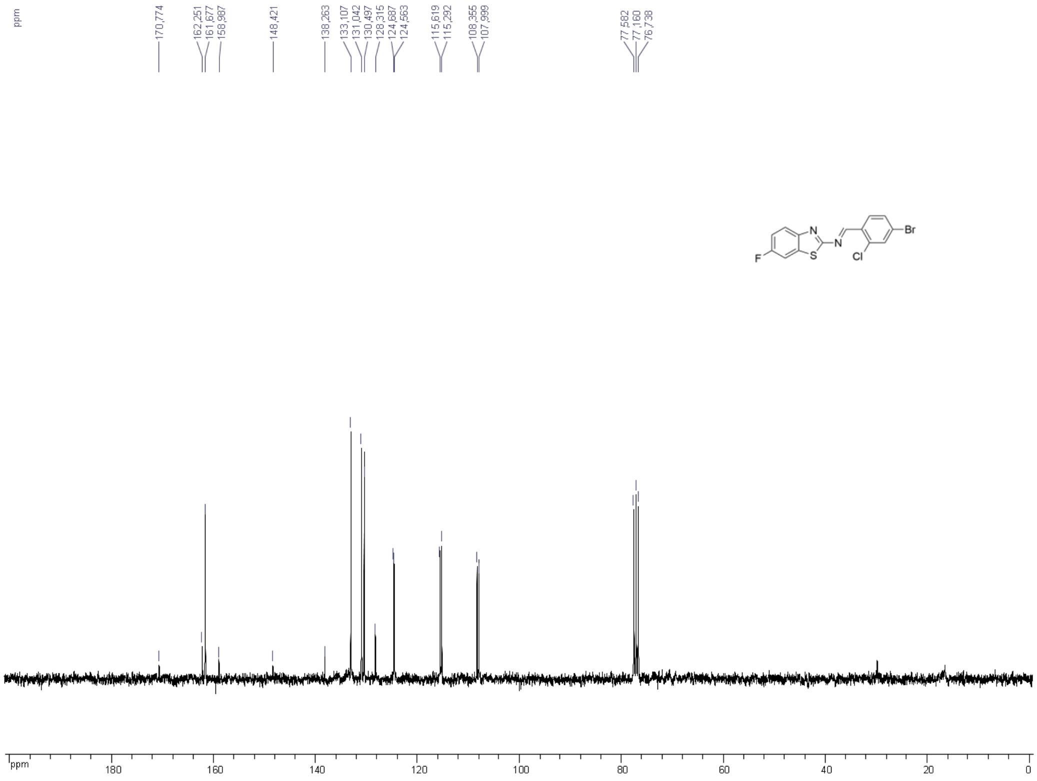

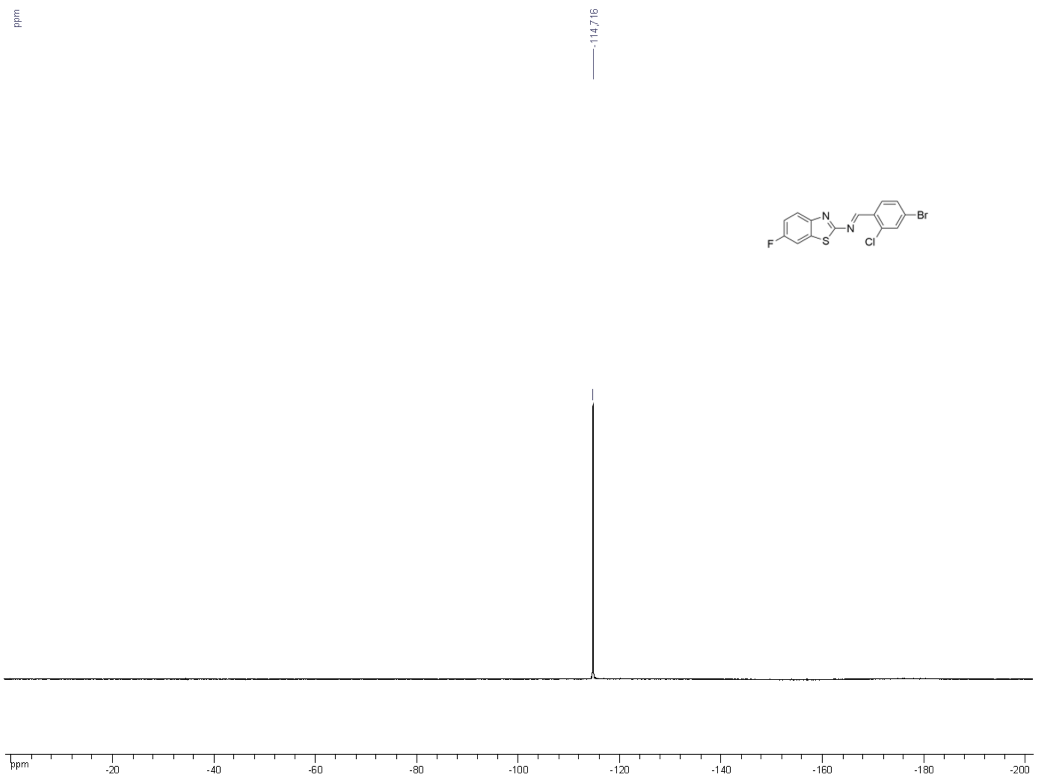
ppm
-114.716
F
N
S
N
Cl
Br
ppm
-20
-40
-60
-80
-100
-120
-140
-160
-180
-200

(*E*)-1-(3-bromophenyl)-*N*-(6-methoxybenzo[*d*]thiazol-2-yl)methanimine
2k

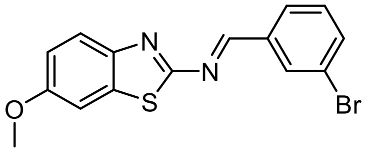

Compound **2k** was prepared according to the general procedure **C** from 3-bromobenzaldehyde (0.50 g, 2.70 mmol) and 2-amino-6-methoxybenzothiazole (0.58 g, 3.24 mmol) as starting materials. Purification on a column of silica gel with a gradient of ethyl acetate in heptane (from 9:1 to 8:2) as eluent gave the desired product.

Chemical formula	$C_{15}H_{11}BrN_2OS$
M (g.mol^{-1})	347.23
Yield	0.84 g, 90%
Aspect	Yellow solid
MP	140-142 °C
Rf	0.3 (Hept/AcOEt : 9/1)
^{1}H NMR (300 MHz, CDCl$_3$)	**δ :** 8.97 (s, 1H), 8.21 (t, *J* = 1.8 Hz, 1H), 7.90 (dt, *J* = 7.7, 1.2 Hz, 1H), 7.87 (d, *J* = 9.0 Hz, 1H), 7.67 (ddd, *J* = 8.0, 2.1, 1.0 Hz, 1H), 7.38 (t, *J* = 7.9 Hz, 1H), 7.30 (d, *J* = 2.6 Hz, 1H), 7.09 (dd, *J* = 8.9, 2.6 Hz, 1H), 3.89 (s, 3H)
^{13}C NMR (75 MHz, CDCl$_3$)	**δ :** 168.8 (C), 163.2 (CH), 158.0 (C), 146.2 (C), 137.0 (C), 136.3 (C), 135.8 (CH), 132.4 (CH), 130.6 (CH), 129.0 (CH), 124.1 (CH), 123.4 (C), 116.1 (CH), 104.5 (CH), 56.0 (CH_3)
IR (Neat, cm^{-1})	3077, 3001, 2948, 2872, 1601, 1562, 1495, 1474, 1423, 1363, 1351, 1325, 1282, 1264, 1228, 1179, 1162, 1119, 1090, 1067, 1051, 1020
HRMS (ESI+, m/z)	$[M+H]^+$ calcd. for $C_{15}H_{12}BrN_2OS$ 346.9854, found 346.9870

(*E*)-1-(3-bromophenyl)-*N*-(6-methoxybenzo[*d*]thiazol-2-yl)methanimine

2k

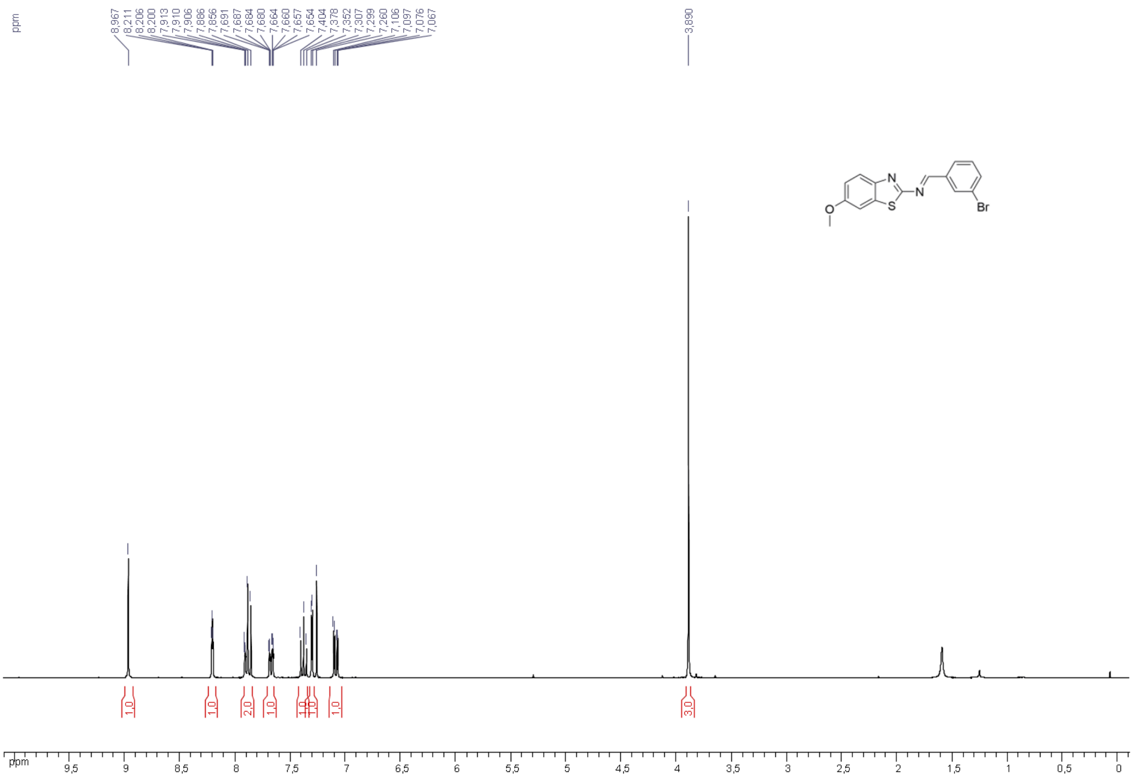

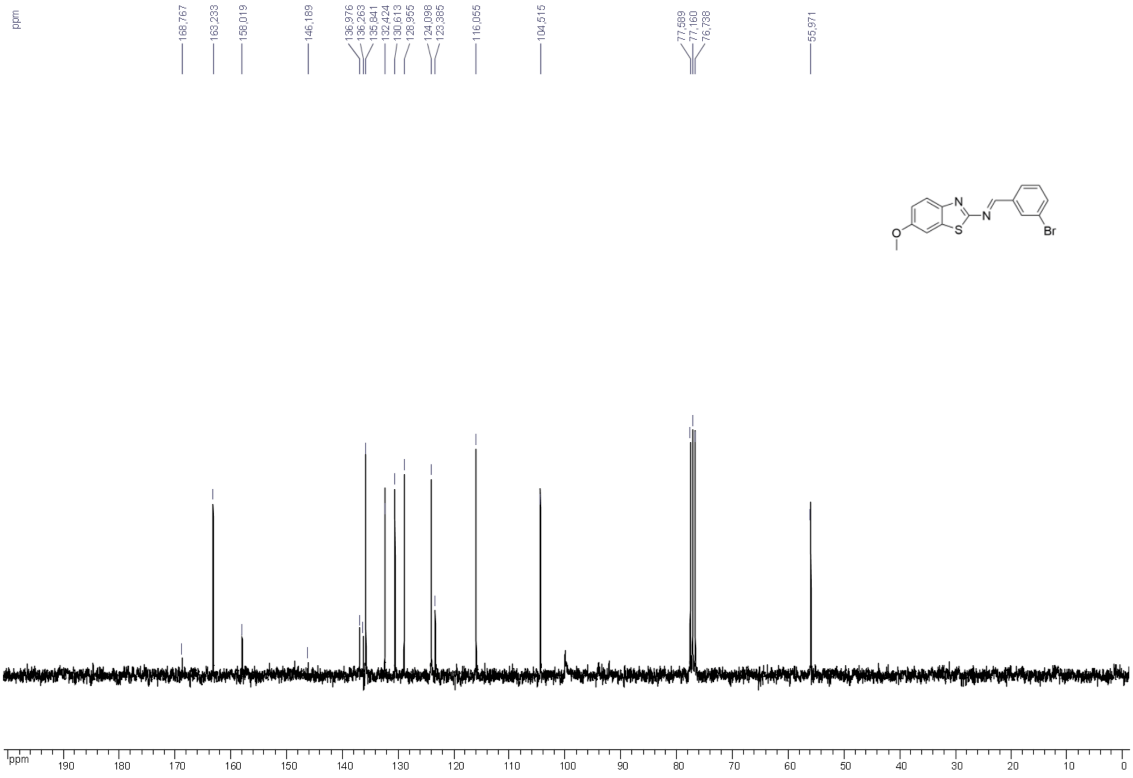

V. Synthesis and Characterization of cycloadducts 4

benzyl ((2*R*,3*S*,4*R*)-2-(4-bromophenyl)-8-methoxy-3-methyl-3,4-dihydro-2*H*-benzo[4,5]thiazolo[3,2-*a*]pyrimidin-4-yl)carbamate

4a

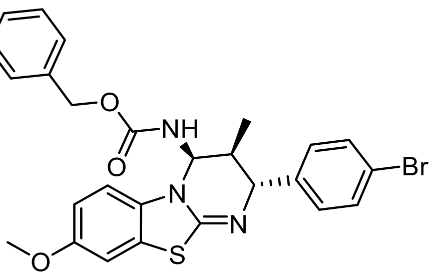

Compound **4a** was prepared according to the general procedure **D** from 2-benzothiazolimine **2a** (34.7 mg, 0.1 mmol) and enecarbamate **3a** (57.4 mg, 0.3 mmol) as starting materials. Purification on a column of silica gel with a gradient of ethyl acetate in heptane (8:2) as eluent gave the desired product as only one diastereomer.

Chemical formula	$C_{26}H_{24}BrN_3O_3S$
M (g.mol^{-1})	538.46
Yield	48.5 mg, 90%
Aspect	White foam
Rf	0.2 (Hept/AcOEt : 7/3)
^{1}H NMR (300 MHz, CD_3CN)	**δ :** 7.50 (d, *J* = 8.6 Hz, 2H), 7.35 (s, 5H), 7.19 (d, *J* = 8.4 Hz, 2H), 6.99 (d, *J* = 2.4 Hz, 1H), 6.94 (d, *J* = 8.6 Hz, 1H), 6.78 (dd, *J* = 8.9, 2.4 Hz, 1H), 6.49 (d, *J* = 10.0 Hz, 1H, NH), 5.77 (dd, *J* = 10.0, 4.4 Hz, 1H), 5.15 (dd, *J* = 19.7, 11.9 Hz, 2H), 4.1 (d, *J* = 11.3 Hz, 1H), 3.76 (s, 3H), 2.11-2.06 (m, 1H), 0.71 (d, *J* = 6.8 Hz, 3H)
^{13}C NMR (75 MHz, CD_3CN)	**δ :** 157.4 (C), 157.1 (C), 156.7 (C), 144.0 (C), 138.0 (C), 134.2 (C), 132.1 (2 CH), 130.9 (2 CH), 129.5 (2 CH), 129.0 (CH), 128.7 (2 CH), 124.1 (C), 121.2 (C), 112.8 (CH), 110.4 (CH), 109.2 (CH), 67.5 (CH_2), 63.0 (CH), 61.7 (CH), 56.5 (CH_3), 37.1 (CH), 13.5 (CH_3)
IR (Neat, cm^{-1})	2960, 1717, 1622, 1586, 1538, 1487, 1282, 1239, 1201, 1073, 1033, 1010
HRMS (ESI+, m/z)	$[M+H]^+$ calcd. for $C_{26}H_{25}BrN_3O_3S$ 538.0800, found 538.0812
HPLC Analysis	Daicel Chiralpak IA, Heptane/EtOH = 95/5, flow rate = 1 mL/min, 254 nm<br>major isomer: t_R = 29.19 min, minor isomer: t_R = 40.49 min
Enantiomeric excess	99%
$[\alpha]_D^{22}$	-96.00° (*c* 0.3, $CHCl_3$)

benzyl ((2*R*,3*S*,4*R*)-2-(4-bromophenyl)-8-methoxy-3-methyl-3,4-dihydro-2*H*-benzo[4,5]thiazolo[3,2-*a*]pyrimidin-4-yl)carbamate

4a

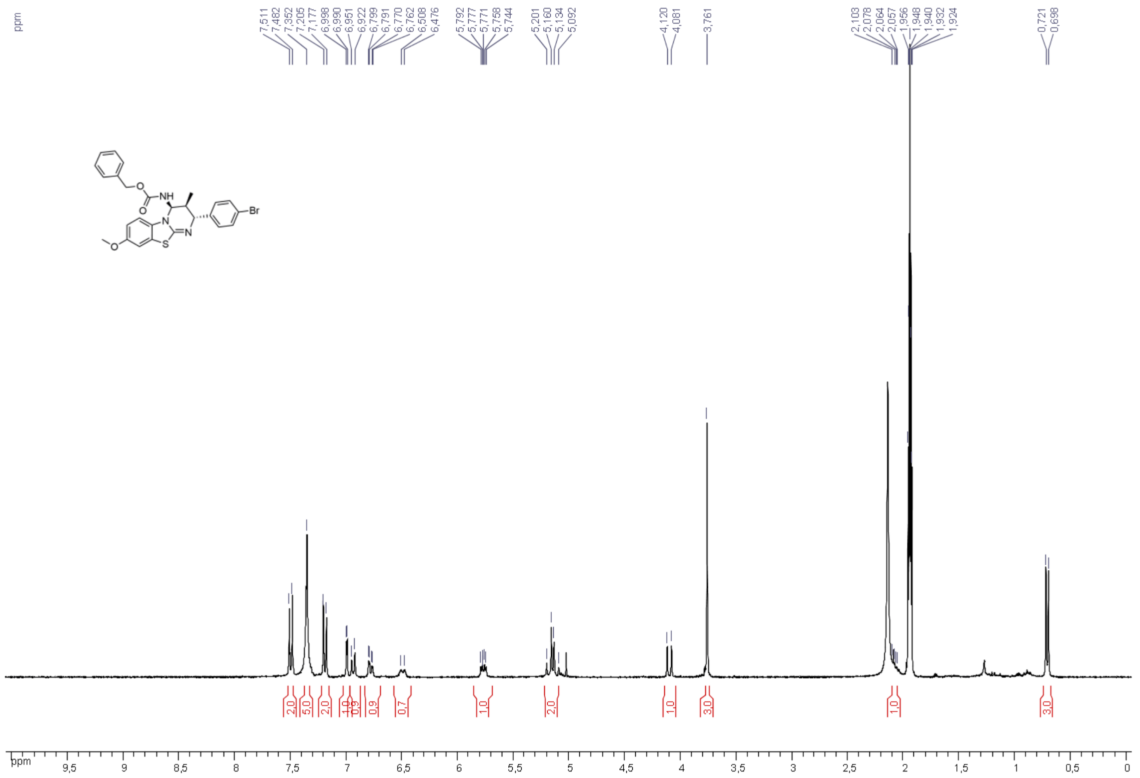

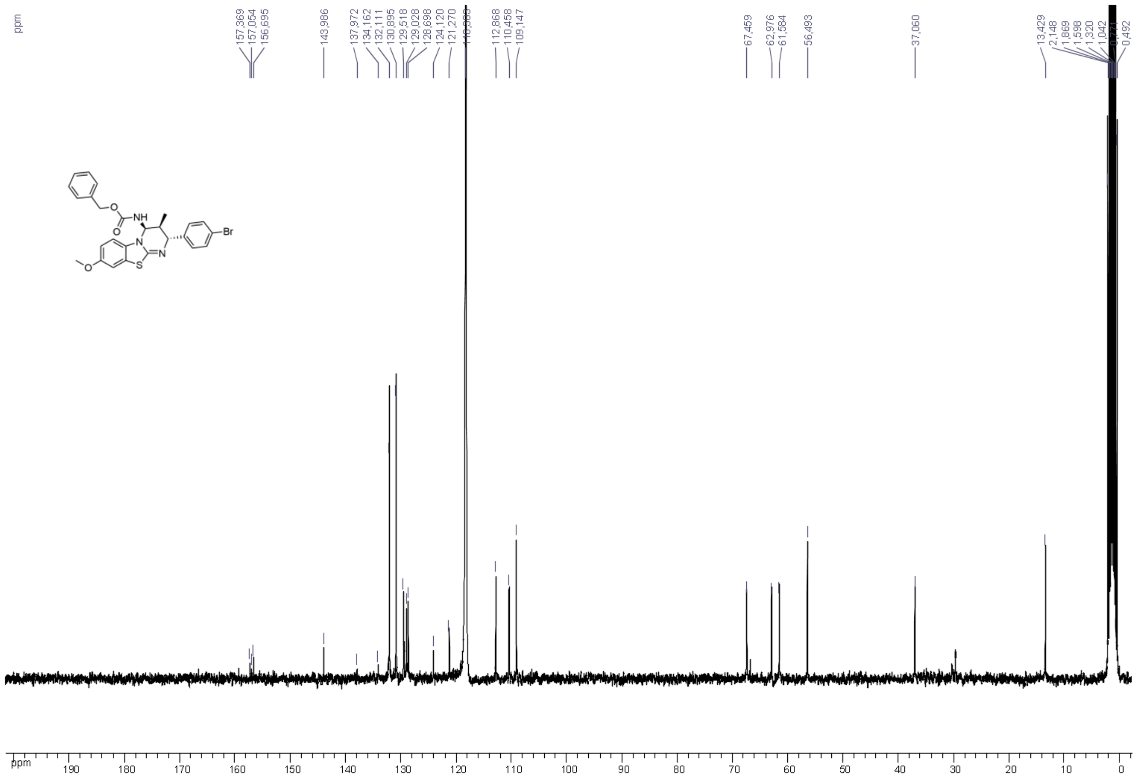

benzyl ((2*R*,3*S*,4*R*)-2-(4-bromophenyl)-8-methoxy-3-methyl-3,4-dihydro-2*H*-benzo[4,5]thiazolo[3,2-*a*]pyrimidin-4-yl)carbamate
4a

Chiralpak IA, Heptane/EtOH = 95/5, 1 mL/min, 254 nm

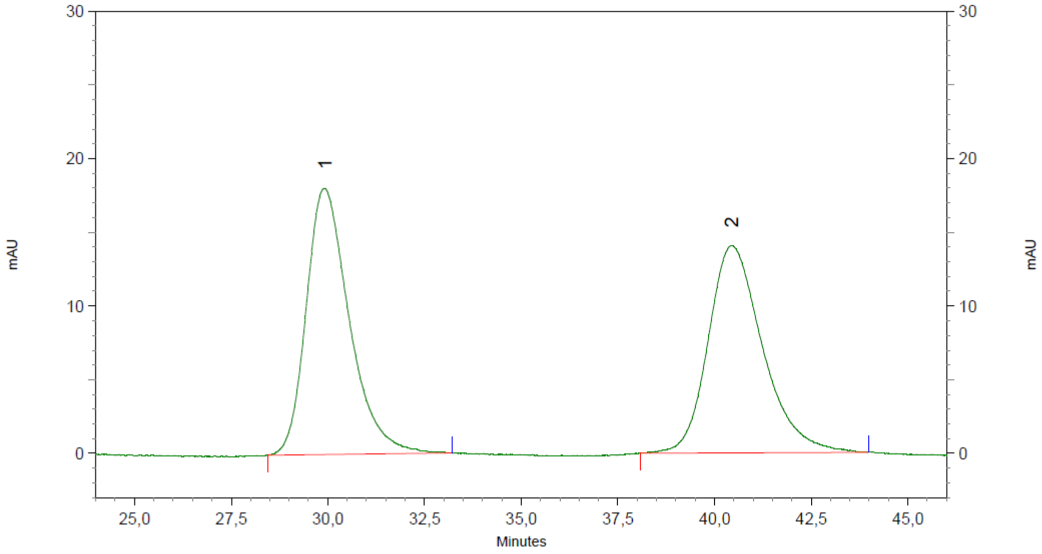

DAD-CH1 254 nm
Results

Pk #	Retention Time	Area	Area %
1	29,92	5586302	50,38
2	40,43	5501835	49,62

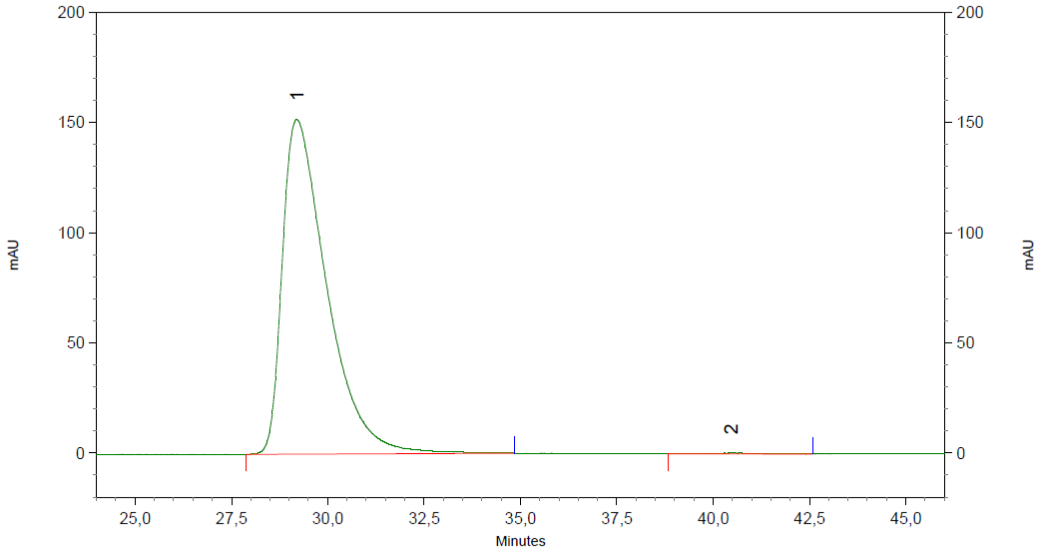

DAD-CH1 254 nm
Results

Pk #	Retention Time	Area	Area %
1	29,19	48602338	99,70
2	40,46	147610	0,30

benzyl ((2*R*,3*S*,4*R*)-2-(4-bromophenyl)-3,8-dimethyl-3,4-dihydro-2*H*-benzo[4,5]thiazolo[3,2-*a*]pyrimidin-4-yl)carbamate

4b

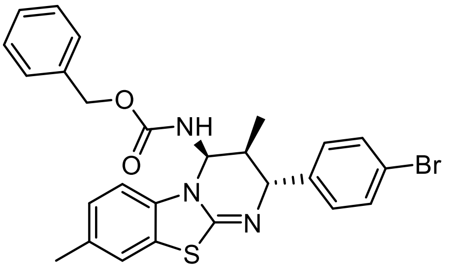

Compound **4b** was prepared according to the general procedure **D** from 2-benzothiazolimine **2b** (33.5 mg, 0.1 mmol) and enecarbamate **3a** (42.3 mg, 0.3 mmol) as starting materials. Purification on a column of silica gel with a gradient of ethyl acetate in heptane (8:2) as eluent gave the desired product as only one diastereomer.

Chemical formula	$C_{26}H_{24}BrN_3O_2S$
M (g.mol^{-1})	522.46
Yield	43.2 mg, 83%
Aspect	White foam
Rf	0.4 (Hept/AcOEt : 7/3)
^{1}H NMR *(300 MHz, Acetone-d$_6$)*	**δ :** 7.85 (d, *J* = 10.7 Hz, 1H, NH), 7.46 (d, *J* = 8.8 Hz, 2H), 7.39-7.29 (m, 5H), 7.14 (d, *J* = 8.7 Hz, 2H), 7.04 (d, *J* = 1.3 Hz, 1H), 7.03 (dd, *J* = 7.9, 2.0 Hz, 1H), 7.02 (d, *J* = 7.7 Hz, 1H), 5.83 (dd, *J* = 10.2, 4.3 Hz, 1H), 5.20 (dd, *J* = 19.8, 12.6 Hz, 2H), 4.14 (d, *J* = 11.6 Hz, 1H), 2.32 (s, 3H), 2.02-1.97 (m, 1H) , 0.65 (s, 3H)
^{13}C NMR *(75 MHz, Acetone-d$_6$)*	**δ :** 157.4 (C), 157.0 (C), 144.0 (C), 138.2 (C), 138.0 (C), 132.6 (C), 131.8 (2 CH), 131.0 (2 CH), 129.2 (2 CH), 128.7 (CH), 128.6 (2 CH), 127.5 (CH), 123.1 (CH), 122.8 (C), 121.1 (C), 109.9 (CH), 67.1 (CH_2), 62.9 (CH), 61.5 (CH), 37.4 (CH), 20.9 (CH_3), 13.5 (CH_3)
IR (Neat, cm^{-1})	2962, 1715, 1619, 1535, 1487, 1456, 1319, 1250, 1227, 1200, 1065, 1010
HRMS (ESI+, m/z)	$[M+H]^+$ calcd. for $C_{26}H_{25}BrN_3O_2S$ 522.0851, found 522.0872
HPLC Analysis	Daicel Chiralpak IA, Heptane/EtOH = 90/10, flow rate = 1 mL/min, 254 nm<br>major isomer: t_R = 12.40 min, minor isomer: t_R = 16.89 min
Enantiomeric excess	98%
$[\alpha]_D^{22}$	-141.00° (*c* 0.3, $CHCl_3$)

benzyl ((2*R*,3*S*,4*R*)-2-(4-bromophenyl)-3,8-dimethyl-3,4-dihydro-2*H*-benzo[4,5]thiazolo[3,2-*a*]pyrimidin-4-yl)carbamate

4b

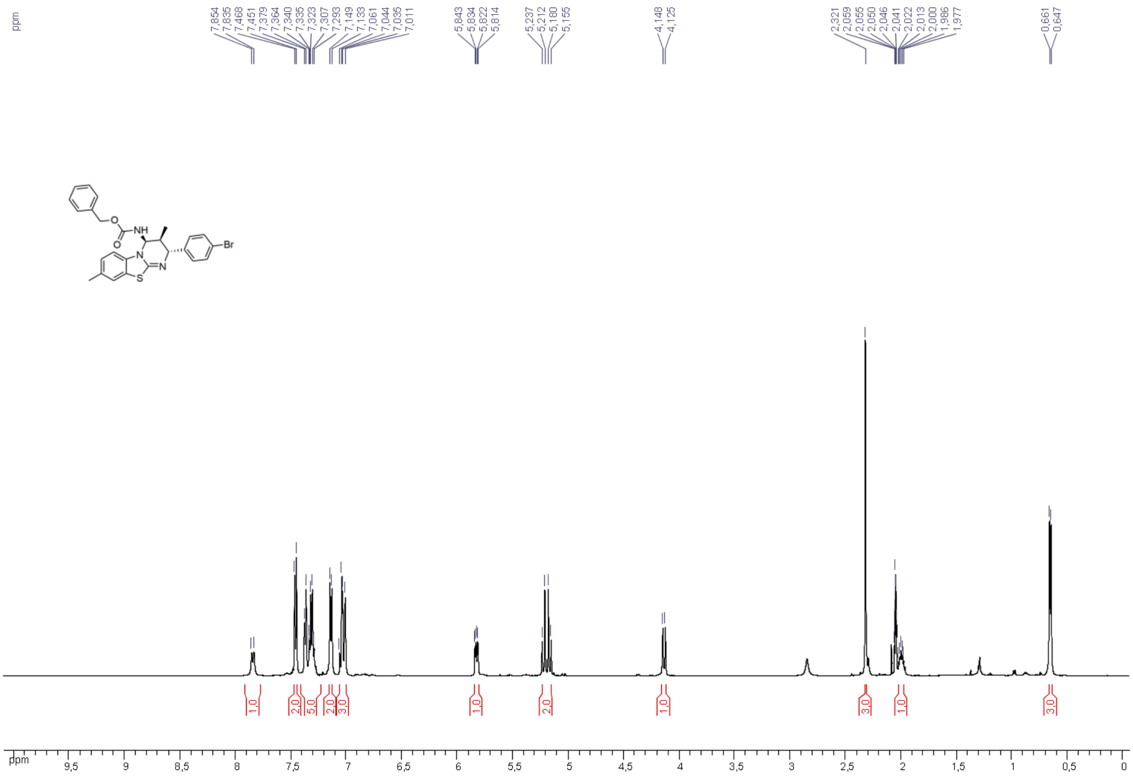

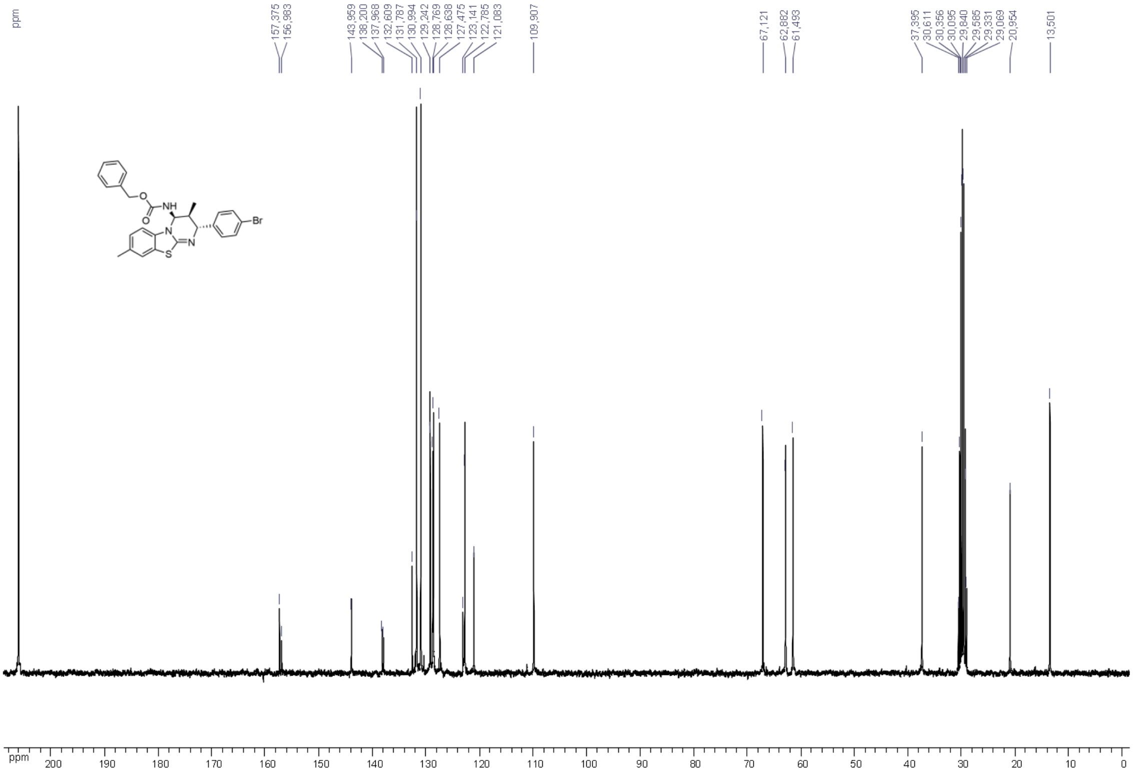

benzyl ((2*R*,3*S*,4*R*)-2-(4-bromophenyl)-3,8-dimethyl-3,4-dihydro-2*H*-benzo[4,5]thiazolo[3,2-*a*]pyrimidin-4-yl)carbamate

4b

Chiralpak IA, Heptane/EtOH = 90/10, 1 mL/min, 254 nm

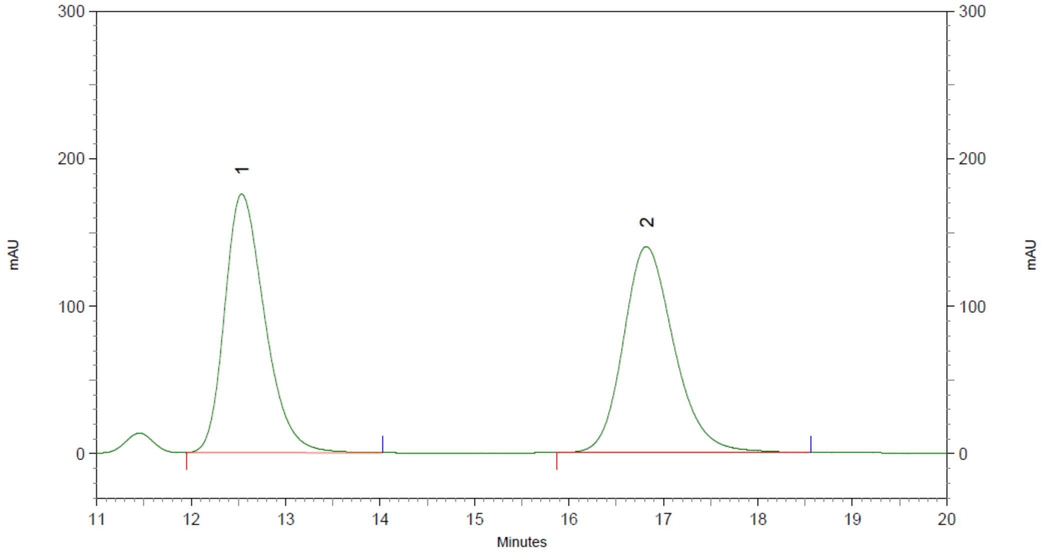

DAD-CH1 254 nm

Results

Pk #	Retention Time	Area	Area %
1	12,53	20634153	49,67
2	16,82	20910372	50,33

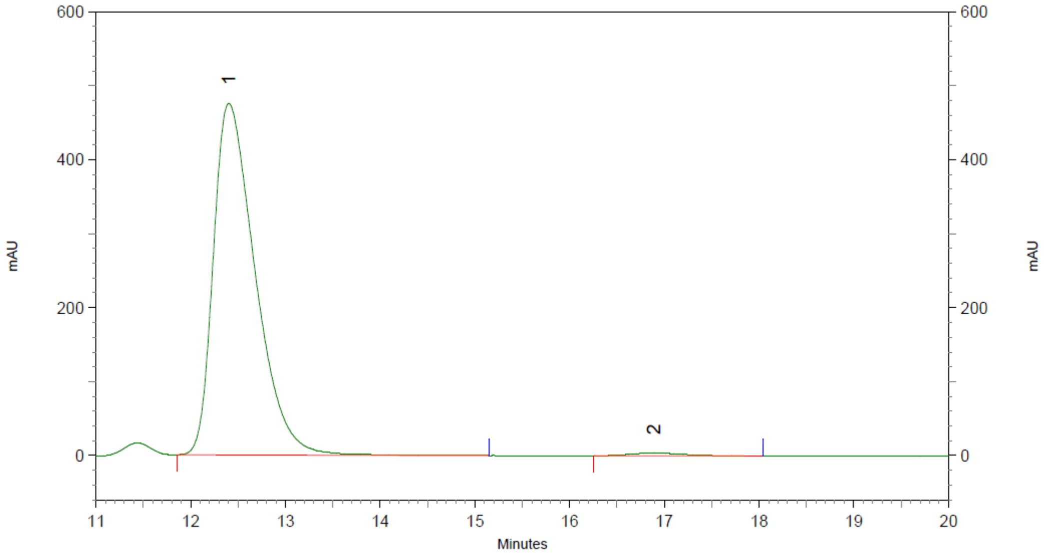

DAD-CH1 254 nm

Results

Pk #	Retention Time	Area	Area %
1	12,40	58171640	98,99
2	16,89	592182	1,01

benzyl ((2*R*,3*S*,4*R*)-2-(4-bromophenyl)-3-methyl-8-(trifluoromethyl)-3,4-dihydro-2*H*-benzo[4,5]thiazolo[3,2-*a*]pyrimidin-4-yl)carbamate

4c

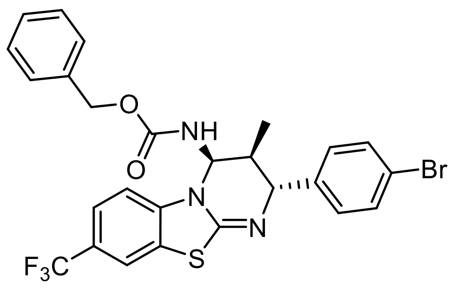

Compound **4c** was prepared according to the general procedure **D** from 2-benzothiazolimine **2c** (38.5 mg, 0.1 mmol) and enecarbamate **3a** (57.4 mg, 0.3 mmol) as starting materials. Purification on a column of silica gel with a gradient of ethyl acetate in heptane (from 85:15) as eluent gave the desired product as only one diastereomer.

Chemical formula	$C_{26}H_{21}BrF_3N_3O_2S$
M (g.mol^{-1})	576.43
Yield	52.6 mg, 91%
Aspect	Colorless oil
Rf	0.8 (Hept/AcOEt : 7/3)
^{1}H NMR (300 MHz, Acetone-d$_6$)	**δ :** 7.63 (d, *J* = 8.5 Hz, 1H), 7.57 (d, *J* = 8.7 Hz, 2H), 7.50 (s, 1H), 7.45-7.30 (m, 7H), 7.15 (d, *J* = 8.7 Hz, 2H), 5.95 (dd, *J* = 10.2, 4.3 Hz, 1H), 5.27 (dd, *J* = 19.6, 12.4 Hz, 2H), 4.08 (d, *J* = 12.1 Hz, 1H), 2.10-2.07 (m, 1H), 0.69 (d, *J* = 6.6 Hz, 3H)
^{13}C NMR (75 MHz, Acetone-d$_6$)	**δ :** 157.4 (C), 156.2 (C), 143.4 (C), 143.2 (C), 137.8 (C), 131.9 (2 CH), 130.9 (2 CH), 129.2 (2 CH), 128.9 (CH), 128.8 (2 CH), 125.3 (q, *J* = 272.3 Hz, CF_3), 124.6 (q, *J* = 41.7 Hz, C), 124.5 (q, *J* = 3.6 Hz, CH), 124.0 (C), 121.3 (C), 119.6 (q, *J* = 4.0 Hz, CH), 110.1 (CH), 67.4 (CH_2), 63.2 (CH), 61.6 (CH), 37.2 (CH), 13.5 (CH_3)
^{19}F NMR (282 MHz, Acetone-d$_6$)	**δ :** -115.45
IR (Neat, cm^{-1})	3163, 2964, 2934, 1711, 1624, 1605, 1588, 1572, 1534, 1489, 1456, 1428, 1405, 1385, 1366, 1345, 1326, 1277, 1246, 1214, 1171, 1144, 1120, 1072, 1029, 1009
HRMS (ESI+, m/z)	$[M+H]^+$ calcd. for $C_{26}H_{22}BrF_3N_3O_2S$ 576.0568, found 576.0550
HPLC Analysis	Daicel Chiralpak IA, Heptane/*i*PrOH = 90/10, flow rate = 1 mL/min, 254 nm<br>minor isomer: t_R = 10.01 min, major isomer: t_R = 17.51 min
Enantiomeric excess	99%
$[\alpha]_D^{22}$	-5.93° (*c* 4.93, $CHCl_3$)

benzyl ((2*R*,3*S*,4*R*)-2-(4-bromophenyl)-3-methyl-8-(trifluoromethyl)-3,4-dihydro-2*H*-benzo[4,5]thiazolo[3,2-*a*]pyrimidin-4-yl)carbamate

4c

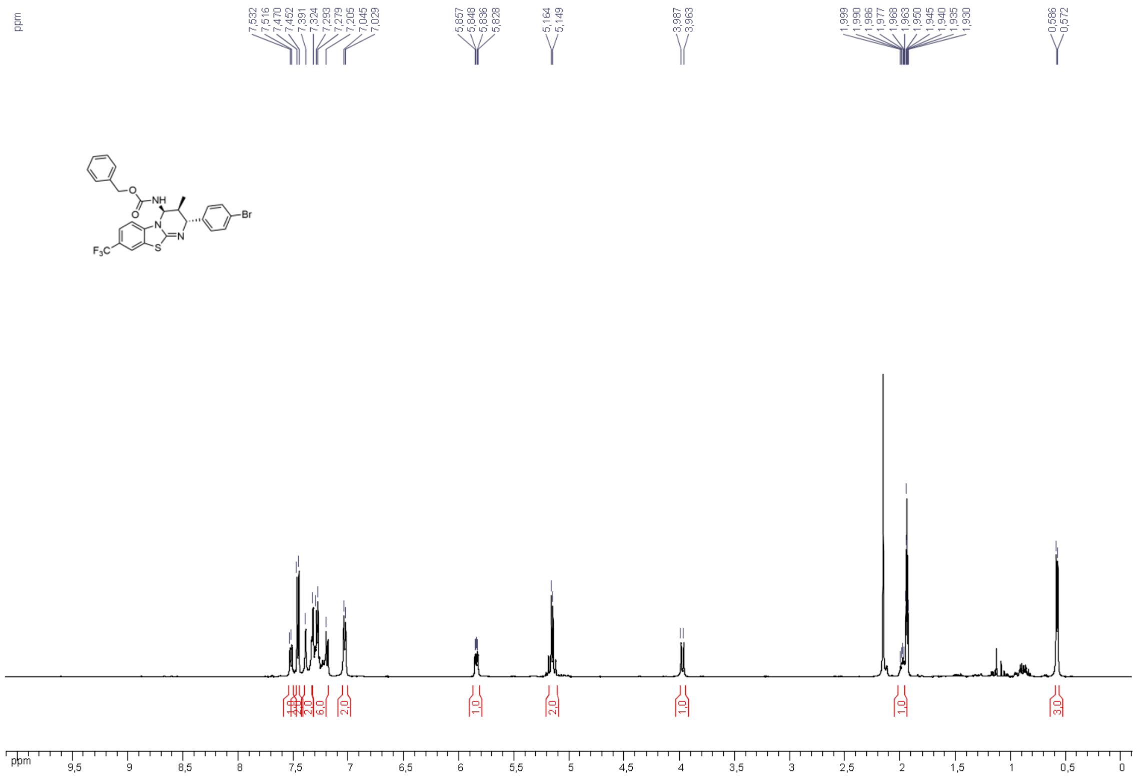

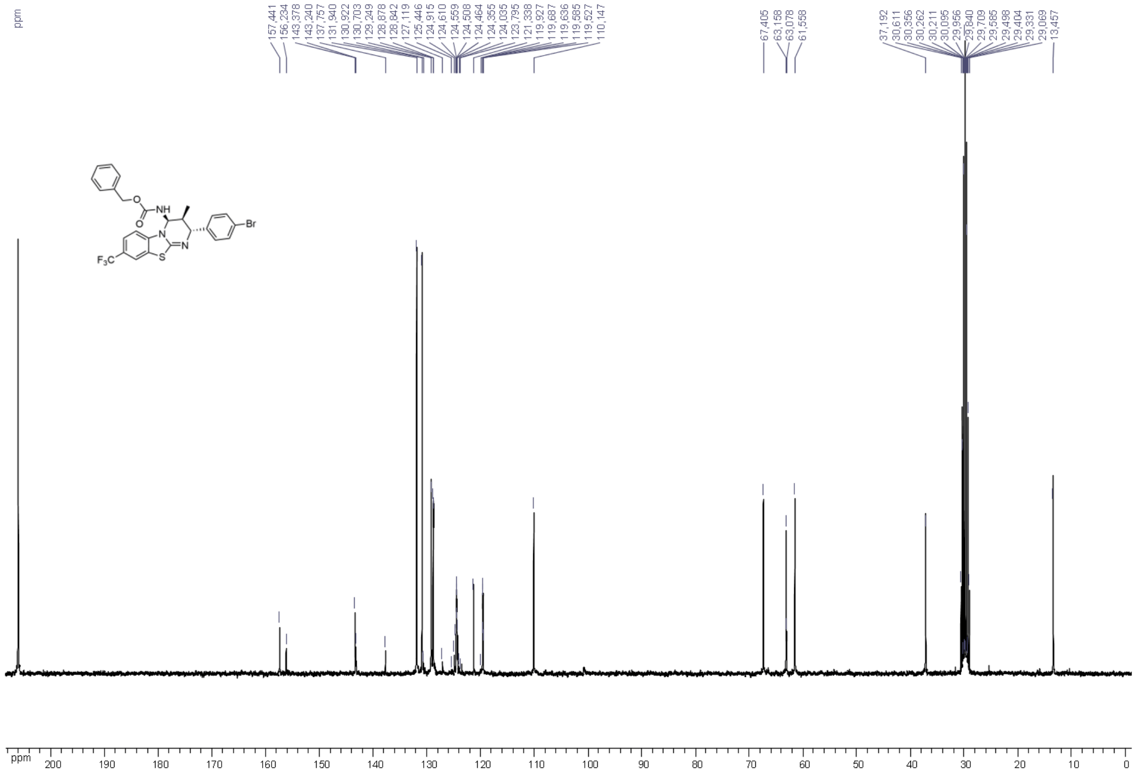

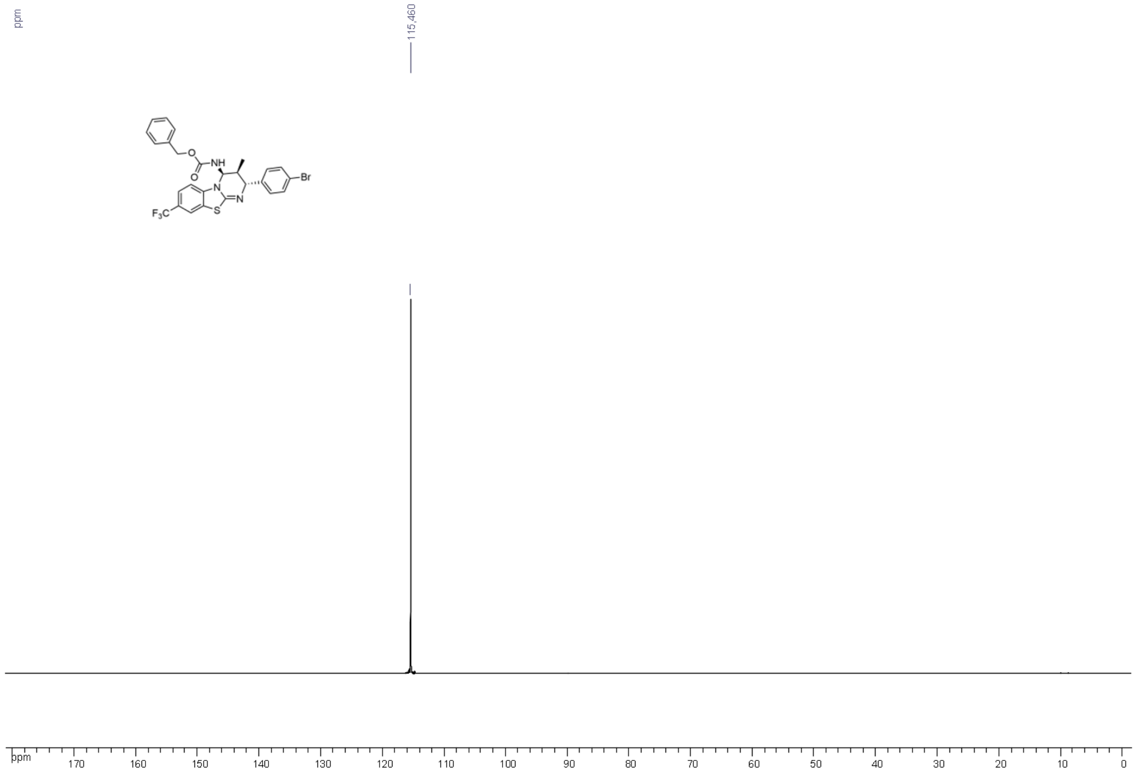

ppm
115.460
ppm
170
160
150
140
130
120
110
100
90
80
70
60
50
40
30
20
10
0

benzyl ((2*R*,3*S*,4*R*)-2-(4-bromophenyl)-3-methyl-8-(trifluoromethyl)-3,4-dihydro-2*H*-benzo[4,5]thiazolo[3,2-*a*]pyrimidin-4-yl)carbamate

4c

Chiralpak IA, Heptane/*i*PrOH = 90/10, 1 mL/min, 254 nm

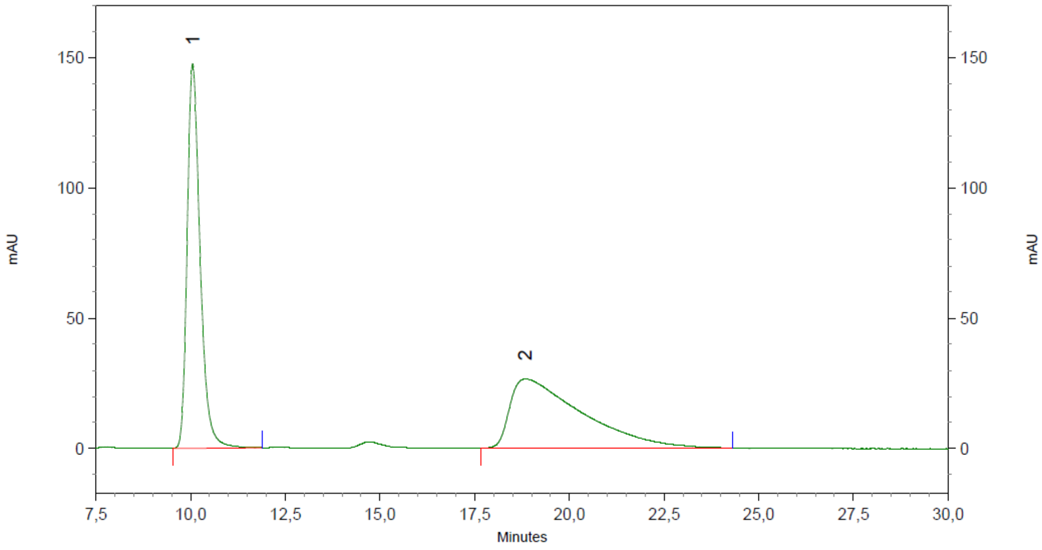

DAD-CH1 254 nm

Results

Pk #	Retention Time	Area	Area %
1	10,05	14007176	49,99
2	18,83	14015522	50,01

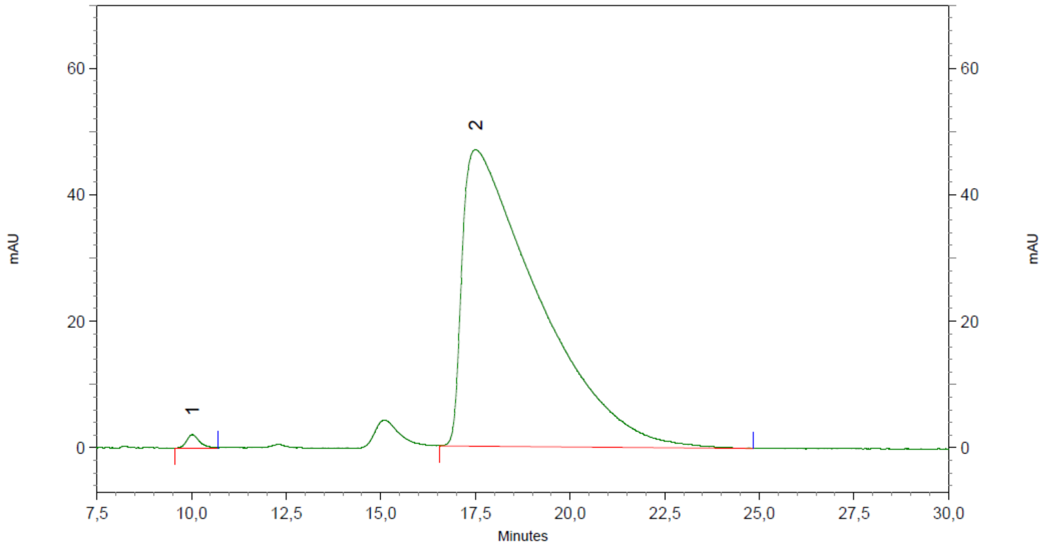

DAD-CH1 254 nm

Results

Pk #	Retention Time	Area	Area %
1	10,01	196977	0,75
2	17,51	26181346	99,25

benzyl ((2*R*,3*S*,4*R*)-2-(4-bromophenyl)-8-fluoro-3-methyl-3,4-dihydro-2*H*-benzo[4,5]thiazolo[3,2-*a*]pyrimidin-4-yl)carbamate

4d

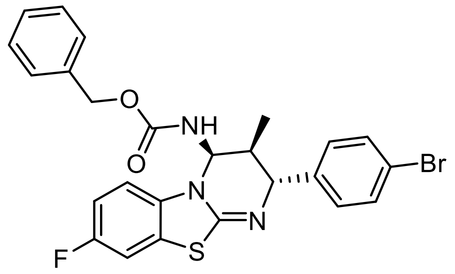

Compound **4d** was prepared according to the general procedure **D** from 2-benzothiazolimine **2d** (33.5 mg, 0.1 mmol) and enecarbamate **3a** (57.4 mg, 0.3 mmol) as starting materials. Purification on a column of silica gel with a gradient of ethyl acetate in heptane (from 85:15 to 8:2) as eluent gave the desired product as only one diastereomer.

Chemical formula	$C_{25}H_{21}BrFN_3O_2S$
M (g.mol^{-1})	526.42
Yield	51.5 mg, 98%
Aspect	Colorless oil
Rf	0.6 (Hept/AcOEt : 7/3)
^{1}H NMR (300 MHz, Acetone-d$_6$)	**δ :** 7.83 (d, *J* = 10.6 Hz, 1H, NH), 7.48 (d, *J* = 8.4 Hz, 2H), 7.39-7.30 (m, 5H), 7.16 (d, *J* = 8.6 Hz, 2H), 7.15 (dd, *J* = 8.3, 4.6 Hz, 1H), 7.13 (dd, *J* = 8.3, 2.7 Hz, 1H), 7.01 (td, *J* = 8.6, 2.7 Hz, 1H), 5.86 (dd, *J* = 10.1, 4.4 Hz, 1H), 5.02 (dd, *J* = 14.5, 12.5 Hz, 2H), 4.16 (d, *J* = 11.3 Hz, 1H), 2.08-2.01 (m, 1H), 0.67 (d, *J* = 6.4 Hz, 3H)
^{13}C NMR (75 MHz, Acetone-d$_6$)	**δ :** 159.35 (d, *J* = 238.0 Hz, CF), 157.4 (C), 156.7 (C), 143.7 (C), 137.9 (C), 136.9 (C), 131.8 (2 CH), 131.0 (2 CH), 129.3 (2 CH), 128.8 (CH), 128.7 (2 CH), 124.7 (d, *J* = 10.9 Hz, C), 121.2 (C), 113.4 (d, *J* = 23.2 Hz, CH), 110.7 (d, *J* = 7.7 Hz, CH), 110.0 (d, *J* = 27.1 Hz, CH), 67.2 (CH_2), 63.1 (CH), 61.4 (CH), 37.2 (CH), 13.5 (CH_3)
^{19}F NMR (282 MHz, Acetone-d$_6$)	**δ :** -55.22
IR (Neat, cm^{-1})	3159, 2962, 2934, 1709, 1619, 1590, 1572, 1538, 1481, 1456, 1429, 1405, 1384, 1341, 1317, 1291, 1255, 1230, 1187, 1125, 1103, 1072, 1029, 1009
HRMS (ESI+, m/z)	$[M+H]^+$ calcd. for $C_{25}H_{22}BrFN_3O_2S$ 526.0600, found 526.0574
HPLC Analysis	Daicel Chiralpak IA, Heptane/EtOH = 90/10, flow rate = 1 mL/min, 254 nm<br>major isomer: t_R = 11.77 min, minor isomer: t_R = 15.42 min
Enantiomeric excess	98%
$[\alpha]_D^{22}$	-6.71° (*c* 4.74, $CHCl_3$)

benzyl ((2*R*,3*S*,4*R*)-2-(4-bromophenyl)-8-fluoro-3-methyl-3,4-dihydro-2*H*-benzo[4,5]thiazolo[3,2-*a*]pyrimidin-4-yl)carbamate

4d

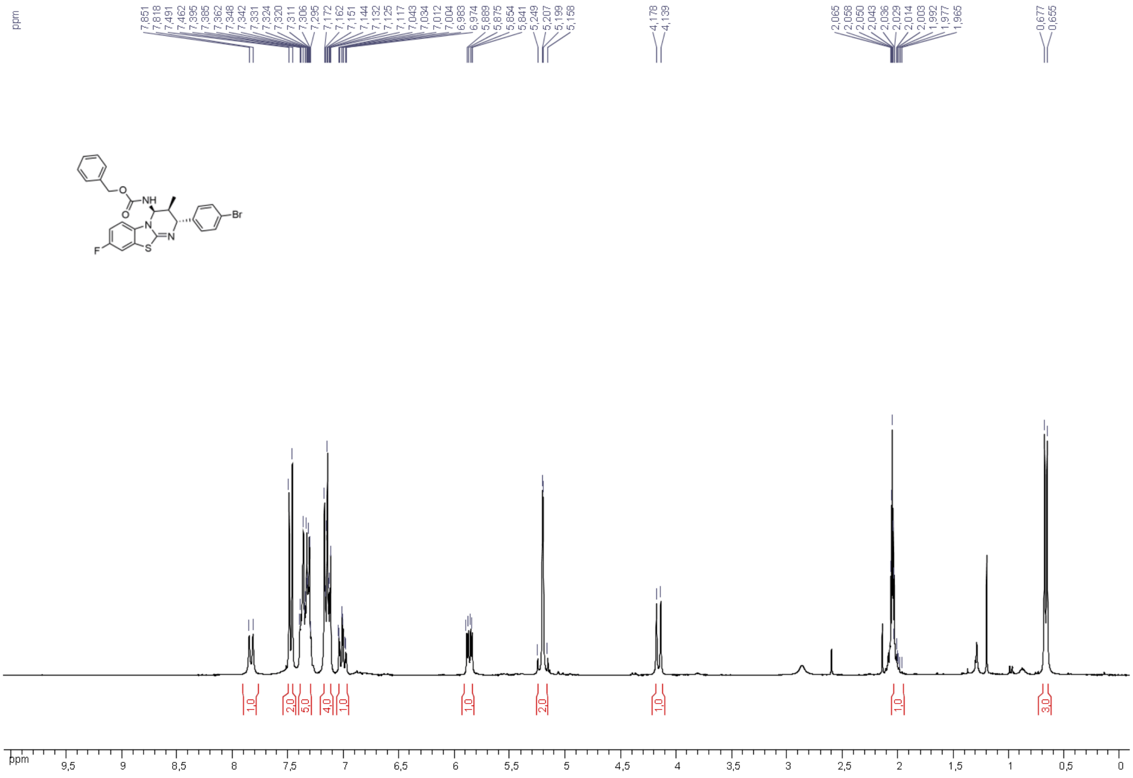

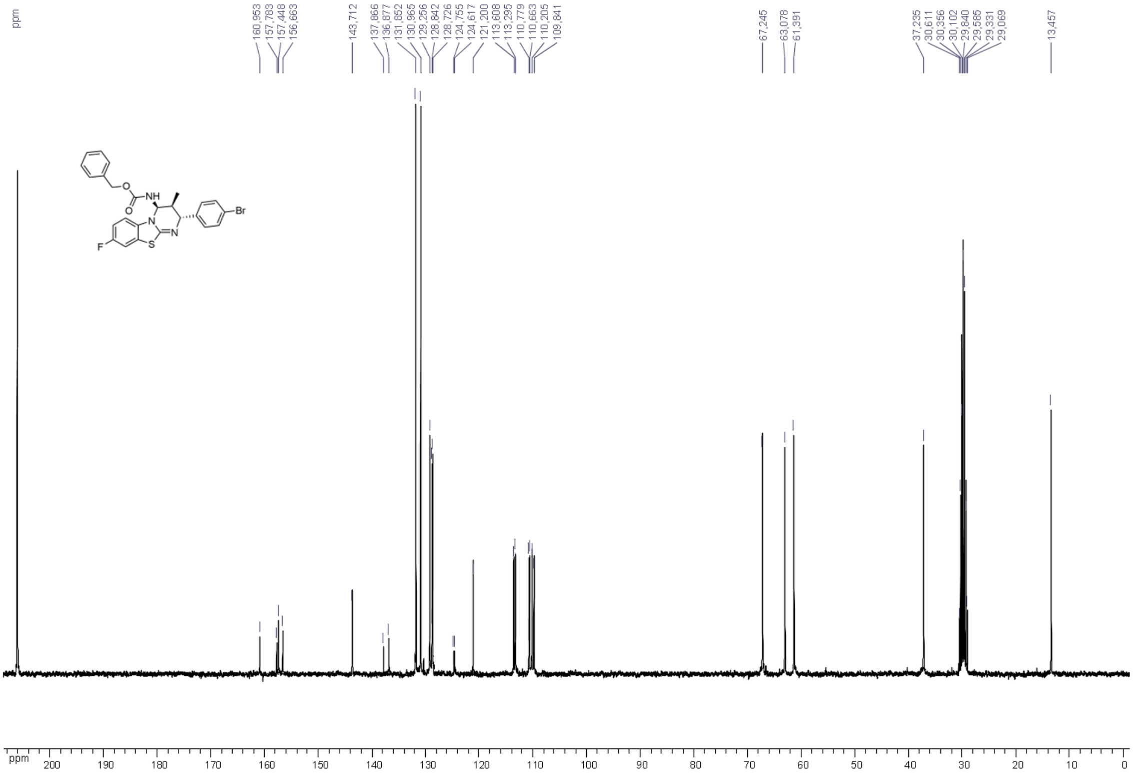

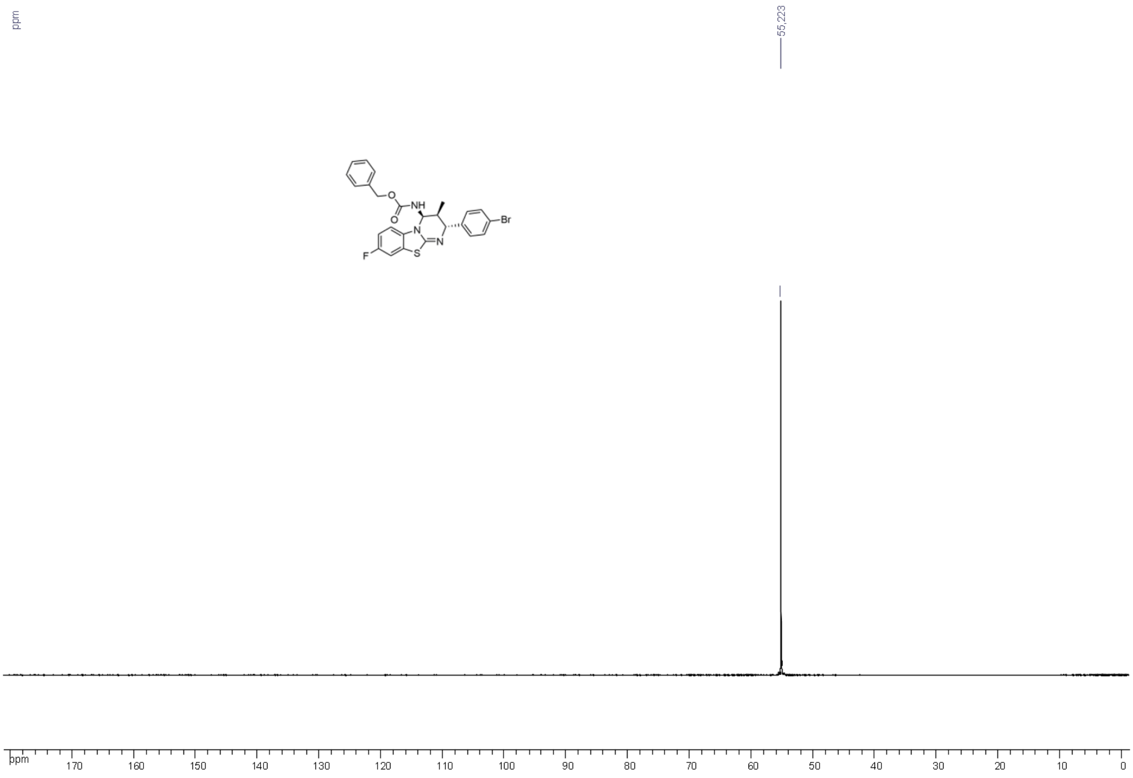
ppm
55.223
NH
O
N
N
S
F
Br
ppm
170
160
150
140
130
120
110
100
90
80
70
60
50
40
30
20
10
0

benzyl ((2*R*,3*S*,4*R*)-2-(4-bromophenyl)-8-fluoro-3-methyl-3,4-dihydro-2*H*-benzo[4,5]thiazolo[3,2-*a*]pyrimidin-4-yl)carbamate
4d

Chiralpak IA, Heptane/EtOH = 90/10, 1 mL/min, 254 nm

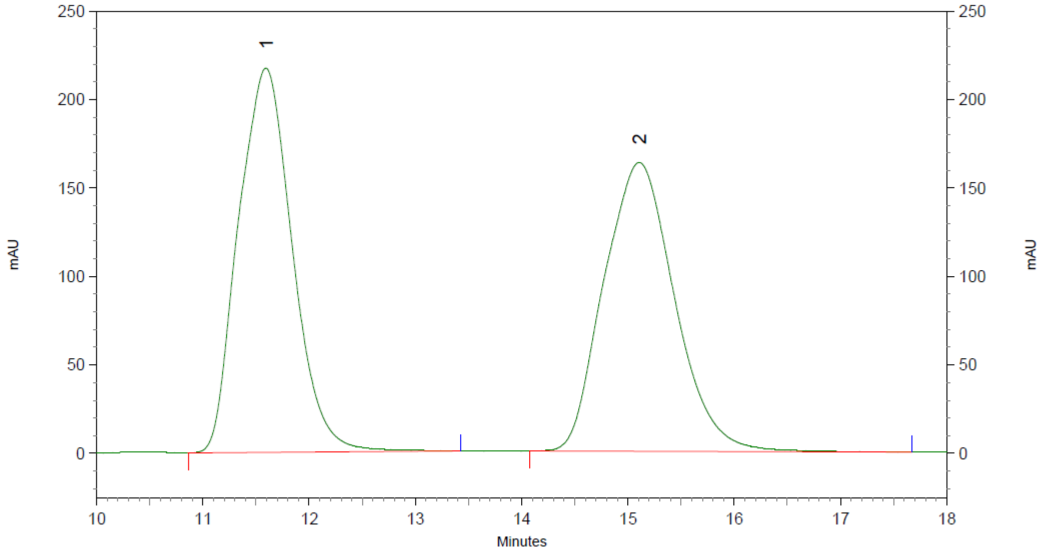

DAD-CH1 250 nm
Results

Pk #	Retention Time	Area	Area %
1	11,59	30645212	50,36
2	15,11	30202702	49,64

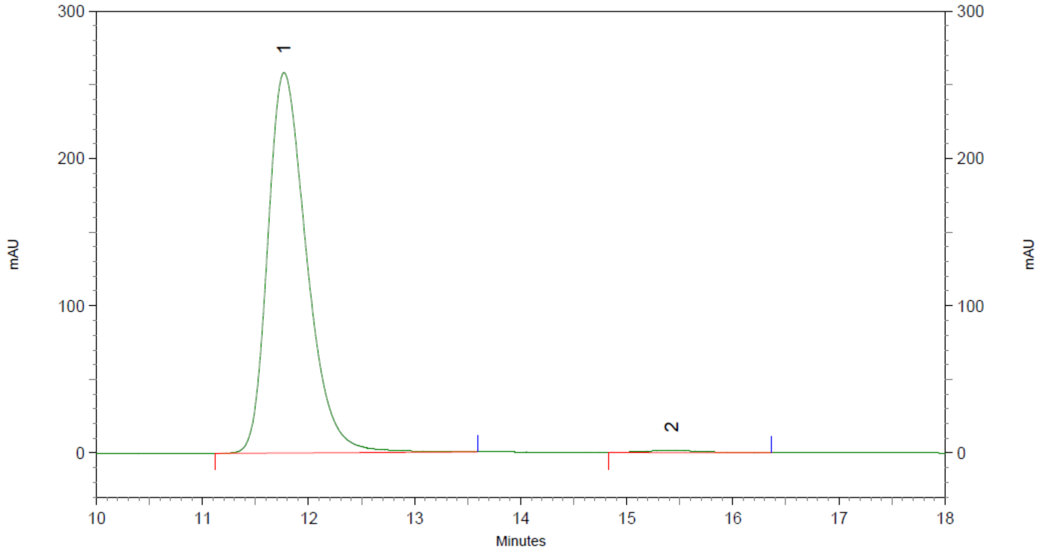

DAD-CH1 254 nm
Results

Pk #	Retention Time	Area	Area %
1	11,77	26313683	99,23
2	15,42	205394	0,77

benzyl ((2*R*,3*S*,4*R*)-8-bromo-2-(4-bromophenyl)-3-methyl-3,4-dihydro-2*H*-benzo[4,5]thiazolo[3,2-*a*]pyrimidin-4-yl)carbamate

4e

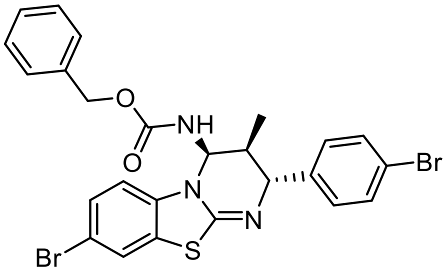

Compound **4e** was prepared according to the general procedure **D** from 2-benzothiazolimine **2e** (33.5 mg, 0.1 mmol) and enecarbamate **3a** (42.3 mg, 0.3 mmol) as starting materials. Purification on a column of silica gel with a gradient of ethyl acetate in heptane (from 85:15 to 8:2) as eluent gave the desired product as only one diastereomer.

Chemical formula	$C_{25}H_{21}Br_2N_3O_2S$
M (g.mol^{-1})	587.33
Yield	51.7 mg, 88%
Aspect	Colorless oil
Rf	0.7 (Hept/AcOEt : 7/3)
^{1}H NMR (300 MHz, CD_3CN)	**δ :** 7.46 (d, *J* = 8.3 Hz, 2H), 7.36 (d, *J* = 2.1 Hz, 1H), 7.34-7.20 (m, 6H), 7.08 (d, *J* = 8.3 Hz, 2H), 7.00 (d, *J* = 10.3 Hz, 1H, NH), 6.97 (d, *J* = 8.4 Hz, 1H), 5.76 (dd, *J* = 10.3, 4.2 Hz, 1H), 5.15 (dd, *J* = 15.9, 12.2 Hz, 2H), 4.00 (d, *J* = 12.0 Hz, 1H), 2.00-1.96 (m, 1H), 0.60 (d, *J* = 6.8 Hz, 3H)
^{13}C NMR (75 MHz, CD_3CN)	**δ :** 157.4 (C), 156.6 (C), 143.5 (C), 139.6 (C), 137.9 (C), 132.1 (2 CH), 130.9 (2 CH), 130.0 (CH), 129.5 (2 CH), 129.1 (CH), 128.8 (2 CH), 125.4 (C), 125.4 (CH), 121.4 (C), 114.8 (C), 111.5 (CH), 67.6 (CH_2), 63.0 (CH), 61.6 (CH), 37.0 (CH), 13.4 (CH_3)
IR (Neat, cm^{-1})	2963, 1716, 1623, 1588, 1571, 1538, 1488, 1468, 1405, 1317, 1247, 1204, 1071, 1010
HRMS (ESI+, m/z)	$[M+H]^+$ calcd. for $C_{25}H_{22}Br_2N_3O_2S$ 585.9799, found 585.9808
HPLC Analysis	Daicel Chiralpak AD-H, Heptane/*i*PrOH = 90/10, flow rate = 1 mL/min, 220 nm major isomer: t_R = 9.12 min, minor isomer: t_R = 19.29 min
Enantiomeric excess	99%
$[\alpha]_D^{22}$	-93.67° (*c* 0.3, $CHCl_3$)

benzyl ((2*R*,3*S*,4*R*)-8-bromo-2-(4-bromophenyl)-3-methyl-3,4-dihydro-2*H*-benzo[4,5]thiazolo[3,2-*a*]pyrimidin-4-yl)carbamate

4e

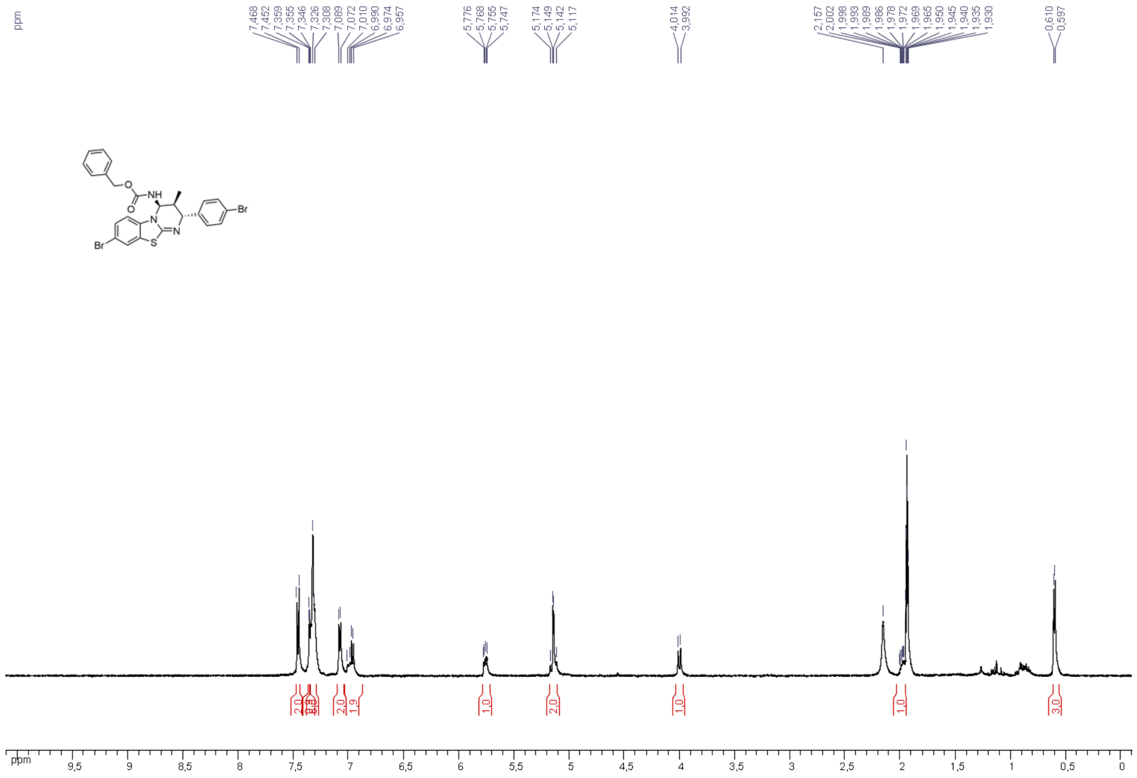

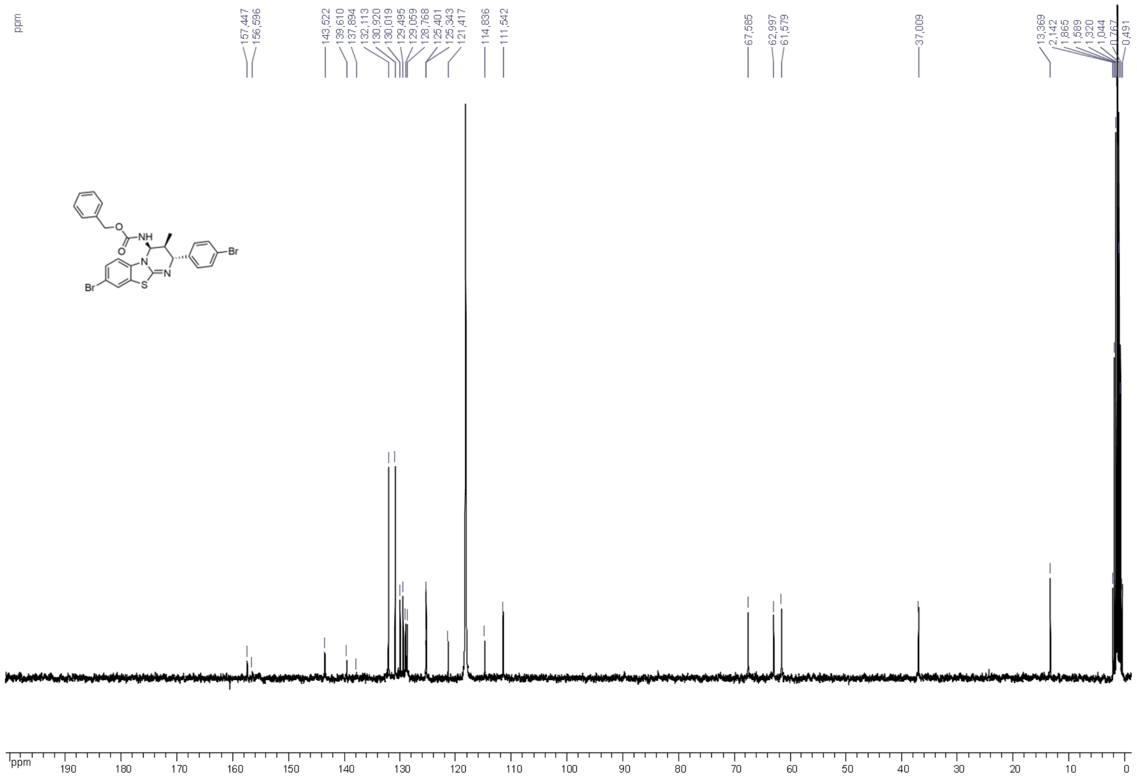

benzyl ((2*R*,3*S*,4*R*)-8-bromo-2-(4-bromophenyl)-3-methyl-3,4-dihydro-2*H*-benzo[4,5]thiazolo[3,2-*a*]pyrimidin-4-yl)carbamate
4e

Chiralpak AD-H, Heptane/*i*PrOH = 90/10, 1 mL/min, 220 nm

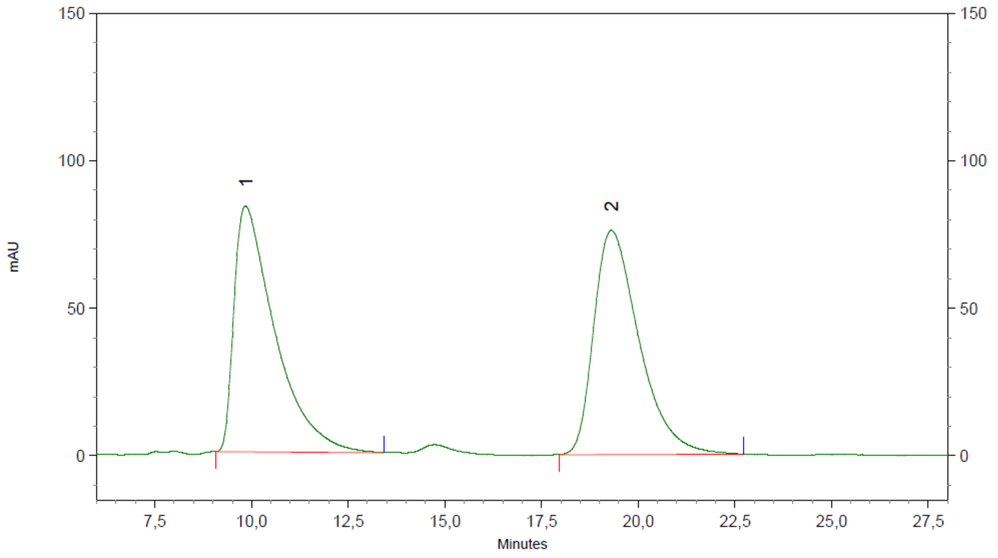

DAD-CH4 220 nm
Results

Pk #	Retention Time	Area	Area %
1	9,85	24483156	49,74
2	19,31	24737131	50,26

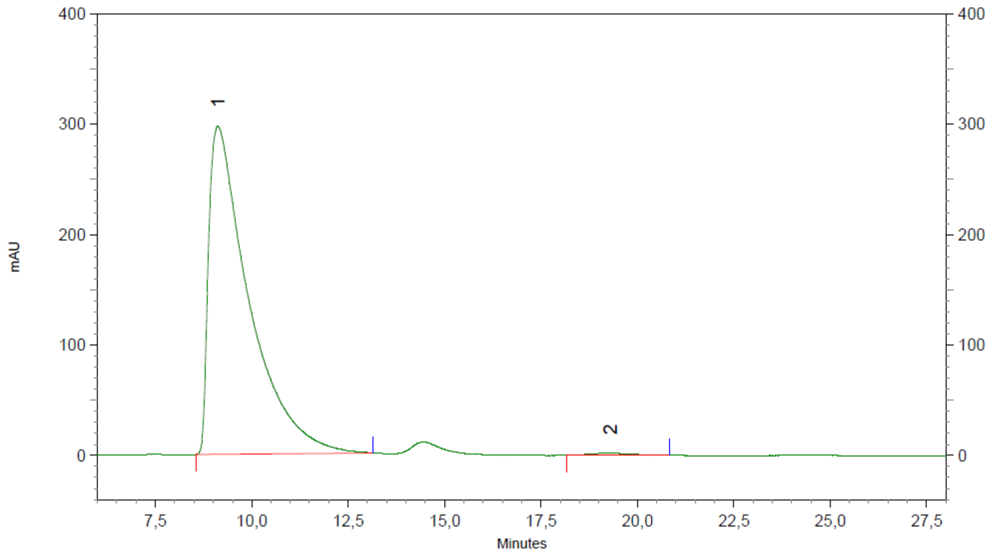

DAD-CH4 220 nm
Results

Pk #	Retention Time	Area	Area %
1	9,12	85964841	99,31
2	19,29	593042	0,69

benzyl ((2*R*,3*S*,4*R*)-2-(4-bromophenyl)-8-chloro-3-methyl-3,4-dihydro-2*H*-benzo[4,5]thiazolo[3,2-*a*]pyrimidin-4-yl)carbamate

4f

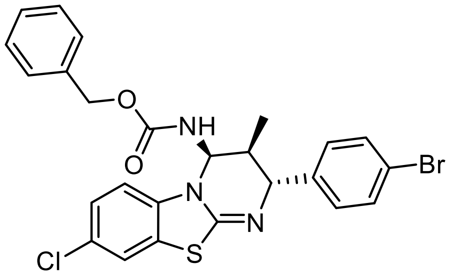

Compound **4f** was prepared according to the general procedure **D** from 2-benzothiazolimine **2f** (35.2 mg, 0.1 mmol) and enecarbamate **3a** (57.4 mg, 0.3 mmol)as starting materials. Purification on a column of silica gel with a gradient of ethyl acetate in heptane (from 9:1 to 85:15) as eluent gave the desired product as only one diastereomer.

Chemical formula	$C_{25}H_{21}BrClN_3O_2S$
M (g.mol^{-1})	542.88
Yield	42.0 mg, 77%
Aspect	White foam
Rf	0.7 (Hept/AcOEt : 7/3)
^{1}H NMR (300 MHz, Acetone-d$_6$)	**δ :** 7.70 (d, *J* = 10.1 Hz, 1H, NH), 7.49 (d, *J* = 8.5 Hz, 2H), 7.39-7.30 (m, 5H), 7.35 (d, *J* = 2.2 Hz, 1H), 7.24 (dd, *J* = 8.5, 2.0 Hz, 1H), 7.18 (d, *J* = 8.5 Hz, 2H), 7.13 (d, *J* = 8.7 Hz, 1H), 5.88 (dd, *J* = 10.2, 4.3 Hz, 1H), 5.19 (s, 2H), 4.21 (d, *J* = 11.5 Hz, 1H), 2.14-2.10 (m, 1H), 0.71 (d, *J* = 6.9 Hz, 3H)
^{13}C NMR (75 MHz, Acetone-d$_6$)	**δ :** 157.4 (C), 156.2 (C), 143.7 (C), 139.3 (C), 137.8 (C), 131.9 (2 CH), 130.9 (2 CH), 129.3 (2 CH), 128.8 (CH), 128.7 (2 CH), 127.6 (C), 126.9 (CH), 125.1 (C), 122.3 (CH), 121.2 (C), 111.1 (CH), 67.3 (CH_2), 63.1 (CH), 61.4 (CH), 37.1 (CH), 13.5 (CH_3)
IR (Neat, cm^{-1})	2961, 1716, 1624, 1572, 1539, 1488, 1471, 1406, 1317, 1247, 1215, 1072, 1009
HRMS (ESI+, m/z)	$[M+H]^+$ calcd. for $C_{25}H_{22}BrClN_3O_2S$ 542.0305, found 542.0309
HPLC Analysis	Daicel Chiralpak AD-H, Heptane/*i*PrOH = 90/10, flow rate = 1 mL/min, 254 nm<br>major isomer: t_R = 7.93 min, minor isomer: t_R = 17.67 min
Enantiomeric excess	98%
$[\alpha]_D^{22}$	-123.00° (*c* 0.3, $CHCl_3$)

benzyl ((2*R*,3*S*,4*R*)-2-(4-bromophenyl)-8-chloro-3-methyl-3,4-dihydro-2*H*-benzo[4,5]thiazolo[3,2-*a*]pyrimidin-4-yl)carbamate

4f

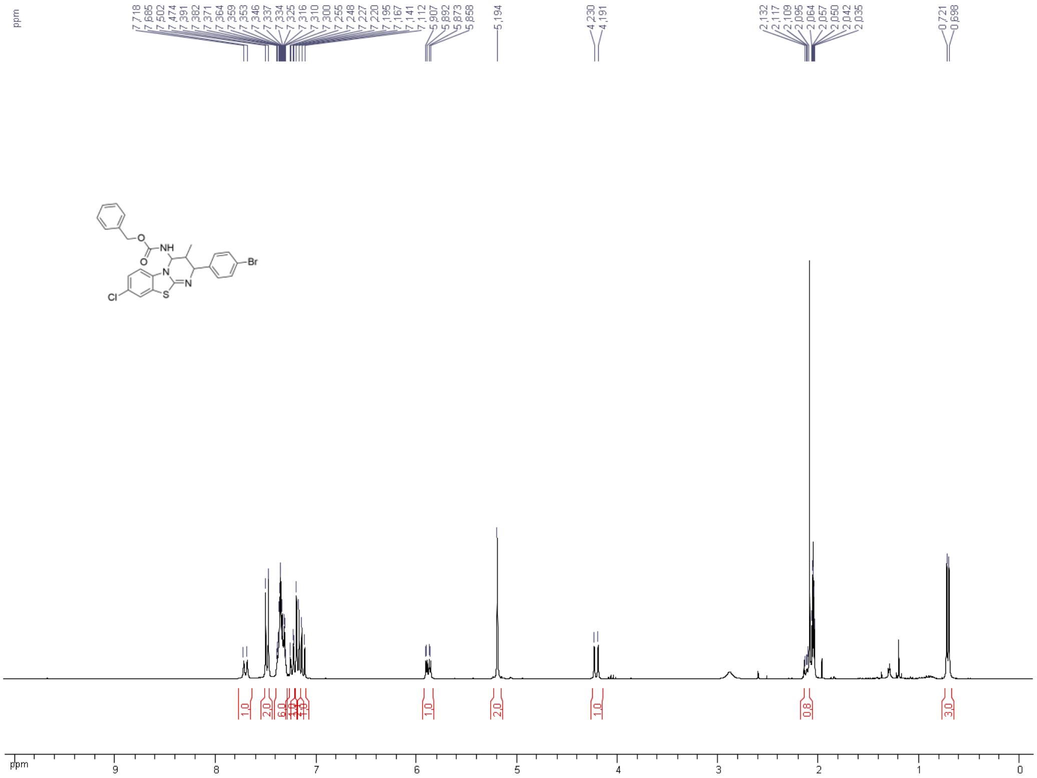

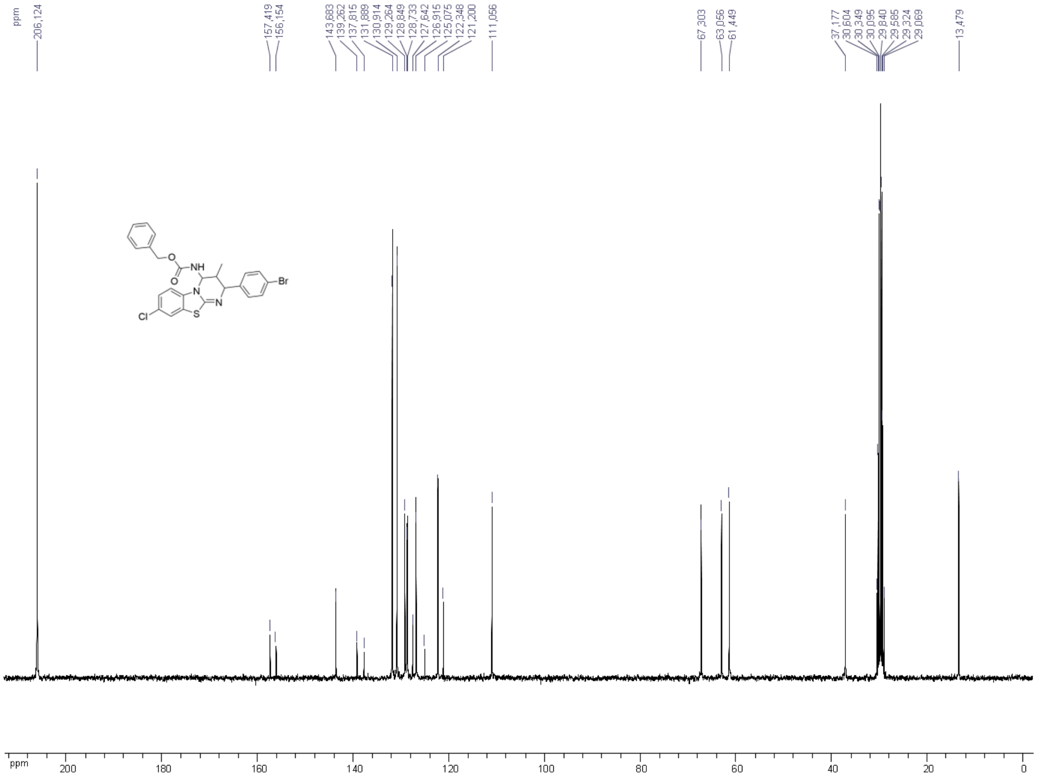

benzyl ((2*R*,3*S*,4*R*)-2-(4-bromophenyl)-8-chloro-3-methyl-3,4-dihydro-2*H*-benzo[4,5]thiazolo[3,2-*a*]pyrimidin-4-yl)carbamate
4f

Chiralpak AD-H, Heptane/*i*PrOH = 90/10, 1 mL/min, 254 nm

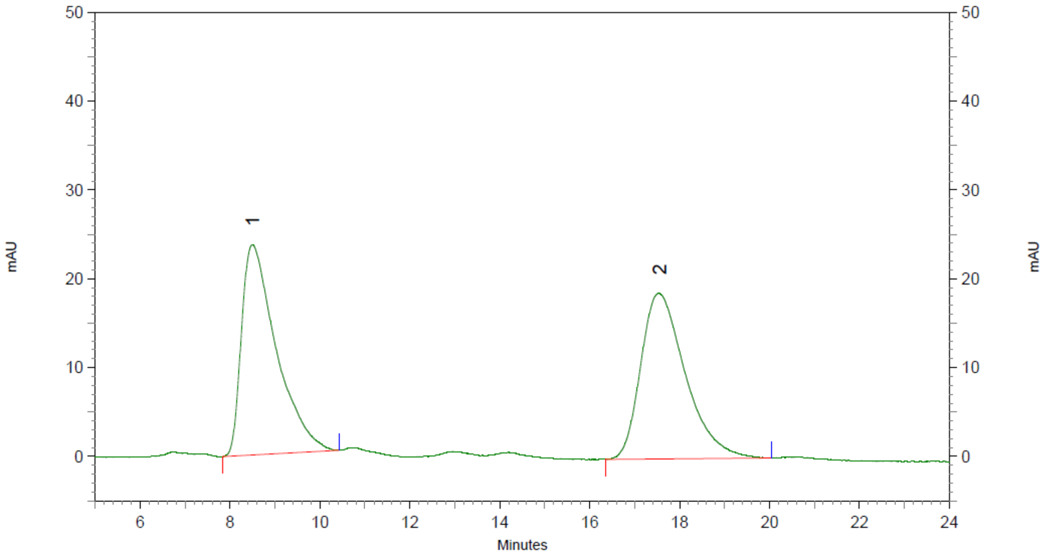

DAD-CH1 254 nm
Results

Pk #	Retention Time	Area	Area %
1	8,49	5081469	49,73
2	17,55	5137107	50,27

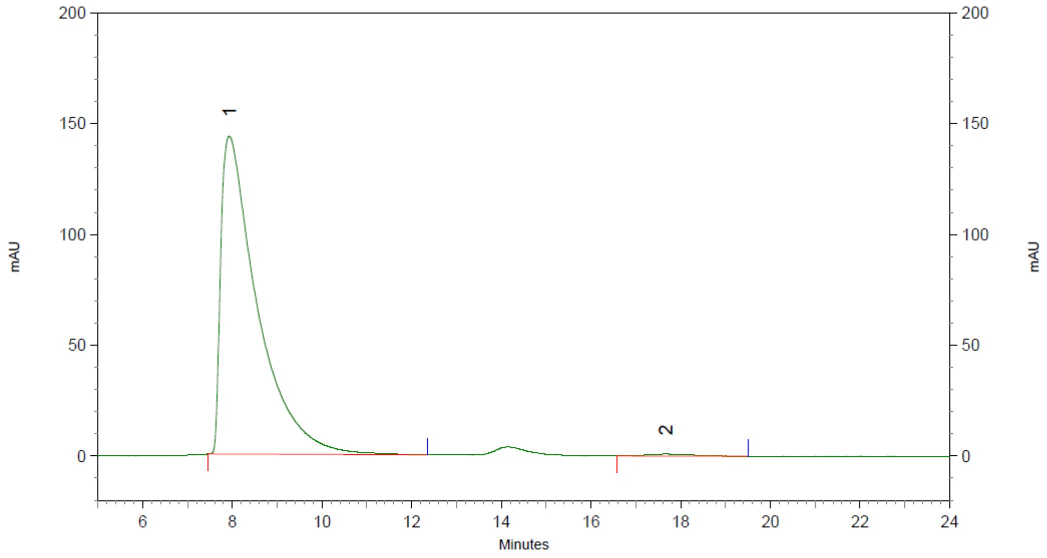

DAD-CH1 254 nm
Results

Pk #	Retention Time	Area	Area %
1	7,93	32363796	99,21
2	17,67	258178	0,79

benzyl ((2*R*,3*S*,4*R*)-3-methyl-2-phenyl-3,4-dihydro-2*H*-benzo[4,5]thiazolo[3,2-*a*]pyrimidin-4-yl)carbamate

4g

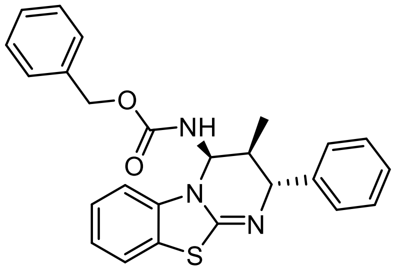

Compound **4g** was prepared according to the general procedure **D** from 2-benzothiazolimine **2g** (23.8 mg, 0.1 mmol) and enecarbamate **3a** (57.4 mg, 0.3 mmol) as starting materials. Purification on a column of silica gel with a gradient of ethyl acetate in heptane (from 9:1) as eluent gave the desired product as only one diastereomer.

Chemical formula	$C_{25}H_{23}N_3O_2S$
M (g.mol^{-1})	429.54
Yield	41.7 mg, 97%
Aspect	White foam
Rf	0.3 (Hept/AcOEt : 7/3)
^{1}H NMR (300 MHz, CD_3CN)	**δ :** 7.67 (d, *J* = 11.7 Hz, 1H, NH), 7.35-7.26 (m, 8H), 7.21 (ddd, *J* = 8.8, 5.3, 2.4 Hz, 1H), 7.11-7.10 (m, 3H), 7.04 (dd, *J* = 7.1, 0.8 Hz, 1H), 7.02 (td, *J* = 7.4, 1.3 Hz, 1H), 5.78 (dd, *J* = 10.3, 5.1 Hz, 1H), 5.19 (d, *J* = 12.5 Hz, 1H), 5.14 (d, *J* = 12.5 Hz, 1H), 3.97 (d, *J* = 11.9 Hz, 1H), 1.91-1.85 (m, 1H), 0.48 (d, *J* = 7.1 Hz, 3H)
^{13}C NMR (75 MHz, CD_3CN)	**δ :** 157.6 (C), 157.1 (C), 144.2 (C), 140.4 (C), 138.1 (C), 129.4 (CH), 129.1 (2 CH), 128.9 (4 CH), 128.6 (2 CH), 128.0 (CH), 127.1 (CH), 123.3 (C), 123.2 (CH), 122.6 (CH), 110.2 (CH), 67.3 (CH_2), 62.9 (CH), 62.1 (CH), 37.4 (CH), 13.4 (CH_3)
IR (Neat, cm^{-1})	3160, 2962, 1713, 1622, 1588, 1543, 1498, 1472, 1455, 1340, 1323, 1304, 1250, 1216, 1083, 1064, 1027
HRMS (ESI+, m/z)	$[M+H]^+$ calcd. for $C_{25}H_{24}N_3O_2S$ 430.1589, found 430.1591
HPLC Analysis	Daicel Chiralpak IA, Heptane/EtOH = 90/10, flow rate = 1 mL/min, 254 nm<br>major isomer: t_R = 11.91 min, minor isomer: t_R = 33.79 min
Enantiomeric excess	99%
$[\alpha]_D^{22}$	-130.33° (*c* 0.3, $CHCl_3$)

benzyl ((2*R*,3*S*,4*R*)-3-methyl-2-phenyl-3,4-dihydro-2*H*-benzo[4,5]thiazolo[3,2-*a*]pyrimidin-4-yl)carbamate

4g

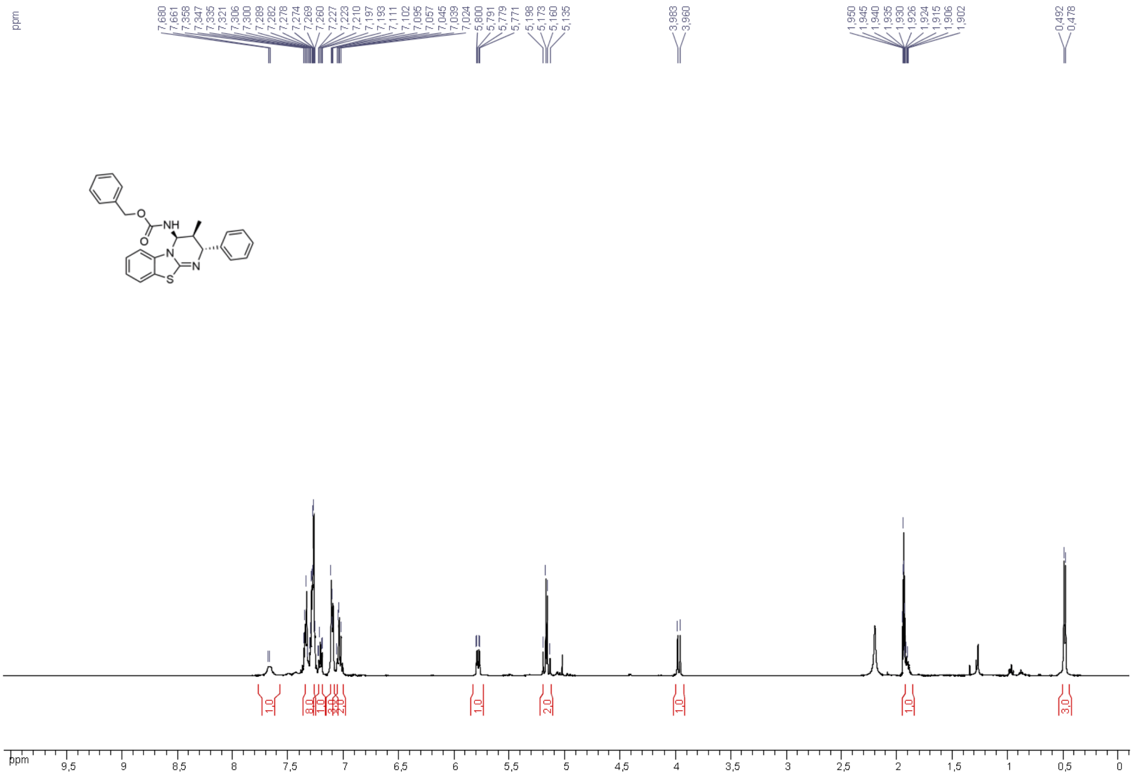

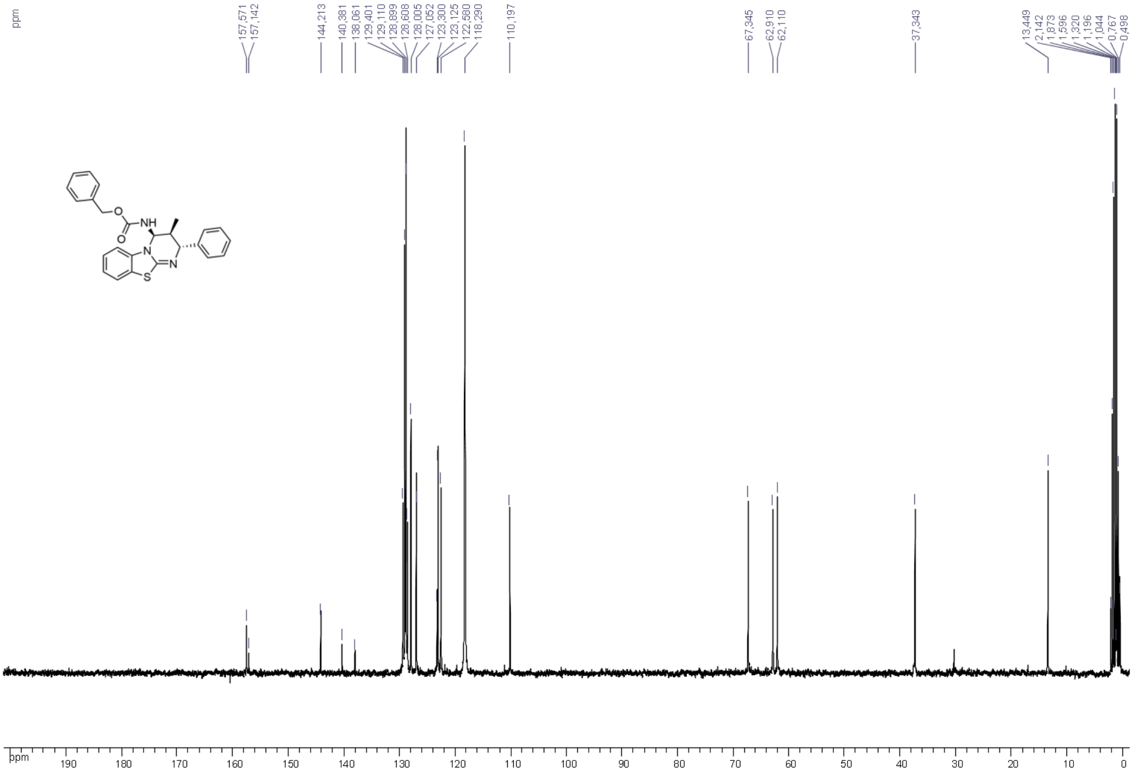

benzyl ((2*R*,3*S*,4*R*)-3-methyl-2-phenyl-3,4-dihydro-2*H*-benzo[4,5]thiazolo[3,2-*a*]pyrimidin-4-yl)carbamate
4g

Chiralpak IA, Heptane/EtOH = 90/10, 1 mL/min, 254 nm

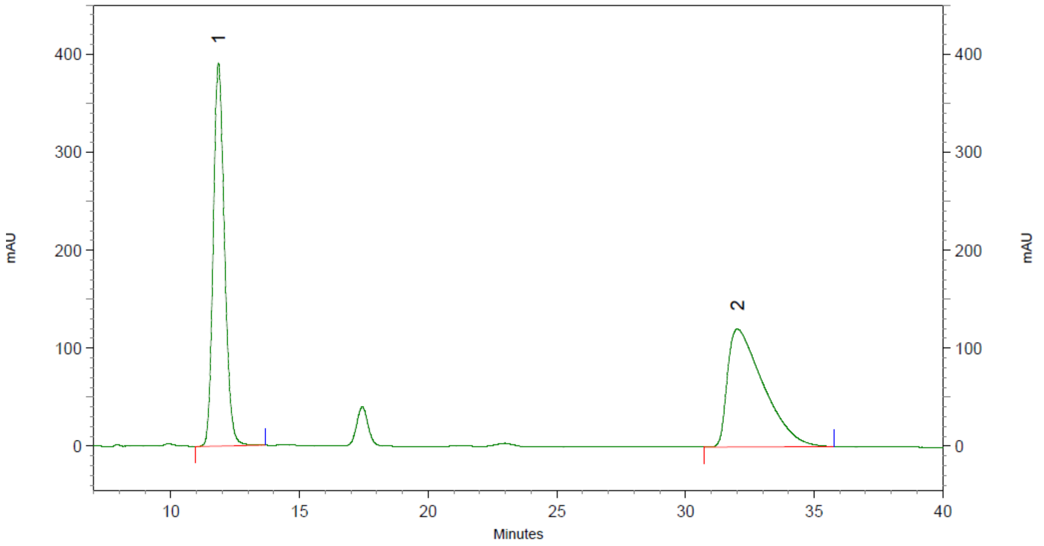

DAD-CH1 254 nm
Results

Pk #	Retention Time	Area	Area %
1	11,85	47547994	50,50
2	32,01	46603558	49,50

0.1 mmol scale:

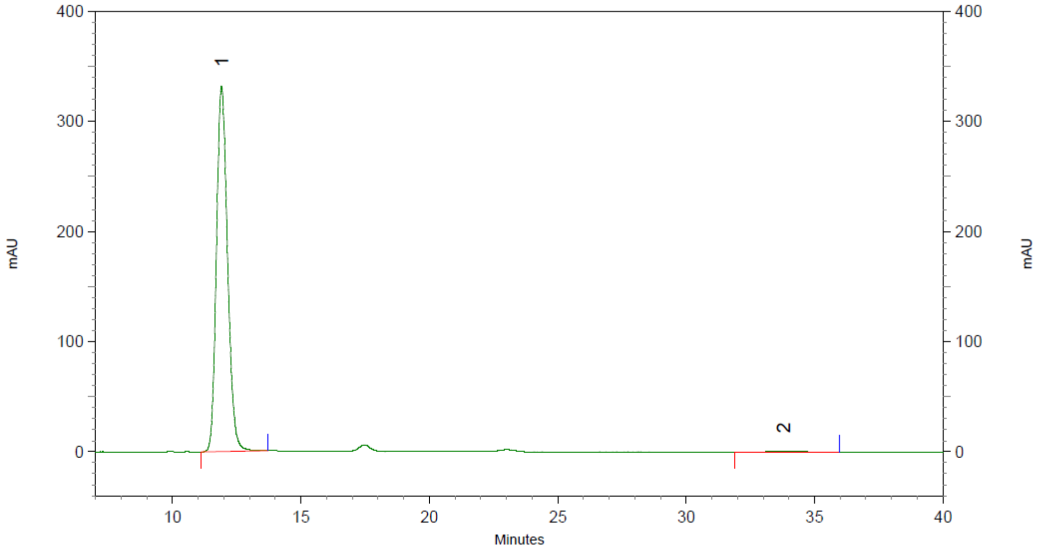

DAD-CH1 254 nm
Results

Pk #	Retention Time	Area	Area %
1	11,91	39918057	99,52
2	33,79	193841	0,48

1.0 mmol scale:

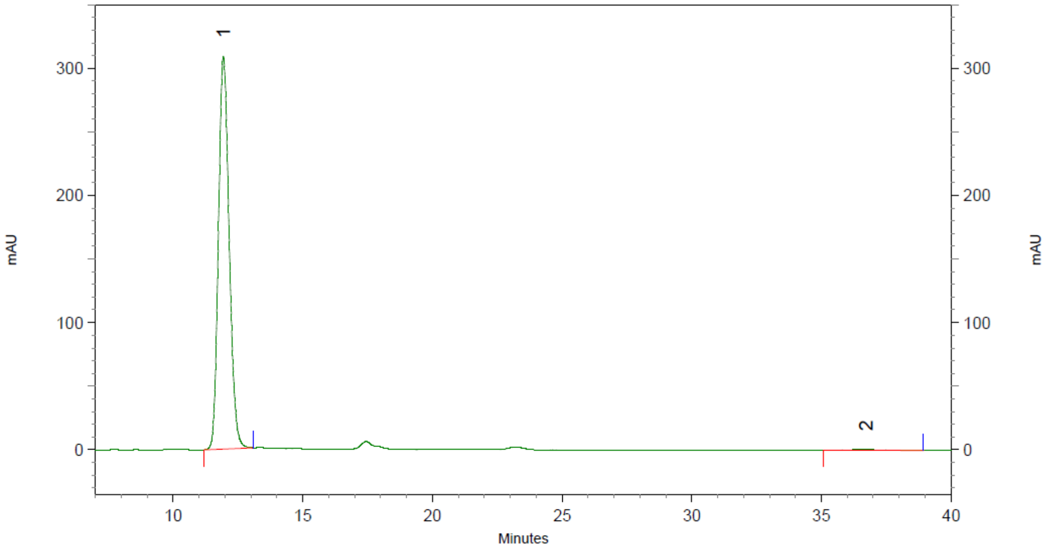

DAD-CH1 254 nm

Results

Pk #	Retention Time	Area	Area %
1	11,93	35525193	99,45
2	36,71	196886	0,55

benzyl ((2*R*,3*S*,4*R*)-8-methoxy-2-(4-methoxyphenyl)-3-methyl-3,4-dihydro-2*H*-benzo[4,5]thiazolo[3,2-*a*]pyrimidin-4-yl)carbamate

4h

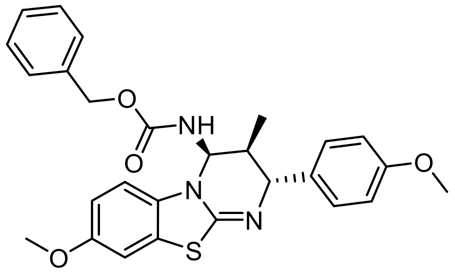

Compound **4h** was prepared according to the general procedure **D** from 2-benzothiazolimine **2h** (29.8 mg, 0.1 mmol) and enecarbamate **3a** (57.4 mg, 0.3 mmol) as starting materials. Purification on a column of silica gel with a gradient of ethyl acetate in heptane (from 9:1 to 8:2) as eluent gave the desired product as only one diastereomer.

Chemical formula	$C_{27}H_{27}N_3O_4S$
M (g.mol^{-1})	489.59
Yield	35.0 mg, 71%
Aspect	White foam
Rf	0.1 (Hept/AcOEt : 7/3)
^{1}H NMR (300 MHz, CD_3CN)	**δ :** 7.36-7.33 (m, 5H), 7.18 (d, *J* = 8.5 Hz, 2H), 7.01 (s, 1H), 6.92 (d, *J* = 9.2 Hz, 1H), 6.89 (d, *J* = 8.6 Hz, 2H), 6.77 (dd, *J* = 9.1, 2.2 Hz, 1H), 6.40 (d, *J* = 10.1 Hz, 1H, NH), 5.76 (dd, *J* = 9.9, 4.3 Hz, 1H), 5.15 (dd, *J* = 12.6, 31.5 Hz, 2H), 4.06 (d, *J* = 12.0 Hz, 1H), 3.78 (s, 3H), 3.76 (s, 3H), 2.10-2.07 (m, 1H), 0.71 (d, *J* = 7.1 Hz, 3H)
^{13}C NMR (75 MHz, CD_3CN)	**δ :** 159.8 (C), 157.5 (C), 156.6 (2 C), 138.1 (C), 136.4 (C), 134.3 (C), 130.0 (2 CH), 129.5 (2 CH), 129.0 (CH), 128.7 (2 CH), 124.4 (C), 114.4 (2 CH), 112.7 (CH), 110.6 (CH), 108.9 (CH), 67.4 (CH_2), 63.1 (CH), 61.4 (CH), 56.5 (CH_3), 55.9 (CH_3), 37.3 (CH), 13.5 (CH_3)
IR (Neat, cm^{-1})	2959, 2835, 1712, 1612, 1583, 1541, 1513, 1488, 1457, 1303, 1246, 1200, 1177, 1105, 1074, 1035
HRMS (ESI+, m/z)	$[M+H]^+$ calcd. for $C_{27}H_{28}N_3O_4S$ 490.1801, found 490.1806
HPLC Analysis	Daicel Chiralpak AS-H, Hexane/EtOH = 95/5, flow rate = 1 mL/min, 254 nm<br>minor isomer: t_R = 5.72 min, major isomer: t_R = 8.35 min
Enantiomeric excess	>99%
$[\alpha]_D^{22}$	-142.33° (*c* 0.3, $CHCl_3$)

benzyl ((2*R*,3*S*,4*R*)-8-methoxy-2-(4-methoxyphenyl)-3-methyl-3,4-dihydro-2*H*-benzo[4,5]thiazolo[3,2-*a*]pyrimidin-4-yl)carbamate

4h

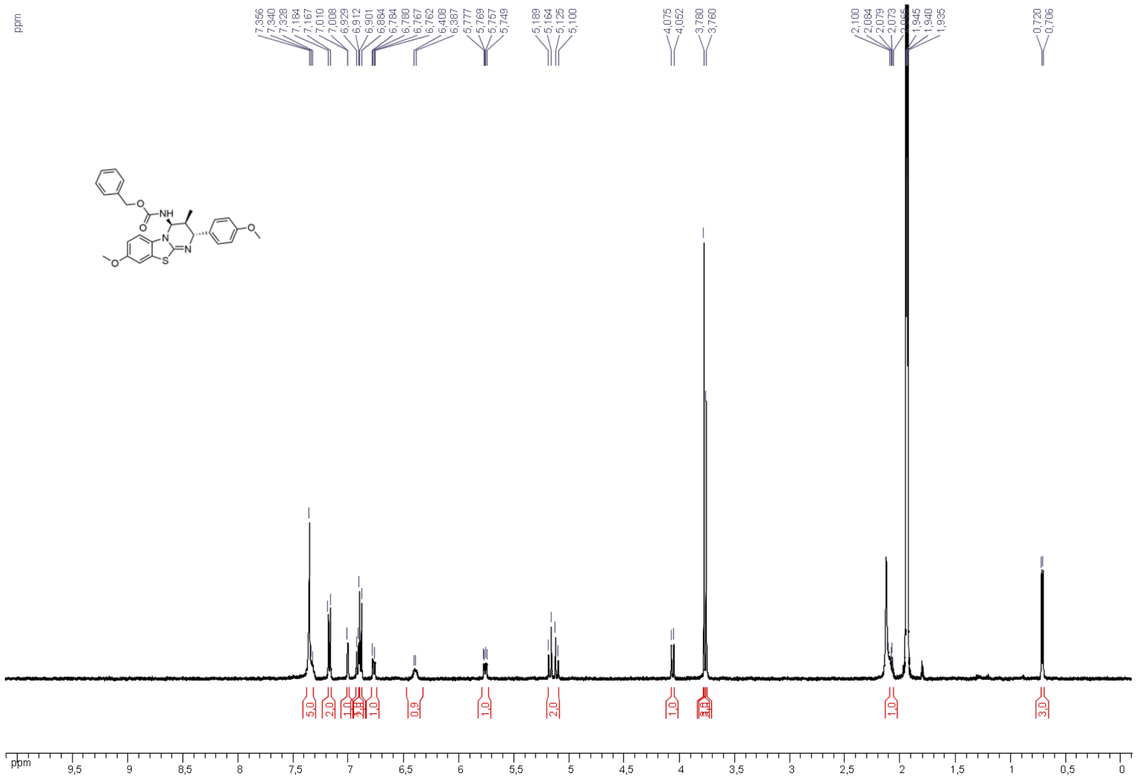

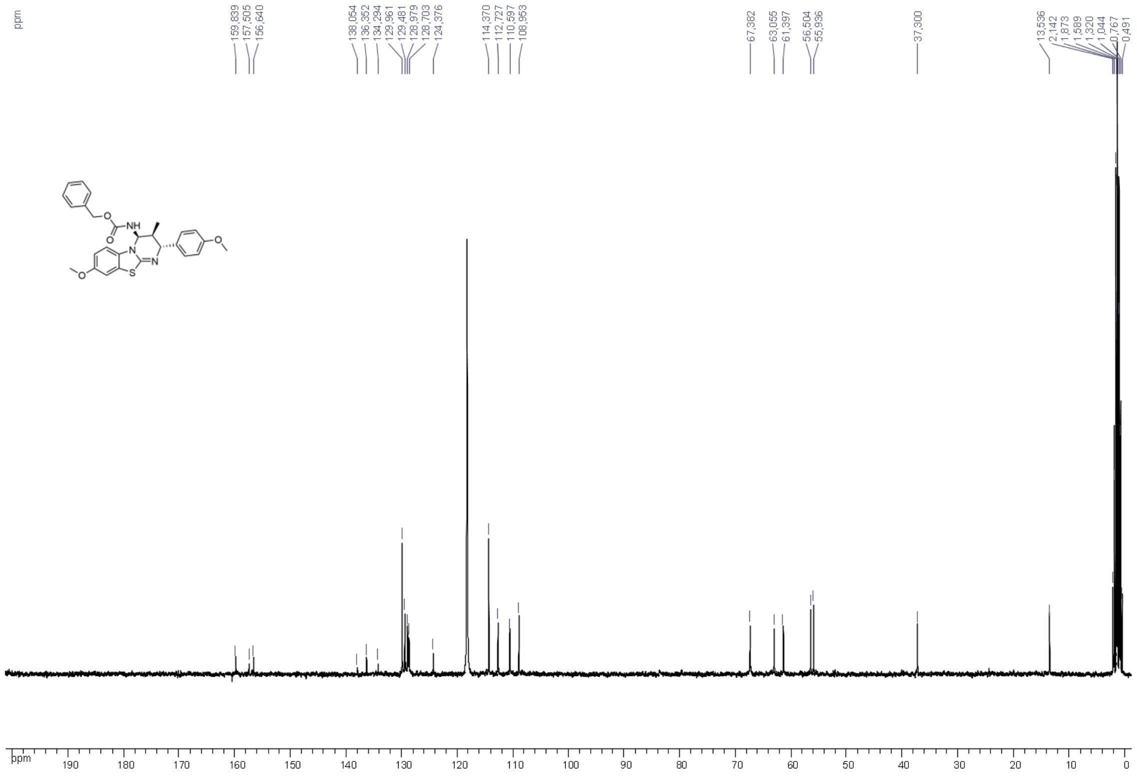

benzyl ((2*R*,3*S*,4*R*)-8-methoxy-2-(4-methoxyphenyl)-3-methyl-3,4-dihydro-2*H*-benzo[4,5]thiazolo[3,2-*a*]pyrimidin-4-yl)carbamate
4h

Chiralpak AS-H, Hexane/EtOH = 95/5, 1 mL/min, 254 nm

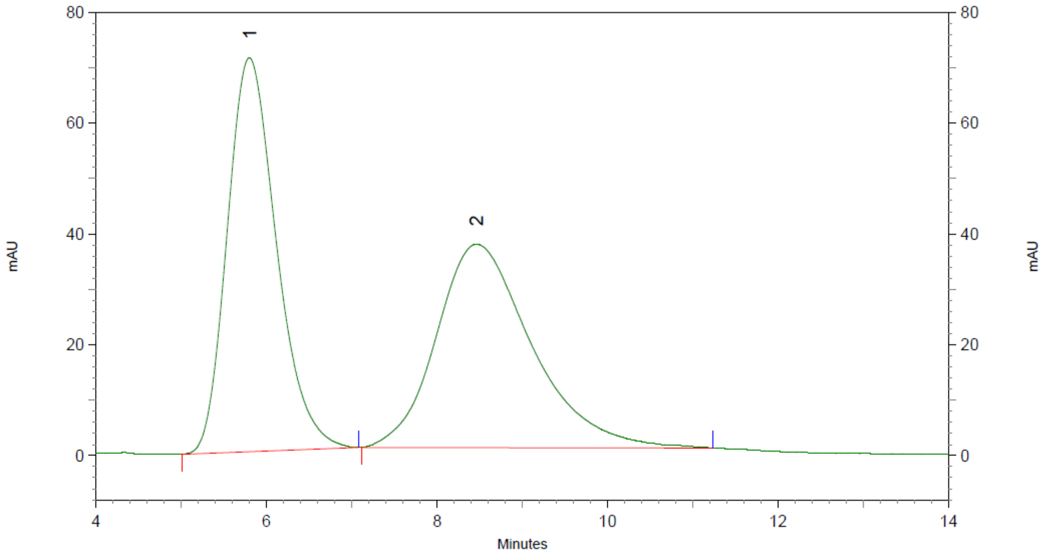

DAD-CH1 254 nm
Results

Pk #	Retention Time	Area	Area %
1	5,80	11352250	49,88
2	8,46	11404760	50,12

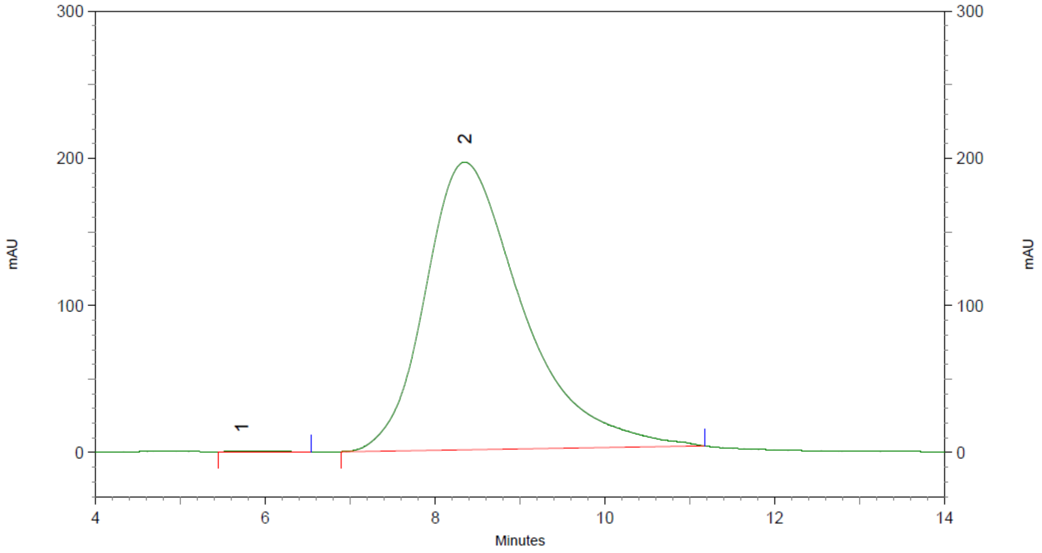

DAD-CH1 254 nm
Results

Pk #	Retention Time	Area	Area %
1	5,72	55684	0,09
2	8,35	62354244	99,91

benzyl ((2*R*,3*S*,4*R*)-2-(4-cyanophenyl)-8-fluoro-3-methyl-3,4-dihydro-2*H*-benzo[4,5]thiazolo[3,2-*a*]pyrimidin-4-yl)carbamate

4i

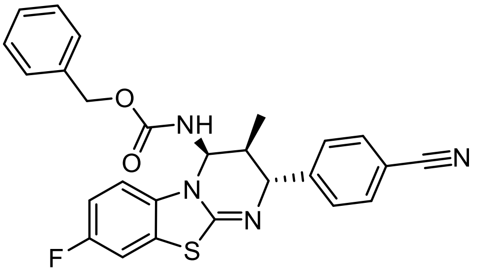

Compound **4i** was prepared according to the general procedure **D** from 2-benzothiazolimine **2i** (28.1 mg, 0.1 mmol) and enecarbamate **3a** (57.4 mg, 0.3 mmol) as starting materials. Purification on a column of silica gel with a gradient of ethyl acetate in heptane (75:25) as eluent gave the desired product as only one diastereomer.

Chemical formula	$C_{26}H_{21}FN_4O_2S$
M (g.mol^{-1})	472.54
Yield	44.7 mg, 95%
Aspect	Colorless oil
Rf	0.2 (Hept/AcOEt : 7/3)
^{1}H NMR *(300 MHz, Acetone-d$_6$)*	**δ :** 7.72 (d, *J* = 8.3 Hz, 2H), 7.65 (d, *J* = 10, 3 Hz, 1H, NH), 7.46 (d, *J* = 8.4 Hz, 2H), 7.40-7.30 (m, 5H), 7.23 (dd, *J* = 8.4, 2.5 Hz, 1H), 7.13 (dd, *J* = 9.0, 4.5 Hz, 1H), 7.01 (td, *J* = 8.9, 2.5 Hz, 1H), 5.90 (dd, *J* = 9.9, 4.1 Hz, 1H), 5.20 (s, 2H), 4.35 (d, *J* = 11.6 Hz, 1H), 2.19-2.11 (m, 1H), 0.74 (d, *J* = 7.0 Hz, 3H)
^{13}C NMR *(75 MHz, Acetone-d$_6$)*	**δ :** 159.21 (d, *J* = 269.8 Hz, CF), 157.8 (C), 156.9 (C), 150.0 (C), 137.8 (C), 136.8 (C), 132.8 (2 CH), 129.9 (2 CH), 129.3 (2 CH), 128.9 (CH), 128.7 (2 CH), 124.6 (d, *J* = 10.9 Hz, C), 119.4 (C), 113.5 (d, *J* = 24,1 Hz, CH), 111.7 (C), 110.7 (d, *J* = 8.8 Hz, CH), 110.2 (d, *J* = 27.5 Hz, CH), 67.3 (CH_2), 63.0 (CH), 61.8 (CH), 37.1 (CH), 13.4 (CH_3)
^{19}F NMR *(282 MHz, Acetone-d$_6$)*	**δ :** -55,35
IR (Neat, cm^{-1})	3304, 3158, 2964, 2935, 2229, 1710, 1621, 1607,1591, 1535, 1480, 1456, 1429, 1414, 1385, 1339, 1318, 1298, 1266, 1255, 1229, 1187, 1125, 1104, 1071
HRMS (ESI+, m/z)	$[M+H]^+$ calcd. for $C_{26}H_{22}FN_4O_2S$ 473.1448, found 473.1448
HPLC Analysis	Daicel Chiralpak AD-H, Heptane/EtOH = 90/10, flow rate = 1 mL/min, 254 nm major isomer: t_R = 18.03 min, minor isomer: t_R = 33.81 min
Enantiomeric excess	97%
$[\alpha]_D^{22}$	-6.98° (*c* 4.23, $CHCl_3$)

benzyl ((2*R*,3*S*,4*R*)-2-(4-cyanophenyl)-8-fluoro-3-methyl-3,4-dihydro-2*H*-benzo[4,5]thiazolo[3,2-*a*]pyrimidin-4-yl)carbamate
4i

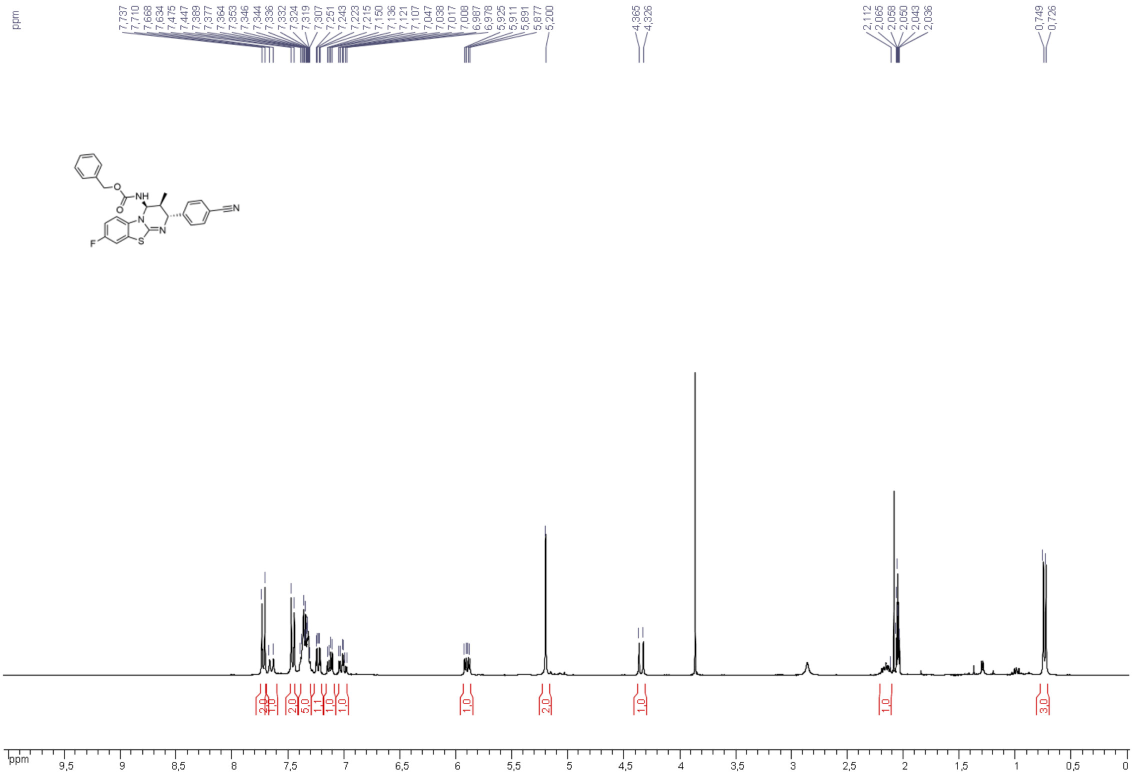

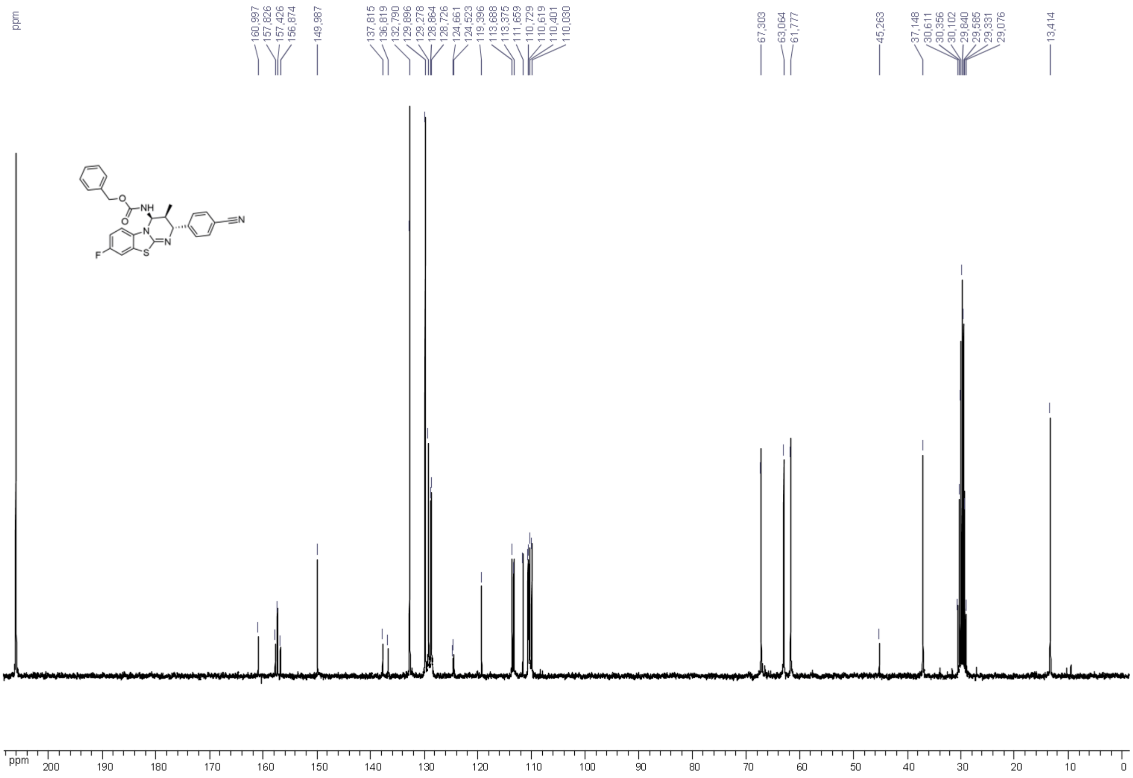

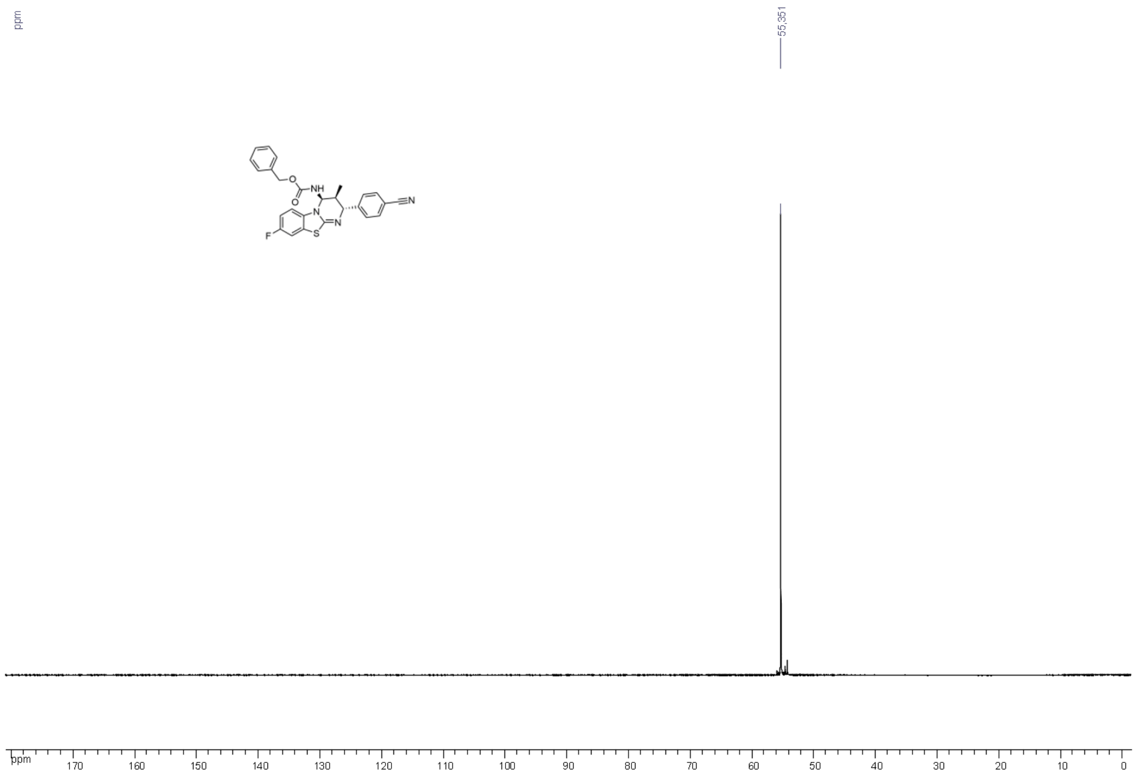
ppm
55.351
NH
O
N
S
F
N
ppm
170
160
150
140
130
120
110
100
90
80
70
60
50
40
30
20
10
0

benzyl ((2*R*,3*S*,4*R*)-2-(4-cyanophenyl)-8-fluoro-3-methyl-3,4-dihydro-2*H*-benzo[4,5]thiazolo[3,2-*a*]pyrimidin-4-yl)carbamate
4i

Chiralpak AD-H, Heptane/EtOH = 90/10, 1 mL/min, 254 nm

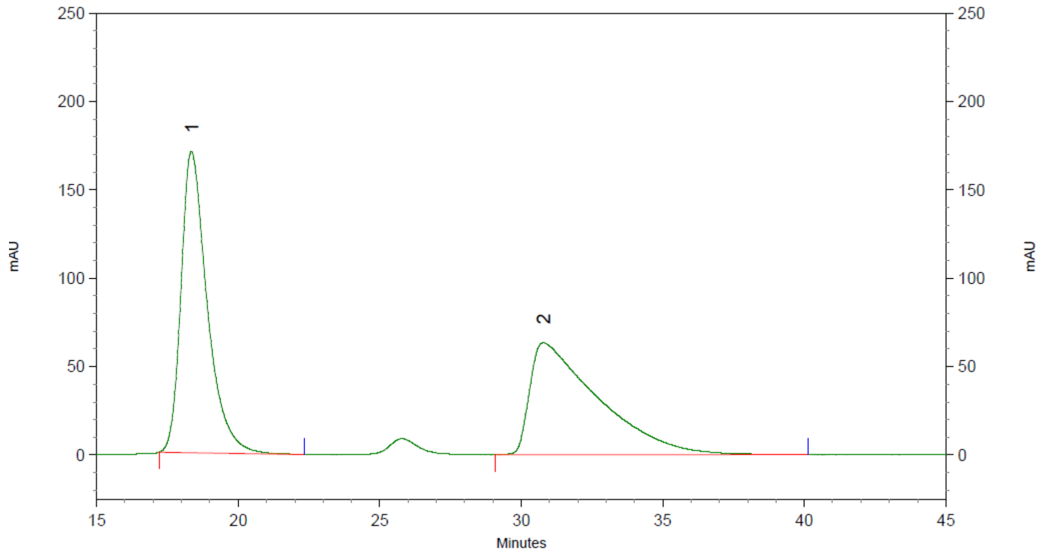

DAD-CH1 254 nm
Results

Pk #	Retention Time	Area	Area %
1	18,35	43805634	50,85
2	30,78	42334611	49,15

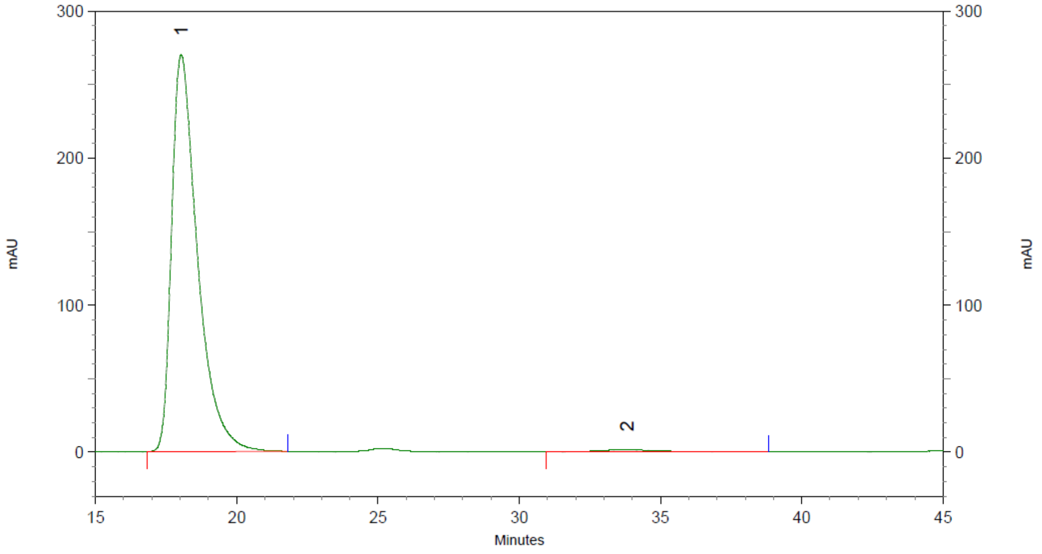

DAD-CH1 254 nm
Results

Pk #	Retention Time	Area	Area %
1	18,03	68874728	98,63
2	33,81	957866	1,37

benzyl ((2*R*,3*S*,4*R*)-2-(4-bromo-2-chlorophenyl)-8-fluoro-3-methyl-3,4-dihydro-2*H*-benzo[4,5]thiazolo[3,2-*a*]pyrimidin-4-yl)carbamate

4j

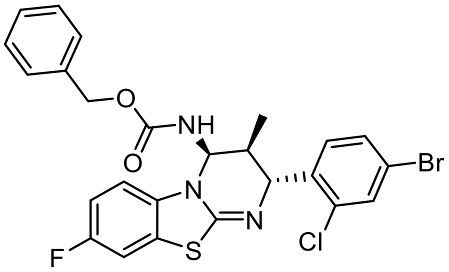

Compound **4j** was prepared according to the general procedure **D** from 2-benzothiazolimine **2j** (37.0 mg, 0.1 mmol) and enecarbamate **3a** (57.4 mg, 0.3 mmol) as starting materials. Purification on a column of silica gel with a gradient of ethyl acetate in heptane (from 9:1 to 8:2) as eluent gave the desired product as only one diastereomer.

Chemical formula	$C_{25}H_{20}BrClFN_3O_2S$
M (g.mol^{-1})	560.87
Yield	52.2 mg, 93%
Aspect	White foam
Rf	0.6 (Hept/AcOEt : 7/3)
^{1}H NMR (300 MHz, Acetone-d$_6$)	**δ :** 7.63 (d, *J* = 11.0 Hz, 1H, NH), 7.60 (d, *J* = 2.3 Hz, 1H), 7.54 (dd, *J* = 8.4, 2.3 Hz, 1H), 7.38 (d, *J* = 4.8 Hz, 1H), 7.36-7.31 (m, 6H), 7.05 (td, *J* = 8.9, 4.4 Hz, 1H), 6.99 (*J* = 8.9, 2.7 Hz, 1H), 5.91 (dd, *J* = 10.1, 4.6 Hz, 1H), 5.23 (d, *J* = 12.4 Hz, 1H), 5.15 (d, *J* = 12.4 Hz, 1H), 4.93 (d, *J* = 12.1 Hz, 1H), 2.30 (m, 1H), 0.89 (d, *J* = 6.7 Hz, 3H)
^{13}C NMR (75 MHz, Acetone-d$_6$)	**δ :** 159.35 (d, *J* = 238.7 Hz, CF) 151.5 (C), 156.2 (C), 142.0 (C), 137.8 (C), 136.9 (C), 135.0 (C), 132.5 (CH), 131.9 (CH), 131.5 (CH), 129.3 (2 CH), 128.8 (CH), 128.6 (2 CH), 124.6 (d, *J* = 10.9 Hz, C), 121.2 (C), 113.5 (d, *J* = 24.4 Hz, CH), 110.4 (d, *J* = 27.8 Hz, CH), 110.3 (d, *J* = 9.0 Hz, CH), 67.3 (CH_2), 63.0 (CH), 57.3 (CH), 38.1 (CH), 12.7 (CH_3)
^{19}F NMR (282 MHz, Acetone-d$_6$)	**δ :** -55.18
IR (Neat, cm^{-1})	3299, 3033, 2971, 1689, 1624, 1592, 1534, 1480, 1456, 1428, 1380, 1342, 1317, 1272, 1258, 1228, 1187, 1143, 1123, 1106, 1073, 1050
HRMS (ESI+, m/z)	$[M+H]^+$ calcd. for $C_{25}H_{21}BrClFN_3O_2S$ 560.0210, found 560.0226
HPLC Analysis	Daicel Chiralpak IA, Heptane/EtOH = 90/10, flow rate = 1 mL/min, 254 nm<br>minor isomer: t_R = 11.14 min, major isomer: t_R = 14.51 min
Enantiomeric excess	98%
$[\alpha]_D^{22}$	-4.34° (*c* 4.99, $CHCl_3$)

benzyl ((2*R*,3*S*,4*R*)-2-(4-bromo-2-chlorophenyl)-8-fluoro-3-methyl-3,4-dihydro-2*H*-benzo[4,5]thiazolo[3,2-*a*]pyrimidin-4-yl)carbamate

4j

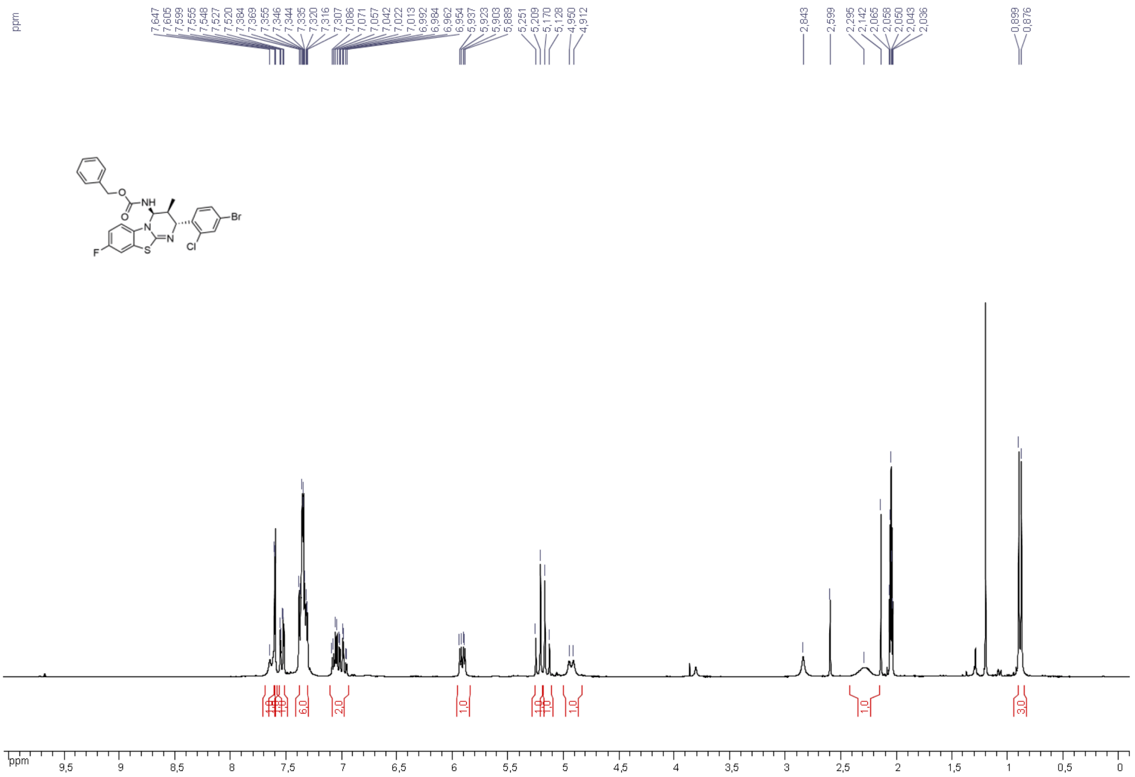

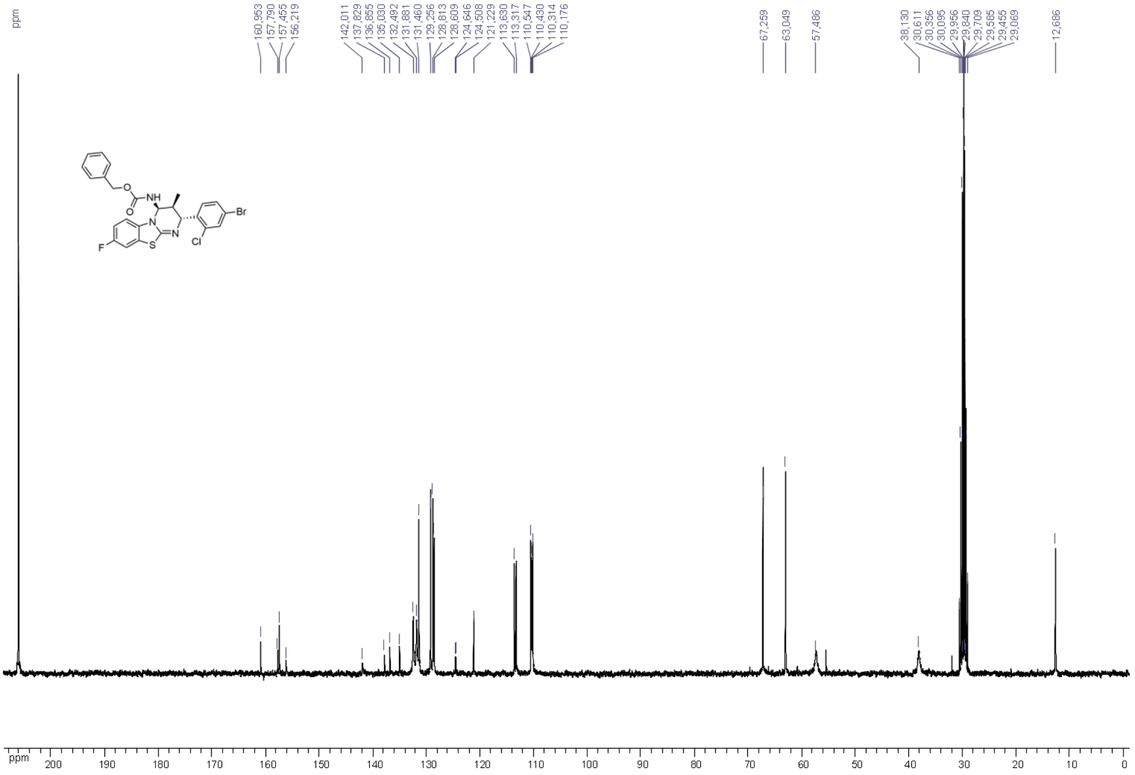

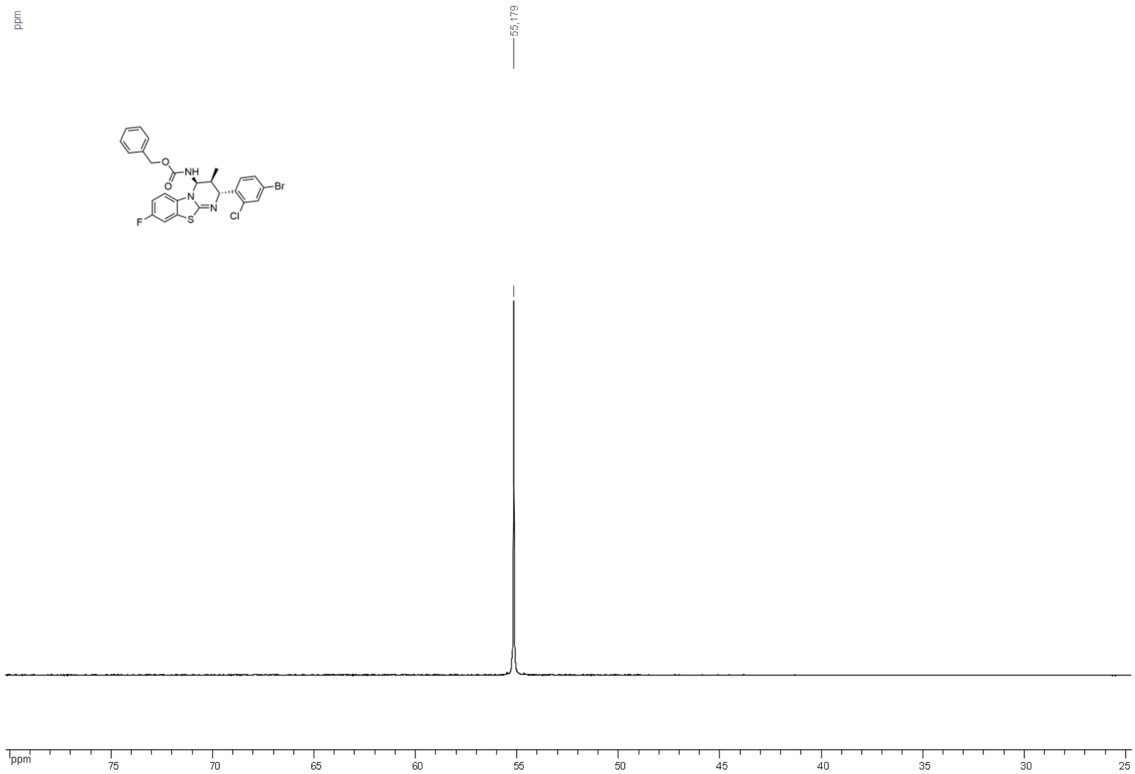
ppm
55.179
NH
O
O
N
N
S
F
Cl
Br
ppm
75
70
65
60
55
50
45
40
35
30
25

benzyl ((2*R*,3*S*,4*R*)-2-(4-bromo-2-chlorophenyl)-8-fluoro-3-methyl-3,4-dihydro-2*H*-benzo[4,5]thiazolo[3,2-*a*]pyrimidin-4-yl)carbamate

4j

Chiralpak IA, Heptane/EtOH = 90/10, 1 mL/min, 254 nm

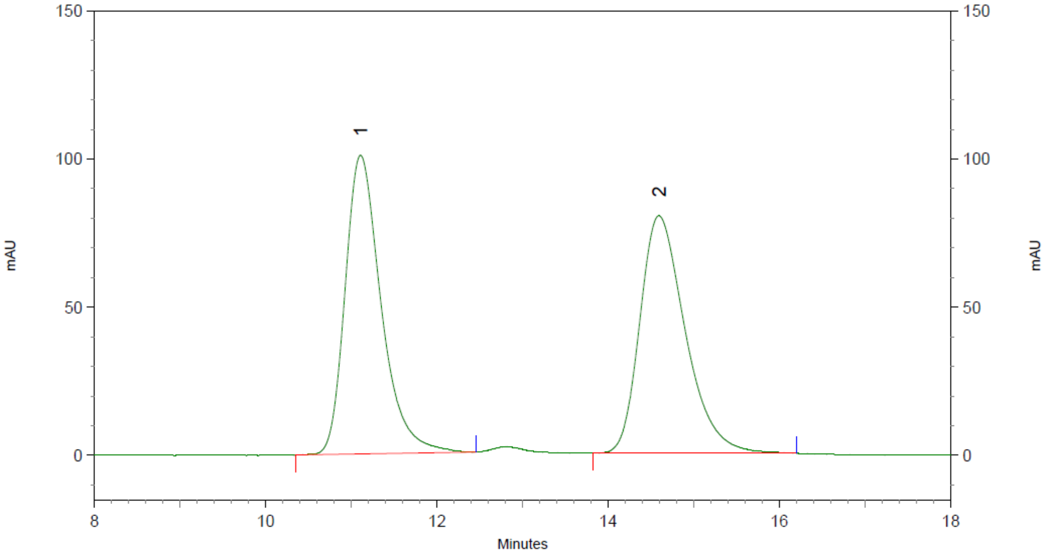

DAD-CH1 254 nm

Results

Pk #	Retention Time	Area	Area %
1	11,11	11750036	49,94
2	14,59	11777992	50,06

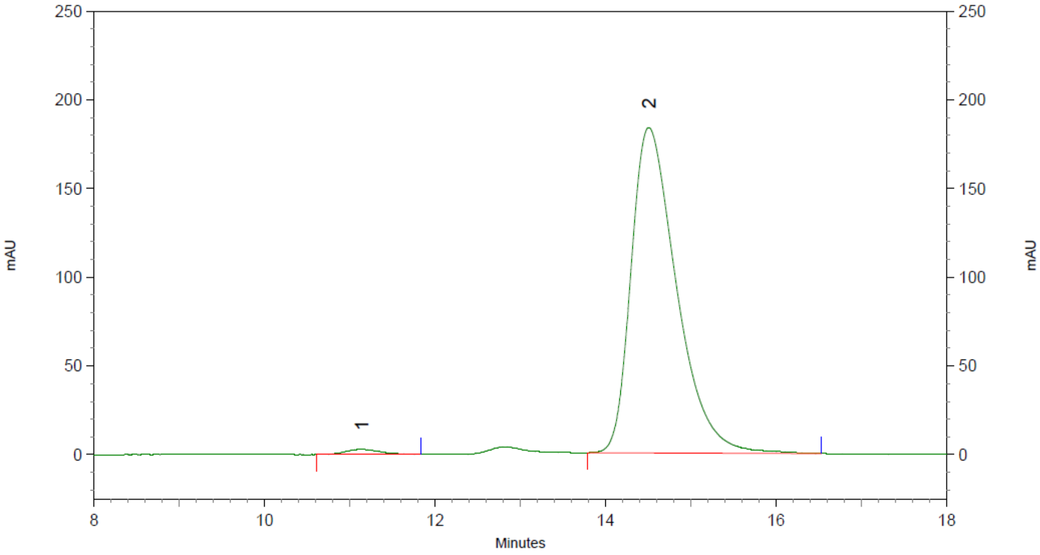

DAD-CH1 254 nm

Results

Pk #	Retention Time	Area	Area %
1	11,14	332457	1,21
2	14,51	27110704	98,79

benzyl ((2*R*,3*S*,4*R*)-2-(3-bromophenyl)-8-methoxy-3-methyl-3,4-dihydro-2*H*-benzo[4,5]thiazolo[3,2-*a*]pyrimidin-4-yl)carbamate

4k

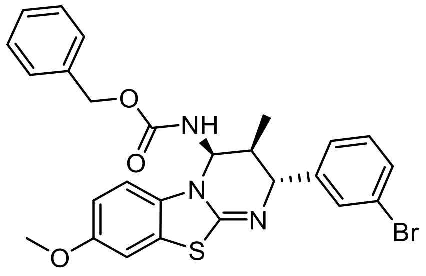

Compound **4k** was prepared according to the general procedure **D** from 2-benzothiazolimine **2k** (34.7 mg, 0.1 mmol) and enecarbamate **3a** (57.4 mg, 0.3 mmol) as starting materials. Purification on a column of silica gel with a gradient of ethyl acetate in heptane (from 85:15 to 75:25) as eluent gave the desired product as only one diastereomer.

Chemical formula	$C_{26}H_{24}BrN_3O_3S$
M (g.mol^{-1})	538.46
Yield	43.0 mg, 80%
Aspect	White foam
Rf	0.2 (Hept/AcOEt : 7/3)
^{1}H NMR (300 MHz, CD_3CN)	**δ :** 7.46-7.43 (m, 2H), 7.38-7.27 (m, 6H), 7.25 (t, *J* = 8.1 Hz, 1H), 7.04 (d, *J* = 7.2 Hz, 1H), 6.98 (d, *J* = 9.5 Hz, 1H), 6.80-6.77 (m, 2H), 5.75 (dd, *J* = 10.7, 4.3 Hz, 1H), 5.23 (d, *J* = 12.2 Hz, 1H), 5.11 (d, *J* = 13.5 Hz, 1H), 3.97 (d, *J* = 11.9 Hz, 1H), 3.77 (s, 3H), 2.00-1.98 (m, 1H), 0.59 (d, *J* = 7.0 Hz, 3H)
^{13}C NMR (75 MHz, CD_3CN)	**δ :** 157.5 (2 C), 156.7 (C), 147.1 (C), 138.0 (C), 134.2 (C), 131.7 (CH), 131.1 (CH), 131.0 (CH), 129.4 (2 CH), 128.9 (CH), 128.6 (2 CH), 128.1 (CH), 124.4 (C), 122.9 (C), 112.7 (CH), 110.7 (CH), 108.9 (CH), 67.4 (CH_2), 62.9 (CH), 61.7 (CH), 56.5 (CH_3), 37.3 (CH), 13.4 (CH_3)
IR (Neat, cm^{-1})	2960, 1716, 1622, 1585, 1548, 1488, 1282, 1239, 1202, 1074, 1033
HRMS (ESI+, m/z)	$[M+H]^+$ calcd. for $C_{26}H_{25}BrN_3O_3S$ 538.0800, found 538.0804
HPLC Analysis	Daicel Chiralpak OD-H, Hexane/EtOH = 95/5, flow rate = 1 mL/min, 254 nm<br>minor isomer: t_R = 14.30 min, major isomer: t_R = 16.26 min
Enantiomeric excess	98%<br>99% (after recrystallization)
$[\alpha]_D^{22}$	-97.33° (*c* 0.3, $CHCl_3$)

benzyl ((2*R*,3*S*,4*R*)-2-(3-bromophenyl)-8-methoxy-3-methyl-3,4-dihydro-2*H*-benzo[4,5]thiazolo[3,2-*a*]pyrimidin-4-yl)carbamate
4k

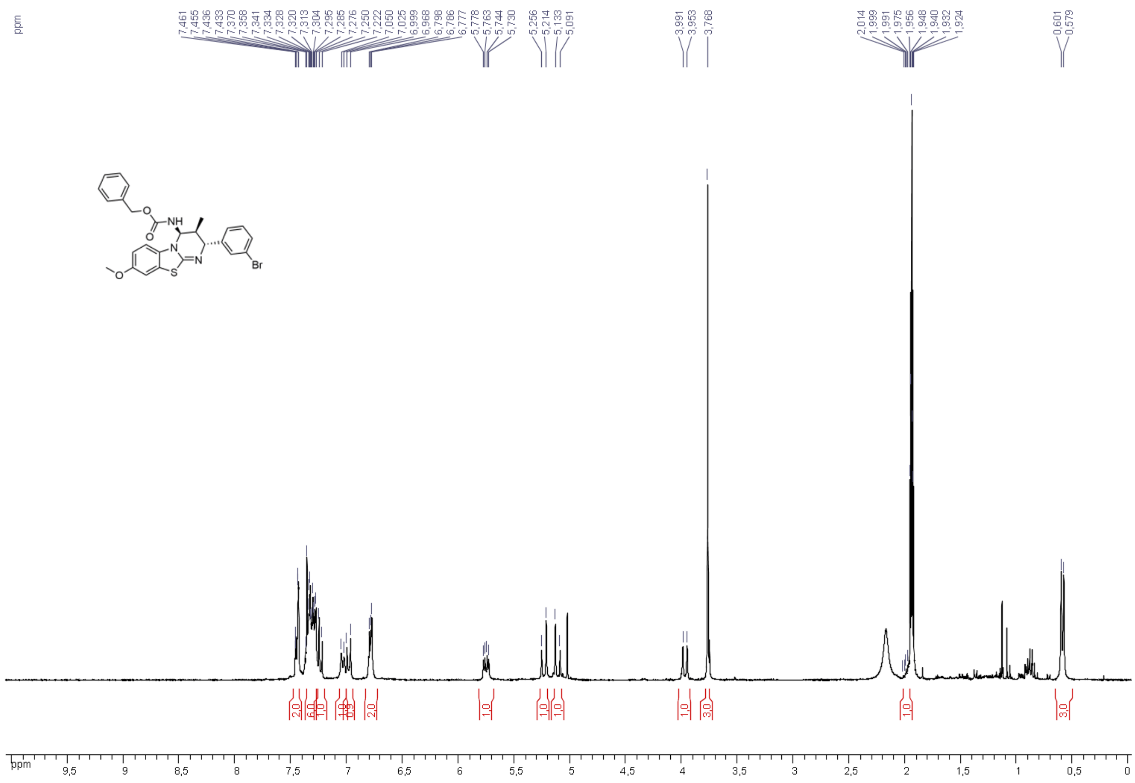

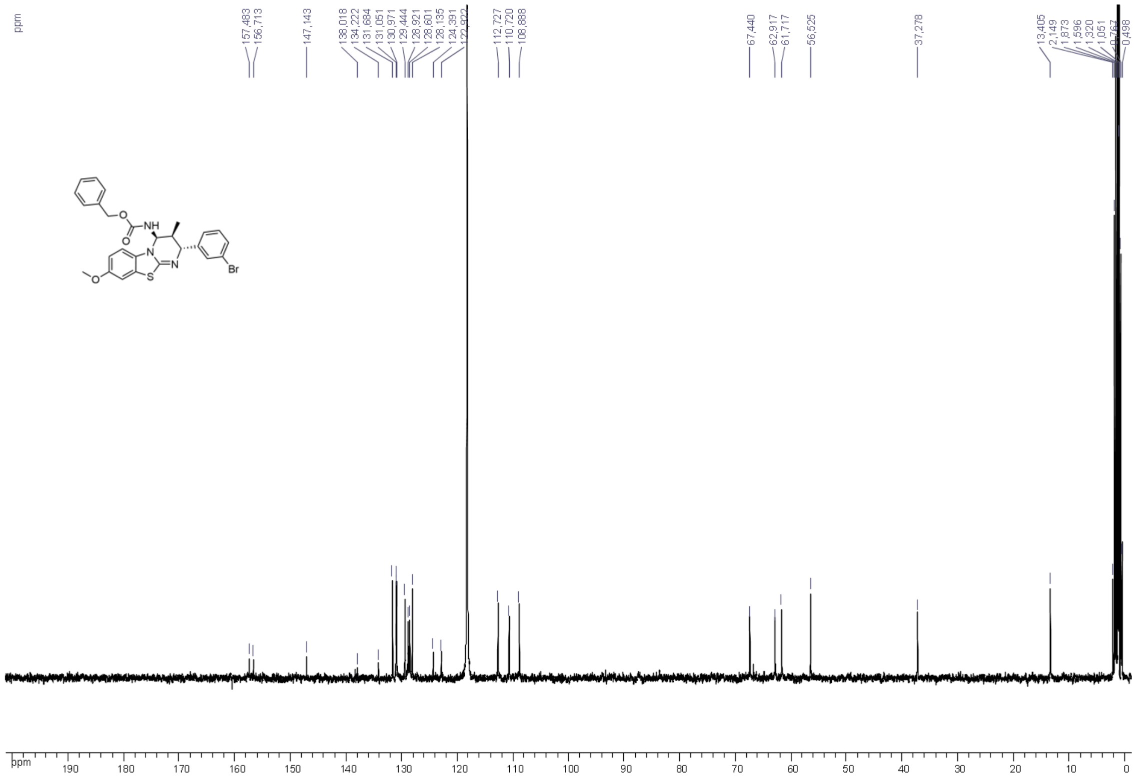

benzyl ((2*R*,3*S*,4*R*)-2-(3-bromophenyl)-8-methoxy-3-methyl-3,4-dihydro-2*H*-benzo[4,5]thiazolo[3,2-*a*]pyrimidin-4-yl)carbamate
4k

Chiralpak OD-H, Hexane/EtOH = 95/5, 1 mL/min, 254 nm

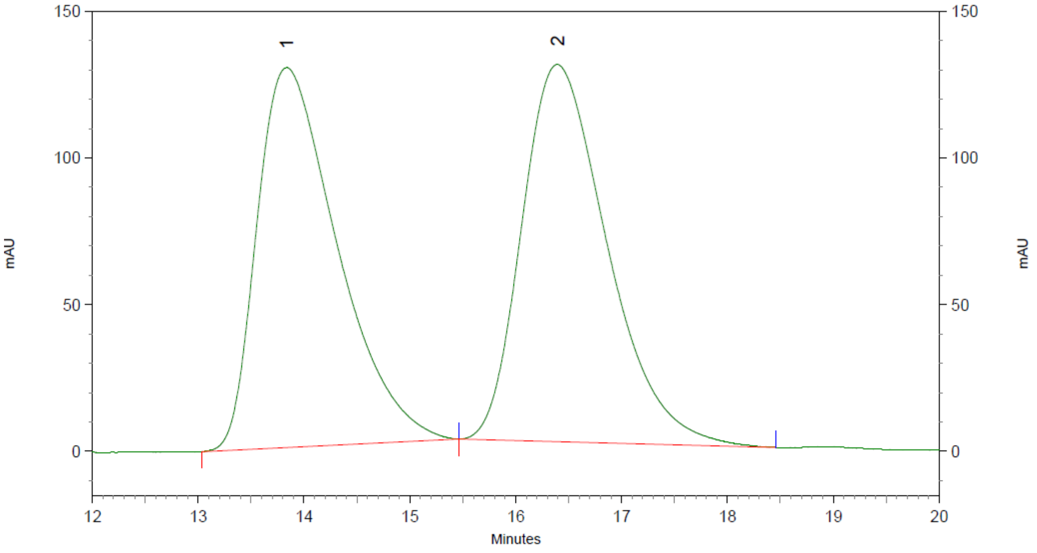

DAD-CH1 254 nm
Results

Pk #	Retention Time	Area	Area %
1	13,83	27281764	48,69
2	16,39	28753091	51,31

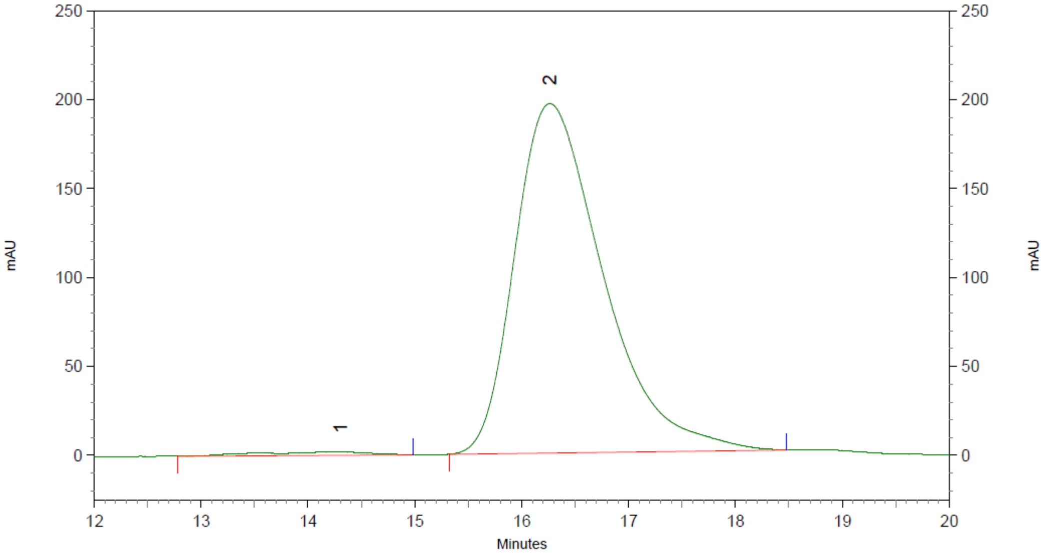

DAD-CH1 254 nm
Results

Pk #	Retention Time	Area	Area %
1	14,30	561372	1,23
2	16,26	45143512	98,77

After recrystallization:

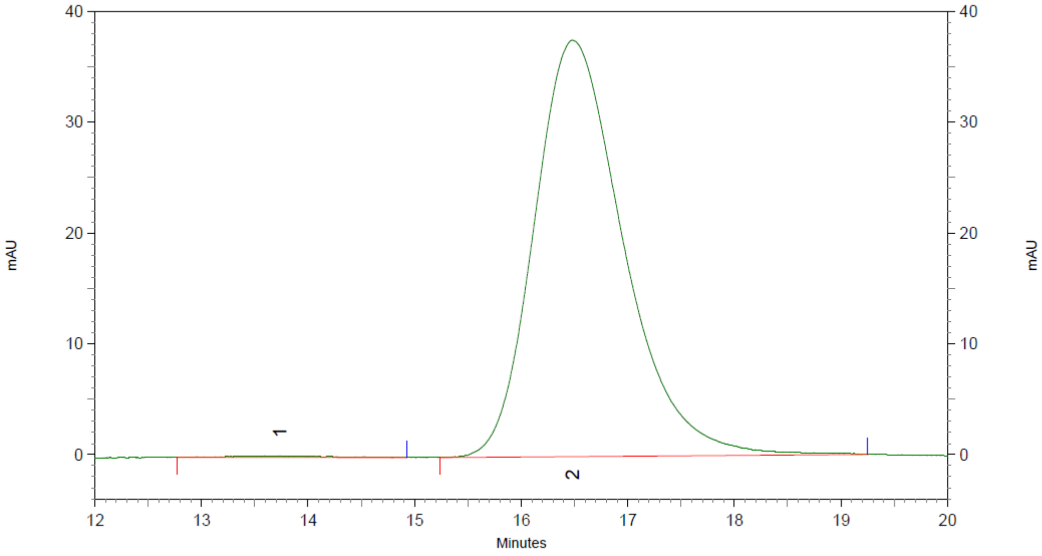

DAD-CH1 254 nm

Results

Pk #	Retention Time	Area	Area %
1	13,73	35062	0,40
2	16,48	8723739	99,60

benzyl ((2*R*,3*S*,4*R*)-3-methyl-2-(thiophen-2-yl)-3,4-dihydro-2*H*-benzo[4,5]thiazolo[3,2-*a*]pyrimidin-4-yl)carbamate

4l

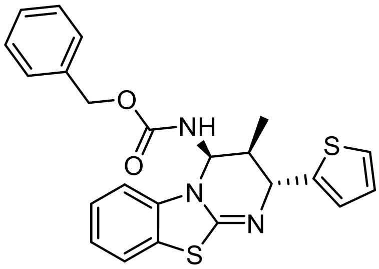

Compound **4l** was prepared according to the general procedure **D** from 2-benzothiazolimine **2l** (24.4 mg, 0.1 mmol) and enecarbamate **3a** (57.4 mg, 0.3 mmol) as starting materials. Purification on a column of silica gel with a gradient of ethyl acetate in heptane (from 8:2 to 75:25) as eluent gave the desired product as only one diastereomer.

Chemical formula	$C_{23}H_{21}N_3O_2S_2$
M (g.mol^{-1})	435.56
Yield	43.1 mg, 99%
Aspect	White foam
Rf	0.3 (Hept/AcOEt : 7/3)
^{1}H NMR (300 MHz, Acetone-d$_6$)	**δ :** 7.59 (d, *J* = 10.2 Hz, 1H, NH), 7.37-7.29 (m, 7H), 7.22 (td, *J* = 7.5, 1.3 Hz, 1H), 7.14 (d, *J* = 7.5 Hz, 1H), 7.05 (td, *J* = 7.5, 1.3 Hz, 1H), 6.98-6.94 (m, 2H), 5.92 (dd, *J* = 10.3, 4.2 Hz, 1H), 5.19 (s, 2H), 4.64 (d, *J* = 11.7 Hz, 1H), 2.20-2.12 (m, 1H), 0.86 (d, *J* = 6.4 Hz, 3H)
^{13}C NMR (75 MHz, Acetone-d$_6$)	**δ :** 157.4 (C), 156.7 (C), 148.9 (C), 140.3 (C), 137.9 (C), 1329.2 (2 CH), 128.7 (CH), 128.5 (2 CH), 127.0 (CH), 126.9 (CH), 125.3 (CH), 125.1 (CH), 123.1 (CH), 123.0 (C), 122.7 (CH), 110.0 (CH), 67.1 (CH_2), 62.9 (CH), 58.2 (CH), 37.9 (CH), 13.7 (CH_3)
IR (Neat, cm^{-1})	3300, 3066, 3032, 2964, 1705, 1613, 1587, 1532, 1497, 1471, 1456, 1413, 1338, 1320, 1280, 1247, 1207, 1136, 1106, 1064, 1027
HRMS (ESI+, m/z)	$[M+H]^+$ calcd. for $C_{23}H_{22}N_3O_2S_2$ 436.1153, found 436.1136
HPLC Analysis	Daicel Chiralpak IA, Heptane/EtOH = 90/10, flow rate = 1 mL/min, 254 nm<br>major isomer: t_R = 16.67 min, minor isomer: t_R = 41.22 min
Enantiomeric excess	98%
$[\alpha]_D^{22}$	-2.77° (*c* 4.35, $CHCl_3$)

benzyl ((2*R*,3*S*,4*R*)-3-methyl-2-(thiophen-2-yl)-3,4-dihydro-2*H*-benzo[4,5]thiazolo[3,2-*a*]pyrimidin-4-yl)carbamate

4l

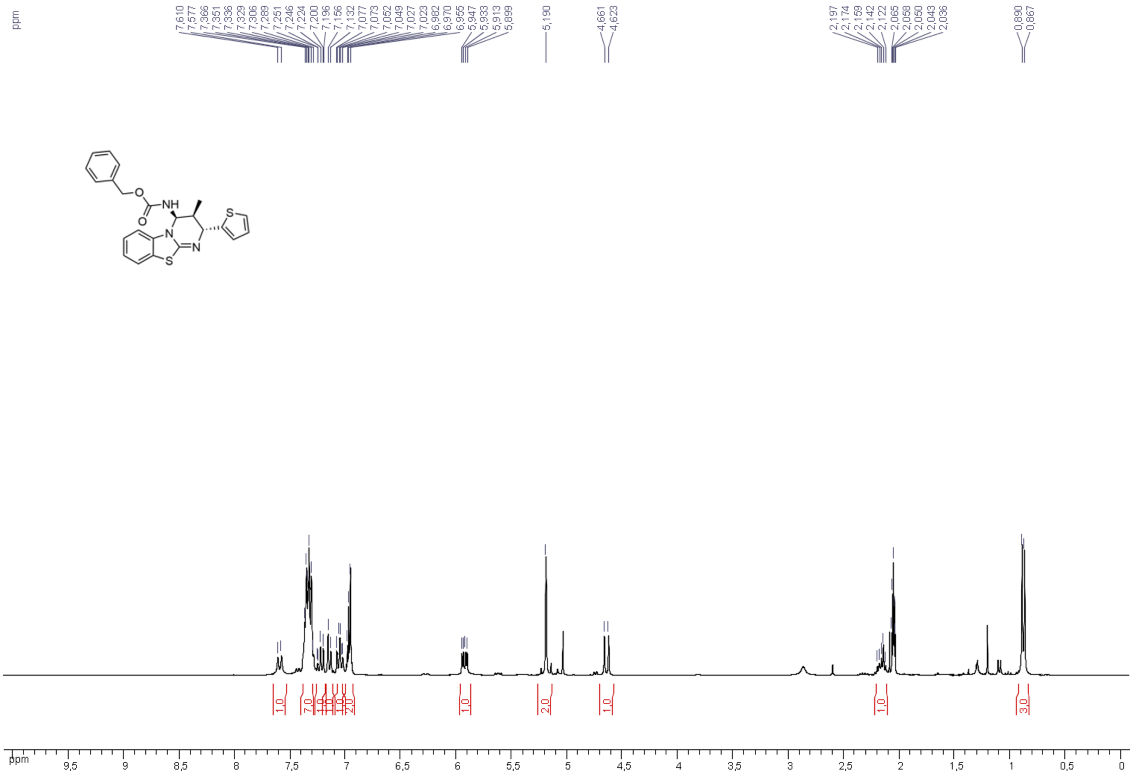

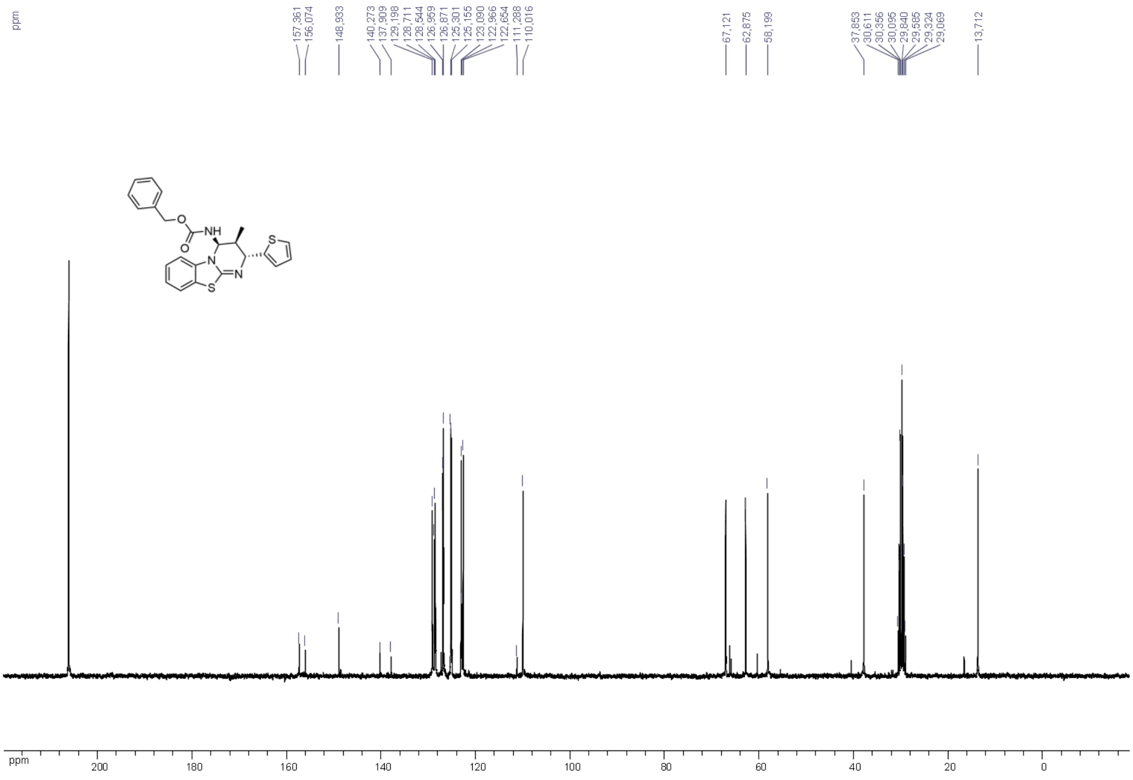

benzyl ((2*R*,3*S*,4*R*)-3-methyl-2-(thiophen-2-yl)-3,4-dihydro-2*H*-benzo[4,5]thiazolo[3,2-*a*]pyrimidin-4-yl)carbamate
4l

Chiralpak IA, Heptane/EtOH = 90/10, 1 mL/min, 254 nm

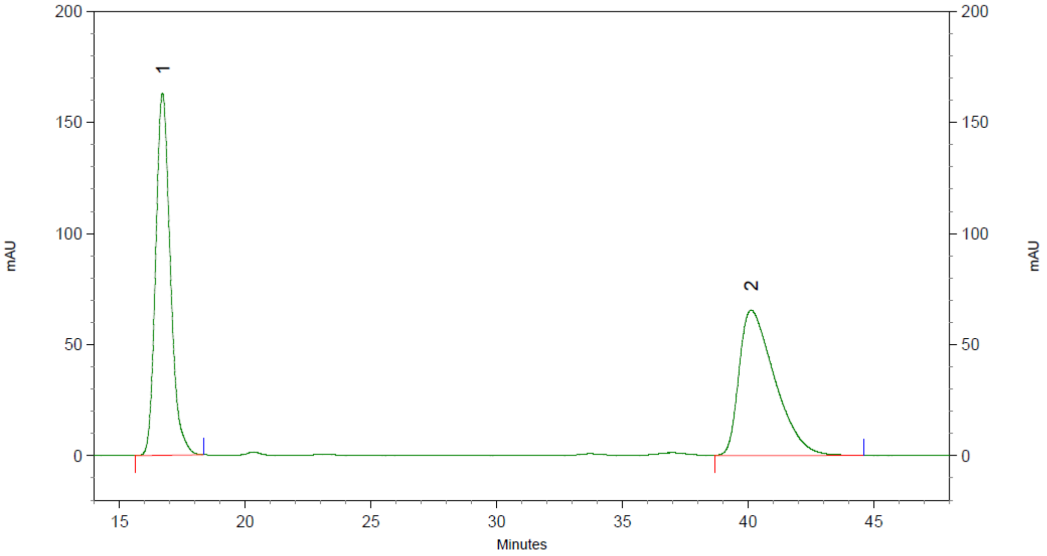

DAD-CH1 254 nm
Results

Pk #	Retention Time	Area	Area %
1	16,71	26344156	50,52
2	40,12	25805514	49,48

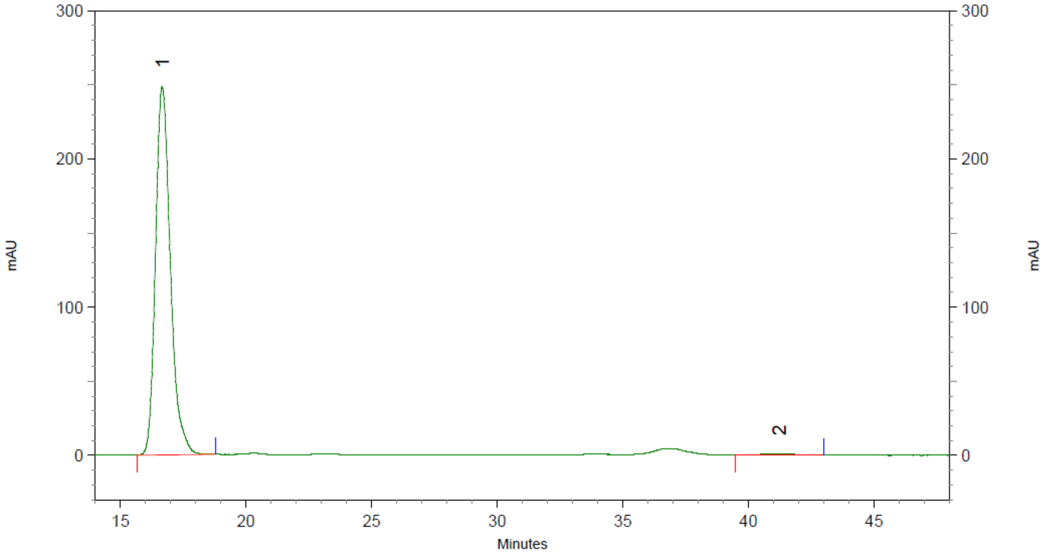

DAD-CH1 254 nm
Results

Pk #	Retention Time	Area	Area %
1	16,67	40893838	99,20
2	41,22	330896	0,80

***tert*-butyl ((2*R*,3*S*,4*R*)-2-(4-bromophenyl)-8-chloro-3-methyl-3,4-dihydro-2*H*-benzo[4,5]thiazolo[3,2-*a*]pyrimidin-4-yl)carbamate**

4m

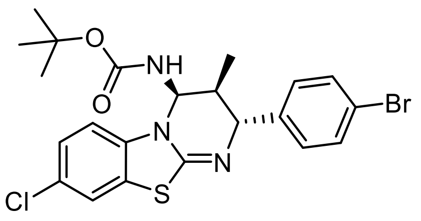

Compound **4m** was prepared according to the general procedure **D** from 2-benzothiazolimine **2f** (35.2 mg, 0.1 mmol) and enecarbamate **3b** (47.2 mg, 0.3 mmol) as starting materials. Purification on a column of silica gel with a gradient of ethyl acetate in heptane (from 9:1 to 85:15) as eluent gave the desired product as only one diastereomer.

Chemical formula	$C_{22}H_{23}BrClN_3O_2S$
M (g.mol^{-1})	508.86
Yield	49.5 mg, 97%
Aspect	White foam
Rf	0.8 (Hept/AcOEt : 7/3)
^{1}H NMR (300 MHz, Acetone-d$_6$)	**δ :** 8.09 (d, *J* = 1.7 Hz, 1H) 7.74 (d, *J* = 9.1 Hz, 1H), 7.69 (d, *J* = 8.6 Hz, 1H), 7.67 (dd, *J* = 9.1, 1.7 Hz, 1H), 7.62 (d, *J* = 8.6 Hz, 2H), 7. 51 (d, *J* = 8.6 Hz, 2H), 6.44 (dd, *J* = 10.6, 4.3 Hz, 1H), 4.87 (d, *J* = 11.7 Hz, 1H), 2.07-2.05 (m, 1H), 1.46 (s, 9H), 0.97 (d, *J* = 6.7 Hz, 3H)
^{13}C NMR (75 MHz, Acetone-d$_6$)	**δ :** 156.7 (C), 156.0 (C), 143.9 (C), 139.4 (C), 131.9 (2 CH), 130.9 (2 CH), 127.6 (C), 126.9 (CH), 125.1 (C), 122.5 (CH), 121.1 (C), 111.0 (CH), 80.2 (C), 62.6 (CH), 61.5 (CH), 37.2 (CH), 28.5 (3 CH_3), 13.5 (CH_3)
IR (Neat, cm^{-1})	3167, 2977, 2933, 1708, 1623, 1572, 1538, 1489, 1472, 1393, 1367, 1339, 1316, 1264, 1251, 1217, 1159, 1093, 1073, 1009
HRMS (ESI+, m/z)	$[M+H]^+$ calcd. for $C_{22}H_{24}BrClN_3O_2S$ 508.0461, found 508.0471
HPLC Analysis	Daicel Chiralpak IA, Heptane/EtOH = 95/5, flow rate = 1 mL/min, 254 nm<br>minor isomer: t_R = 8.20 min , major isomer: t_R = 9.87 min
Enantiomeric excess	98%
$[\alpha]_D^{22}$	-2.29° (*c* 2.36, $CHCl_3$)

tert-butyl ((2_R_,3_S_,4_R_)-2-(4-bromophenyl)-8-chloro-3-methyl-3,4-dihydro-2_H_-benzo[4,5]thiazolo[3,2-_a_]pyrimidin-4-yl)carbamate

4m

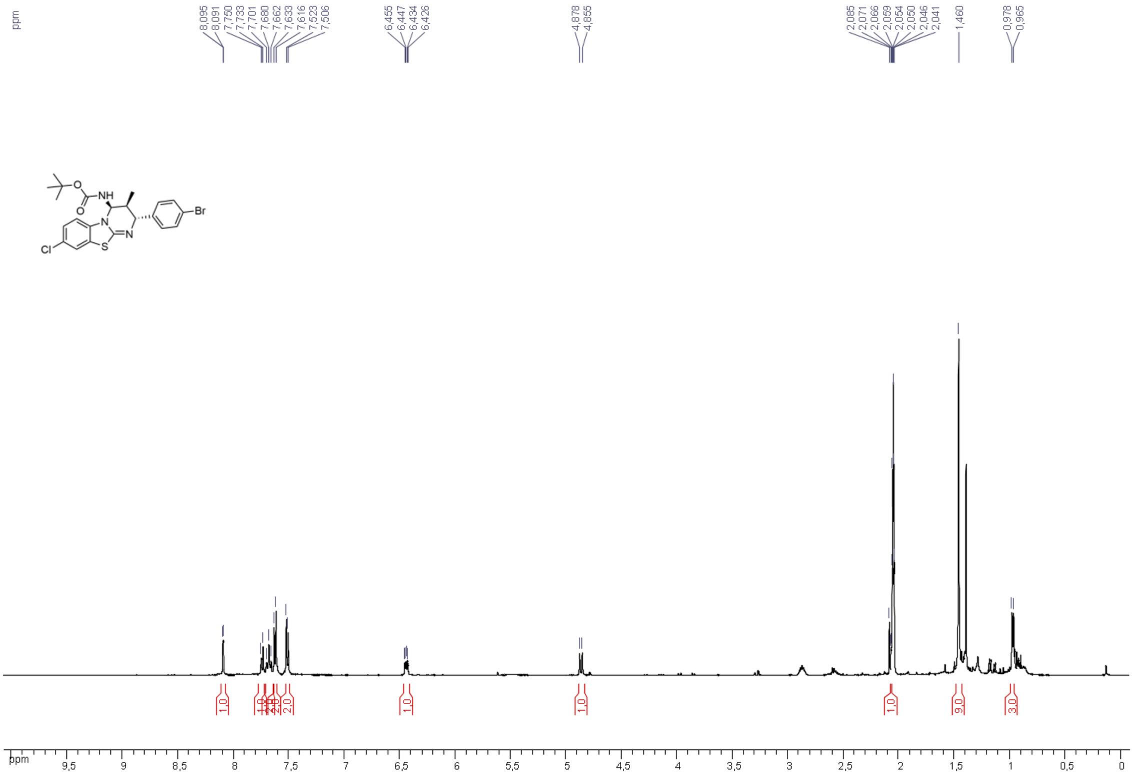

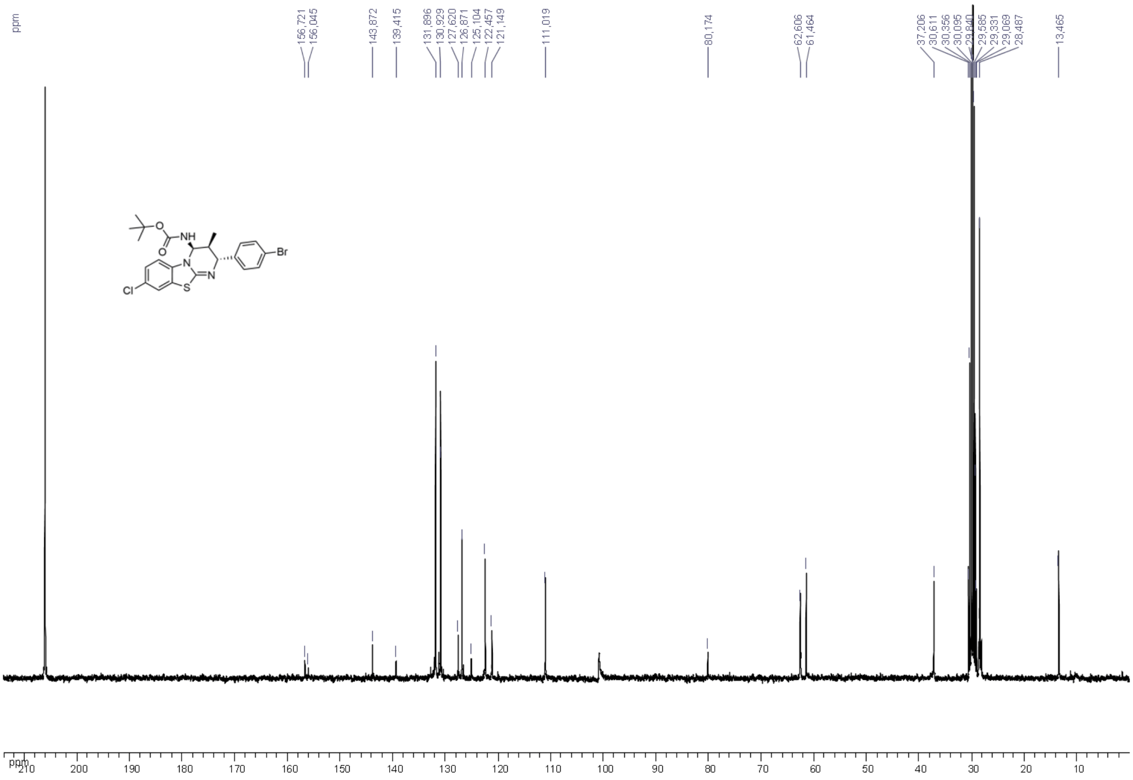

tert-butyl ((2_R_,3_S_,4_R_)-2-(4-bromophenyl)-8-chloro-3-methyl-3,4-dihydro-2_H_-benzo[4,5]thiazolo[3,2-_a_]pyrimidin-4-yl)carbamate

4m

Chiralpak IA, Heptane/EtOH = 95/5, 1 mL/min, 254 nm

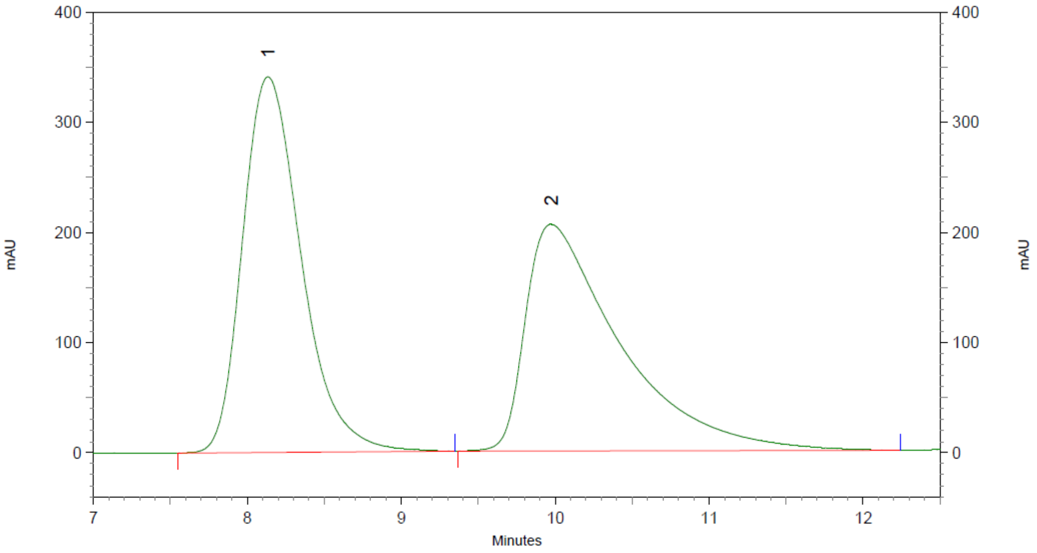

DAD-CH1 254 nm

Results

Pk #	Retention Time	Area	Area %
1	8,13	36099449	50,72
2	9,97	35073086	49,28

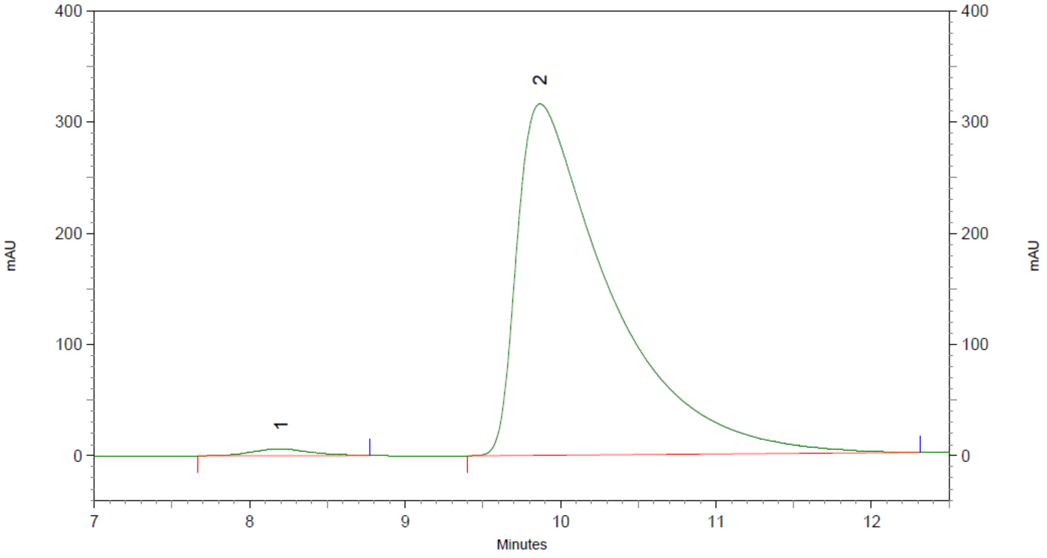

DAD-CH1 254 nm

Results

Pk #	Retention Time	Area	Area %
1	8,20	622507	1,17
2	9,87	52567418	98,83

allyl ((2*R*,3*S*,4*R*)-2-(4-bromophenyl)-8-chloro-3-methyl-3,4-dihydro-2*H*-benzo[4,5]thiazolo[3,2-*a*]pyrimidin-4-yl)carbamate

4n

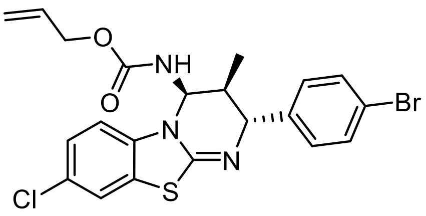

Compound **4n** was prepared according to the general procedure **D** from 2-benzothiazolimine **2f** (35.2 mg, 0.1 mmol) and enecarbamate **3c** (42.3 mg, 0.3 mmol) as starting materials. Purification on a column of silica gel with a gradient of ethyl acetate in heptane (from 9:1 to 85:15) as eluent gave the desired product as only one diastereomer.

Chemical formula	$C_{21}H_{19}BrClN_3O_2S$
M (g.mol^{-1})	492.82
Yield	40.9 mg, 83%
Aspect	White foam
Rf	0.7 (Hept/AcOEt : 7/3)
^{1}H NMR (300 MHz, Acetone-d$_6$)	**δ :** 7.54 (d, *J* = 8.6 Hz, 3H), 7.43 (d, *J* = 1.9 Hz, 1H), 7.26 (dd, *J* = 8.4, 2.5 Hz, 1H), 7.25 (d, *J* = 8.5 Hz, 2H), 7.11 (d, *J* = 8.4 Hz, 1H), 5.95 (ddt, *J* = 17.2, 10.4, 5.3 Hz, 1H), 5.88 (dd, *J* = 10.0, 4.4 Hz, 1H), 5.30 (dq, *J* = 17.3, 1.7 Hz, 1H), 5.18 (dq, *J* = 10.6, 1.6 Hz, 1H), 4.63 (d, *J* = 4.5 Hz, 2H), 4.28 (d, *J* = 11.5 Hz, 1H), 2.20-2.15 (m, 1H), 0.77 (d, *J* = 6.7Hz, 3H)
^{13}C NMR (75 MHz, Acetone-d$_6$)	**δ :** 157.2 (C), 155.9 (C), 143.9 (C), 139.3 (C), 134.0 (CH), 131.9 (2 CH), 130.9 (2 CH), 127.6 (CH), 126.9 (C), 125.0 (C), 122.5 (CH), 121.2 (C), 117.5 (CH_2), 110.9 (CH), 66.7 (CH_2), 63.0 (CH), 61.5 (CH), 37.2 (CH), 13.5 (CH_3)
IR (Neat, cm^{-1})	2965, 1717, 1623, 1572, 1539, 1489, 1471, 1406, 1317, 1247, 1215, 1073, 1009
HRMS (ESI+, m/z)	$[M+H]^+$ calcd. for $C_{21}H_{20}BrClN_3O_2S$ 492.0148, found 492.0146
HPLC Analysis	Daicel Chiralpak OD-H, Hexane/EtOH = 95/5, flow rate = 1 mL/min, 254 nm<br>minor isomer: t_R = 7.39 min , major isomer: t_R = 11.39 min
Enantiomeric excess	98%
$[\alpha]_D^{22}$	-149.00° (*c* 0.3, $CHCl_3$)

allyl ((2*R*,3*S*,4*R*)-2-(4-bromophenyl)-8-chloro-3-methyl-3,4-dihydro-2*H*-benzo[4,5]thiazolo[3,2-*a*]pyrimidin-4-yl)carbamate

4n

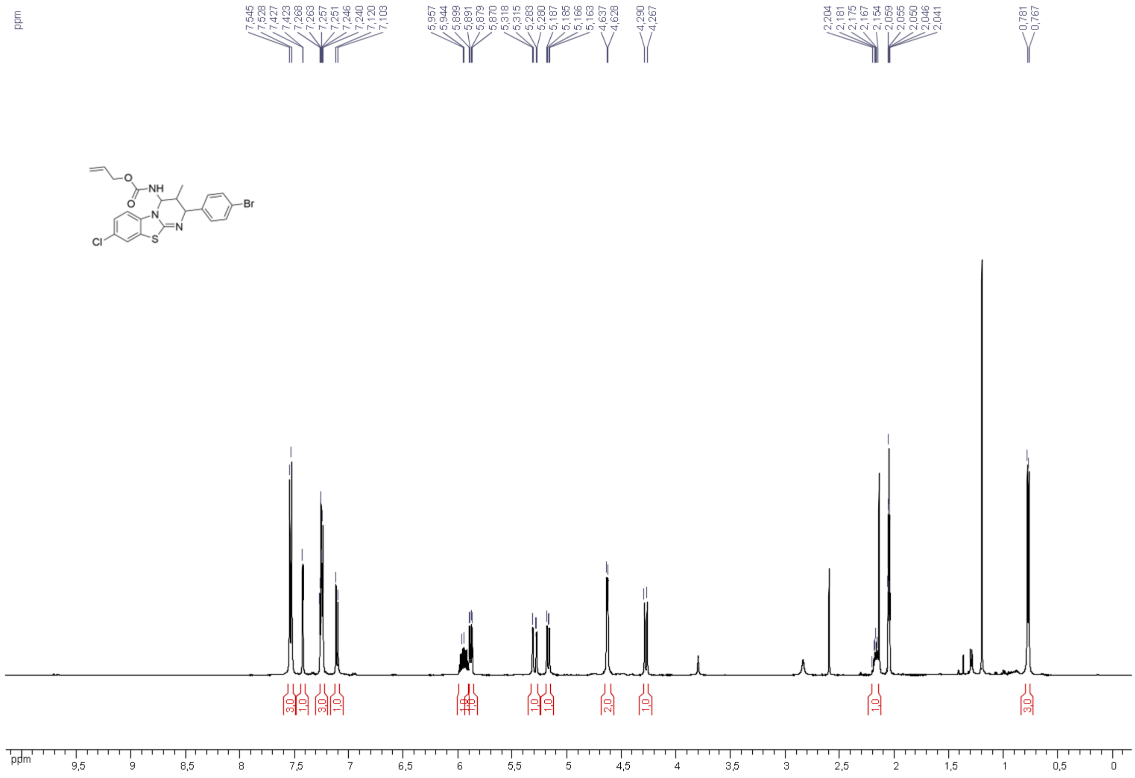

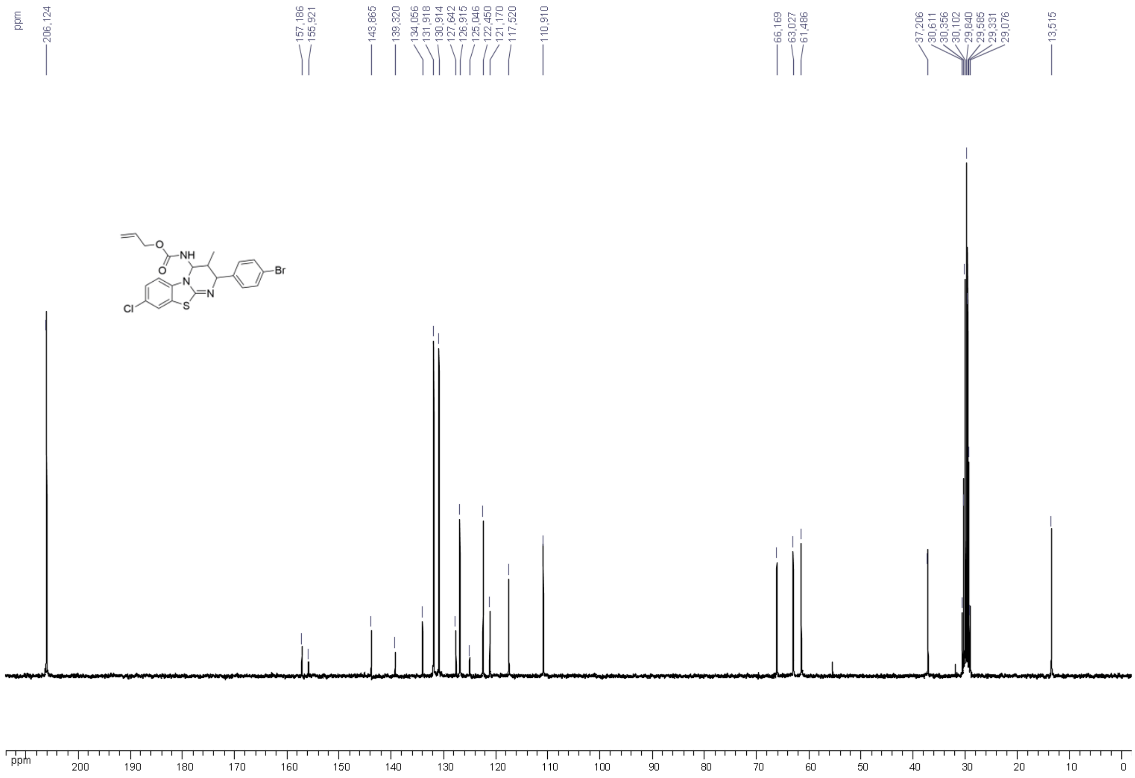

allyl ((2*R*,3*S*,4*R*)-2-(4-bromophenyl)-8-chloro-3-methyl-3,4-dihydro-2*H*-benzo[4,5]thiazolo[3,2-*a*]pyrimidin-4-yl)carbamate
4n

Chiralpak OD-H, Hexane/EtOH = 95/5, 1 mL/min, 254 nm

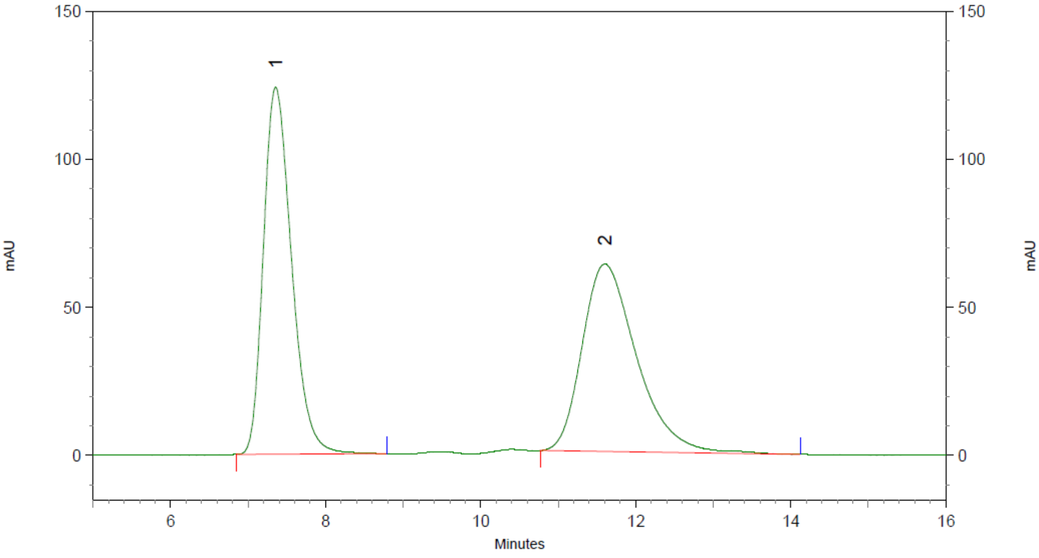

DAD-CH1 254 nm
Results

Pk #	Retention Time	Area	Area %
1	7,35	12514403	50,26
2	11,60	12386079	49,74

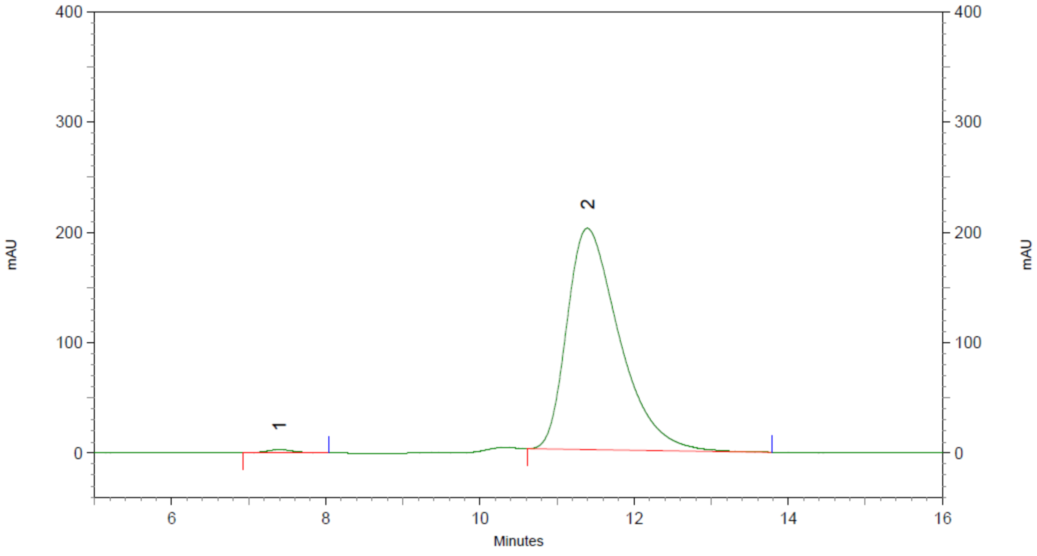

DAD-CH1 254 nm
Results

Pk #	Retention Time	Area	Area %
1	7,39	291826	0,76
2	11,39	37884061	99,24

4-(trifluoromethyl)benzyl ((2*R*,3*S*,4*R*)-2-(4-bromophenyl)-8-chloro-3-methyl-3,4-dihydro-2*H*-benzo[4,5]thiazolo[3,2-*a*]pyrimidin-4-yl)carbamate

4o

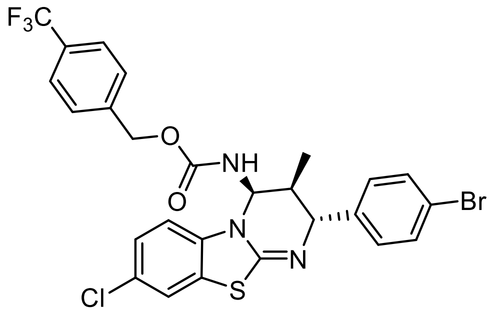

Compound **4o** was prepared according to the general procedure **D** from 2-benzothiazolimine **2f** (35.2 mg, 0.1 mmol) and enecarbamate **3d** (77.8 mg, 0.3 mmol) as starting materials. Purification on a column of silica gel with a gradient of ethyl acetate in heptane (from 85:15 to 8:2) as eluent gave the desired product as only one diastereomer.

Chemical formula	$C_{26}H_{20}BrClF_3N_3O_2S$
M (g.mol^{-1})	610.87
Yield	45.8 mg, 75%
Aspect	Colorless oil
Rf	0.7 (Hept/AcOEt : 7/3)
^{1}H NMR (300 MHz, Acetone-d$_6$)	**δ :** 7.82 (d, *J* = 10.4 Hz, 1H, NH), 7.69 (d, *J* = 8.0 Hz, 2H), 7.60 (d, *J* = 8.0 Hz, 2H), 7.51 (d, *J* = 8.8 Hz, 2H), 7.36 (d, *J* = 2.0 Hz, 1H), 7.22 (dd, *J* = 8.9, 2.2 Hz, 1H), 7.21 (d, *J* = 8.6 Hz, 2H), 7.09 (d, *J* = 8.9 Hz, 1H), 5.90 (dd, *J* = 10.4, 3.8 Hz, 1H), 5.31 (s, 2H), 4.22 (d, *J* = 10.9 Hz, 1H), 2.17-2.10 (m, 1H), 0.74 (d, *J* = 6.9 Hz, 3H)
^{13}C NMR (75 MHz, Acetone-d$_6$)	**δ :** 157.2 (C), 156.2 (C), 143.7 (C), 142.5 (C), 139.2 (C), 131.9 (2 CH), 130.9 (2 CH), 128.9 (2 CH), 127.7 (C), 126.9 (CH), 126.9 (q, *J* = 3,6 Hz, 2 CH), 125.22 (q, *J* = 273.0 Hz, CF_3), 125.17 (q, *J* = 34.0 Hz, C), 125.0 (C), 122.4 (CH), 121.2 (C), 111.0 (CH), 66.4 (CH_2), 63.1 (CH), 61.5 (CH), 37.2 (CH), 13.5 (CH_3)
^{19}F NMR (282 MHz, Acetone-d$_6$)	**δ :** -114.53
IR (Neat, cm^{-1})	3162, 2964, 2934, 1919, 1716, 1621, 1572, 1536, 1489, 1471, 1406, 1384, 1361, 1323, 1291, 1247, 1215, 1166, 1126, 1112, 1094, 1066, 1009
HRMS (ESI+, m/z)	$[M+H]^+$ calcd. for $C_{26}H_{21}BrClF_3N_3O_2S$ 610.0178, found 610.0189
HPLC Analysis	Daicel Chiralpak AD-H, Heptane/EtOH = 90/10, flow rate = 1 mL/min, 254 nm major isomer: t_R = 9.35 min , minor isomer: t_R = 14.02 min
Enantiomeric excess	99%
$[\alpha]_D^{22}$	-3.62° (*c* 3.59, $CHCl_3$)

4-(trifluoromethyl)benzyl ((2*R*,3*S*,4*R*)-2-(4-bromophenyl)-8-chloro-3-methyl-3,4-dihydro-2*H*-benzo[4,5]thiazolo[3,2-*a*]pyrimidin-4-yl)carbamate

4o

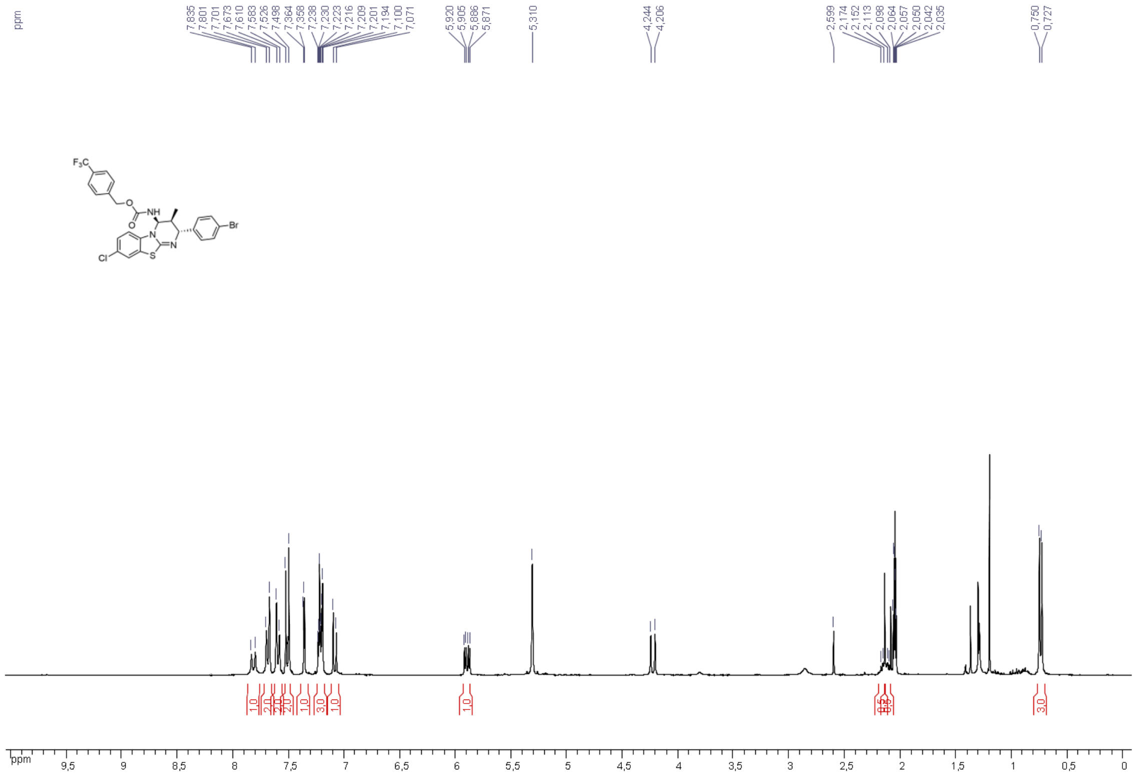

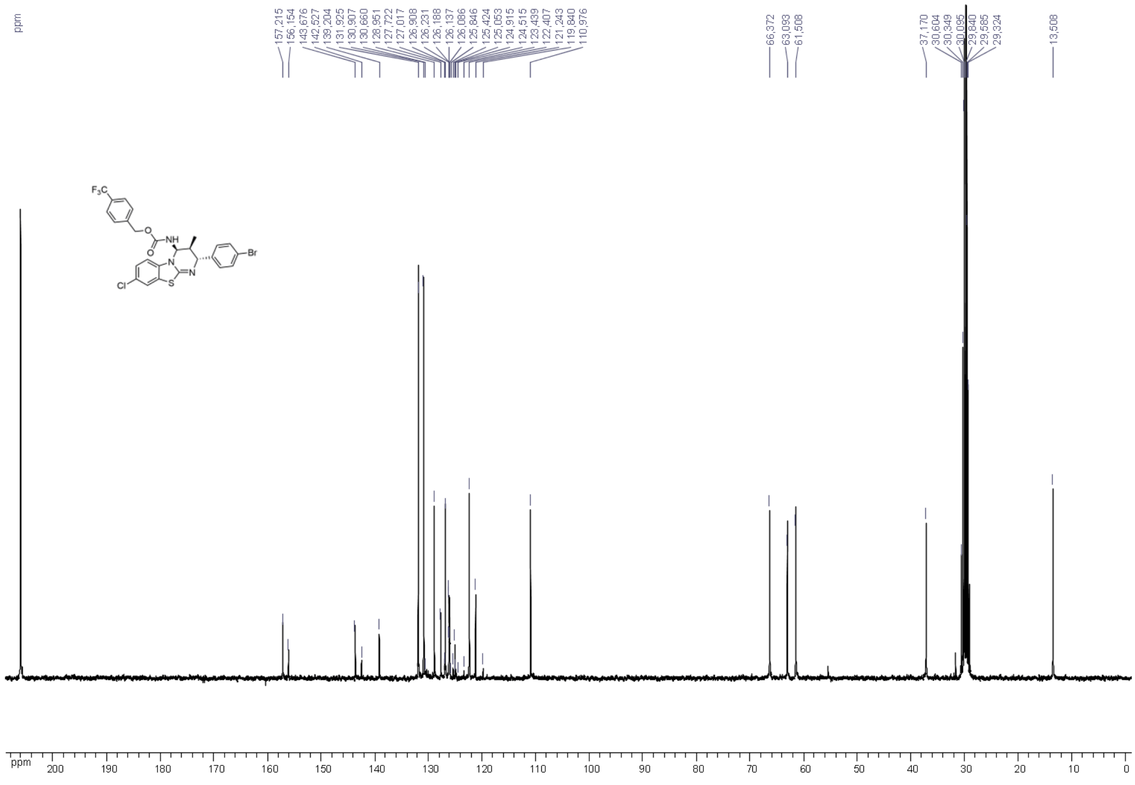

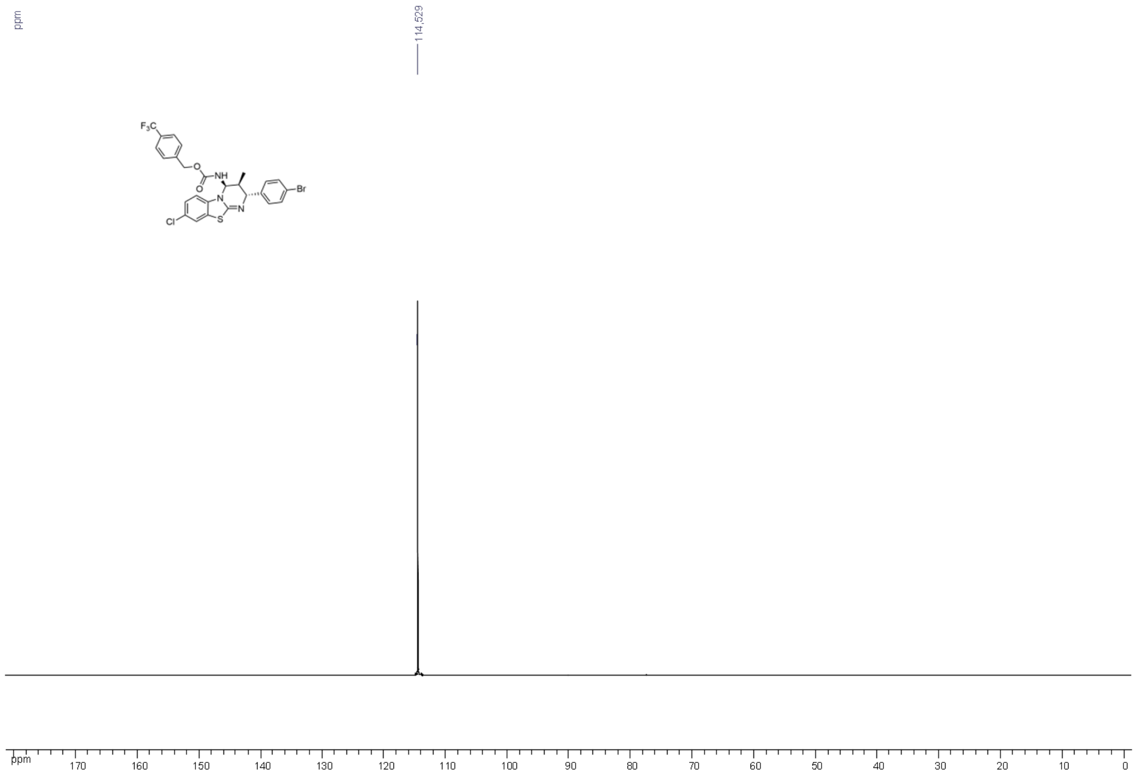
ppm
114.529
F3C
O
NH
O
N
Br
Cl
S
N
ppm
170
160
150
140
130
120
110
100
90
80
70
60
50
40
30
20
10
0

4-(trifluoromethyl)benzyl ((2*R*,3*S*,4*R*)-2-(4-bromophenyl)-8-chloro-3-methyl-3,4-dihydro-2*H*-benzo[4,5]thiazolo[3,2-*a*]pyrimidin-4-yl)carbamate
4o

Chiralpak AD-H, Heptane/EtOH = 90/10, 1 mL/min, 254 nm

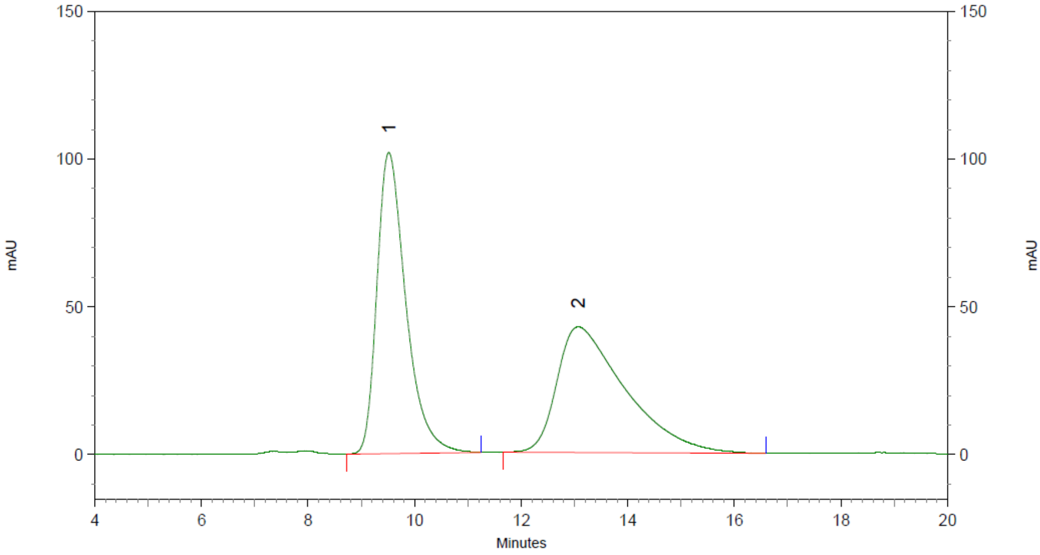

DAD-CH1 254 nm
Results

Pk #	Retention Time	Area	Area %
1	9,51	15366815	50,40
2	13,07	15122006	49,60

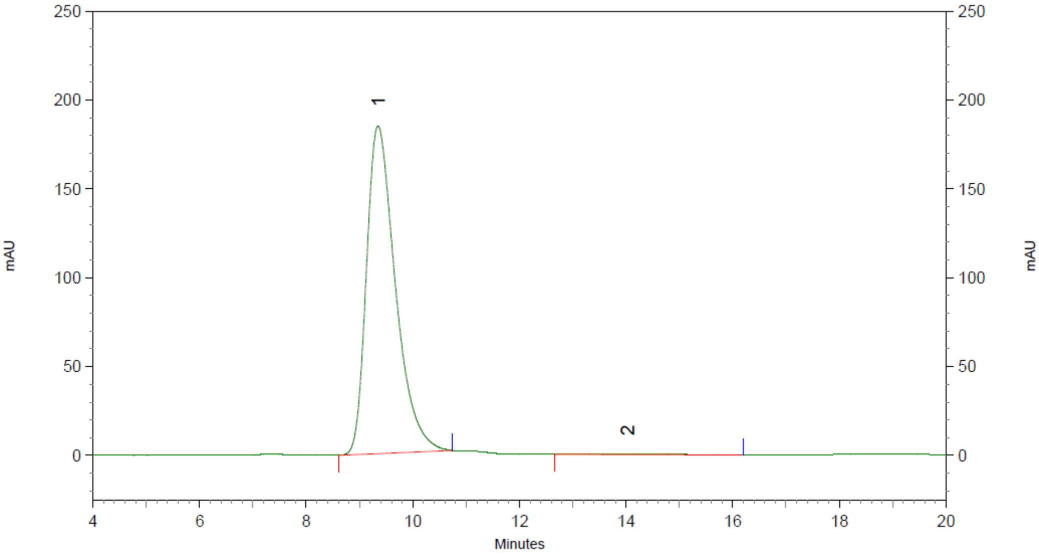

DAD-CH1 254 nm
Results

Pk #	Retention Time	Area	Area %
1	9,35	27768110	99,36
2	14,02	179775	0,64

allyl ((2*R*,3*S*,4*R*)-2-(4-bromophenyl)-8-fluoro-3-methyl-3,4-dihydro-2*H*-benzo[4,5]thiazolo[3,2-*a*]pyrimidin-4-yl)carbamate

4p

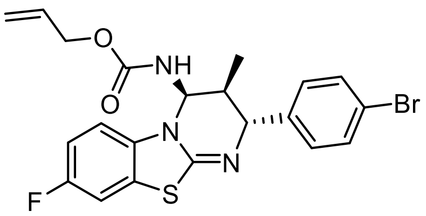

Compound **4p** was prepared according to the general procedure **D** from 2-benzothiazolimine **2d** (33.5 mg, 0.1 mmol) and enecarbamate **3c** (42.3 mg, 0.3 mmol) as starting materials. Purification on a column of silica gel with a gradient of ethyl acetate in heptane (from 9:1 to 8:2) as eluent gave the desired product as only one diastereomer.

Chemical formula	$C_{21}H_{19}BrFN_3O_2S$
M (g.mol^{-1})	476.36
Yield	37.6 mg, 79%
Aspect	White foam
Rf	0.6 (Hept/AcOEt : 7/3)
^{1}H NMR (300 MHz, Acetone-d$_6$)	**δ :** 7.58 (d, *J* = 10.6 Hz, 1H, NH), 7.54 (d, *J* = 8.4, 4Hz, 2H), 7.25 (d, *J* = 8.3 Hz, 2H), 7.23 (dd, *J* = 8.3, 2.1 Hz, 1H), 7.11 (dd, *J* = 8.7, 4.3 Hz, 1H), 7.02 (td, *J* = 8.9, 2.6 Hz, 1H), 5.95 (dp, J = 16.5, 5.0 Hz, 1H), 5.87 (dd, *J* = 9.9, 4.7 Hz, 1H), 5.30 (dq, *J* = 17.5, 1.7 Hz, 1H), 5.17 (dq, *J* = 10.5, 1.6 Hz, 1H), 4.63 (d, *J* = 11.1 Hz, 2H), 4.26 (d, *J* = 11.1 Hz, 1H), 2.19-2.11 (m, 1H), 0.76 (d, *J* = 6.8 Hz, 3H)
^{13}C NMR (75 MHz, Acetone-d$_6$)	**δ :** 159.1 (d, *J* = 280.6 Hz, CF), 157.8 (C), 156.3 (C), 143.9 (C), 136.9 (C), 134.1 (CH), 131.9 (2 CH), 130.9 (2 CH), 124.6 (d, *J* = 10.6 Hz, C), 121.1 (C), 117.5 (CH_2), 113.5 (d, *J* = 24.2 Hz, CH), 110.5 (d, *J* = 7.6 Hz, CH), 110.2 (d, *J* = 28.0 Hz, CH), 66.1 (CH_2), 63.1 (CH), 61.4 (CH), 37.3 (CH), 13.5 (CH_3)
^{19}F NMR (282 MHz, Acetone-d$_6$)	**δ :** -55.11
IR (Neat, cm^{-1})	3155, 2964, 2934, 2773, 1713, 1620, 1591, 1573, 1538, 1481, 1458, 1428, 1405, 1384, 1368, 1340, 1318, 1291, 1254, 1231, 1188, 1127, 1103, 1073, 1010
HRMS (ESI+, m/z)	$[M+H]^+$ calcd. for $C_{21}H_{20}BrFN_3O_2S$ 476.0444, found 476.0422
HPLC Analysis	Daicel Chiralpak OD-H, Hexane/EtOH = 95/5, flow rate = 1 mL/min, 254 nm<br>minor isomer: t_R = 6.87 min, major isomer: t_R = 9.75 min
Enantiomeric excess	98%
$[\alpha]_D^{22}$	-5.91° (*c* 3.51, $CHCl_3$)

allyl ((2*R*,3*S*,4*R*)-2-(4-bromophenyl)-8-fluoro-3-methyl-3,4-dihydro-2*H*-benzo[4,5]thiazolo[3,2-*a*]pyrimidin-4-yl)carbamate

4p

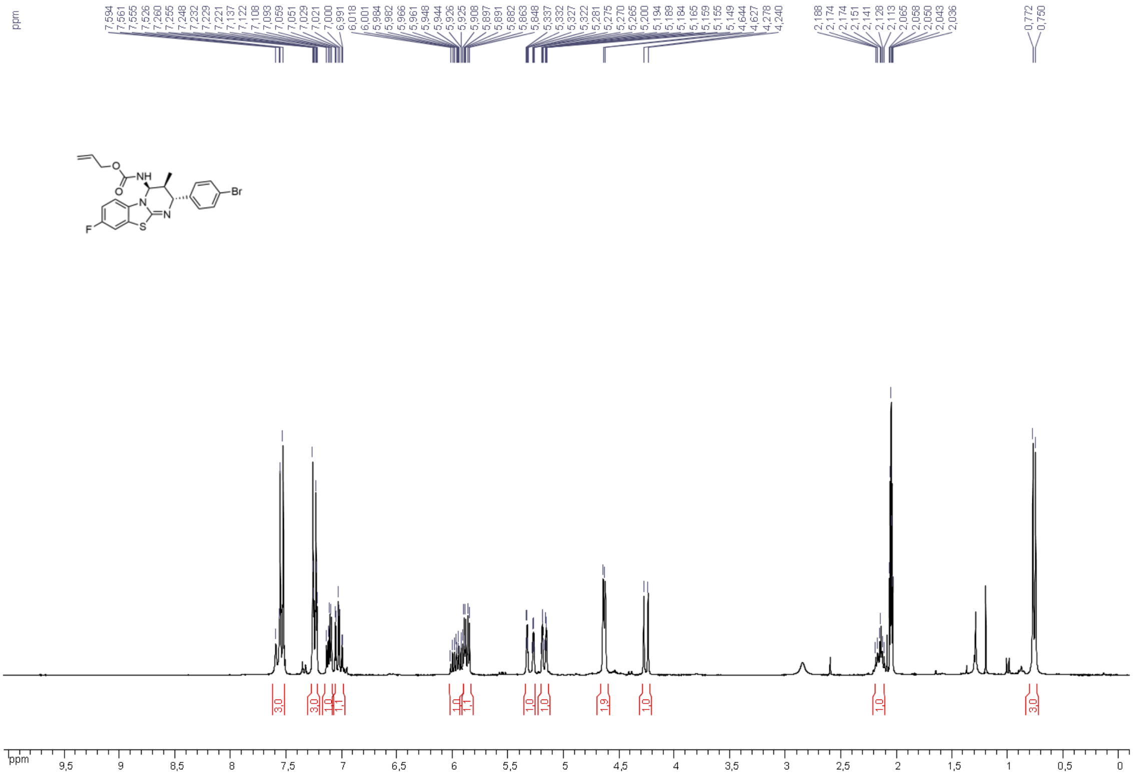

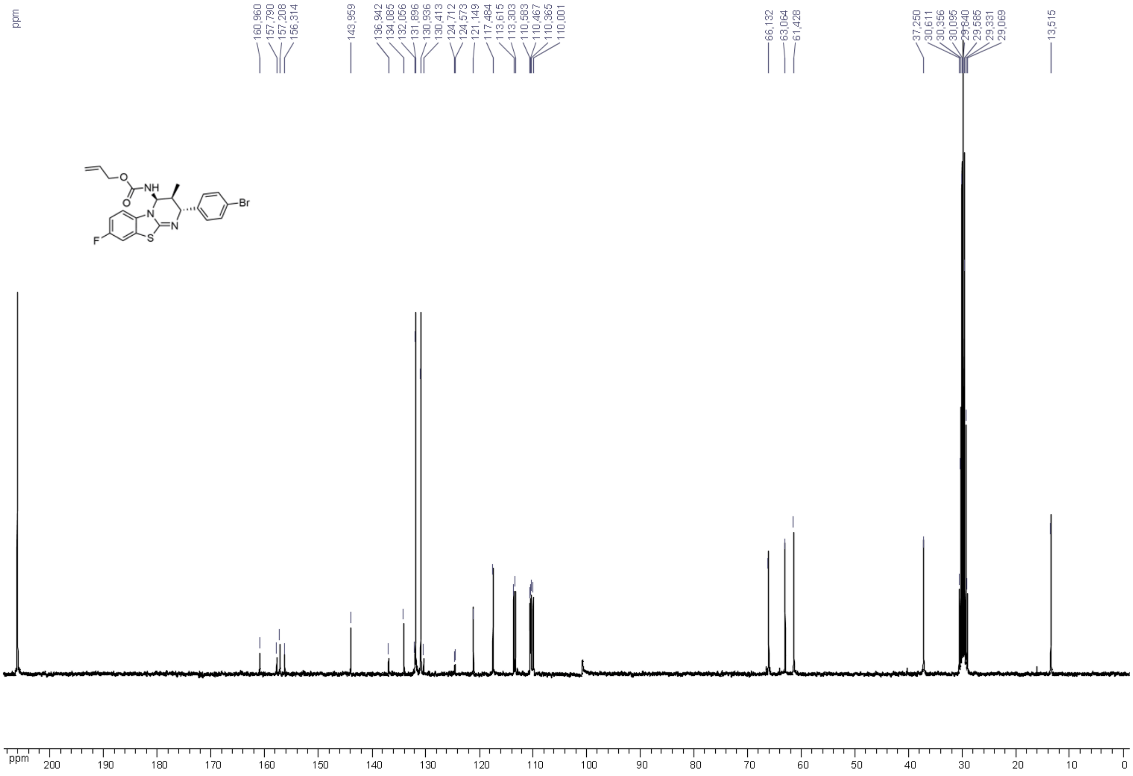

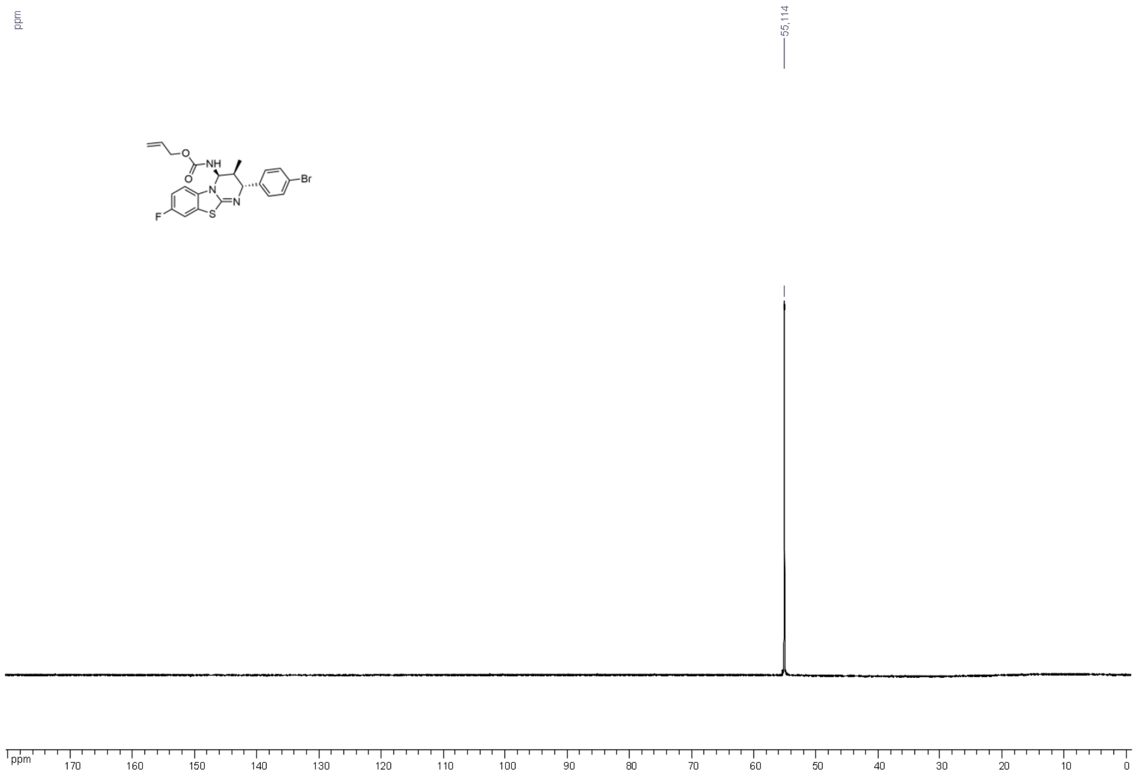

ppm
55.114
NH
O
N
N
S
F
Br
ppm
170
160
150
140
130
120
110
100
90
80
70
60
50
40
30
20
10
0

allyl ((2*R*,3*S*,4*R*)-2-(4-bromophenyl)-8-fluoro-3-methyl-3,4-dihydro-2*H*-benzo[4,5]thiazolo[3,2-*a*]pyrimidin-4-yl)carbamate
4p

Chiralpak OD-H, Hexane/EtOH = 95/5, 1 mL/min, 254 nm

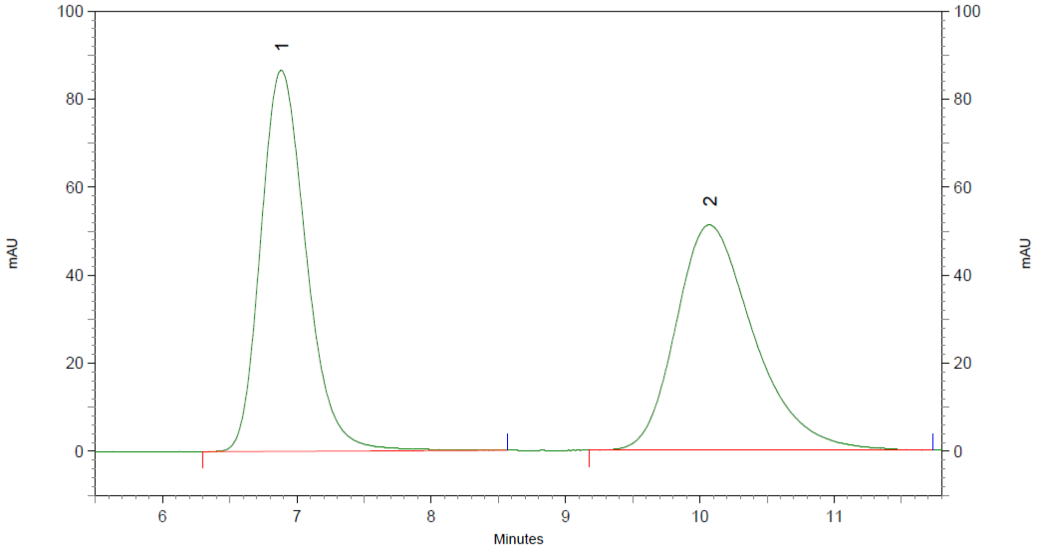

DAD-CH1 254 nm
Results

Pk #	Retention Time	Area	Area %
1	6,89	8137161	49,54
2	10,07	8289743	50,46

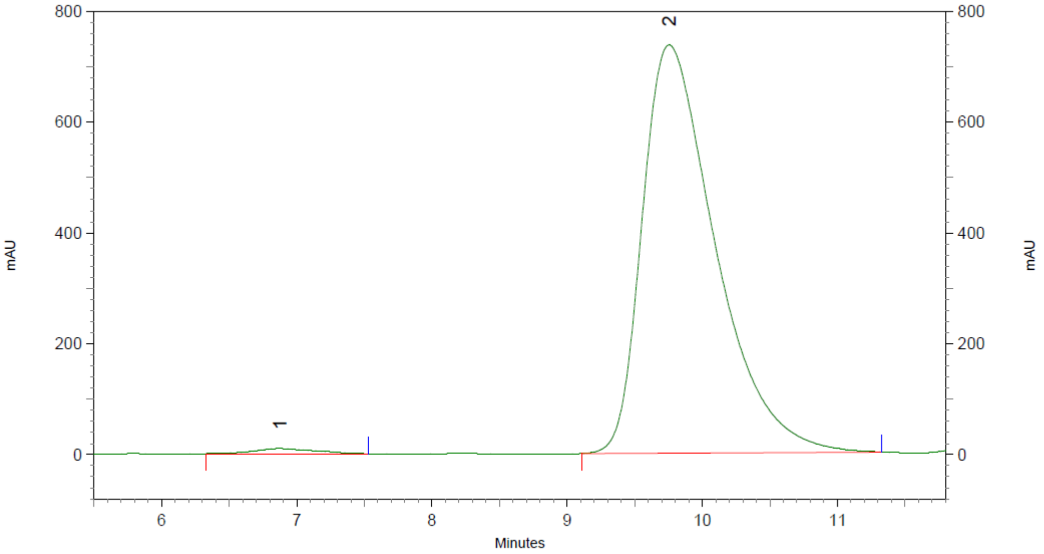

DAD-CH1 254 nm
Results

Pk #	Retention Time	Area	Area %
1	6,87	1213243	1,09
2	9,75	110481960	98,91

tert-butyl ((2R,3S,4R)-2-(4-bromophenyl)-3,8-dimethyl-3,4-dihydro-2H-benzo[4,5]thiazolo[3,2-a]pyrimidin-4-yl)carbamate

4q

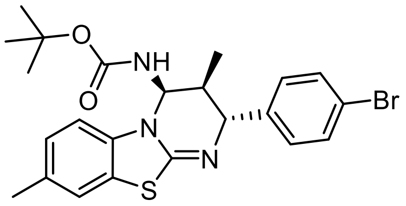

Compound **4q** was prepared according to the general procedure **D** from 2-benzothiazolimine **2b** (33.1 mg, 0.1 mmol) and enecarbamate **3b** (47.2 mg, 0.3 mmol) as starting materials. Purification on a column of silica gel with a gradient of ethyl acetate in heptane (from 9:1 to 85:15) as eluent gave the desired product as only one diastereomer.

Chemical formula	$C_{23}H_{26}BrN_3O_2S$
M (g.mol^{-1})	488.44
Yield	40.5 mg, 83%
Aspect	Colorless oil
Rf	0.6 (Hept/AcOEt : 7/3)
^{1}H NMR (300 MHz, Acetone-d$_6$)	**δ :** 7.52 (d, *J* = 8.3 Hz, 2H), 7.25 (d, *J* = 8.6 Hz, 2H), 7.18 (s, 1H), 7.13-7.06 (m, 1H, NH), 7.04 (s, 2H), 5.79 (dd, *J* = 10.0, 4.3 Hz, 1H), 4.25 (d, *J* = 11.4 Hz, 1H), 2.31 (s, 3H), 2.14-2.07 (m, 1H), 1.45 (s, 9H), 0.79 (d, *J* = 8.2 Hz, 3H)
^{13}C NMR (75 MHz, Acetone-d$_6$)	**δ :** 156.7 (2 C), 144.4 (C), 138.4 (C), 132.6 (C), 131.8 (2 CH), 131.0 (2 CH), 127.4 (CH), 123.0 (C and CH), 121.0 (C), 109.7 (CH), 79.9 (C), 62.4 (CH), 61.6 (CH), 37.4 (CH), 28.5 (3 CH_3), 20.9 (CH_3), 13.5 (CH_3)
IR (Neat, cm^{-1})	3163, 2976, 2932, 1706, 1616, 1584, 1572, 1540, 1486, 1455, 1405, 1391, 1366, 1338, 1319, 1286, 1267, 1251, 1228, 1200, 1159, 1122, 1108, 1068, 1009
HRMS (ESI+, m/z)	$[M+H]^+$ calcd. for $C_{23}H_{27}BrN_3O_2S$ 488.1007, found 488.0985
HPLC Analysis	Daicel Chiralpak AD-H, Heptane/*i*PrOH = 90/10, flow rate = 1 mL/min, 254 nm major isomer: t_R = 5.01 min , minor isomer: t_R = 7.80 min
Enantiomeric excess	97%
$[\alpha]_D^{22}$	-5.05° (*c* 4.27, $CHCl_3$)

tert-butyl ((2_R_,3_S_,4_R_)-2-(4-bromophenyl)-3,8-dimethyl-3,4-dihydro-2_H_-benzo[4,5]thiazolo[3,2-_a_]pyrimidin-4-yl)carbamate

4q

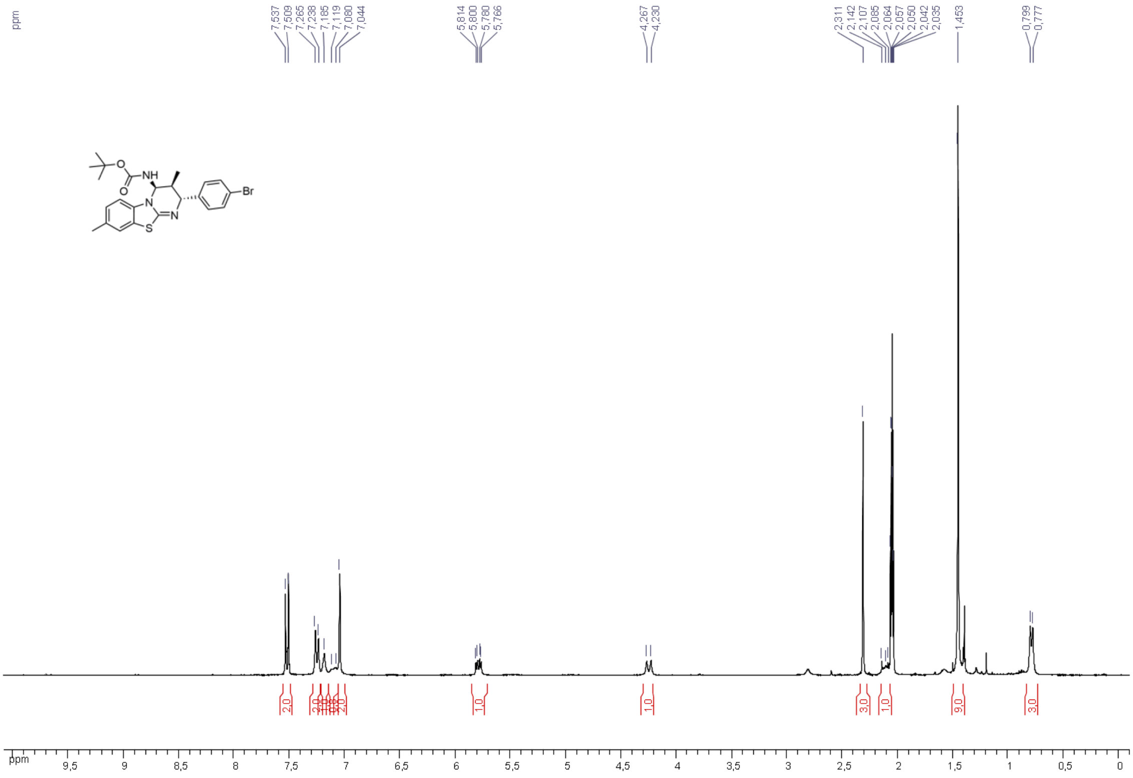

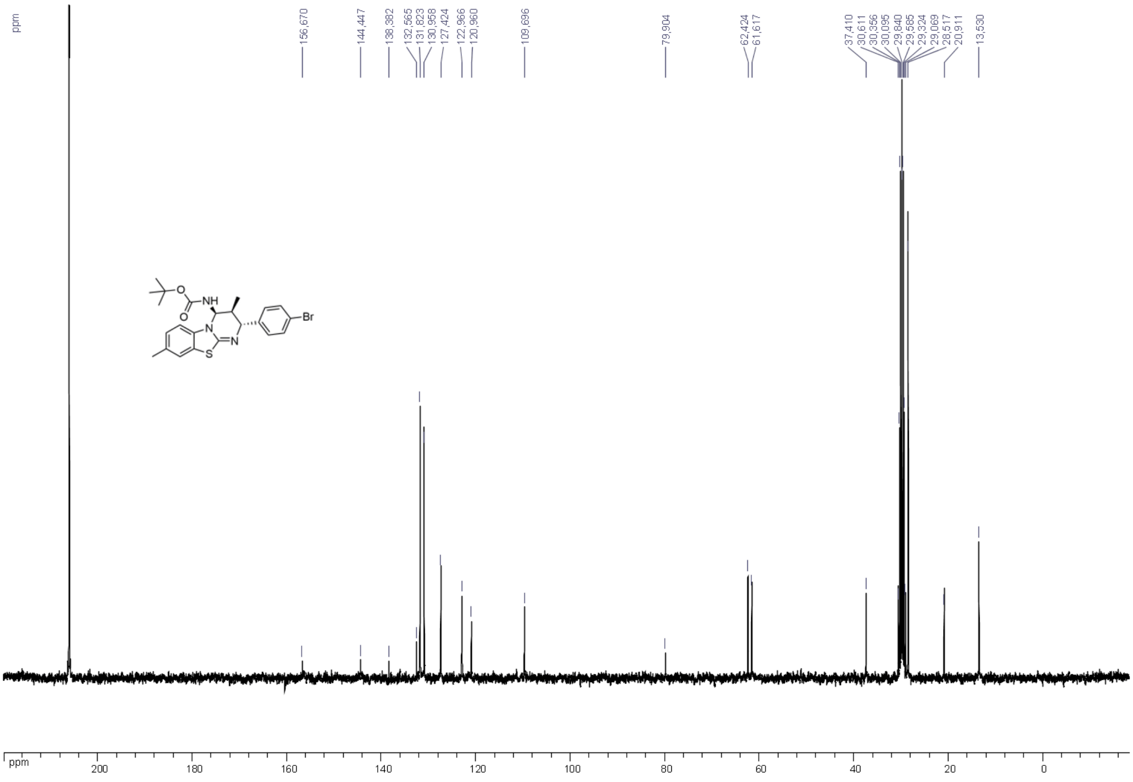

tert-butyl ((2_R_,3_S_,4_R_)-2-(4-bromophenyl)-3,8-dimethyl-3,4-dihydro-2_H_-benzo[4,5]thiazolo[3,2-_a_]pyrimidin-4-yl)carbamate
4q

Chiralpak AD-H, Heptane/_i_PrOH = 90/10, 1 mL/min, 254 nm

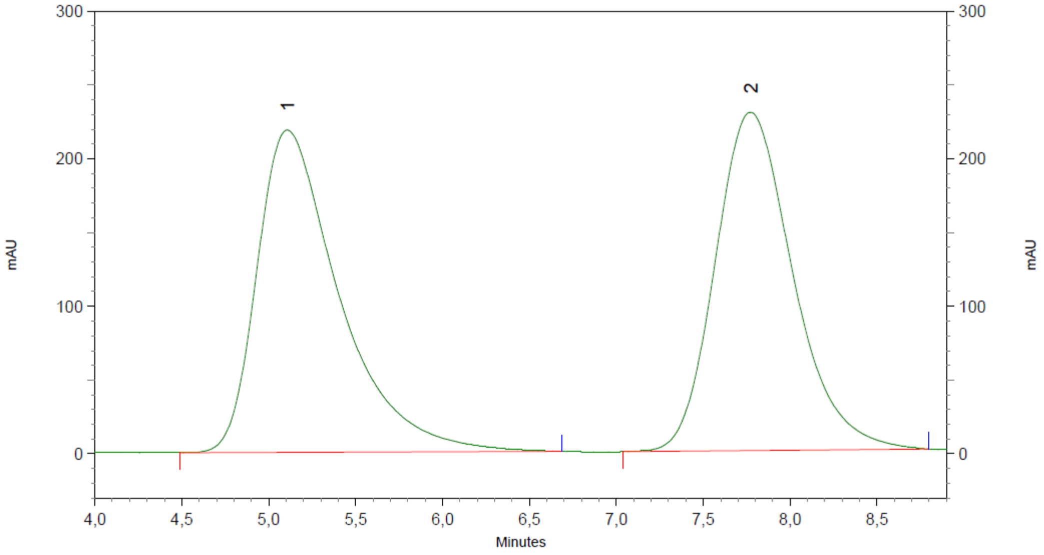

DAD-CH1 254 nm
Results

Pk #	Retention Time	Area	Area %
1	5,11	28788100	50,27
2	7,77	28481071	49,73

0.1 mmol scale:

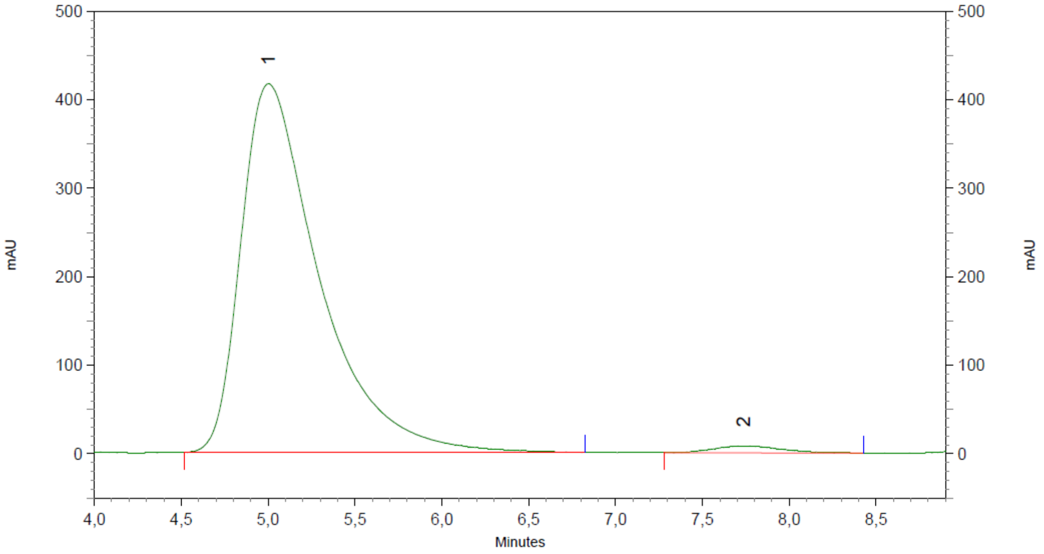

DAD-CH1 254 nm
Results

Pk #	Retention Time	Area	Area %
1	5,00	52173776	98,39
2	7,73	854234	1,61

0.3 mmol scale:

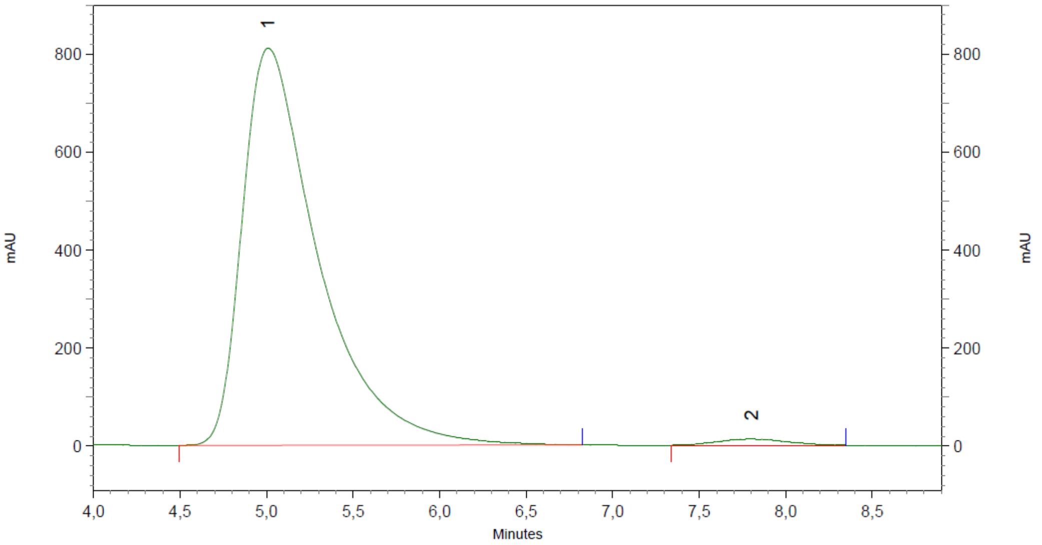

DAD-CH1 254 nm

Results

Pk #	Retention Time	Area	Area %
1	5,01	98995405	98,56
2	7,80	1447483	1,44

benzyl ((2*R*,3*S*,4*R*)-2-(4-bromophenyl)-8-chloro-3-propyl-3,4-dihydro-2*H*-benzo[4,5]thiazolo[3,2-*a*]pyrimidin-4-yl)carbamate

4r

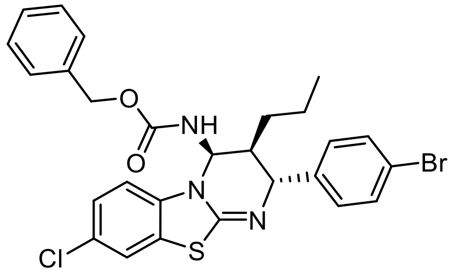

Compound **4r** was prepared according to the general procedure **D** from 2-benzothiazolimine **2f** (35.2 mg, 0.1 mmol) and enecarbamate **3e** (65.8 mg, 0.3 mmol) as starting materials. Purification on a column of silica gel with a gradient of ethyl acetate in heptane (from 9:1 to 85:15) as eluent gave the desired product as only one diastereomer.

Chemical formula	$C_{27}H_{25}BrClN_3O_2S$
M (g.mol^{-1})	570.93
Yield	52.4 mg, 92%
Aspect	White foam
Rf	0.8 (Hept/AcOEt : 7/3)
^{1}H NMR (300 MHz, Acetone-d$_6$)	**δ :** 7.87 (d, *J* = 10.5 Hz, 1H, NH), 7.47 (d, *J* = 8.3 Hz, 2H), 7.39-7.32 (m, 5H), 7.29 (d, *J* = 8.4 Hz, 1H), 7.29 (d, *J* = 1.9 Hz, 1H), 7.24 (dd, *J* = 8.6, 2.2 Hz, 1H), 7.2 (d, *J* = 8.6 Hz, 2H), 5.97 (dd, *J* = 10.1, 4.3 Hz, 1H), 5.26 (d, *J* = 11.9 Hz, 1H), 5.13 (d, *J* = 11.9 Hz, 1H), 4.4 (d, *J* = 11.5 Hz, 1H), 2.02-1.95 (m, 1H), 1.44-1.37 (m, 1H), 1.07-0.99 (m, 2H), 0.92-0.86 (m, 1H), 0.69 (t, *J* = 7.0 Hz, 3H)
^{13}C NMR (75 MHz, Acetone-d$_6$)	**δ :** 157.4 (C), 156.1 (C), 143.9 (C), 139.3 (C), 137.9 (C), 131.9 (2 CH), 131.2 (2 CH), 129.3 (2 CH), 128.9 (CH), 128.8 (2 CH), 127.6 (C), 126.9 (CH), 125.1 (C), 122.2 (CH), 121.3 (C), 111.5 (CH), 67.3 (CH_2), 61.1 (CH), 61.0 (CH), 41.8 (CH), 30.8 (CH_2), 20.0 (CH_2), 14.4 (CH_3)
IR (Neat, cm^{-1})	3146, 2958, 2872, 1710, 1627, 1573, 1542, 1489, 1470, 1407, 1377, 1331, 1316, 1245, 1213, 1099, 1072, 1012
HRMS (ESI+, m/z)	$[M+H]^+$ calcd. for $C_{27}H_{26}BrClN_3O_2S$ 570.0618, found 570.0635
HPLC Analysis	Daicel Chiralpak IA, Heptane/EtOH = 95/5, flow rate = 1 mL/min, 254 nm<br>major isomer: t_R = 12.50 min, minor isomer: t_R = 18.25 min
Enantiomeric excess	98%
$[\alpha]_D^{22}$	-140.33° (*c* 0.3, $CHCl_3$)

benzyl ((2*R*,3*S*,4*R*)-2-(4-bromophenyl)-8-chloro-3-propyl-3,4-dihydro-2*H*-benzo[4,5]thiazolo[3,2-*a*]pyrimidin-4-yl)carbamate

4r

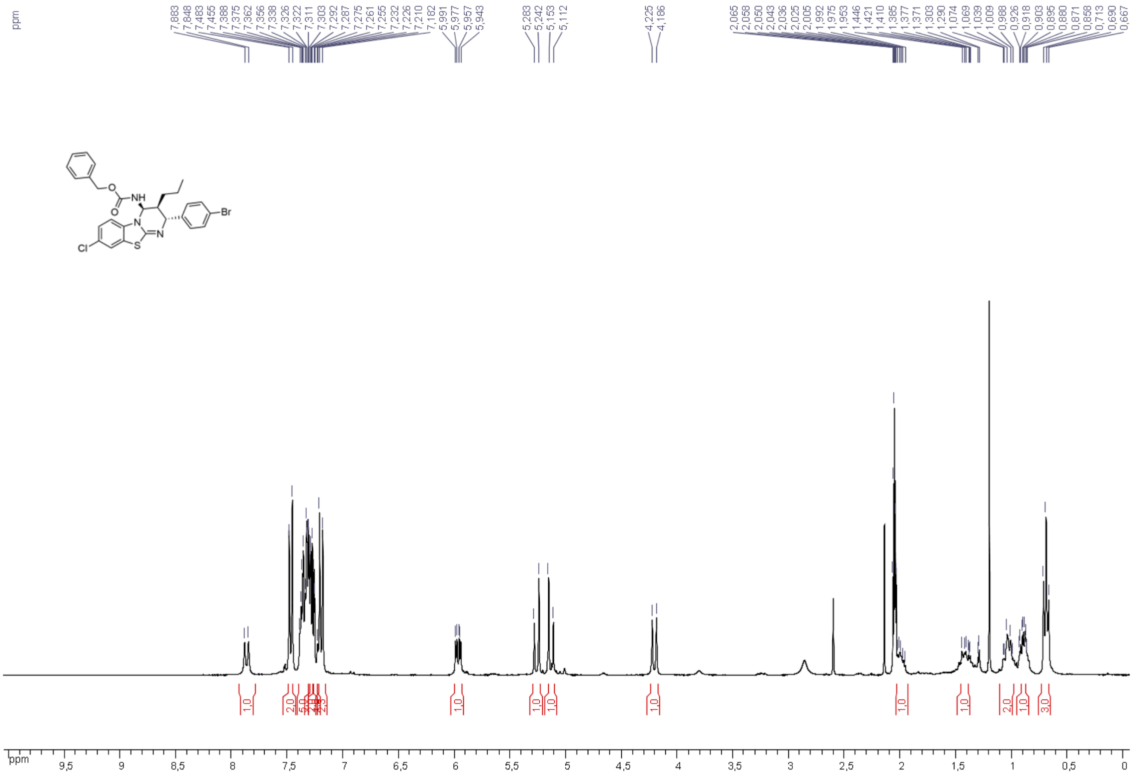

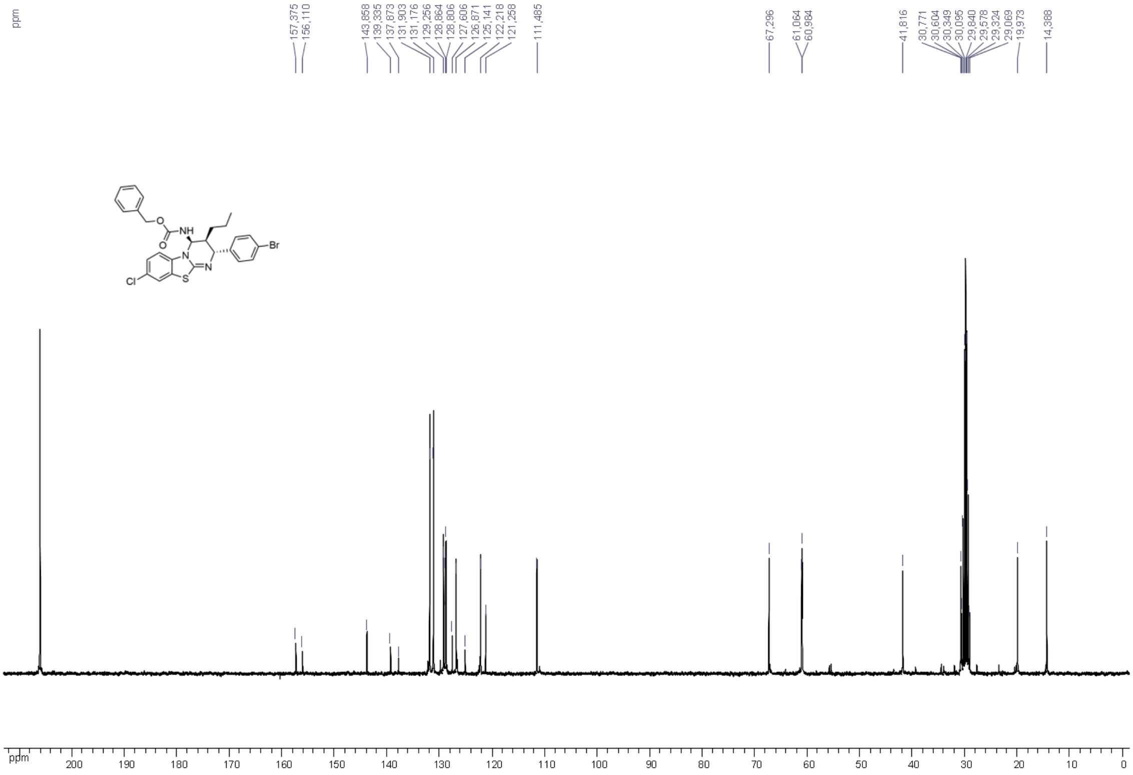

benzyl ((2*R*,3*S*,4*R*)-2-(4-bromophenyl)-8-chloro-3-propyl-3,4-dihydro-2*H*-benzo[4,5]thiazolo[3,2-*a*]pyrimidin-4-yl)carbamate
4r

Chiralpak IA, Heptane/EtOH = 95/5, 1 mL/min, 254 nm

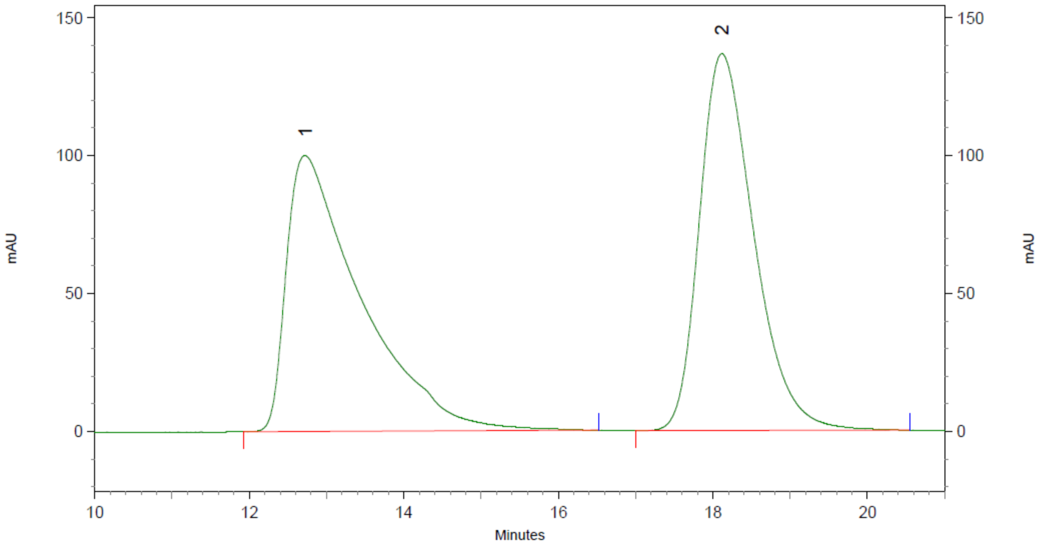

DAD-CH1 254 nm
Results

Pk #	Retention Time	Area	Area %
1	12,72	26887984	50,12
2	18,11	26753981	49,88

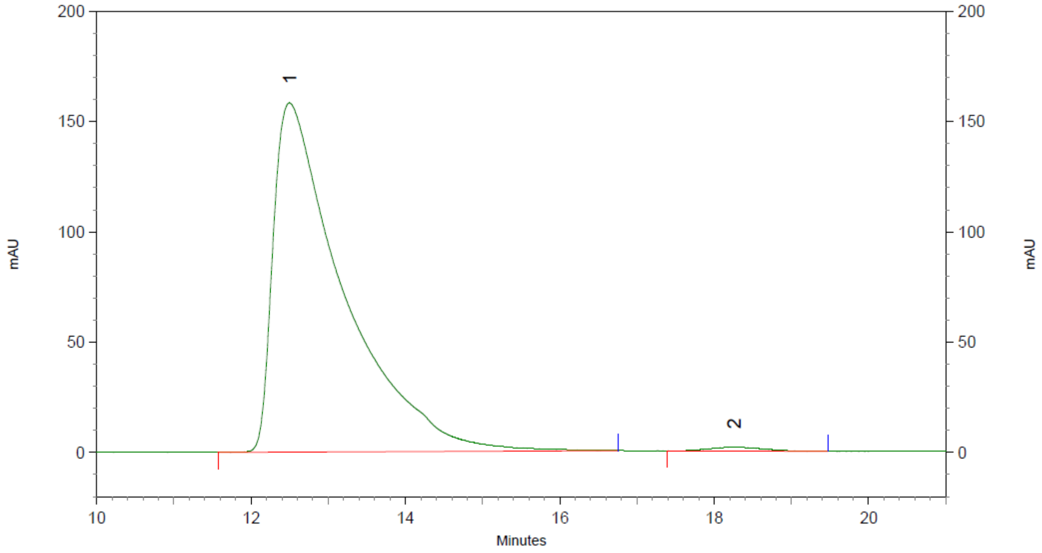

DAD-CH1 254 nm
Results

Pk #	Retention Time	Area	Area %
1	12,50	40002868	99,13
2	18,25	349897	0,87

benzyl ((2*R*,3*S*,4*R*)-2-(4-bromophenyl)-8-chloro-3-cyclopropyl-3,4-dihydro-2*H*-benzo[4,5]thiazolo[3,2-*a*]pyrimidin-4-yl)carbamate

4s

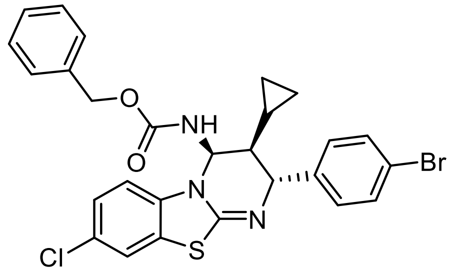

Compound **4s** was prepared according to the general procedure **D** from 2-benzothiazolimine **2f** (35.2 mg, 0.1 mmol) and enecarbamate **3f** (65.2 mg, 0.3 mmol) as starting materials. Purification on a column of silica gel with a gradient of ethyl acetate in heptane (from 9:1 to 75:25) as eluent gave the desired product as only one diastereomer.

Chemical formula	$C_{27}H_{23}BrClN_3O_2S$
M (g.mol^{-1})	568.91
Yield	42.0 mg, 74%
Aspect	Colorless oil
Rf	0.7 (Hept/AcOEt : 7/3)
^{1}H NMR (300 MHz, Acetone-d$_6$)	**δ :** 7.81 (d, *J* = 10.7 Hz, 1H, NH), 7.44 (d, *J* = 8.6 Hz, 2H), 7.38 (m, 6H), 7.24 (d, *J* = 1.0 Hz, 2H), 7.20 (d, *J* = 8.4 Hz, 2H), 5.92 (dd, *J* = 10.4, 4.5 Hz, 1H), 5.23 (d, *J* = 12.4 Hz, 1H), 5.14 (d, *J* = 12.4 Hz, 1H), 4.50 (d, *J* = 11.7 Hz, 1H), 1.18-1.12 (m, 1H), 0.48-0.40 (m, 1H), 0.33-0.18 (m, 2H), 0.04- -0.03 (m, 1H), -0.72- -0.77 (m, 1H)
^{13}C NMR (75 MHz, Acetone-d$_6$)	**δ :** 157.2 (C), 156.3 (C), 144.1 (C), 139.3 (C), 137.9 (C), 131.4 (2 CH), 131.2 (2 CH), 129.2 (2 CH), 128.8 (CH), 128.7 (2 CH), 127.6 (C), 126.9 (CH), 125.1 (C), 122.3 (CH), 120.9 (C), 111.4 (CH), 67.2 (CH_2), 62.6 (CH), 60.9 (CH), 48.2 (CH), 10.3 (CH), 6.8 (CH_2), 2.9 (CH_2)
IR (Neat, cm^{-1})	3160, 3006, 2959, 2926, 1711, 1621, 1591, 1573, 1538, 1488, 1469, 1405, 1360, 1331, 1315, 1293, 1243, 1211, 1104, 1069, 1051, 1026, 1009
HRMS (ESI+, m/z)	$[M+H]^+$ calcd. for $C_{27}H_{24}BrClN_3O_2S$ 568.0461, found 568.0471
HPLC Analysis	Daicel Chiralpak IA, Heptane/EtOH = 95/5, flow rate = 1 mL/min, 254 nm<br>major isomer: t_R = 18.68 min, minor isomer: t_R = 24.45 min
Enantiomeric excess	98%
$[\alpha]_D^{22}$	-5.47° (*c* 4.10, $CHCl_3$)

benzyl ((2*R*,3*S*,4*R*)-2-(4-bromophenyl)-8-chloro-3-cyclopropyl-3,4-dihydro-2*H*-benzo[4,5]thiazolo[3,2-*a*]pyrimidin-4-yl)carbamate

4s

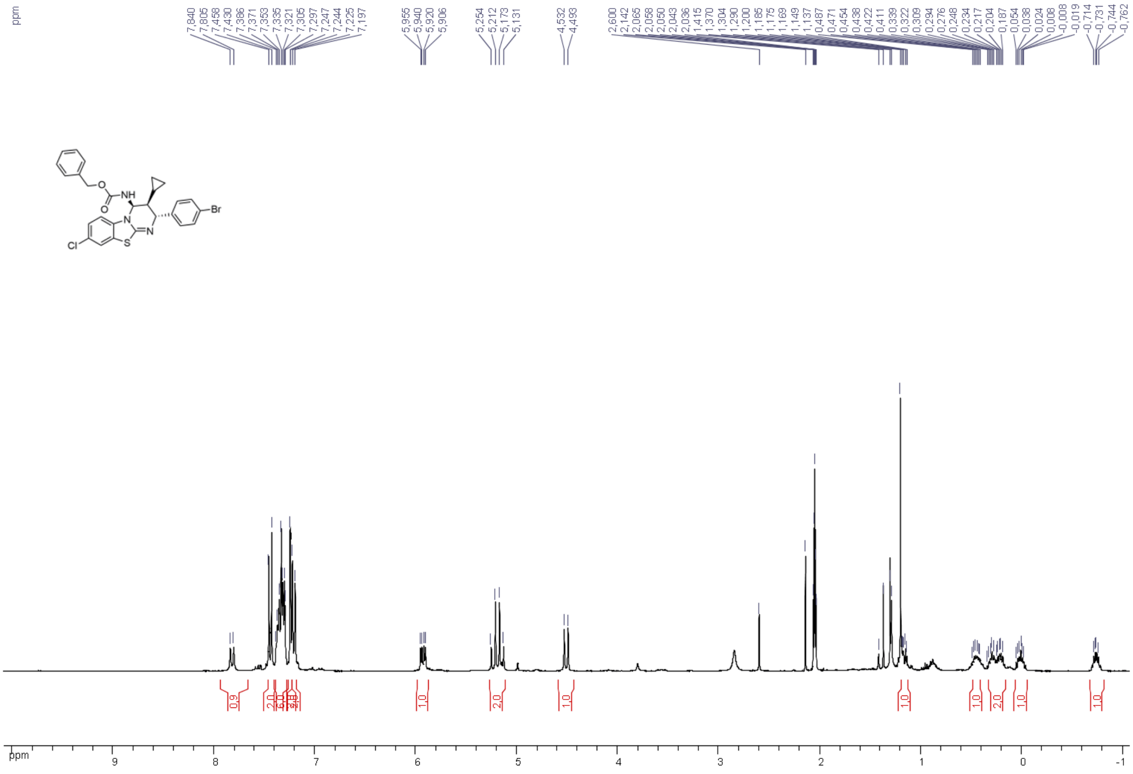

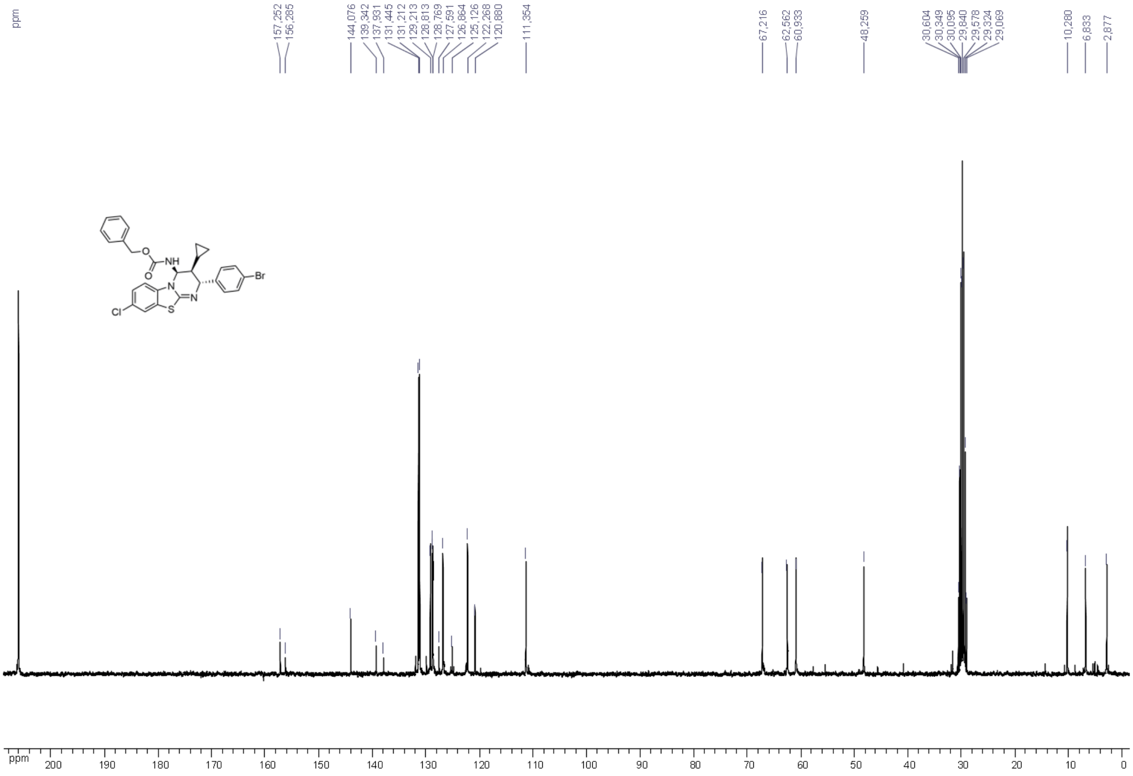

benzyl ((2*R*,3*S*,4*R*)-2-(4-bromophenyl)-8-chloro-3-cyclopropyl-3,4-dihydro-2*H*-benzo[4,5]thiazolo[3,2-*a*]pyrimidin-4-yl)carbamate
4s

Chiralpak IA, Heptane/EtOH = 95/5, 1 mL/min, 254 nm

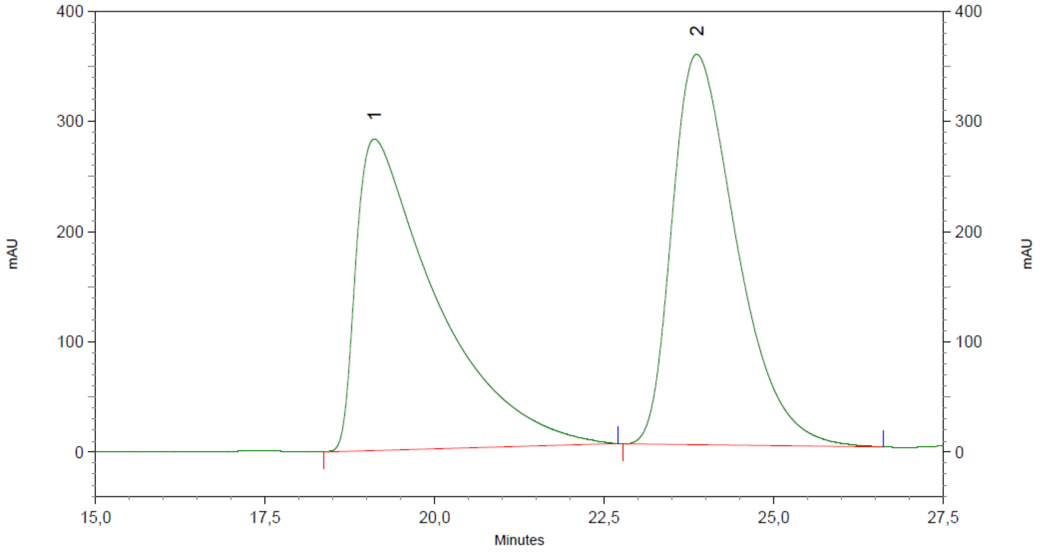

DAD-CH4 220 nm
Results

Pk #	Retention Time	Area	Area %
1	19,11	93079245	49,84
2	23,87	93668709	50,16

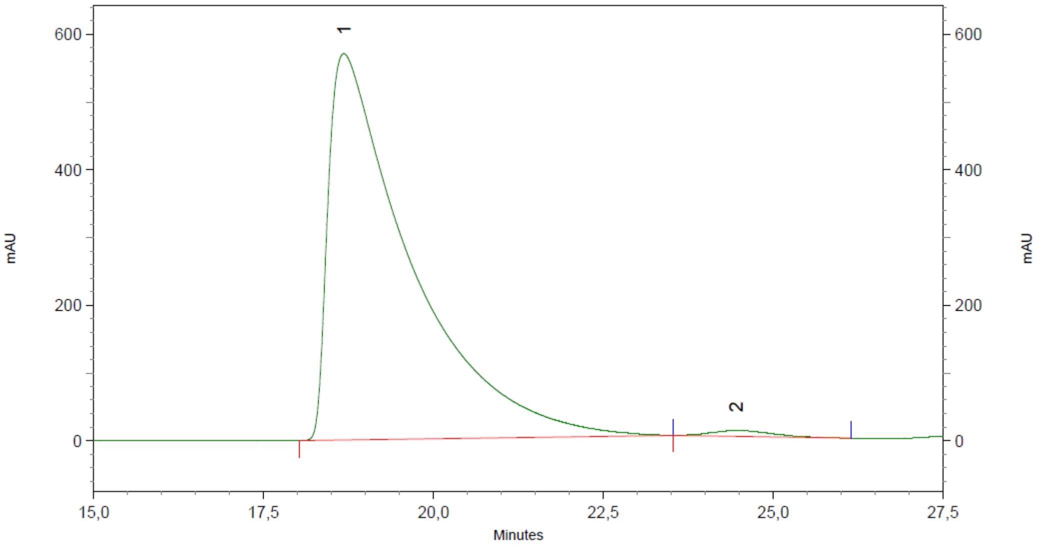

DAD-CH4 220 nm
Results

Pk #	Retention Time	Area	Area %
1	18,68	191893889	98,92
2	24,45	2095356	1,08

benzyl ((2*R*,3*S*,4*R*)-2-(4-bromophenyl)-3-(3-((*tert*-butyldiphenylsilyl)oxy)propyl)-8-chloro-3,4-dihydro-2*H*-benzo[4,5]thiazolo[3,2-*a*]pyrimidin-4-yl)carbamate

4t

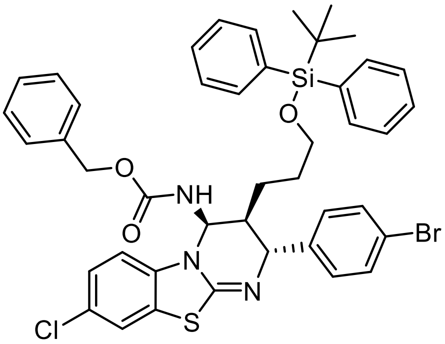

Compound **4t** was prepared according to the general procedure **D** from 2-benzothiazolimine **2f** (35.2 mg, 0.1 mmol) and enecarbamate **3g** (142.0 mg, 0.3 mmol) as starting materials. Purification on a column of silica gel with a gradient of ethyl acetate in heptane (from 9:1 to 85:15) as eluent gave the desired product as only one diastereomer.

Chemical formula	$C_{43}H_{43}BrClN_3O_3SSi$
M (g.mol^{-1})	825.33
Yield	48.0 mg, 58%
Aspect	White foam
Rf	0.7 (Hept/AcOEt : 7/3)
^{1}H NMR (300 MHz, Acetone-d$_6$)	**δ :** 7.73-7.56 (m, 6H), 7.50 (d, *J* = 8.2 Hz, 3H), 7.46-7.39 (m, 6H), 7.36-7.28 (m, 4H), 7.25 (d, *J* = 8.6 Hz, 4H), 6.05 (d, *J* = 10.5, 4.3 Hz, 1H), 5.19 (d, *J* = 12.8 Hz, 1H), 5.14 (d, *J* = 12.8 Hz, 1H), 4.4 (d, *J* = 11.8 Hz, 1H), 3.64-3.45 (m, 2H), 2.28-2.15 (m, 1H), 1.78-1.70 (m, 1H), 1.48-1.42 (m, 1H), 1.42-1.33 (m, 2H), 0.93 (s, 9H)
^{13}C NMR (75 MHz, Acetone-d$_6$)	**δ :** 157.4 (C), 155.6 (C), 144.1 (C), 139.4 (C), 137.8 (C), 136.3 (2 CH), 136.2 (2 CH), 134.5 (C), 132.0 (2 CH), 131.0 (2 CH), 130.6 (2 CH), 130.5 (2 CH), 129.2 (CH), 128.9 (CH), 128.8 (C or CH), 128.7 (CH or C), 128.6 (4 CH), 127.3 (C), 126.8 (CH), 125.1 (C), 122.5 (CH), 121.3 (C), 111.3 (CH), 67.3 (CH_2), 64.4 (CH_2), 61.2 (CH), 61.1 (CH), 41.9 (CH), 29.8 (CH_2), 27.1 (3 CH_3), 25.5 (CH_2), 19.6 (C)
IR (Neat, cm^{-1})	3161, 3070, 3030, 2957 2930, 2857, 1711, 1623, 1590, 1573, 1540, 1488, 1471, 1428, 1407, 1389, 1362, 1331, 1291, 1245, 1214, 1148, 1107, 1091, 1075, 1011
HRMS (ESI+, m/z)	$[M+H]^+$ calcd. for $C_{43}H_{44}BrClN_3O_3SSi$ 824.1745, found 824.1765
HPLC Analysis	Daicel Chiralpak IB, Heptane/*i*PrOH = 95/5, flow rate = 1 mL/min, 254 nm<br>major isomer: t_R = 9.87 min, minor isomer: t_R = 13.97 min
Enantiomeric excess	97%
$[\alpha]_D^{22}$	-2.78° (*c* 3.41, $CHCl_3$)

benzyl ((2*R*,3*S*,4*R*)-2-(4-bromophenyl)-3-(3-((*tert*-butyldiphenylsilyl)oxy)propyl)-8-chloro-3,4-dihydro-2*H*-benzo[4,5]thiazolo[3,2-*a*]pyrimidin-4-yl)carbamate
4t

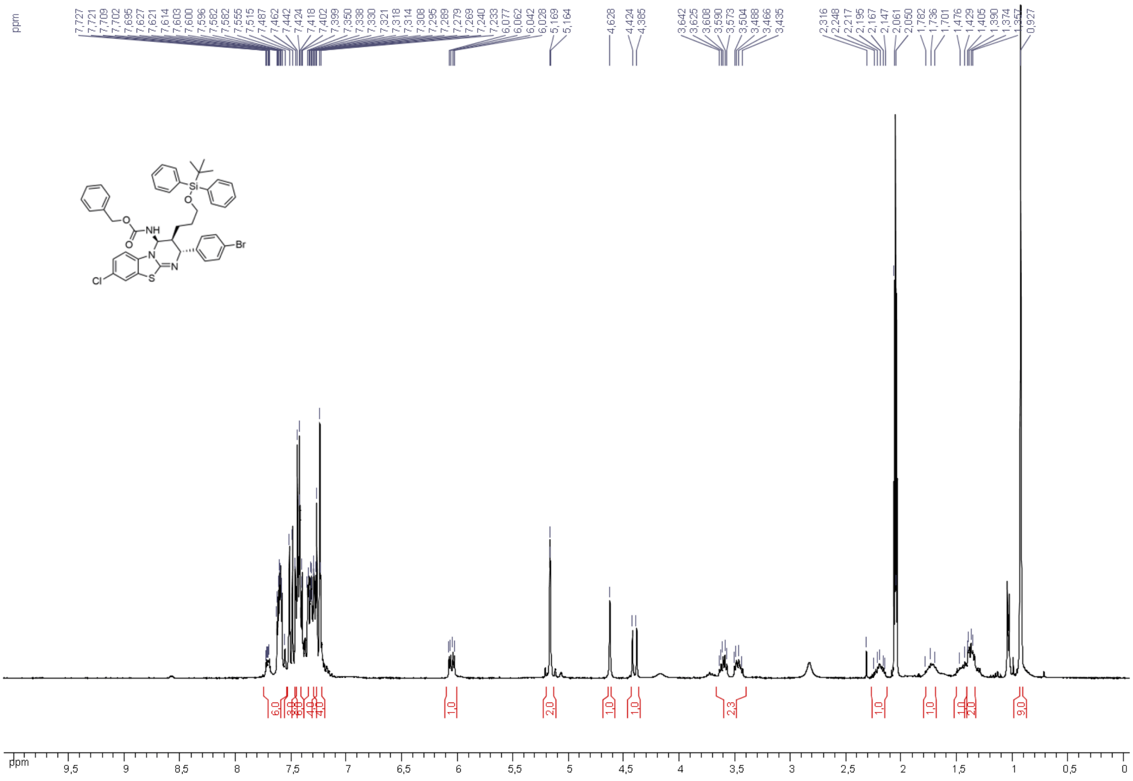

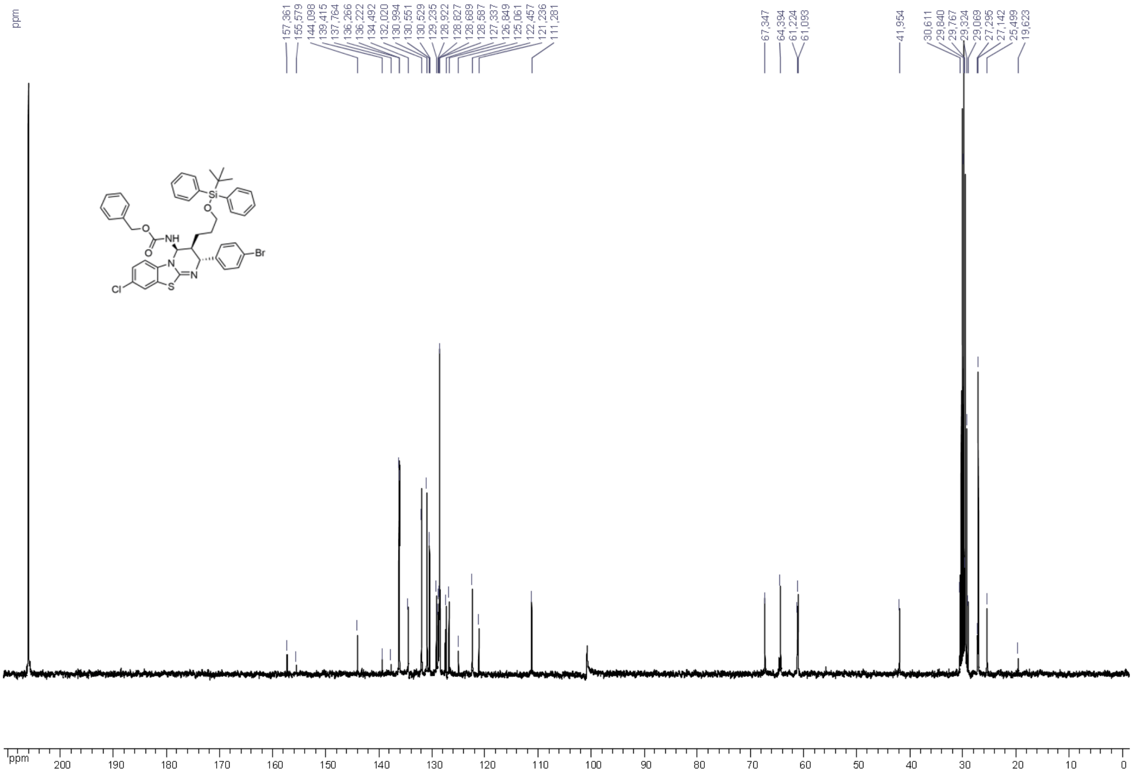

benzyl ((2*R*,3*S*,4*R*)-2-(4-bromophenyl)-3-(3-((*tert*-butyldiphenylsilyl)oxy)propyl)-8-chloro-3,4-dihydro-2*H*-benzo[4,5]thiazolo[3,2-*a*]pyrimidin-4-yl)carbamate
4t

Chiralpak IB, Heptane/*i*PrOH = 95/5, 1 mL/min, 254 nm

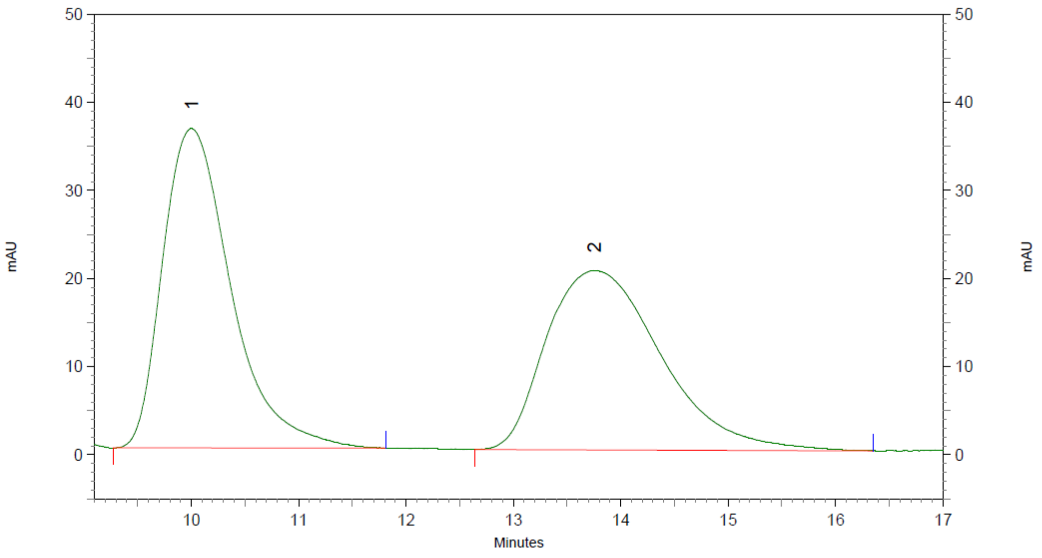

DAD-CH1 254 nm
Results

Pk #	Retention Time	Area	Area %
1	10,00	6408385	51,49
2	13,75	6036952	48,51

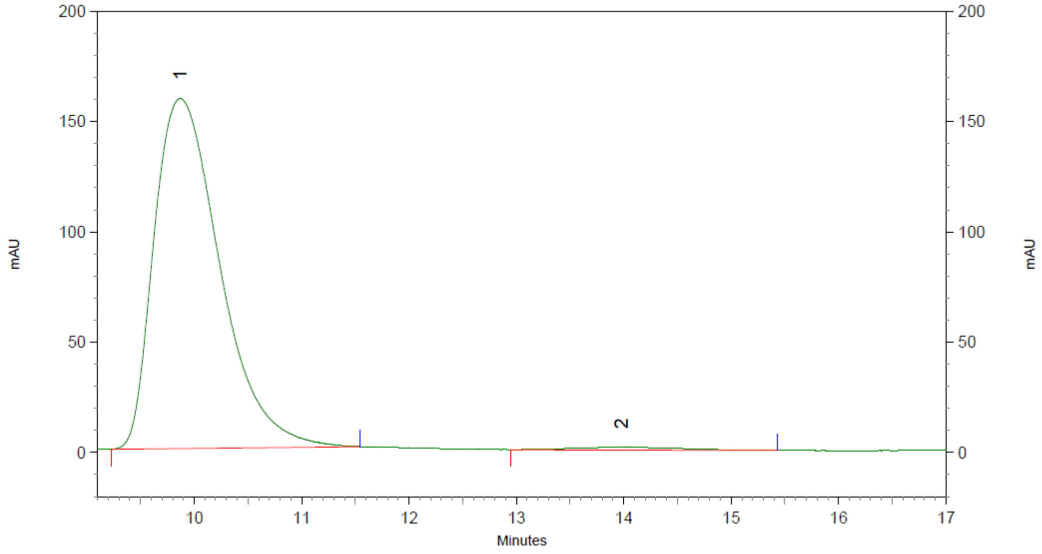

DAD-CH1 254 nm
Results

Pk #	Retention Time	Area	Area %
1	9,87	27042297	98,69
2	13,97	359628	1,31

benzyl ((2*R*,3*S*,4*R*)-2-(4-bromophenyl)-8-chloro-3-((*S*)-2,6-dimethylhept-5-en-1-yl)-3,4-dihydro-2*H*-benzo[4,5]thiazolo[3,2-*a*]pyrimidin-4-yl)carbamate

4u

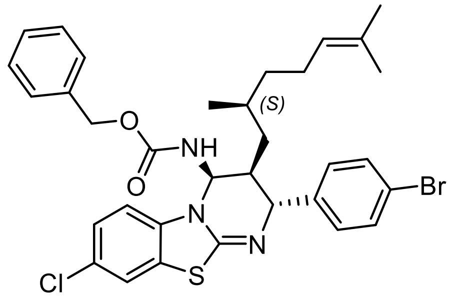

Compound **4u** was prepared according to the general procedure **D** from 2-benzothiazolimine **2f** (35.2 mg, 0.1 mmol) and enecarbamate **3h** (97.1 mg, 0.3 mmol) as starting materials. Purification on a column of silica gel with a gradient of ethyl acetate in heptane (from 9:1 to 85:15) as eluent gave the desired product as only one diastereomer.

Chemical formula	$C_{33}H_{35}BrClN_3O_2S$
M (g.mol^{-1})	653.08
Yield	45.0 mg, 69%
Aspect	White foam
Rf	0.8 (Hept/AcOEt : 7/3)
^{1}H NMR (300 MHz, Acetone-d$_6$)	**δ :** 7.73 (d, *J* = 10.3 Hz, 1H, NH), 7.49 (d, *J* = 8.5 Hz, 2H), 7.39-7.31 (m, 6H), 7.26 (s, 2H), 7.24 (d, *J* = 8.6 Hz, 2H), 5.92 (dd, *J* = 10.5, 4.2 Hz, 1H), 5.26 (d, *J* = 12.5 Hz, 1H), 5.13 (d, *J* = 12.5 Hz, 1H), 4.99 (tt, *J* = 6.8, 1.0 Hz, 1H), 4.27 (d, *J* = 11.7 Hz, 1H), 2.22-2.12 (m, 1H), 1.73 (q, *J* = 7.2 Hz, 2H), 1.63 (s, 3H), 1.52 (s, 3H), 1.47-1.32 (m, 1H), 1.27-1.15 (m, 1H), 1.13-0.99 (m, 1H), 0.94-0.79 (m, 2H), 0.72 (d, *J* = 7.8 Hz, 3H)
^{13}C NMR (75 MHz, Acetone-d$_6$)	**δ :** 157.4 (C), 155.9 (C), 143.9 (C), 139.4 (C), 138.3 (C), 131.9 (2 CH), 131.6 (C), 131.2 (2 CH), 129.3 (2 CH), 128.9 (2 CH or CH), 128.8 (CH or 2 CH), 127.7 (C), 126.9 (CH), 125.5 (CH), 125.2 (C), 122.4 (CH), 121.3 (C), 111.4 (CH), 67.3 (CH_2), 61.6 (CH), 61.2 (CH), 39.6 (CH), 36.1 (CH_2), 35.9 (CH_2), 29.4 (CH), 25.9 (CH_3), 25.4 (CH_2), 20.8 (CH_3), 17.8 (CH_3)
IR (Neat, cm^{-1})	2957, 2924, 1710, 1625, 1572, 1542, 1489, 1470, 1406, 1377, 1334, 1244, 1213, 1073, 1010
HRMS (ESI+, m/z)	$[M+H]^+$ calcd. for $C_{33}H_{36}BrClN_3O_2S$ 652.1400, found 652.1417
HPLC Analysis	Daicel Chiralpak IA, Heptane/*i*PrOH = 98/2, flow rate = 1 mL/min, 220 nm<br>major isomer: t_R = 20.19 min, minor isomer: t_R = 60.09 min
Enantiomeric excess	94%
$[\alpha]_D^{22}$	-135.00° (*c* 0.3, $CHCl_3$)

benzyl ((2*R*,3*S*,4*R*)-2-(4-bromophenyl)-8-chloro-3-((*S*)-2,6-dimethylhept-5-en-1-yl)-3,4-dihydro-2*H*-benzo[4,5]thiazolo[3,2-*a*]pyrimidin-4-yl)carbamate

4u

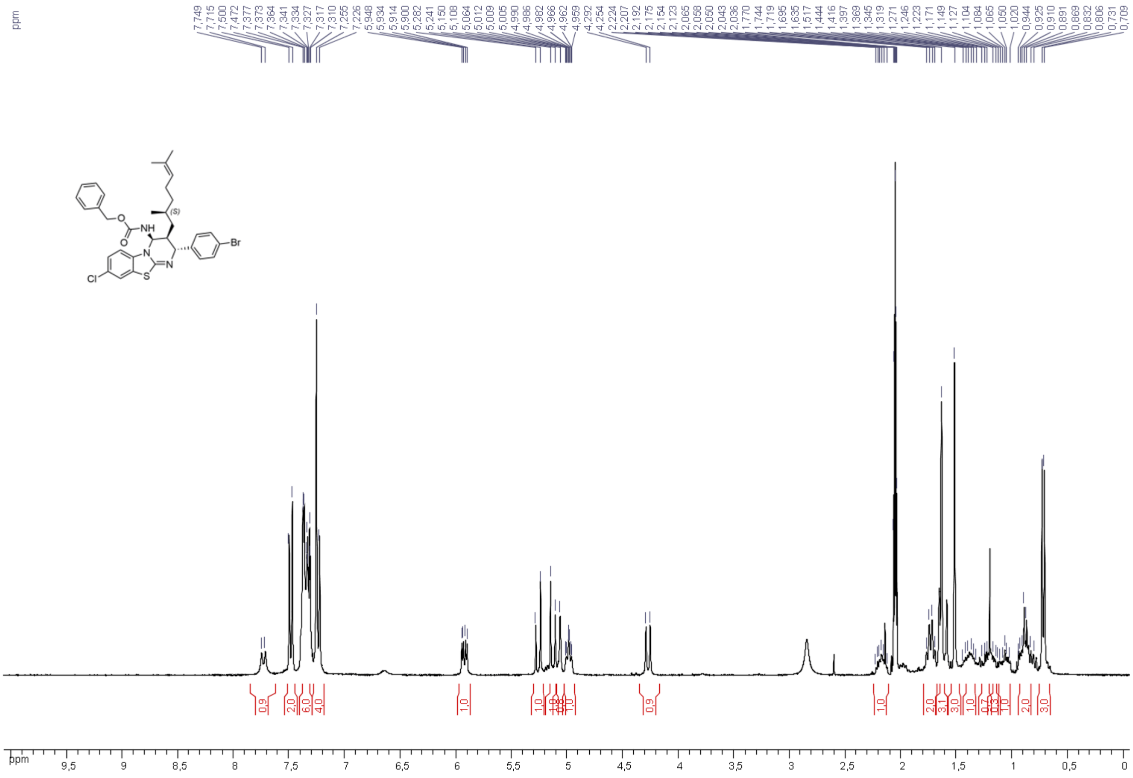

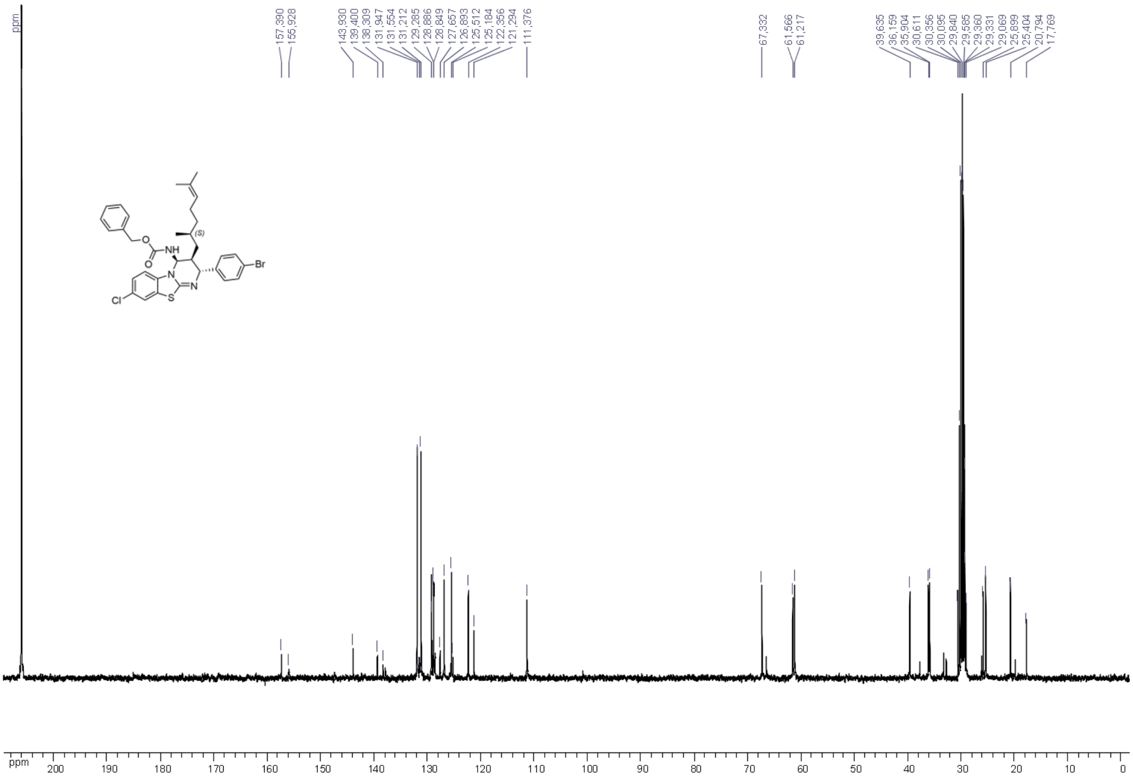

benzyl ((2*R*,3*S*,4*R*)-2-(4-bromophenyl)-8-chloro-3-((*S*)-2,6-dimethylhept-5-en-1-yl)-3,4-dihydro-2*H*-benzo[4,5]thiazolo[3,2-*a*]pyrimidin-4-yl)carbamate
4u

Chiralpak IA, Heptane/*i*PrOH = 98/2, 1 mL/min, 220 nm

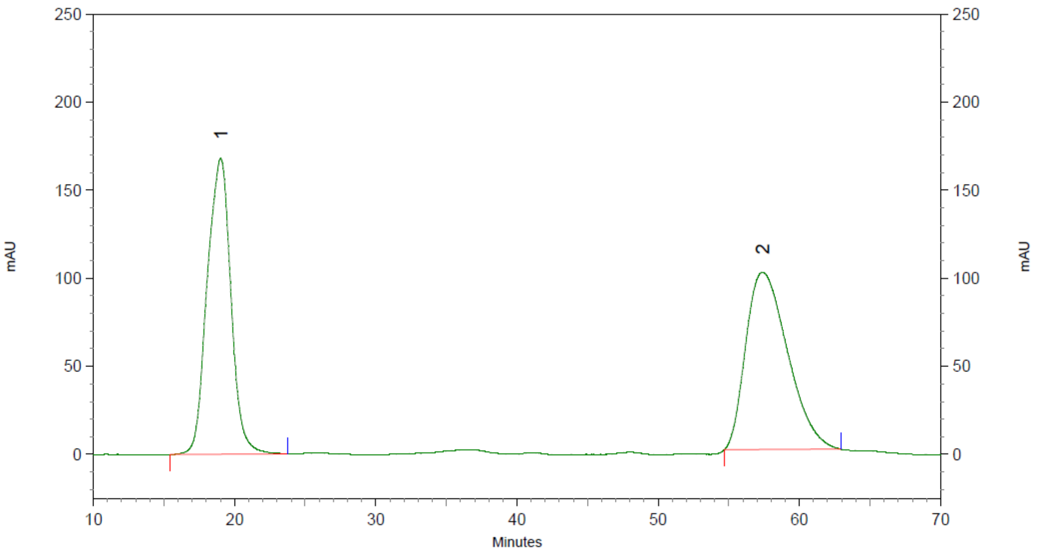

DAD-CH4 220 nm
Results

Pk #	Retention Time	Area	Area %
1	19,01	78406050	48,59
2	57,39	82966182	51,41

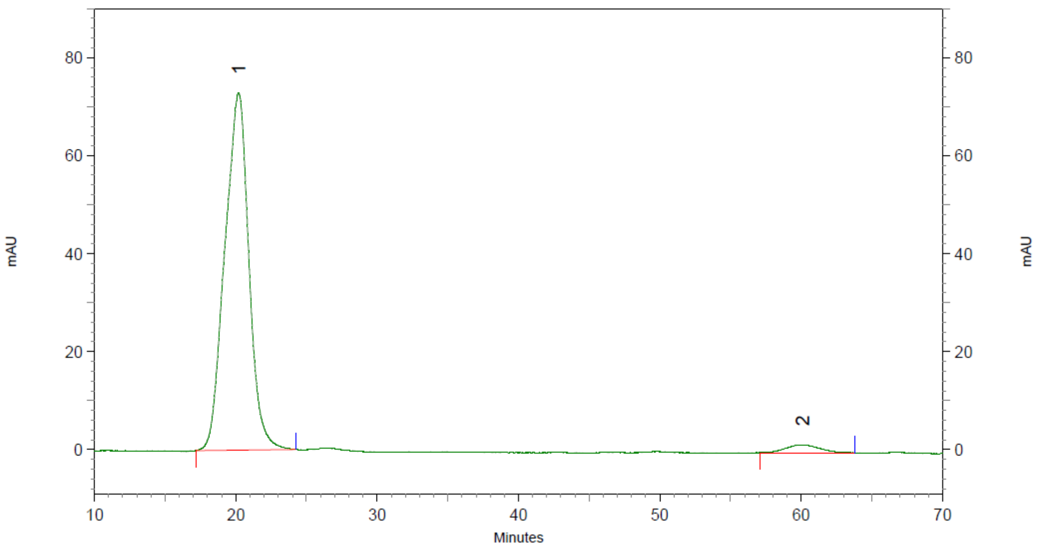

DAD-CH4 220 nm
Results

Pk #	Retention Time	Area	Area %
1	20,19	34431549	96,92
2	60,09	1094887	3,08

benzyl (*E*)-(2-(3-bromophenyl)-8-methoxy-3-(prop-1-en-1-yl)-3,4-dihydro-2H-benzo[4,5]thiazolo[3,2-*a*]pyrimidin-4-yl)carbamate
4v

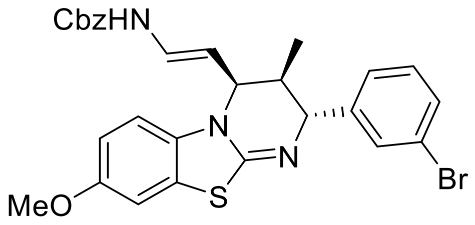

Compound **4v** was prepared according to the general procedure **F** from 2-benzothiazolimine **2k** (34.7 mg, 0.1 mmol) and dienecarbamate **3i** (65.2 mg, 0.3 mmol) as starting materials. Purification on a column of silica gel with a gradient of ethyl acetate in heptane (from 85:15) as eluent gave the desired product as only one diastereomer.

Chemical formula	$C_{28}H_{26}BrN_3O_3S$
M (g.mol-1)	564.50
Yield	28.7 mg, 51%
Aspect	Colorless oil
Rf	0.2 (Hept/AcOEt : 7/3)
^{1}H NMR (300 MHz, Acetonitrile-d3)	**δ :** 7.61 (s, 1H), 7.44–7.39 (m, 1H), 7.38–7.33 (m, 8H), 7.26 (d, *J* = 7.8 Hz, 1H), 7.04 (s, 1H), 6.79 (s, 1H), 6.51 (dd, *J* = 14.1, 10.5 Hz, 1H), 5.36 (dd, *J* = 14.3, 5.4 Hz, 1H), 5.05 (d, *J* = 17.3 Hz, 2H), 4.73 (d, *J* = 3.6 Hz, 1H), 4.46 (d, *J* = 5.9 Hz, 1H), 3.76 (s, 3H), 2.22 (s, 1H), 0.59 (d, *J* = 7.0 Hz, 3H).
^{13}C NMR (75 MHz, Acetonitrile-d3)	**δ :** 180.7 (C), 147.3 (C), 131.1 (CH), 130.9 (CH), 130.1 (CH), 129.6 (C), 129.5 (CH), 129.5 (CH), 129.4 (C), 129.2 (C), 129.1 (CH), 128.9 (CH), 128.7 (CH), 127.8 (CH), 127.7 (C), 127.7 (C), 127.0 (CH), 112.8 (CH), 110.2 (CH), 109.4 (CH), 109.1 (CH), 67.6 (CH_2), 66.8 (C), 59.5 (CH), 57.4 (CH), 56.5 (CH), 36.3 (CH_3), 12.6 (CH_3).
IR (Neat, cm-1)	3301, 2965, 1716, 1675, 1612, 1583, 1490, 1330, 1266, 1232, 1184, 1041, 738, 699.
HRMS (ESI+, m/z)	$[M+H]^+$ calcd. for $C_{28}H_{27}BrN_3O_3S$ 564.0951, found 564.0956
HPLC Analysis	Daicel Chiralpak IA, Heptane/EtOH = 90/10, flow rate = 1 mL/min, 250 nm minor isomer: t_R = 26.85 min, major isomer: t_R = 17.70 min
Enantiomeric excess	98%
$[\alpha]_D^{22}$	-6.32° (*c* 0.1, CH_3CN)

benzyl (2-(3-bromophenyl)-8-methoxy-3-propyl-3,4-dihydro-2H-benzo[4,5]thiazolo[3,2-*a*]pyrimidin-4-yl)carbamate

4w

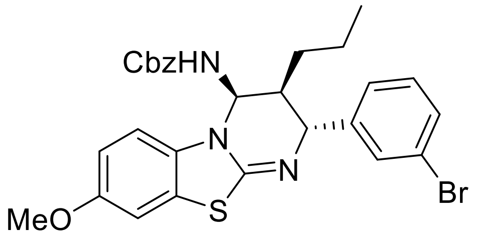

Compound **4w** was prepared according to the general procedure **I** from 2-benzothiazolimine **2k** (34.7 mg, 0.1 mmol) and enecarbamate **3e** (65.8 mg, 0.3 mmol) as starting materials. Purification on a column of silica gel with a gradient of ethyl acetate in heptane (from 85:15) as eluent gave the desired product as only one diastereomer.

Chemical formula	$C_{28}H_{28}BrN_3O_3S$
M (g.mol-1)	566.51
Yield	22.8 mg, 40%
Aspect	Colorless oil
Rf	0.2 (Hept/AcOEt : 7/3)
^{1}H NMR (300 MHz, Acetone-d6)	**δ :** 7.50 (s, 1H), 7.47 – 7.26 (m, 9H), 7.18 (d, *J* = 8.7 Hz, 1H), 7.08 (s, 1H), 6.81 (d, *J* = 8.7 Hz, 1H), 5.95 (dd, *J* = 10.2, 3.8 Hz, 1H), 5.19 (q, *J* = 12.5 Hz, 2H), 4.38 (d, *J* = 11.6 Hz, 1H), 3.79 (s, 3H), 2.26 – 2.09 (m, 1H), 1.57 – 1.29 (m, 2H), 1.20 – 0.87 (m, 2H), 0.73 (t, *J* = 7.1 Hz, 3H).
^{13}C NMR (75 MHz, Acetone-d6)	**δ :** 157.3 (C), 156.7 (C), 156.2 (C), 148.0, 138.0 (C), 136.4 (C), 134.4 (C), 131.8 (CH), 130.8 (CH), 130.7 (CH), 129.3 (CH), 129.2 (CH), 128.8 (CH), 128.6 (CH), 128.1 (2 CH), 124.1 (C), 122.7 (C), 112.6 (CH), 110.7 (CH), 108.9 (CH), 67.2 (CH), 61.1 (CH_2), 61.1 (CH), 56.1 (CH), 42.0 (CH_2), 30.9 (CH_2), 20.2 (CH_3), 14.4 (CH_3).
IR (Neat, cm-1)	3316, 2956, 1711, 1605, 1542, 1471, 1437, 1265, 1223, 1057, 1029, 830, 697.
HRMS (ESI+, m/z)	$[M+H]^+$ calcd. for $C_{28}H_{28}BrN_3O_3S$ 566.1108, found 566.1102
HPLC Analysis	Daicel Chiralpak IA, Heptane/EtOH = 90/10, flow rate = 1 mL/min, 250 nm<br>minor isomer: t_R = 8.28 min, major isomer: t_R = 10.53 min
Enantiomeric excess	78%
$[\alpha]_D^{22}$	-4.72° (*c* 0.1, CH_3CN)

benzyl (2-(3-bromophenyl)-8-methoxy-3-propyl-3,4-dihydro-2H-benzo[4,5]thiazolo[3,2-*a*]pyrimidin-4-yl)carbamate
4w

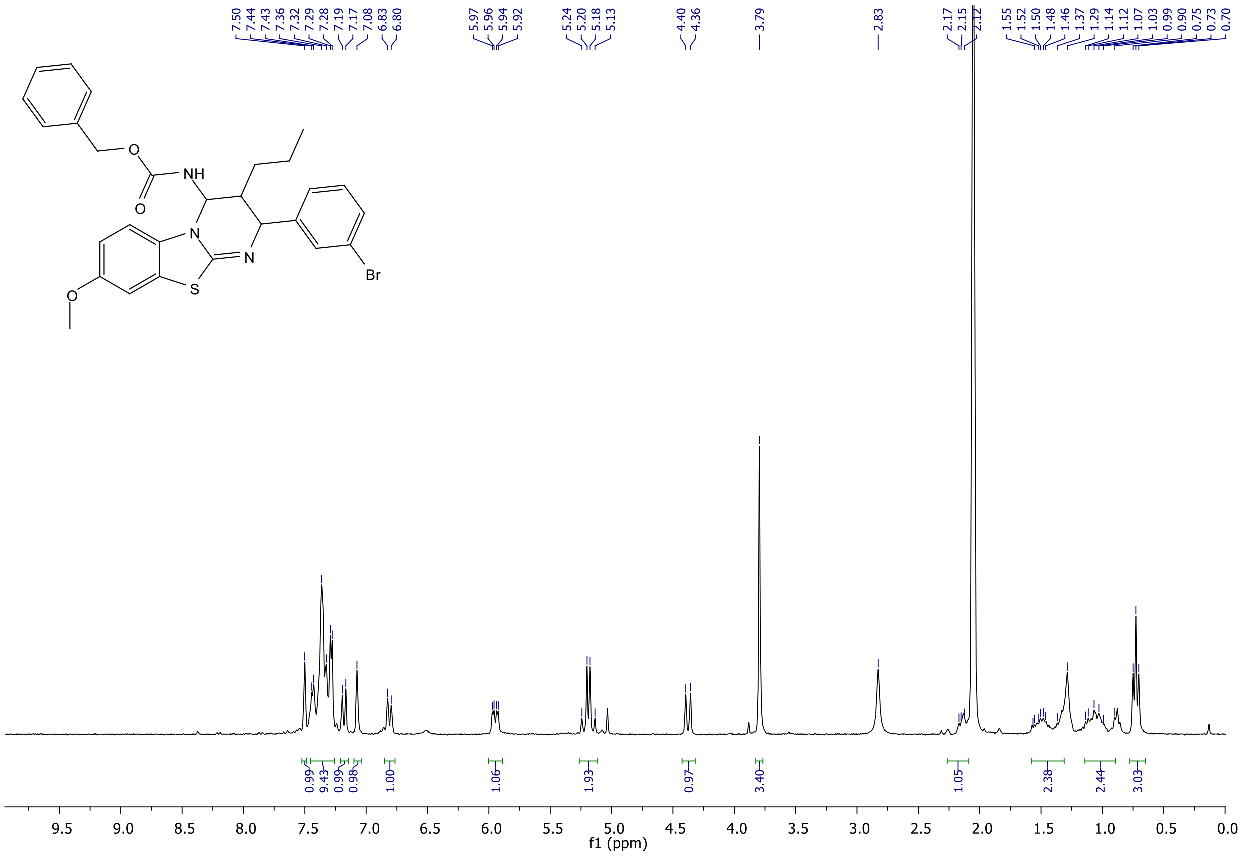

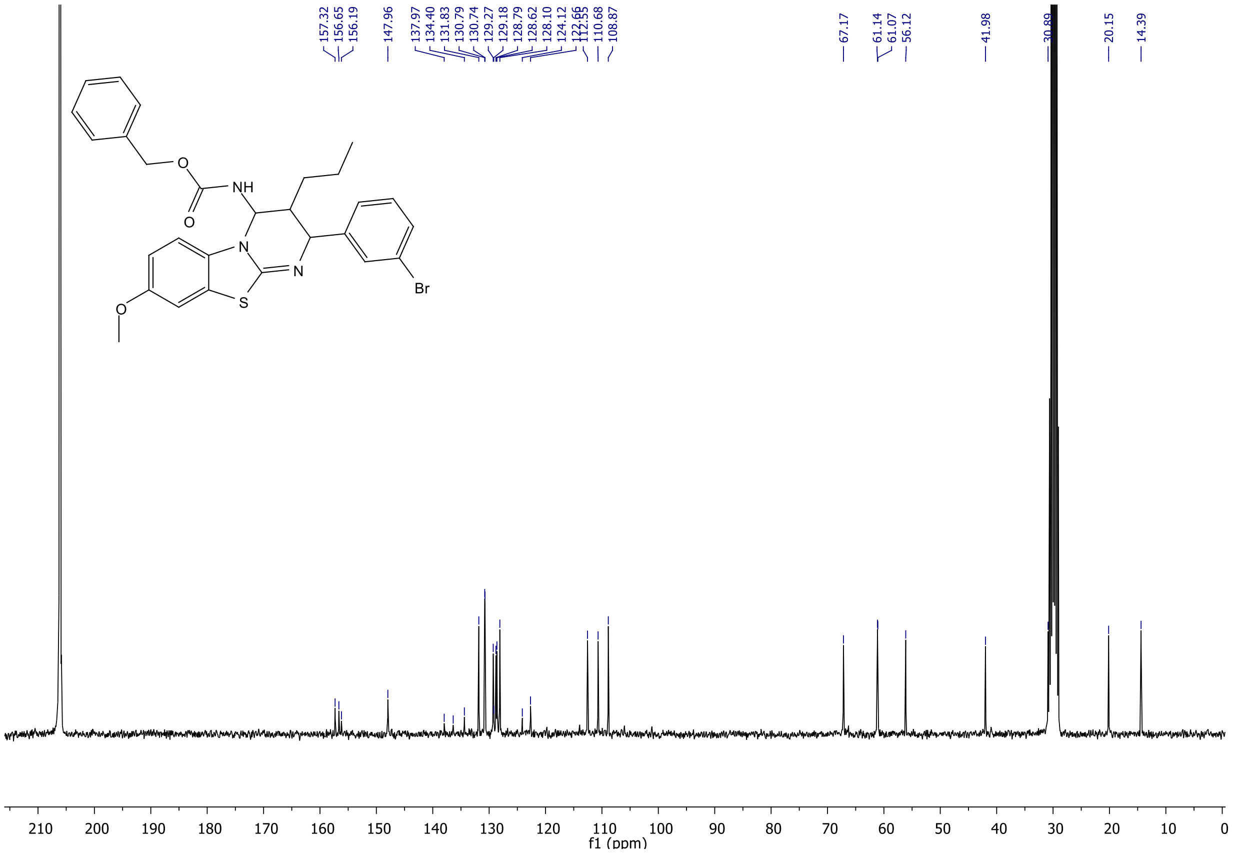

benzyl (2-(3-bromophenyl)-8-methoxy-3-propyl-3,4-dihydro-2H-benzo[4,5]thiazolo[3,2-*a*]pyrimidin-4-yl)carbamate
4w

Chiralpak IA, Heptane/EtOH = 90/10, 1 mL/min, 250 nm

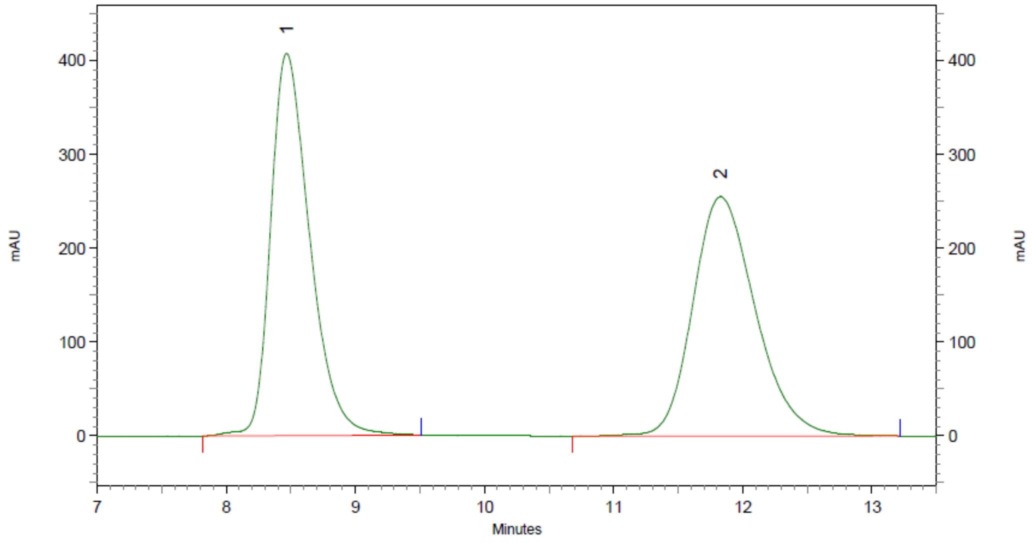

DAD-CH1 250 nm
Results

Pk #	Retention Time	Area	Area %
1	8,47	34340598	50,00
2	11,83	34339559	50,00

Chiralpak IA, Heptane/EtOH = 90/10, 1 mL/min, 254 nm

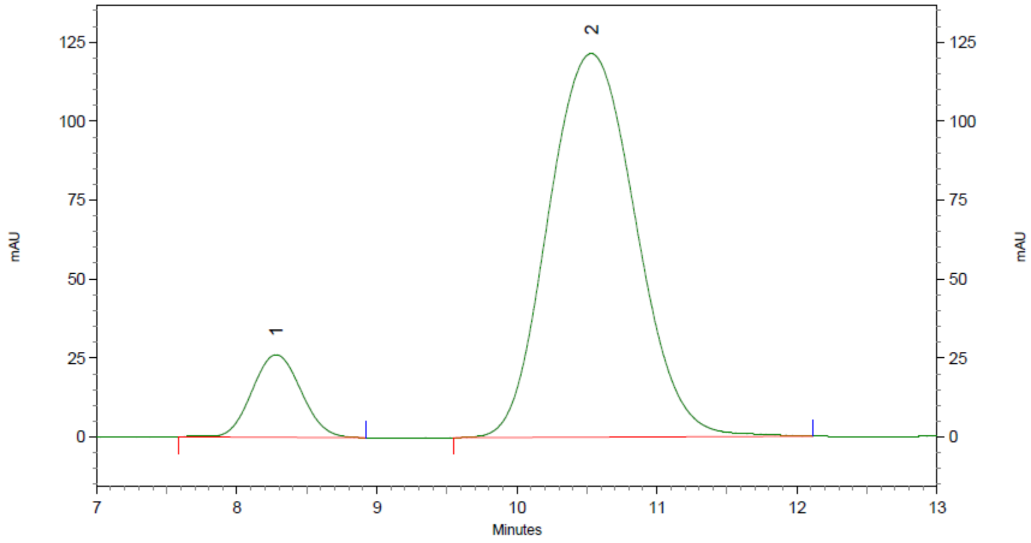

DAD-CH1 254 nm
Results

Pk #	Retention Time	Area	Area %
1	8,28	2547668	10,81
2	10,53	21024406	89,19

VI. Post-transformation : Synthesis of compound 5 and 10

benzyl (2-((3-bromophenyl)((6-methoxybenzo[*d*]thiazol-2-yl)amino)methyl)-1-ethoxypentyl)carbamate
5

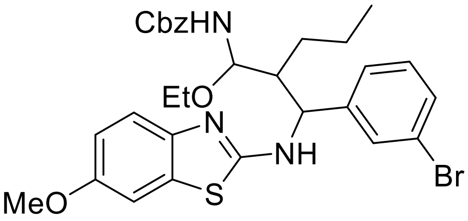

Compound **12** was prepared according to the general procedure **I** from 2-benzothiazolimine **2k** (34.7 mg, 0.1 mmol) and enecarbamate **3e** (65.8 mg, 0.3 mmol) as starting materials. Purification on a column of silica gel with a gradient of ethyl acetate in heptane (from 85:15) as eluent gave the desired product as only one diastereomer.

Chemical formula	$C_{30}H_{34}BrN_3O_4S$
M (g.mol-1)	612.58
Yield	9.5 mg, 16%
Aspect	Colorless oil
Rf	0.7 (Hept/AcOEt : 7/3)
^{1}H NMR (300 MHz, Acetone-d6)	**δ :** 7.75 (s, 1H), 7.63 (s, 1H), 7.55 (d, *J* = 7.6 Hz, 1H), 7.52 (d, *J* = 7.9 Hz, 1H), 7.41 (d, *J* = 8.7 Hz, 2H), 7.38 – 7.32 (m, 4H), 7.30 (s, 2H), 6.90 (d, *J* = 8.7 Hz, 1H), 6.63 (s, 1H), 6.37 (s, 1H), 5.06 (s, 2H), 3.90 – 3.83 (m, 1H), 3.80 (s, 3H), 3.75 – 3.66 (m, 1H), 1.77 (s, 1H), 1.41 – 1.28 (m, 2H), 1.25 (t, *J* = 7.0 Hz, 3H), 1.22 – 1.05 (m, 2H), 0.96 – 0.77 (m, 3H).
^{13}C NMR (75 MHz, Acetone-d6)	**δ :** 180.7 (C), 164.6 (C), 136.5 (C), 132.9 (C), 132.0 (2 CH), 131.2 (2 CH), 130.2 (CH), 129.8 (C), 129.2 (C), 128.6 (2 CH), 126.3 (CH), 124.6 (CH), 122.7 (C), 120.4 (CH), 116.9 (CH), 114.3 (CH), 106.1 (CH), 105.4 (C), 84.9 (CH), 66.5 (CH_2), 64.4 (CH), 56.2 (CH_3), 56.1 (CH_2), 28.5 (CH_2), 22.9 (CH_2), 15.5 (CH_3), 14.3 (CH_3).
IR (Neat, cm-1)	3316, 2956, 1711, 1605, 1542, 1471, 1437, 1265, 1223, 1057, 1029, 830, 697.
HRMS (ESI+, m/z)	$[M+H]^+$ calcd. for $C_{30}H_{35}BrN_3O_4S$ 612.1526, found 612.1548
HPLC Analysis	Daicel Chiralpak IA, Heptane/EtOH = 90/10, flow rate = 1 mL/min, 280 nm<br>minor isomer: t_R = 14.87 min, major isomer: t_R = 12.54 min
Enantiomeric excess	78%
$[\alpha]_D22$	-2.17° (*c* 0.1, CH_3CN)

benzyl (2-((3-bromophenyl)((6-methoxybenzo[*d*]thiazol-2-yl)amino)methyl)-1-ethoxypentyl)carbamate

5

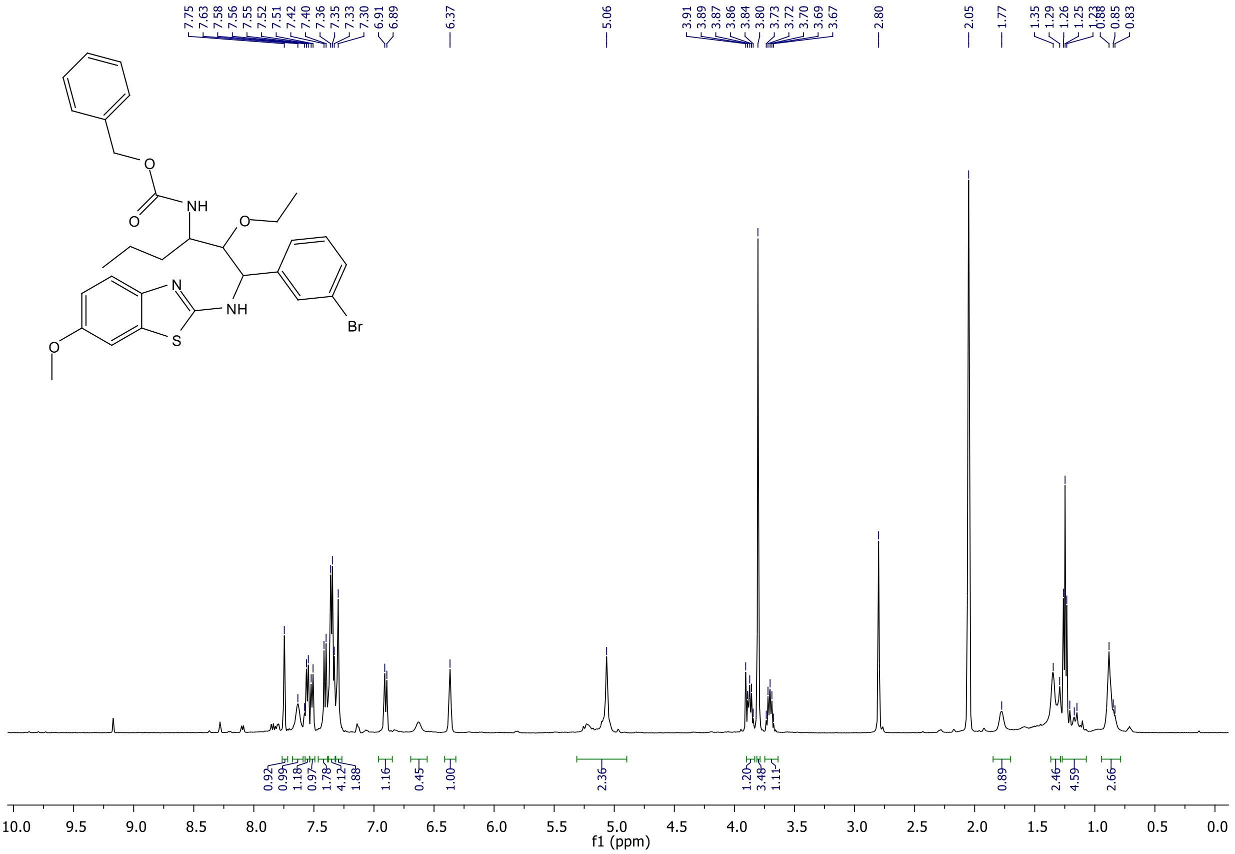

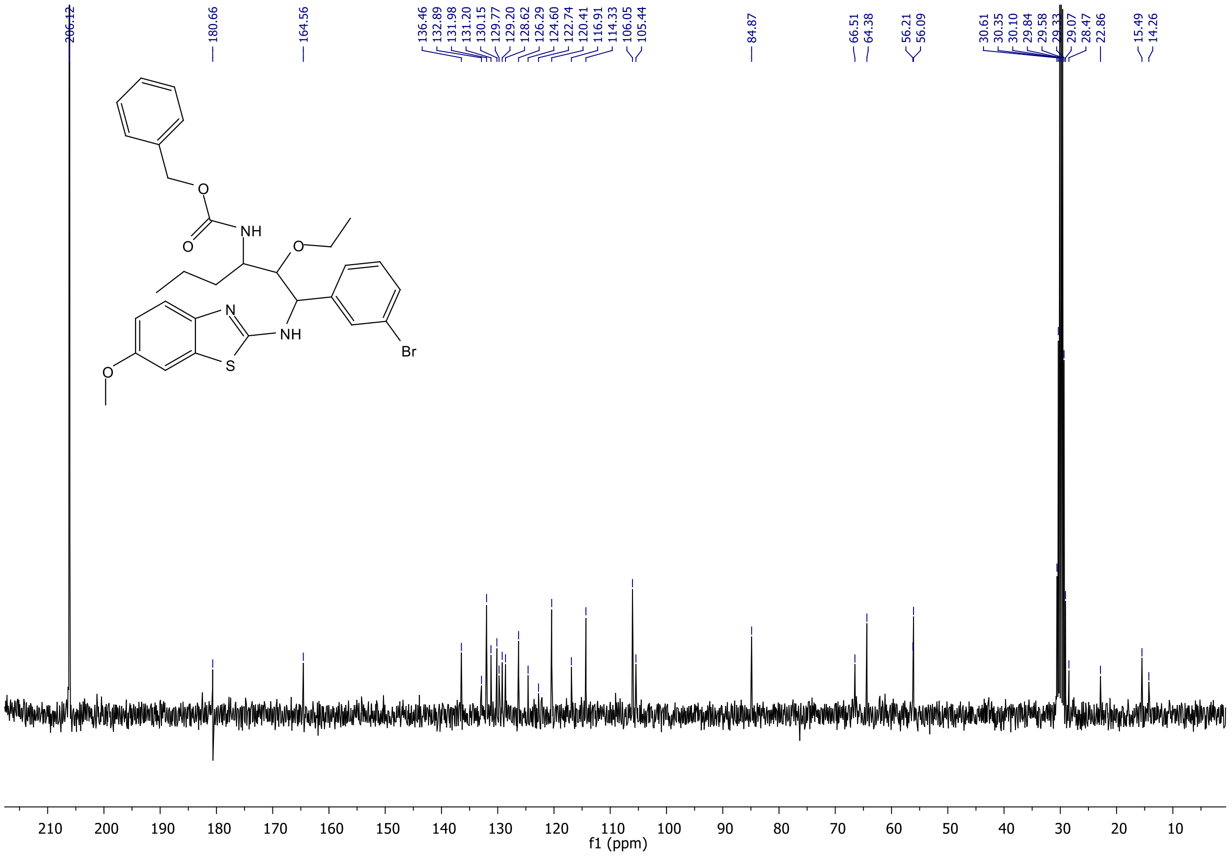

benzyl (2-((3-bromophenyl)((6-methoxybenzo[*d*]thiazol-2-yl)amino)methyl)-1-ethoxypentyl)carbamate
5

Chiralpak IA, Heptane/EtOH = 90/10, 1 mL/min, 280 nm

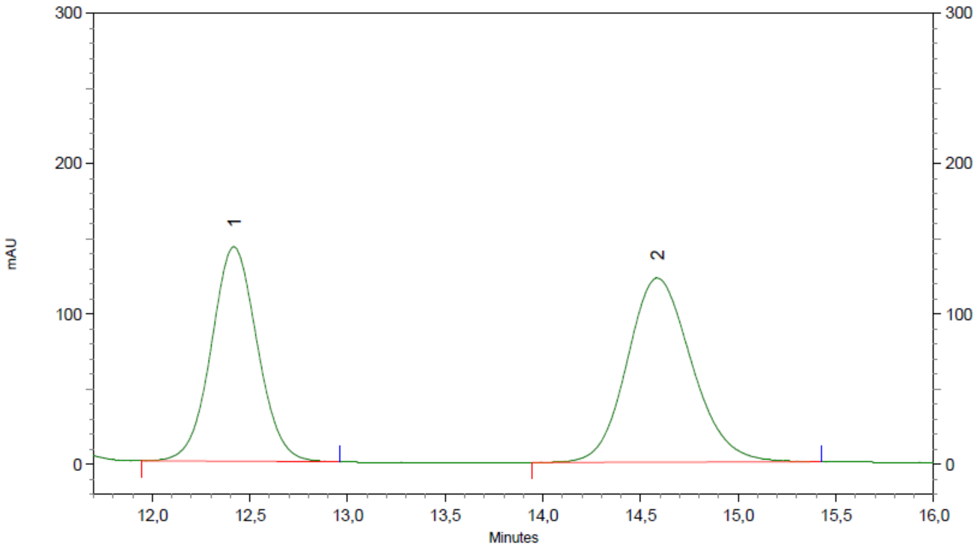

DAD-CH3 280 nm
Results

Pk #	Retention Time	Area	Area %
1	12,42	11274491	50,62
2	14,59	10998065	49,38

Chiralpak IA, Heptane/EtOH = 90/10, 1 mL/min, 280 nm

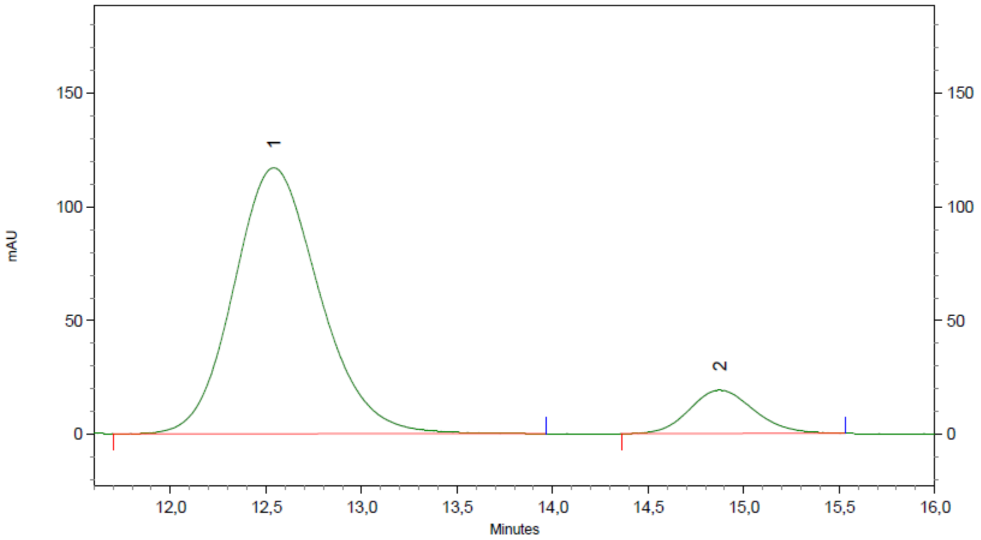

DAD-CH3 280 nm
Results

Pk #	Retention Time	Area	Area %
1	12,54	13505406	88,75
2	14,87	1711516	11,25

(2*R*,3*S*,4S)-2-(4-bromophenyl)-3,8-dimethyl-3,4-dihydro-2*H*-benzo[4,5]thiazolo[3,2-*a*]pyrimidin-4-ol
10

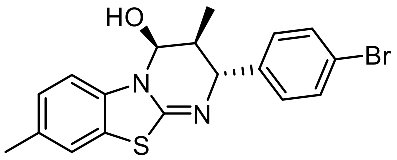

To a solution of compound **4q** (15 mg, 0.03 mmol, 1 equiv) in 1,4-dioxane (1 mL) was added concentrated HCl (37%, 5 drops) at room temperature and the reaction mixture was stirred for 1 h 30. After completion (monitored by TLC), the resulting solution was evaporated under reduced pressure and the crude product was diluted in methanol (2 mL). The solution was passed three times through a small pad (1 cm^3) of Dowex® strong base anion exchange resin (OH^-) which was washed several times with methanol. The solvent was removed under reduced pressure and the desired compound **11** was directly obtained pure, without any purification step.

Chemical formula	$C_{18}H_{17}BrN_2OS$
M (g.mol^{-1})	389.31
Yield	9 mg, 75%
Aspect	Colorless oil
Rf	0.2 (Hept/AcOEt : 7/3)
^{1}H NMR (300 MHz, Acetone-d$_6$)	**δ :** 7.50 (d, *J* = 8.4 Hz, 2H), 7.26 (d, *J* = 8.7 Hz, 2H), 7.23 (m, 1H), 7.03 (m, 2H), 4.96 (m, 1H), 4.30 (d, *J* = 10.8 Hz, 1H), 2.30 (s, 3H), 1.98-1.87 (m, 1H), 0.87 (d, *J* = 7.0 Hz, 3H)<br>*OH was not visible in the spectrum*
^{13}C NMR (75 MHz, Acetone-d$_6$)	**δ :** 145.2 (C), 138.8 (C), 131.9 (C), 131.7 (2 CH), 130.9 (2 CH), 130.2 (C), 127.3 (CH), 123.4 (C), 123.0 (CH), 120.7 (C), 109.2 (CH), 65.2 (CH), 61.2 (CH), 38.5 (CH), 20.9 (CH_3), 14.7 (CH_3)
IR (Neat, cm^{-1})	3215, 2927, 1619, 1573, 1486, 1456, 1404, 1315, 1284, 1266, 1226,1195, 1071, 1010
HRMS (ESI-, m/z)	$[M-H]^-$ calcd. for $C_{18}H_{16}BrN_2OS$ 387.0167, found 387.0155
HPLC Analysis	Daicel Chiralpak IA, Heptane/*i*PrOH = 85/15, flow rate = 1 mL/min, 254 nm<br>major isomer: t_R = 13.65 min , minor isomer: t_R = 29.20 min
Enantiomeric excess	97%
$[\alpha]_D^{22}$	-0.55 (*c* 0.9, $CHCl_3$)

(2*R*,3*S*,4S)-2-(4-bromophenyl)-3,8-dimethyl-3,4-dihydro-2*H*-benzo[4,5]thiazolo[3,2-*a*]pyrimidin-4-ol
10

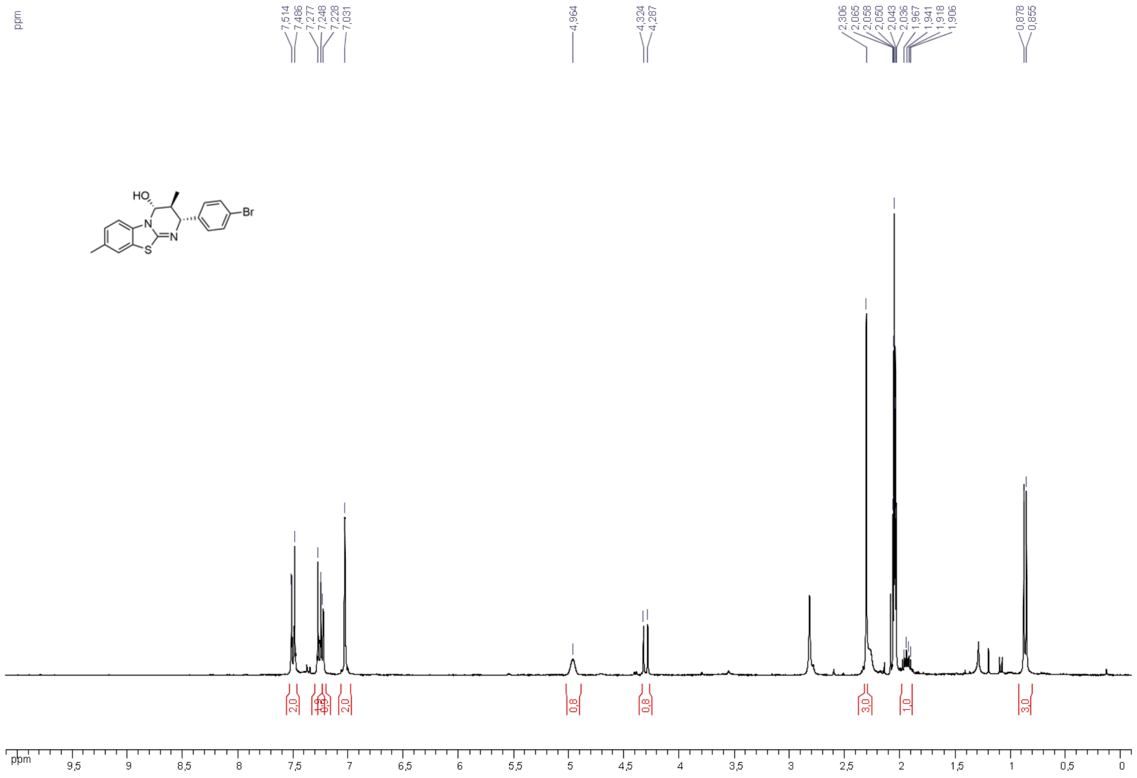

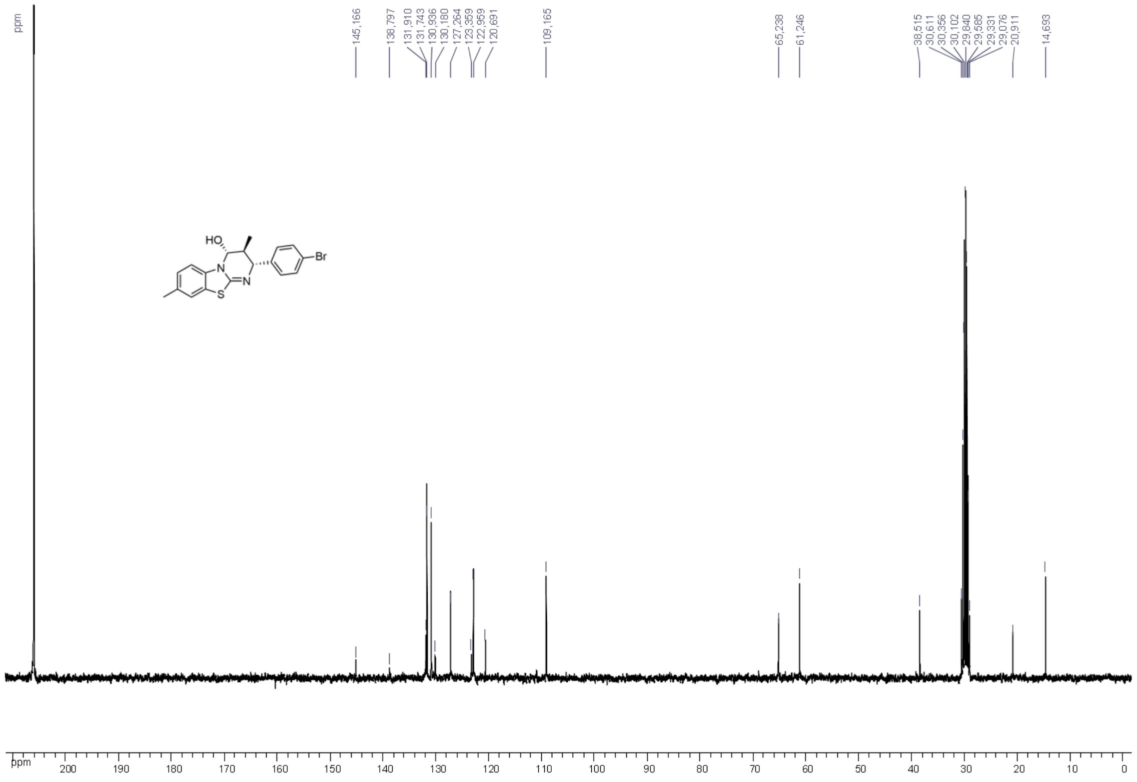

(2*R*,3*S*,4S)-2-(4-bromophenyl)-3,8-dimethyl-3,4-dihydro-2*H*-benzo[4,5]thiazolo[3,2-*a*]pyrimidin-4-ol

10

Chiralpak IA, Heptane/*i*PrOH = 85/15, 1 mL/min, 254 nm

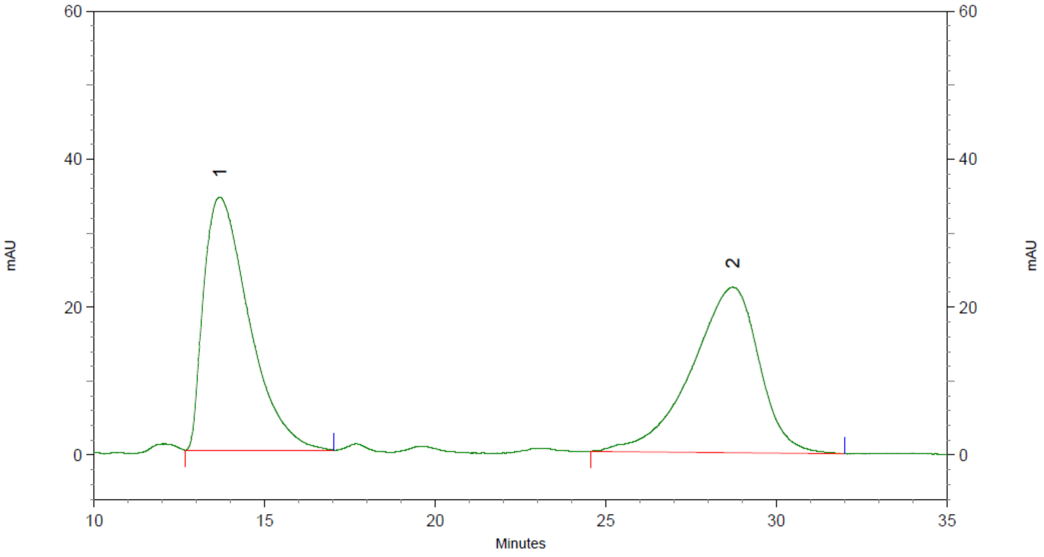

DAD-CH1 254 nm

Results

Pk #	Retention Time	Area	Area %
1	13,68	12601048	50,52
2	28,71	12340372	49,48

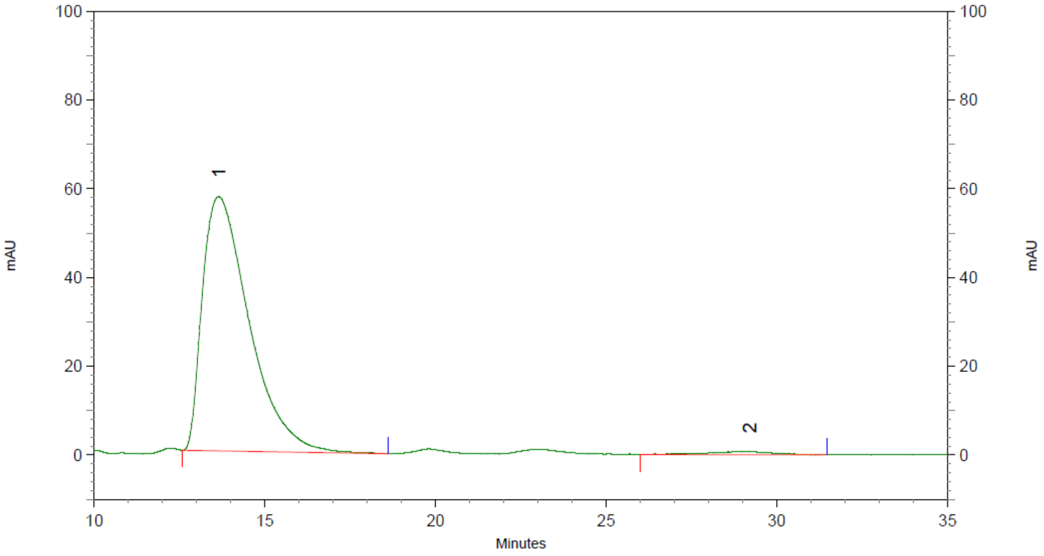

DAD-CH1 254 nm

Results

Pk #	Retention Time	Area	Area %
1	13,65	21781892	98,35
2	29,20	366511	1,65

VII. Computational models of *cis* and *trans* diastereoisomers of cycloadduct 4g

Computational method: Low energy conformations of ***cis*** and ***trans*-4g** were firstly identified using MMFF94[4] force field as implemented in the Tinker v8.4.4[5] software package. Geometries were then further optimised at the B3LYP/6-31G(d)[6] level using the Gaussian 09 revD.01[7] software package and characterized by vibrational analysis within the harmonic approximation at the same level of theory.

cis-4g

Electronic energy (RB3LYP): −1679.43181469 Ha

Lowest frequency: 5.5667 cm^{-1}

Free energy: −1679.058019 Ha

Cartesian coordinates:

C	-0.0892324489	1.9670253276	-1.3820164734
C	0.1567909829	3.2008226189	-1.9918063699
C	-0.5404836654	4.3492151472	-1.6125761433
C	-1.5278776288	4.2756355954	-0.6259046787
C	-1.7783101930	3.0509160134	-0.0177821961
C	-1.0442147441	1.8998425474	-0.3646843861
S	-3.0259499530	2.6849563045	1.1838801391
C	-2.5980779162	0.9456584680	1.1275454619
N	-1.4322180335	0.7728031677	0.3773534443
C	-0.7546353421	-0.5415504568	0.3633977997
C	-1.3253000864	-1.4188391707	1.5083150916
C	-2.8745622853	-1.3227687621	1.5245572150
N	-3.3125404352	0.0616646136	1.7030994683
C	-3.5203602130	-1.9983045233	0.3096862956
C	-4.2344326137	-1.2852606301	-0.6586705750
C	-4.7991494925	-1.9427366850	-1.7549961326
C	-4.6635845489	-3.3227777924	-1.8970833075
C	-3.9644988548	-4.0471897638	-0.9284332128

[4] Halgren, T. A. *J. Comput. Chem.* **1996**, *17*, 490.

[5] Ponder, J. W. *et al. Journal of Chemical Theory and Computation*, **2018**, *14*, 5273.

[6] (a) Sham, L.J. *et al. Phys. Rev.* **1965**, *140*, A1133. (b) Kohn, W. *et al. Phys. Rev.* **1964**, *136*, B864. (c) Becke, A. D. *J. Chem. Phys.* **1993**, *98*, 5648. (d) Parr, R. G. *et al. Phys. Rev.* **1988**, B37. (e) Pople, J. A. *et al. Ab Initio Molecular Orbital Theory; Wiley: New York*, **1986**.

[7] Gaussian 09, Revision D.01, M. J. Frisch, G. W. Trucks, H. B. Schlegel, G. E. Scuseria, M. A. Robb, J. R. Cheeseman, G. Scalmani, V. Barone, B. Mennucci, G. A. Petersson, H. Nakatsuji, M. Caricato, X. Li, H. P. Hratchian, A. F. Izmaylov, J. Bloino, G. Zheng, J. L. Sonnenberg, M. Hada, M. Ehara, K. Toyota, R. Fukuda, J. Hasegawa, M. Ishida, T. Nakajima, Y. Honda, O. Kitao, H. Nakai, T. Vreven, J. A. Montgomery, Jr., J. E. Peralta, F. Ogliaro, M. Bearpark, J. J. Heyd, E. Brothers, K. N. Kudin, V. N. Staroverov, T. Keith, R. Kobayashi, J. Normand, K. Raghavachari, A. Rendell, J. C. Burant, S. S. Iyengar, J. Tomasi, M. Cossi, N. Rega, J. M. Millam, M. Klene, J. E. Knox, J. B. Cross, V. Bakken, C. Adamo, J. Jaramillo, R. Gomperts, R. E. Stratmann, O. Yazyev, A. J. Austin, R. Cammi, C. Pomelli, J. W. Ochterski, R. L. Martin, K. Morokuma, V. G. Zakrzewski, G. A. Voth, P. Salvador, J. J. Dannenberg, S. Dapprich, A. D. Daniels, O. Farkas, J. B. Foresman, J. V. Ortiz, J. Cioslowski, and D. J. Fox, Gaussian, Inc., Wallingford CT, 2013.

C	-3.4029405308	-3.3894114105	0.1641364172
C	-0.7548381233	-1.0721272212	2.8945358176
N	0.6782919661	-0.3880528260	0.4768042907
C	1.5586931186	-0.8179287086	-0.4774816509
O	2.8278124764	-0.6715022427	-0.0218015838
O	1.2576587351	-1.2519151272	-1.5761269311
C	3.8687963114	-1.0472705026	-0.9576210874
C	5.1873627232	-0.5595904172	-0.4183062947
C	5.4016990861	0.8085755487	-0.2010217752
C	6.6273639924	1.2647662573	0.2791053605
C	7.6590480936	0.3584863204	0.5410722534
C	7.4552470554	-1.0041284505	0.3255279925
C	6.2223242341	-1.4595099422	-0.1473937265
H	0.4265380545	1.0780227140	-1.7228293684
H	0.8983432659	3.2555579382	-2.7837983905
H	-0.3336220871	5.2993739685	-2.0960685898
H	-2.1005290185	5.1552856029	-0.3469297601
H	-0.9306706012	-1.0286368729	-0.5995452039
H	-1.0333144937	-2.4462902198	1.2644877750
H	-3.2152367309	-1.8747513030	2.4102920776
H	-4.3676261103	-0.2150693069	-0.5446087496
H	-5.3503474334	-1.3701839954	-2.4965012575
H	-5.1035841962	-3.8326184968	-2.7499509971
H	-3.8611173017	-5.1252504929	-1.0211136530
H	-2.8728847465	-3.9667249028	0.9195213544
H	-1.2304056974	-1.7014961567	3.6541172376
H	0.3221839860	-1.2612618680	2.9525270794
H	-0.9454767960	-0.0301158874	3.1717537196
H	1.0690846466	-0.0391074799	1.3405885947
H	3.8623646525	-2.1349320308	-1.0788422552
H	3.6318082479	-0.6037504667	-1.9297130784
H	4.5994147718	1.5139193340	-0.4023686083
H	6.7810688097	2.3275890485	0.4458587530
H	8.6162608815	0.7151547086	0.9118114402
H	8.2516617554	-1.7147073442	0.5295554864
H	6.0638484749	-2.5234557880	-0.3079761283

***trans*-4g**

Electronic energy (RB3LYP): −1679.44053587 Ha

Lowest frequency: 6.1942 cm^{-1}

Free energy: −1679.066265 Ha

Cartesian coordinates:

C	-0.3019717826	-0.2937356160	-0.2533280062
C	-0.6383556543	-0.6255520084	1.0626727573
C	0.3177363413	-1.1179866377	1.9534992535
C	1.6418826755	-1.2939211821	1.5401043747
C	1.9840232568	-0.9646370988	0.2335407138
C	1.0225351060	-0.4603558096	-0.6613302698
S	3.5744851908	-1.0919510998	-0.5350012166
C	2.9141789209	-0.4606539862	-2.0775715766
N	1.5601356990	-0.1736829581	-1.9227330889
C	0.7973746377	0.3612064806	-3.0541649589
C	1.7759264837	1.0983694491	-3.9972468659
C	3.0245763261	0.2210106725	-4.3145961414
N	3.6514487501	-0.3136203225	-3.1075518482
C	2.7464232405	-0.8761239921	-5.3496077792
C	2.8249104340	-2.2376193471	-5.0325354315
C	2.5498009638	-3.2115081663	-5.9981274976
C	2.2045330321	-2.8392586262	-7.2964318085
C	2.1426050204	-1.4831716279	-7.6284024528
C	2.4128365405	-0.5142707751	-6.6641858509
C	2.1929648143	2.4493699785	-3.3937750346
N	0.0092485772	-0.6589745792	-3.7195562588
C	-1.3557425813	-0.6478782085	-3.6940930678
O	-1.8423293819	-1.6778057179	-4.4278209245
O	-2.0476423677	0.1669244872	-3.1008575229
C	-3.2876337619	-1.7709626718	-4.4734511052
C	-3.6553221700	-3.0573219548	-5.1639280018
C	-4.3547275778	-3.0417927012	-6.3743742436
C	-4.7150387600	-4.2356027023	-7.0034915477
C	-4.3694206999	-5.4582742696	-6.4286054074
C	-3.6649640089	-5.4834475111	-5.2214183218
C	-3.3141577711	-4.2908451535	-4.5923394341
H	-1.0587530321	0.0663462198	-0.9429173884
H	-1.6665978973	-0.4990803351	1.3892667653
H	0.0361024187	-1.3703123110	2.9715535895
H	2.3906499457	-1.6798980648	2.2255814408
H	0.0698506773	1.0740249685	-2.6603077150

H	1.2259869593	1.2850751613	-4.9255205928
H	3.7647268026	0.8914628069	-4.7731096349
H	3.1246373573	-2.5315316013	-4.0319862827
H	2.6144359787	-4.2632754012	-5.7316769921
H	1.9916621068	-3.5964322677	-8.0460479796
H	1.8858617012	-1.1805250443	-8.6402670015
H	2.3692316739	0.5383521756	-6.9374174833
H	2.8231507121	3.0014601062	-4.0995870015
H	1.3166339745	3.0685371845	-3.1707317530
H	2.7645464864	2.3222277638	-2.4690397011
H	0.4733382951	-1.3667161109	-4.2750818077
H	-3.6671561788	-1.7331878840	-3.4475616873
H	-3.6845464778	-0.9016861945	-5.0066167175
H	-4.6199806211	-2.0894933070	-6.8278346476
H	-5.2611768831	-4.2083648244	-7.9426063804
H	-4.6471171778	-6.3886695979	-6.9167280721
H	-3.3937543356	-6.4336554823	-4.7690687705
H	-2.7655519795	-4.3114843039	-3.6539856229

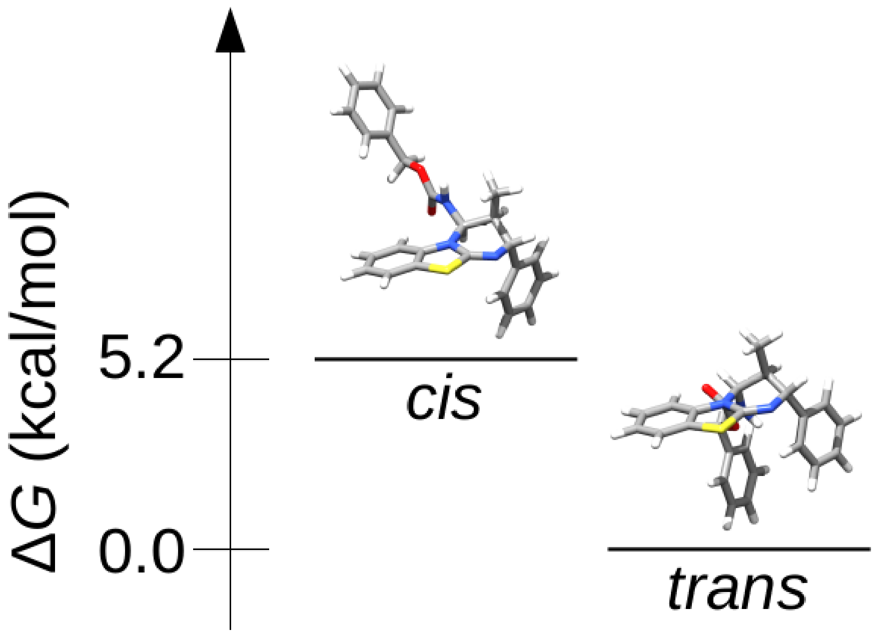

VIII. X-Ray data of compounds 4k and 11

X-ray Structure determination of **4k** was performed on a colorless acicular crystal grown from slow evaporation of dichloromethane solvent, using a Rigaku diffractometer constituted by a MM007 HF rotating-anode generator, delivering copper radiation through Osmic CMF confocal optics, and a Rapid II curved Image Plate detector. Data collection and reduction were carried out with softwares implemented in CrystalClear 2.0 like Fs_process.[8] The structure was solved by intrinsic phasing methods[9] and refined by full-matrix least-squares methods[10] on 313 parameters, using the following weighted refinement scheme: $w = 1/[\sigma^2(F_o^2) + (0.0330P)^2+3.6724P]$ with $P = [\max(F_o^2,0) + 2F_c^2]/3$. Non-hydrogen atoms were improved by anisotropic refinement whereas hydrogen atoms were located from difference Fourier synthesis and refined isotropically assuming a riding motion model for those borne by carbon atoms, with C – H distances of 0.94 Å (aromatic), 0.97 (Å) (-CH_3), 0.98 Å (-CH_2), 0.99 Å (-CH) and with $U_{iso}(H) = 1.2U_{eq}(C, N)$ or 1.5Ueq (C_{methyl}). A restraint was applied to the bond distance N-H, 0.89(3) Å. Crystal data, data collection and structure refinement details are summarized in the table 1 below. Given the enantiopurity of the compound, the assignment of the benzyl ((2*R*,3*S*,4*R*)-2-(4-bromophenyl)-8-methoxy-3-methyl-3,4-dihydro-2*H*benzo[4,5]thiazolo[3,2-*a*]pyramidin-4-yl)carbamate was unambiguoulsy determined by the X-ray data through the refined value as a 2-component twin of the Flack[11] parameter -0.024(11) or the Hooft[12] parameter 0.045(13) derived from Bayesian analysis of the 1963 Bijvoet pairs.

Good-quality colorless acicular crystals of **11** were obtained by slow evaporation of ethyl acetate. The crystal chosen was attached to the tip of a 200µm nylon loop with paratone-N oil. X-ray diffraction measurements were carried out using a Rigaku XtaLabPro diffractometer equipped with a microfocus source (MicroMax003) delivering Mo Kα radiation, λ = 0.71073 Å through Rigaku confocal Max-Flux® multilayer double bounce optic. The initial crystal orientation and unit cell were indexed using a least-squares analysis of a random set of reflections collected from three series of 0.5° ω-scans, 30 seconds per frame and 10 frames per series, that were distributed in reciprocal space. For complete redundant (≥5) dataset collection optimising Friedel pair measurements, seven ω-scan frame series were collected with 0.5° wide scans, thirty second frames for a total of 1156 frames at varying κ (38, 47, and 57°) and φ (-144, -60, 0 , 30, 60, and 120°) angles. The crystal-to-detector distance was set to 2.9 cm. Cell refinement and data reduction were performed using CrysalisPro,[4] which corrects for Lorentz and polarization effects in particular and applies a multi-scan absorption correction (SCALE3 ABSPACK scaling algorithm). The structure was solved by intrinsic phasing methods with the ShelXT program[5] and refined using a full-matrix least-squares method on F^2 with the ShelXL.[6] All non-hydrogen atoms were refined with anisotropic displacement parameters and H atoms, although located in residual density, were included at geometrically idealized positions and treated as riding on their parent atoms. The isotropic thermal parameters of the hydrogen atoms were fixed at 1.2Ueq of the parent carbon of 1.5Ueq for methyl ones. C19 methyl appeared disordered with two sites rotated by 60 degrees from one another. The corresponding site occupation factors were refined via a 'free variable' so that their sum is unity (0.53/0.47) and the recommended HFIX123 riding model was employed. The absolute structure of **11** was characterized as ((2*R*,3*S*,4*S*)-2-(4-bromophenyl)-3,8-dimethyl-3,4-dihydro-2*H*-benzo[4,5]thiazolo[3,2-*a*]pyrimidin-4-ol with an non-ambiguous absolute structure Flack parameter,[7] x = -0.006(7) using 1072 quotients [(I+)-(I-)]/[(I+)+(I-)], from 100% complete Bijvoet pair coverage.

---

[8] Rigaku, CrystalClear-SM Expert 2.0 r4, Rigaku Corporation, Tokyo, Japan (**2009**).
[9] Sheldrick, G. M. *Acta Crystallogr. A* **2015,** *71*, 3.
[10] Sheldrick, G. M. *Acta Crystallogr. C* **2015,** *71*, 3.
[11] Flack, H.D. *Acta Cryst. A* **1983**, *39*, 876.
[12] Hooft, R.W.W.; Straver, L.H.; Spek, A.L. *J. Appl. Cryst.* **2008**, *41*, 96.

- **4k**

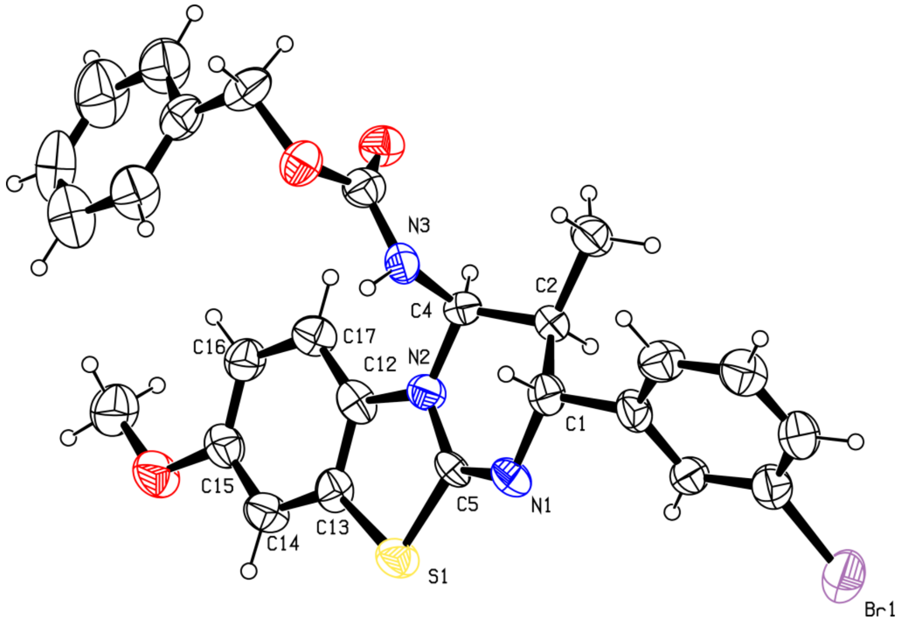

Figure S1. ORTEP-style plot of **4k**. Ellipsoids are drawn at the 30% probability level and H atoms are shown as spheres of arbitrary radius.

- **11**

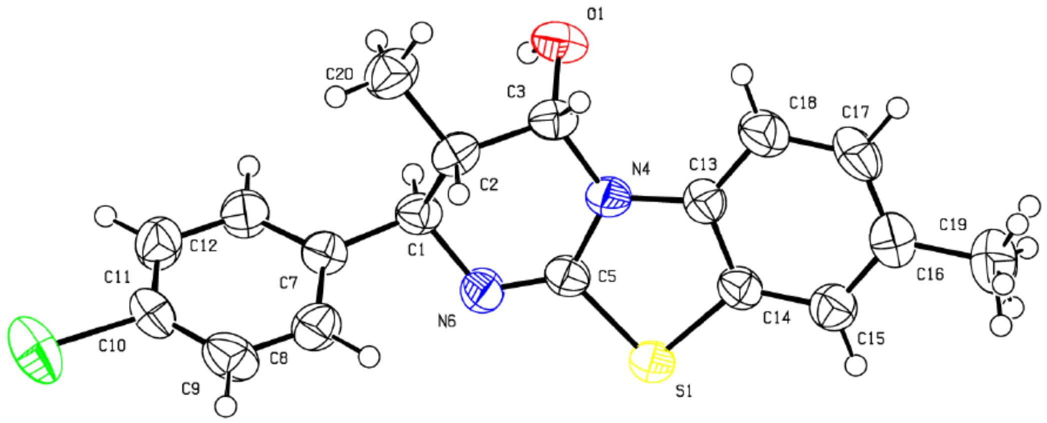

Figure S2. ORTEP-style plot of **11**. Ellipsoids are drawn at the 30% probability level and H atoms are shown as spheres of arbitrary radius.

Crystallographic data for **4k** and **11** have been deposited with the Cambridge Crystallographic Data Centre (deposit no. CCDC 1815484 and 1851627). These data can be obtained, free of charge, from The Cambridge Crystallographic Data Centre via www.ccdc.cam.ac.uk/data_request/cif.

Table S2. Crystal data and structure refinement for **4k**

Scheme

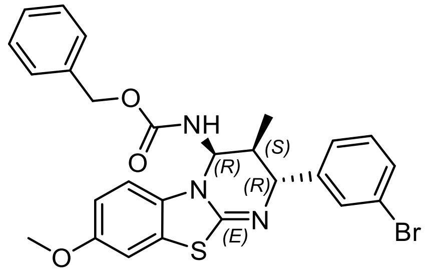

Identification code	**4k**	
Empirical formula	$C_{26}H_{24}BrN_3O_3S$	
Formula weight	538.45	
Temperature	213(2) K	
Wavelength	1.54187 Å	
Crystal system	Hexagonal	
Space group	$P6_3$	
Unit cell dimensions	a = 17.660(2) Å	α = 90°.
	b = 17.660(2) Å	β = 90°.
	c = 13.5994(11) Å	γ = 120°.
Volume	3673.1(9) $Å^3$	
Z 6		
Density (calculated)	1.461 Mg/m^3	
Absorption coefficient	3.358 mm^{-1}	
F(000)	1656	
Crystal size	0.38 x 0.05 x 0.03 mm^3	
Theta range for data collection	2.889 to 68.235°.	
Index ranges	$-21 \leq h \leq 11$, $-18 \leq k \leq 18$, $-16 \leq l \leq 13$	
Reflections collected	15330	
Independent reflections	4302 [R(int) = 0.0531]	
Completeness to theta = 67.687°	99.5 %	
Absorption correction	Semi-empirical from equivalents	
Max. and min. transmission	1.000 and 0.670	
Refinement method	Full-matrix least-squares on F^2	
Data / restraints / parameters	4300 / 38 / 313	
Goodness-of-fit on F^2	1.118	
Final R indices [I>2σ(I)]	R1 = 0.0556, wR2 = 0.0966	
R indices (all data)	R1 = 0.1239, wR2 = 0.1477	
Absolute structure parameter	-0.024(11)	
Largest diff. peak and hole	0.370 and -0.537 e.$Å^{-3}$	
CCDC deposit number	1815484	

Table S3. Atomic coordinates (x 10^4) and equivalent isotropic displacement parameters ($Å^2$ x 10^3) for **4k**. U(eq) is defined as one third of the trace of the orthogonalized U^{ij} tensor.

	x	y	z	U(eq)
C(1)	4029(8)	8212(8)	3140(9)	66(3)
O(1)	5152(6)	8826(6)	9080(7)	92(3)
N(1)	3435(6)	7977(6)	4011(7)	64(3)
S(1)3221(2)	7901(2)	5965(3)	72(1)	
Br(1)	1611(1)	8758(1)	1458(2)	125(1)
N(2)	4690(6)	8708(6)	5038(7)	59(3)
O(2)	7128(5)	9638(5)	4483(6)	72(2)
C(2)	4871(7)	9096(7)	3322(8)	57(3)
N(3)	5735(6)	8512(6)	4138(7)	60(2)
O(3)	6824(5)	8225(5)	4315(6)	75(2)
C(4)	5327(7)	9054(8)	4251(9)	60(3)
C(5)	3812(7)	8208(7)	4867(8)	52(3)
C(6)	3561(9)	8233(8)	2224(10)	68(3)
C(7)	2905(8)	8448(8)	2258(9)	64(3)
C(8)	2507(8)	8488(8)	1378(12)	76(3)
C(9)	2751(10)	8331(10)	498(12)	90(4)
C(10)	3378(10)	8076(10)	452(11)	88(5)
C(11)	3777(8)	8038(8)	1323(11)	77(4)
C(12)	4928(9)	8820(7)	6065(8)	61(3)
C(13)	4173(7)	8394(7)	6644(9)	63(3)
C(14)	4268(9)	8399(8)	7653(10)	74(4)
C(15)	5110(10)	8840(9)	8040(10)	74(4)
C(16)	5844(8)	9239(8)	7471(9)	72(4)
C(17)	5759(7)	9236(6)	6437(11)	67(3)
C(18)	5995(9)	9330(10)	9541(11)	101(5)
C(19)	5514(8)	9418(8)	2438(9)	75(4)
C(21)	6602(9)	8858(10)	4319(9)	69(3)
C(22)	7723(9)	8518(9)	4651(12)	89(5)
C(24)	7171(10)	7993(9)	6353(13)	101(5)
C(25)	7257(13)	8065(12)	7382(13)	116(6)
C(26)	8550(14)	9360(14)	7222(15)	142(8)
C(27)	8505(11)	9311(10)	6196(13)	112(5)
C(28)	7791(10)	8608(9)	5755(12)	78(4)
C(30)	7950(16)	8765(13)	7819(15)	128(7)

Table S4. Bond lengths [Å] and angles [°] for **4k**.

C(1)-N(1)	1.497(14)	C(12)-N(2)-C(4)	123.1(10)
C(1)-C(6)	1.507(17)	C(4)-C(2)-C(1)	110.5(9)
C(1)-C(2)	1.545(15)	C(4)-C(2)-C(19)	110.7(9)
O(1)-C(15)	1.418(15)	C(1)-C(2)-C(19)	113.9(10)
O(1)-C(18)	1.440(15)	C(21)-N(3)-C(4)	119.9(10)
N(1)-C(5)	1.301(13)	C(21)-O(3)-C(22)	114.4(10)
S(1)-C(13)	1.724(11)	N(2)-C(4)-N(3)	109.5(9)
S(1)-C(5)	1.745(11)	N(2)-C(4)-C(2)	108.4(9)
Br(1)-C(8)	1.870(12)	N(3)-C(4)-C(2)	112.9(10)
N(2)-C(5)	1.367(13)	N(1)-C(5)-N(2)	126.3(10)
N(2)-C(12)	1.443(14)	N(1)-C(5)-S(1)	122.3(9)
N(2)-C(4)	1.449(13)	N(2)-C(5)-S(1)	111.3(9)
O(2)-C(21)	1.238(14)	C(11)-C(6)-C(7)	118.1(13)
C(2)-C(4)	1.520(15)	C(11)-C(6)-C(1)	120.3(12)
C(2)-C(19)	1.552(14)	C(7)-C(6)-C(1)	121.6(12)
N(3)-C(21)	1.358(14)	C(6)-C(7)-C(8)	119.4(13)
N(3)-C(4)	1.467(14)	C(9)-C(8)-C(7)	121.7(12)
O(3)-C(21)	1.357(14)	C(9)-C(8)-Br(1)	120.2(12)
O(3)-C(22)	1.475(14)	C(7)-C(8)-Br(1)	118.1(11)
C(6)-C(11)	1.378(17)	C(8)-C(9)-C(10)	119.8(14)
C(6)-C(7)	1.388(16)	C(9)-C(10)-C(11)	118.7(15)
C(7)-C(8)	1.407(16)	C(6)-C(11)-C(10)	122.2(13)
C(8)-C(9)	1.346(18)	C(17)-C(12)-C(13)	123.9(12)
C(9)-C(10)	1.39(2)	C(17)-C(12)-N(2)	126.3(12)
C(10)-C(11)	1.397(18)	C(13)-C(12)-N(2)	109.7(10)
C(12)-C(17)	1.367(15)	C(14)-C(13)-C(12)	118.3(11)
C(12)-C(13)	1.401(15)	C(14)-C(13)-S(1)	128.3(10)
C(13)-C(14)	1.382(16)	C(12)-C(13)-S(1)	113.3(9)
C(14)-C(15)	1.392(17)	C(13)-C(14)-C(15)	118.1(12)
C(15)-C(16)	1.364(16)	C(16)-C(15)-C(14)	123.3(13)
C(16)-C(17)	1.414(18)	C(16)-C(15)-O(1)	122.0(12)
C(22)-C(28)	1.51(2)	C(14)-C(15)-O(1)	114.7(12)
C(24)-C(28)	1.361(19)	C(15)-C(16)-C(17)	119.3(12)
C(24)-C(25)	1.41(2)	C(12)-C(17)-C(16)	117.0(12)
C(25)-C(30)	1.37(2)	O(2)-C(21)-O(3)	123.7(12)
C(26)-C(30)	1.33(3)	O(2)-C(21)-N(3)	125.6(13)
C(26)-C(27)	1.40(2)	O(3)-C(21)-N(3)	110.7(12)
C(27)-C(28)	1.388(18)	O(3)-C(22)-C(28)	111.2(11)
N(1)-C(1)-C(6)	110.1(10)	C(28)-C(24)-C(25)	121.0(17)
N(1)-C(1)-C(2)	109.9(10)	C(30)-C(25)-C(24)	121.5(19)
C(6)-C(1)-C(2)	111.7(10)	C(30)-C(26)-C(27)	124(2)
C(15)-O(1)-C(18)	118.0(11)	C(28)-C(27)-C(26)	119.0(18)
C(5)-N(1)-C(1)	116.3(9)	C(24)-C(28)-C(27)	117.7(16)
C(13)-S(1)-C(5)	91.2(6)	C(24)-C(28)-C(22)	121.4(14)
C(5)-N(2)-C(12)	114.3(10)	C(27)-C(28)-C(22)	120.9(15)
C(5)-N(2)-C(4)	122.5(10)	C(26)-C(30)-C(25)	117(2)

Symmetry transformations used to generate equivalent atoms:

Table S5. Anisotropic displacement parameters ($Å^2$ x 10^3) for **4k**. The anisotropic displacement factor exponent takes the form : $-2\pi^2$ [$h^2 a^{*2}U^{11}$ + ... + 2 h k a* b* U^{12}]

	U^{11}	U^{22}	U^{33}	U^{23}	U^{13}	U^{12}
C(1)	73(8)	76(8)	50(8)	2(6)	-4(7)	38(7)
O(1)	89(7)	114(7)	51(6)	3(5)	-5(5)	34(6)
N(1)	72(7)	69(6)	45(6)	6(5)	12(5)	31(5)
S(1)	66(2)	84(2)	53(2)	2(2)	3(2)	28(2)
Br(1)	122(1)	186(2)	104(1)	35(2)	4(1)	104(1)
N(2)	52(6)	62(6)	54(7)	4(5)	2(5)	21(5)
O(2)	64(5)	76(6)	68(6)	-9(5)	-3(4)	29(5)
C(2)	56(7)	60(7)	52(8)	12(6)	6(6)	27(6)
N(3)	71(7)	61(6)	53(6)	9(5)	3(5)	37(6)
O(3)	80(6)	77(5)	78(7)	-9(5)	-7(5)	48(5)
C(4)	45(6)	70(8)	61(9)	8(6)	3(6)	26(6)
C(5)	65(7)	56(7)	30(7)	6(5)	12(6)	27(6)
C(6)	75(8)	74(8)	46(8)	9(7)	-3(7)	32(7)
C(7)	60(7)	80(8)	52(8)	1(7)	1(6)	36(7)
C(8)	78(8)	72(8)	71(10)	5(8)	-6(9)	32(7)
C(9)	96(12)	117(13)	60(10)	10(9)	-5(8)	54(10)
C(10)	86(10)	99(11)	58(10)	-11(8)	-9(8)	32(9)
C(11)	73(8)	94(9)	63(10)	-10(8)	2(8)	40(7)
C(12)	84(8)	51(7)	44(8)	-8(5)	2(7)	31(7)
C(13)	69(8)	59(7)	47(8)	-11(7)	-19(6)	23(6)
C(14)	66(8)	71(9)	67(10)	2(7)	5(7)	21(7)
C(15)	85(10)	80(9)	57(9)	6(8)	4(8)	42(8)
C(16)	67(8)	82(9)	64(9)	-5(7)	-10(7)	35(7)
C(17)	63(7)	57(7)	66(8)	-10(7)	-3(7)	19(6)
C(18)	97(11)	122(12)	77(10)	-13(9)	-23(9)	50(10)
C(19)	69(8)	79(9)	61(10)	10(7)	2(7)	25(7)
C(21)	67(8)	87(9)	54(8)	-6(7)	2(7)	39(8)
C(22)	67(9)	96(11)	117(14)	-7(10)	-7(9)	51(8)
C(24)	109(10)	84(9)	93(11)	-10(9)	-19(9)	35(8)
C(25)	168(16)	106(12)	90(11)	0(9)	-5(11)	80(11)
C(26)	158(18)	133(16)	112(14)	-24(11)	-57(13)	55(12)
C(27)	106(11)	94(10)	117(13)	-2(9)	-24(10)	35(8)
C(28)	78(9)	70(8)	98(11)	-9(7)	-9(7)	48(7)
C(30)	190(20)	118(14)	109(15)	-8(10)	-52(11)	101(14)

Table S6. Hydrogen coordinates (x 10^4) and isotropic displacement parameters (Å^2 x 10^3) for **4k**.

	x	y	z	U(eq)
H(1)	4194	7755	3058	79
H(2)	4691	9537	3435	68
H(3N)	5480(60)	7930(20)	4220(80)	72
H(4)	5786	9655	4428	72
H(7)	2727	8565	2863	77
H(9)	2499	8394	-82	108
H(10)	3530	7932	-154	105
H(11)	4207	7873	1293	93
H(14)	3778	8112	8066	89
H(16)	6401	9513	7764	87
H(17)	6251	9507	6024	80
H(18L)	6234	9944	9384	152
H(18M)	6386	9135	9301	152
H(18P)	5931	9251	10248	152
H(19H)	6045	9949	2621	113
H(19J)	5244	9538	1884	113
H(19K)	5653	8971	2258	113
H(22D)	8125	9082	4348	106
H(22F)	7895	8095	4441	106
H(24)	6677	7513	6075	122
H(25)	6828	7622	7778	140
H(26)	9034	9845	7508	170
H(27)	8952	9747	5810	135
H(30)	8001	8823	8507	154

Table S7. Torsion angles [°] for **4k**.

C(6)-C(1)-N(1)-C(5)	-158.1(10)
C(2)-C(1)-N(1)-C(5)	-34.7(13)
N(1)-C(1)-C(2)-C(4)	57.4(12)
C(6)-C(1)-C(2)-C(4)	179.9(10)
N(1)-C(1)-C(2)-C(19)	-177.3(10)
C(6)-C(1)-C(2)-C(19)	-54.8(14)
C(5)-N(2)-C(4)-N(3)	-100.8(11)
C(12)-N(2)-C(4)-N(3)	75.7(13)
C(5)-N(2)-C(4)-C(2)	22.7(14)
C(12)-N(2)-C(4)-C(2)	-160.7(9)
C(21)-N(3)-C(4)-N(2)	-115.5(11)
C(21)-N(3)-C(4)-C(2)	123.6(11)
C(1)-C(2)-C(4)-N(2)	-50.0(13)
C(19)-C(2)-C(4)-N(2)	-177.1(9)
C(1)-C(2)-C(4)-N(3)	71.5(12)
C(19)-C(2)-C(4)-N(3)	-55.6(13)
C(1)-N(1)-C(5)-N(2)	6.4(17)
C(1)-N(1)-C(5)-S(1)	-174.0(8)
C(12)-N(2)-C(5)-N(1)	-176.7(11)
C(4)-N(2)-C(5)-N(1)	0.1(17)
C(12)-N(2)-C(5)-S(1)	3.7(11)
C(4)-N(2)-C(5)-S(1)	-179.5(8)
C(13)-S(1)-C(5)-N(1)	176.8(10)
C(13)-S(1)-C(5)-N(2)	-3.6(8)
N(1)-C(1)-C(6)-C(11)	-150.4(12)
C(2)-C(1)-C(6)-C(11)	87.3(14)
N(1)-C(1)-C(6)-C(7)	29.6(16)
C(2)-C(1)-C(6)-C(7)	-92.8(14)
C(11)-C(6)-C(7)-C(8)	-2.2(18)
C(1)-C(6)-C(7)-C(8)	177.8(11)
C(6)-C(7)-C(8)-C(9)	-0.6(19)
C(6)-C(7)-C(8)-Br(1)	178.8(9)
C(7)-C(8)-C(9)-C(10)	4(2)
Br(1)-C(8)-C(9)-C(10)	-175.8(11)
C(8)-C(9)-C(10)-C(11)	-4(2)
C(7)-C(6)-C(11)-C(10)	2(2)
C(1)-C(6)-C(11)-C(10)	-178.0(13)
C(9)-C(10)-C(11)-C(6)	1(2)
C(5)-N(2)-C(12)-C(17)	175.2(10)
C(4)-N(2)-C(12)-C(17)	-1.6(17)
C(5)-N(2)-C(12)-C(13)	-1.7(12)
C(4)-N(2)-C(12)-C(13)	-178.4(10)
C(17)-C(12)-C(13)-C(14)	0.6(18)
N(2)-C(12)-C(13)-C(14)	177.6(10)
C(17)-C(12)-C(13)-S(1)	-178.1(9)

N(2)-C(12)-C(13)-S(1)	-1.1(11)
C(5)-S(1)-C(13)-C(14)	-175.9(12)
C(5)-S(1)-C(13)-C(12)	2.7(9)
C(12)-C(13)-C(14)-C(15)	1.1(18)
S(1)-C(13)-C(14)-C(15)	179.6(10)
C(13)-C(14)-C(15)-C(16)	-2(2)
C(13)-C(14)-C(15)-O(1)	179.1(11)
C(18)-O(1)-C(15)-C(16)	6.7(19)
C(18)-O(1)-C(15)-C(14)	-174.8(13)
C(14)-C(15)-C(16)-C(17)	2(2)
O(1)-C(15)-C(16)-C(17)	-179.5(11)
C(13)-C(12)-C(17)-C(16)	-1.0(17)
N(2)-C(12)-C(17)-C(16)	-177.4(10)
C(15)-C(16)-C(17)-C(12)	-0.4(18)
C(22)-O(3)-C(21)-O(2)	7.7(17)
C(22)-O(3)-C(21)-N(3)	-171.3(10)
C(4)-N(3)-C(21)-O(2)	-8.0(18)
C(4)-N(3)-C(21)-O(3)	171.0(9)
C(21)-O(3)-C(22)-C(28)	76.4(13)
C(28)-C(24)-C(25)-C(30)	2(3)
C(30)-C(26)-C(27)-C(28)	1(3)
C(25)-C(24)-C(28)-C(27)	-1(2)
C(25)-C(24)-C(28)-C(22)	177.5(14)
C(26)-C(27)-C(28)-C(24)	-1(2)
C(26)-C(27)-C(28)-C(22)	-178.8(15)
O(3)-C(22)-C(28)-C(24)	43.4(16)
O(3)-C(22)-C(28)-C(27)	-138.6(13)
C(27)-C(26)-C(30)-C(25)	0(3)
C(24)-C(25)-C(30)-C(26)	-2(3)

Symmetry transformations used to generate equivalent atoms:

Table S8. Hydrogen bonds for **4k** [Å and °].

D-H...A	d(D-H)	d(H...A)	d(D...A)	<(DHA)
N(3)-H(3N)...N(1)#1	0.89(3)	2.16(5)	3.007(13)	158(10)
N(3)-H(3N)...S(1)#1	0.89(3)	2.98(8)	3.649(9)	133(9)
C(7)-H(7)...O(3)#2	0.94	2.61	3.523(15)	162.9
C(17)-H(17)...O(2)	0.94	2.55	3.419(16)	154.6

Symmetry transformations used to generate equivalent atoms:

#1 -x+y,-x+1,z   #2 -y+1,x-y+1,z

Table S9. Crystal data and structure refinement for **11**

Scheme	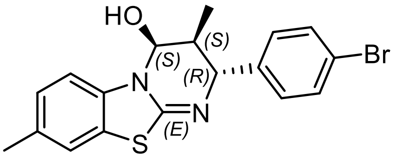	
Identification code	**11**	
Empirical formula	$C_{18}H_{17}BrN_2OS$	
Formula weight	389.30	
Temperature	293(2) K	
Wavelength	0.71073 Å	
Crystal system	Monoclinic	
Space group	P 21	
Unit cell dimensions	a = 9.6687(7) Å	α = 90°.
	b = 8.1636(5) Å	β = 104.070 (7)°.
	c = 11.2423(8) Å	γ = 90°.
Volume	860.75(11) $Å^3$	
Z 2		
Density (calculated)	1.502 Mg/m^3	
Absorption coefficient	2.814 mm^{-1}	
F(000)	396	
Crystal size	0.17 x 0.05 x 0.05 mm^3	
Theta range for data collection	3.531 to 25.999°	
Index ranges	$-11 \leq h \leq 11$, $-10 \leq k \leq 10$, $-13 \leq l \leq 13$	
Reflections collected	17429	
Independent reflections	3379 [R(int) = 0.0684]	
Completeness to theta = 25.242°	99.6 %	
Absorption correction	Semi-empirical from equivalents	
Max. and min. transmission	1.00000 and 0.36373	
Refinement method	Full-matrix least-squares on F^2	
Data / restraints / parameters	3378 / 1 / 215	
Goodness-of-fit on F2	1.024	
Final R indices [I>2σ(I)]	R1 = 0.0418, wR2 = 0.0829	
R indices (all data)	R1 = 0.0544, wR2 = 0.0874	
Absolute structure parameter	-0.006(7)	
Largest diff. peak and hole	0.433 and -0.354 e.$Å^{-3}$	
CCDC deposit number	1851627	
